# ﻿An integrative approach to alpha taxonomy in *Erica* L. (Ericaceae) with three new species from the Western Cape, South Africa

**DOI:** 10.3897/phytokeys.257.139457

**Published:** 2025-06-04

**Authors:** Rendert D. Hoekstra, Seth D. Musker, Michael D. Pirie, Jan H. J. Vlok

**Affiliations:** 1 Department of Botany and Zoology, Stellenbosch University, Private Bag X1, Matieland 7602, South Africa Stellenbosch University Matieland South Africa; 2 Department of Biological Sciences, University of Cape Town, Private Bag, Rondebosch 7701, South Africa University of Cape Town Rondebosch South Africa; 3 University Museum, University of Bergen, Postboks 7800, NO-5020 Bergen, Norway University of Bergen Bergen Norway; 4 Department of Botany, Nelson Mandela University, P.O. Box 77000, Port Elizabeth, 6031, South Africa Nelson Mandela University Port Elizabeth South Africa

**Keywords:** Cape Floristic Region, Global Conservation Consortium for *Erica*, phylogeny, species diversity, threat status

## Abstract

The megagenus *Erica* L. (Ericaceae) comprises 851 species across its global distribution, with an extraordinary focus of diversity in the Cape Floristic Region (CFR) of South Africa where almost 700 species are endemic. The genus is remarkable for both its morphological diversity and the large number of species and subspecific taxa occurring in small populations, often in specialised habitats, putting them at high risk of extinction. Despite significant taxonomic work over the past century, part of this diversity remains undescribed. The sheer size of the genus, its morphological, ecological and geographical variability, and the absence of a modern, consolidated revision make alpha taxonomy challenging. By combining traditional taxonomic methods, standard DNA sequencing methods building on openly available data matrices, and an openly available specialised taxonomic tool for the genus, we present an integrative, reproducible approach to alpha taxonomy in *Erica*. This approach provided support for the recognition of three new species from the Western Cape in South Africa and aided in ruling out two further putative new species, confirming one as a natural hybrid and the other as a morphological variation within an existing species. We describe the three new species *Ericaarida* R.D.Hoekstra, *Ericahessequae* R.D.Hoekstra and *Ericainopina* J.H.J.Vlok.

## ﻿Introduction

The megadiverse genus *Erica* L. (Ericaceae) forms an extraordinary focus of diversity in the Cape Floristic Region (CFR) of South Africa, where nearly 700 of its 851 species are found, almost all of which are regionally endemic (Elliott et al. 2024). This reflects the overall high levels of diversity and endemism in the CFR (Goldblatt and Manning 2000; Linder, 2003). Its sheer size and taxonomic complexity make *Erica* challenging to work with. It has been the subject of substantial taxonomic work, from the foundational treatment of the South African species by Guthrie and Bolus in *Flora Capensis* (1905) and the comprehensive revision of Dulfer (1964, 1965), to works focused on particular regions and groups (Oliver 2000; Oliver and Oliver 2002, 2005; Beentje 2006; Nelson 2011). However, these represent fragmented and in part outdated taxonomic accounts. Morphological variation in *Erica* is complex, and the task of distinguishing known and unknown diversity has become increasingly challenging. Undescribed taxa may be effectively hidden within species complexes that have only recently begun to be resolved with the aid of molecular studies (e.g. Pirie et al. 2017; Musker et al. 2024).

Many species of *Erica* are infrequently encountered or collected because of their natural rarity, ongoing decline in species populations, and tendency to occur in remote and inaccessible mountainous areas. Many are also highly localised endemics, some restricted to areas as small as a few hundred m^2^, and many of these, such as *E.burchelliana* E.G.H.Oliv., *E.chrysocodon* Guthrie & Bolus, *E.extrusa* Compton, *E.feminarum* E.G.H.Oliv., *E.gerhardii* E.G.H.Oliv. & I.M.Oliv., *E.heleogena* T.M.Salter, *E.hillburttii* (E.G.H.Oliv.) E.G.H.Oliv., *E.karwyderi* E.G.H.Oliv., *E.petrusiana* E.G.H.Oliv. & I.M.Oliv., *E.perplexa* E.G.H.Oliv., *E.remota* (N.E.Br.) E.G.H.Oliv., and *E.sociorum* L.Bolus, are critically endangered (Raimondo et al. 2009). Their specialised niches and highly restricted geographical distributions place them at high risk of extinction. According to the International Union for Conservation of Nature (IUCN) Red List definitions, more than 150 species and subspecies can be considered threatened (Raimondo et al. 2009); however, most assessments fall outside the 10-year threshold specified by IUCN, they leave many as Data Deficient or unassessed, and they cannot address undescribed species’ diversity (Pirie et al. 2024). The latter is particularly critical as undescribed taxa are more likely to be threatened with extinction in the wild (Brown et al. 2023). Our research, and specimens collected and curated by the pre-eminent expert on the genus E.G.H. Oliver (Nelson et al. 2024) prior to the cessation of his active work, point to a significant remaining shortfall in the documentation of *Erica* alpha diversity. New species descriptions need to be prioritised as the first step towards justifying targeted conservation of these taxa.

Most of the taxonomic work on Cape *Erica* has been limited to the prolific output of few, largely successively working specialists, with the majority of recent work having been performed over much of the 20^th^ and into the 21^st^ century by the South African botanists E.G.H. and I.M. Oliver (Nelson et al. 2024). Sadly, they are no longer with us, and in the absence of a modern, consolidated revision it is hard even for experts to identify novelties amongst the enormous species diversity of a complex group like *Erica*. Fortunately, an overview of accepted taxa and variation in morphological characters is provided by ‘Genus *Erica*: an Identification Aid (the *Erica* ID aid; Oliver et al. 2024a) Version 4.00.02 (https://doi.org/10.5281/zenodo.10651955). The *Erica* ID aid offers a means by which to identify the most similar taxa to a given specimen, even when it may represent an undescribed taxon, making this tool an accessible means to aid species identification and guide alpha taxonomy.

A further resource for comparing known variation is the body of published DNA sequence data, which represents over 65% of the recognised species of *Erica* (Pirie et al. 2024). Despite limited resolution of phylogenetic relationships, particularly within the Cape clade, a researcher may be able to identify the closest relatives of putative new species or to discern them from morphologically similar distant relatives with the use of standard protocols and widely used DNA sequence regions such as the nuclear ribosomal Internal Transcribed Spacers (ITS) and plastid trnF-ndhJ intergenic spacer.

In this paper, we assess five examples of apparently undescribed variation in *Erica*. In each case, we used the *Erica* ID aid to generate lists of most similar known taxa. To test the relatedness of the putative new taxa, we collected and analysed new DNA sequence data, including data from new collections of similar and potentially related known taxa. We then assessed potential diagnostic differences between focal specimens and known taxa, concurrently implementing improvements to the *Erica* ID aid. Where the results support the recognition of new taxa, we describe them formally. Using this combined approach, we describe three new *Erica* species from the Eastern Langeberg and Karoo Mountain regions in the Western Cape, South Africa, presenting diagnoses and descriptions, as well as distributions, photos and threat status.

## ﻿Materials and methods

### ﻿Field research and collection

Collections of potentially undescribed taxa were made in the field under research permits from Cape Nature (CN35-28-27705) and permission from private landowners. Herbarium vouchers were lodged at NBG with some duplicates at BG (acronyms after Thiers 2018), and fresh leaf samples were dried in silica gel for DNA extraction. Details of habitat and ecology were recorded in the field and, where possible, photographic records were taken of specimens in their habitat and of floral and vegetative parts. Limited surveys were performed in surrounding localities to assess the extent of distributions. Additional targeted surveys were conducted to locate further subpopulations. We used GeoCAT (https://geocat.iucnredlist.org) to calculate an Extent of Occurrence (EOO) and Area of Occupancy (AOO) for each taxon.

### ﻿Morphological comparisons using the *Erica* ID aid

We performed character-based searches using the *Erica* ID aid. The key features a strict “Match” and a more flexible “Probability” algorithm for narrowing down species identifications. The “Match” algorithm provides a list of only those taxa with characteristics that are consistent with those selected, while considering intra-specific character variability. For example, under the character “Corolla Size”, when “1–5 mm” and “5–10 mm” are selected, all species which have corollas of size 1–5 mm and/or 5–10 mm are possible identifications. When we processed species using this feature, results were recorded for three scenarios: “region and flowering month excluded”; “region included and flowering month excluded”, and “all characters included”. This was done to further minimise errors as it accounted for variability in geographic range, which might have been underestimated in previous records of recognised species, and flowering times, which might similarly be longer than previously recognised or have changed over time.

The “Probability” algorithm provides a complete list of taxa ordered by probability, whereby rarer characteristics impart higher probabilities than more common ones and narrow variation imparts higher probabilities than wide. The most probable results can therefore include taxa that are excluded altogether when using the strict “Match” algorithm and strict matches may score as less probable if characters are coded to multiple states (Oliver et al. 2024b). Both sets of results can be reproduced with the same character inputs presented in the Results with the same (permanently archived) version of the *Erica* ID aid.

### ﻿Identifying closely related taxa using phylogenetic inference

To identify close relatives of the putative new taxa and potentially exclude morphologically similar but distantly related species from considerations of species boundaries, we sequenced ITS and trnF-ndhJ, markers used in the most comprehensive phylogenetic analyses of *Erica* (Pirie et al. 2016, 2024) and reanalysed the available data. We rarely had access to multiple accessions of the same taxon; where only one individual was found, we sequenced two samples of the same individual that had been collected on separate occasions to confirm the result. DNA was isolated with the DNeasy Plant Mini Kit (Qiagen, Hilden, Germany) followed by PCR amplification with universal angiosperm primers (Taberlet et al. 1991; Shaw et al. 2007) as in Mugrabi de Kuppler et al. (2015).

In most cases, Sanger sequencing delivered unambiguously interpretable sequences. In one case (*Erica* sp. RDH184), the resulting ITS chromatograms showed clear double peaks of similar heights at 12 informative sites. Plastid data indicated a sister group relationship to the morphologically similar species *E.peltata* Andrews (see Results). At each polymorphic site in the ITS sequence we observed that one of the two overlapping peaks corresponded to the same base observed in *E.peltata*. Under the assumption that this pattern reflected inheritance from one of two distinct parental species, we recreated two separate ITS sequences, one with *E.peltata* base variants and the other with the unknown alternative variants.

New sequences were added manually to the alignments of ITS and of trnF-ndhJ sequences in Pirie et al. (2024) and analysed separately to assess supported phylogenetic conflict before (in the absence of such conflict) being combined in a supermatrix of the complete nrDNA and plastid sequence data. For *Erica* sp. RDH184, the one ITS sequence corresponding to *E.peltata* was combined with the plastid data whilst the other was included as a separate operational taxonomic unit in the combined analyses, as in the approach of Pirie et al. (2009).

We performed phylogenetic analyses using IQ-TREE v. 2.3.4, with automatic partition merging and model selection, followed by 3,000 iterations of tree searching (Chernomor et al. 2016; Kalyaanamoorthy et al. 2017; Minh et al. 2020). We evaluated branch support using 10,000 replicates of Ultrafast Bootstrap (Hoang et al. 2018) and SH-alrt (Guindon et al. 2010).

### ﻿Morphological comparisons with herbarium records

Material in the BOL and NBG herbaria (Thiers 2018) was examined, both of morphologically similar and closely related taxa, as identified above, to find additional collections of each putative novel taxon. Potentially diagnostic characters for putative new taxa were compared to the morphologically similar and closely related species.

## ﻿Results

### ﻿Field research and collection

We made collections of 10 specimens representing five unidentified and/or potentially undescribed species and five additional known taxa not previously sequenced. Observations were recorded on the open platform iNaturalist (https://www.inaturalist.org). Collection and observation data, along with GenBank accession numbers, were uploaded to GBIF (Suppl. material 1; https://doi.org/10.15468/abecqd).

### ﻿Morphological comparisons using the *Erica* ID aid

We found little overlap between taxon lists generated from the strict matching versus highest-scoring taxa probabilistic analyses (Tables 1, 2; Suppl. material 2). In some cases, there was no strict match at all; in others, taxa that are consistent with the coding of the putative novelties but more variable were recovered with lower probability. Adding distribution and phenological data to the analysis dramatically reduced options for similar taxa.

**Table 1.** Coded characters for putative new species used in character-based searches on the *Erica* ID aid. Characters which are substantially variable in some cases, e.g. leaf hairiness and style exsertion, and therefore less informative for strict matching, are omitted here; however, all characters are documented in the updated version of the *Erica* ID aid.

Character	*Erica* sp. RDH181	*Erica* sp. RDH183	*Erica* sp. RDH184	*Erica* sp. RDH218	*Erica* sp. Vlok2988
Corolla Size (mm)	1–5	1–5 OR 5–10	1–5	1–5	1–5
Corolla Shape	Bell OR Cup OR Urn	Bell OR Cup	Bell OR Cup	Bell OR Cup	Urn OR Cup
Corolla Colour	White	White	Pink OR Red	White	Green OR Yellow OR Red
Sepal/Corolla Length Ratio	< ½ OR >½	< ½	< ½ OR > ½	< ½	< ½
Hairy structures	Stem AND Leaf AND Pedicel AND Sepal AND Ovary	Leaf AND Pedicel AND Sepal AND Corolla AND Ovary	Stem AND Leaf AND Pedicel AND Sepal AND Ovary	Ovary	Stem
Non-Hairy structures	Corolla	-	Corolla	Stem AND Pedicel AND Corolla	Leaf AND Pedicel AND Sepal AND Corolla AND Ovary
Style	Exserted	-	Exserted	Exserted	Exserted
Anthers	-	Non exserted	Non exserted	Non exserted	-
Anther appendages	Present	Present	Absent	Absent	Absent
Stamens (no.)	8	8	8	8	6
Sepals (no.)	4	4	4	4	4
Corolla lobes (no.)	4	4	4	4	4
Leaves (n-nate)	3	3	3	3	3
Bracts and bracteoles (no.)	3	3	3	3	3
Fire survival	Reseeder	Reseeder	Reseeder	Reseeder	Reseeder
Flowering month	December OR January	January OR February	January OR February	December OR January OR February	April OR May OR June
Region	Langeberg	Langeberg	Agulhas	Langeberg	Karoo Mountains

**Table 2.** Findings from character-based searches for putative new species using the *Erica* ID aid “Match” and “Probability” algorithms. Results for the “Probability” algorithm are presented in descending order of probability. For illustrative purposes, the first three taxon outputs for probabilistic identification are presented; full outputs are presented in Suppl. material 2.

	*Erica* sp. RDH181	*Erica* sp. RDH183	*Erica* sp. RDH184	*Erica* sp. RDH218	*Erica* sp. Vlok2988
Match function					
Region and flowering month excluded (no.)	*E.hispidula*, *E.magistrati*, *E.oakesiorum*, *E.rivularis*, *E.tegetiformis*, *E.umbratica*, *E.woodii* (7)	*E.cryptanthera*, *E.cymosa*, E.cymosasubsp.grandiflora, *E.galantha*, *E.myriocodon*, *E.oxyandra*, *E.setosa*, *E.woodii* (7)	*E.adnata*, *E.blancheana*, *E.comata*, *E.cordata*, *E.elimensis*, *E.granulatifolia*, E.langebergensis, *E.lavandulifolia*, *E.macrotrema*, *E.melanthera*, *E.mundii*, *E.natalitia*, *E.newdigateae*, *E.pearsoniana*, *E.peltata*, *E.pilaarkopensis*, *E.rhodella*, *E.sparsa*, *E.tenuipes* (19)	*E.tenuicaulis* (1)	(0)
Region included and flowering month excluded (no.)	*E.hispidula* (1)	*E.oxyandra* (1)	*E.elimensis* (1)	*E.tenuicaulis* (1)	(0)
All characters included (no.)	*E.hispidula* (1)	*E.oxyandra* (1)	(0)	*E.tenuicaulis* (1)	(0)
Probability function					
Region and flowering month excluded	*E.woodii*, *E.galantha*, *E.setosa*	*E.viscaria*, *E.varderi*, *E.distorta*	*E.cymosa*, *E.diotiflora*, *E.zitzikammensis*	*E.tenuicaulis*, *E.argentea*, *E.oliveranthus*	*E.binaria*, *E.atricha*, *E.heterophylla*
Region included and flowering month excluded	*E.heterophylla*, *E.woodii*, *E.galantha*	*E.albescens*, *E.oxyandra*, *E.cristiflora*	*E.axilliflora*, *E.nudiflora*, *E.aghillana*	*E.tenuicaulis*, *E.oliveranthus*, *E.argentea*	*E.binaria*, *E.atricha*, *E.heterophylla*
All characters included	*E.heterophylla*, *E.podophylla*, *E.woodii*	*E.albescens*, *E.oxyandra*, *E.viscaria*	*E.axilliflora*, *E.canescens*, *E.nudiflora*	*E.tenuicaulis*, *E.oliveranthus*, *E.heterophylla*	*E.binaria*, *E.heterophylla*, *E.atricha*

### ﻿Identifying closely related taxa using phylogenetic inference

**Figure 1.**
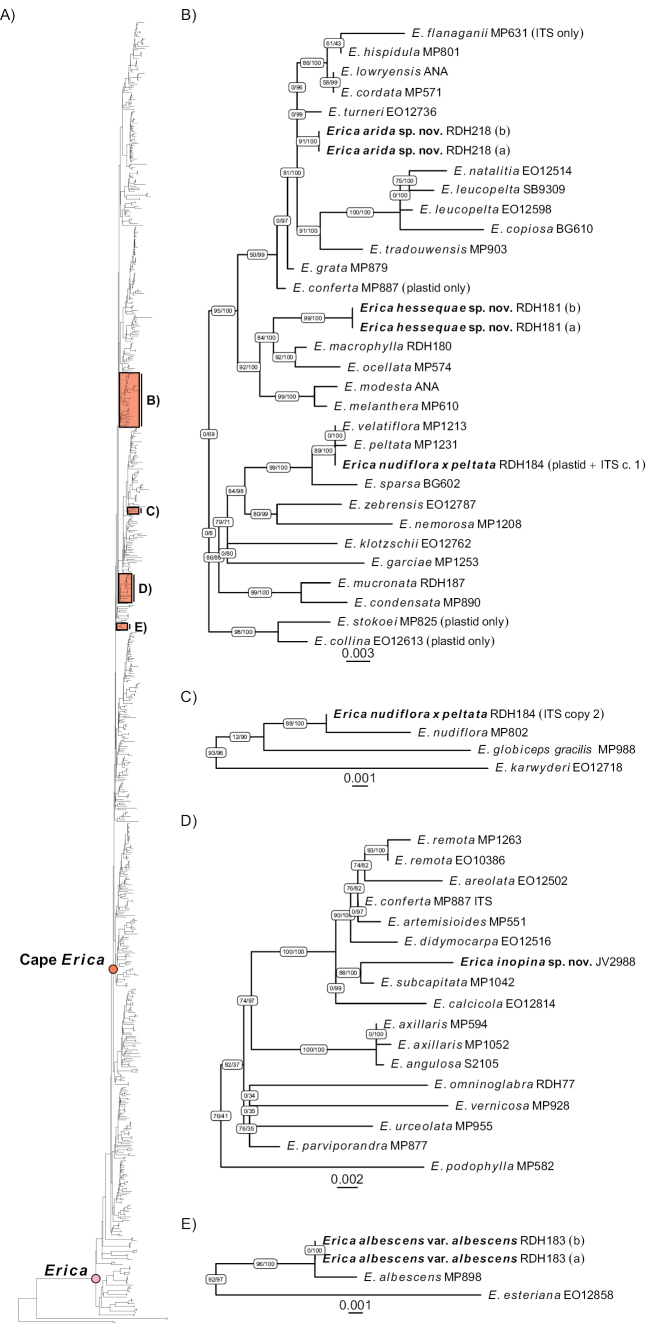
Selected clades from the phylogenetic analysis of combined plastid and nuclear sequence data illustrating the relatedness of the focal specimens analysed here. The complete phylogeny is shown in **A** and the positions of the focal clades (**B–D**) are highlighted.

In most cases, phylogenetic analyses of the separate ITS and trnF-ndhJ sequence alignments yielded consistent results (except for the two ITS copies in *Erica* sp. RDH184) and thus the data from both partitions were combined into a single analysis (Fig. 1; Suppl. material 3). The following are the closest related taxa for the focal putative novel taxa based on ITS and plastid data:

For *Erica* sp. RDH184, the plastid phylogeny placed this sample in a well-supported clade (ultrafast bootstrap support [BS] = 100%; SH-alrt support [AS] = 90%) along with *E.peltata*, *E.sparsa* G.Sinclair, and *E.velatiflora* E.G.H.Oliver (Suppl. material 3: fig. S1). The two copies of ITS “phased” manually from the sequence chromatograms showed contrasting relationships in the nuclear phylogeny (Suppl. material 3: fig. S2). As expected, the copy that was phased based on its close match to *E.peltata* (Copy 1) was placed with good support (BS = 100%, AS = 97%) in a clade with *E.peltata* and *E.velatiflora*, while the other copy (Copy 2) was well supported (BS = 100% AS = 83%) as sister to *E.nudiflora* L.. Based on the concordance between the plastid and nuclear results, we concatenated Copy 1 with the plastid data for the combined nuclear and plastid analysis, while retaining Copy 2 separately. The combined analysis showed a pattern consistent with the plastid and nuclear phylogenies, with the “primary” RDH184 sequence grouping with *E.peltata* and *E.velatiflora* (BS = 100%, AS = 89%; Fig. 1B) and the “secondary” (alternative ITS-only) copy grouping with *E.nudiflora* (BS = 100%, AS = 88%; Fig. 1C).

For *Erica* sp. RDH181, the nuclear phylogeny suggested a well-supported (BS = 100%, AS = 91%) sister relationship with *E.ocellata* Guthrie & Bolus and *E.macrophylla* Klotzsch ex Benth. (Suppl. material 3: fig. S2). In contrast, the plastid phylogeny supported a sister relationship with *E.grata* Guthrie & Bolus (BS = 100%, AS = 87%), although the placement of this pair was poorly resolved (BS = 51%, AS = 0%) within a broader well-supported (BS = 99%, AS = 88%) clade including, among several others, *E.macrophylla* and *E.ocellata* (Suppl. material 3: fig. S1). The combined analysis agreed with the nuclear phylogeny, placing RDH181 in a clade with *E.macrophylla* and *E.ocellata* (BS = 100%, AS = 84%; Fig. 1B).

In all three analyses, *Erica* sp. RDH218 was included in a clade with, among others, *E.grata*, *E.tradouwensis* Compton, *E.turneri* E.G.H.Oliv., *E.cordata* Andrews, and *E.hispidula* L., although this grouping received variable support depending on the analysis (nuclear: BS = 100%, AS = 90%; plastid: BS = 51%, AS = 0%; combined: BS = 97%, AS = 0%; Fig. 1B; Suppl. material 3: figs S1, S2).

The placement of *Erica* sp. JV2988 was not resolved by the plastid data (Suppl. material 3: fig. S1), but the nuclear and combined analyses indicated a well-supported (nuclear: BS = 100%, AS = 89%; combined: BS = 100%, AS = 88%) close relationship to *E.subcapitata* (N.E.Br.) E.G.H.Oliv., the two forming a sister pair nested in a clade including *E.calcicola* (E.G.H.Oliv.) E.G.H.Oliv., *E.areolata* (N.E.Br.) E.G.H.Oliv., *E.artemisioides* (Klotzsch) E.G.H.Oliv., *E.conferta* Andrews, *E.didymocarpa* E.C.Nelson & E.G.H.Oliv., and *E.remota* (N.E.Br.) E.G.H.Oliv., which had strong support (nuclear: BS = 100%, AS = 97%; combined: BS = 100%, AS = 100%; Fig. 1D; Suppl. material 3: fig. S2).

*Erica* sp. RDH183 was placed as sister to *E.albescens* Klotzsch ex Benth. (nuclear: BS = 100%, AS = 87%; plastid: BS = 100%, AS = 93%; combined: BS = 100%, AS = 96%; Suppl. material 3: figs S1, S2, S3).

## ﻿Discussion

In this work we focused on five apparently distinct Cape ericas potentially representing new species. In three cases we were able to compare the specimens to both the most similar species and to their closest relatives (insofar known) and show that they can be clearly distinguished and warrant describing as new (see taxonomic treatment below). In the two other cases, the approach allowed us to better explain the variation observed and we were able to rule out the necessity for describing new taxa.

**Figure 2.**
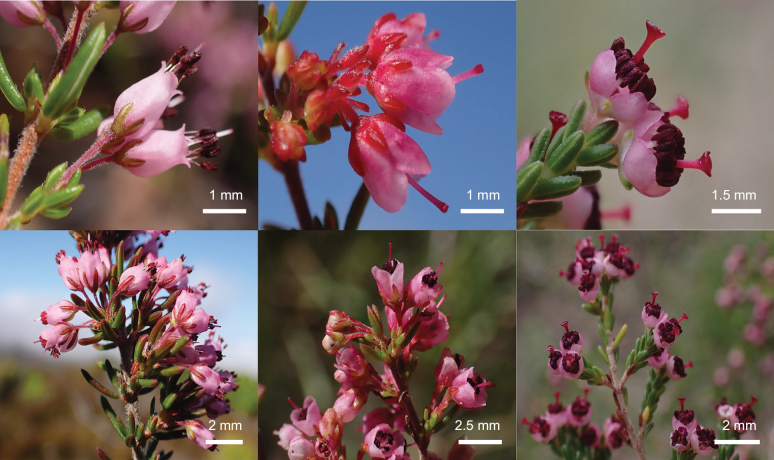
Morphological comparison between *Ericanudiflora* (top and bottom left), *E.* sp. RDH184 (top and bottom middle) and *E.peltata* (top and bottom right).

In the case of *Erica* sp. RDH184, the molecular evidence indicated that a morphologically distinct individual represents a (naturally occurring) hybrid. The data represents two independent gene trees: that of the generally maternally inherited plastid and biparentally inherited nuclear ribosomal genes. The latter can be problematic for phylogenetic inference because they are present in numerous copies in the genome, including potentially paralogous copies (Baldwin et al. 1995). However, in *Erica* this rarely appears to present a problem (several exceptions are listed in Pirie et al. 2024), and in this case it was possible to discern two copies with apparently comparable copy numbers but distinct phylogenetic signals: one consistent with the plastid data, indicating a maternal inheritance from *E.peltata* (or potentially an unsampled close relative), the other more similar to *E.nudiflora*. During character-based searches with the *Erica* ID aid, *E.peltata* was included in the results for the “Match” algorithm and *E.nudiflora* appeared in all three searches with the “Probability” algorithm where region was included as a character. Both putative parent species are known from the area and there are clear morphological similarities (see Fig. 2, Table 3 for comparisons). Additionally, only one individual like *Erica* sp. RDH184 has been found despite exhaustive surveys in the area. The data therefore provide strong evidence that it is an F_1_ hybrid. This result contributes to our knowledge of reproductive barriers amongst *Erica* species and highlights how an integrative approach could avoid artificially inflating the numbers of true species with morphologically distinct hybrids.

**Table 3.** Comparison of key morphological features between *Ericanudiflora*, *E.* sp. RDH184 and *E.peltata*.

	*Ericanudiflora*	*E.* sp. RDH184	*E.peltata*
Branches	pubescent	glabrous	pubescent
Leaves	3-nate	3-nate	3-nate
	4–10 mm long	2.5–5.0 mm long	2.0–3.5 mm long
	lanceolate, acute	lanceolate, acute	lanceolate, acute
	glabrous to hispidulous	mostly glabrous	mostly glabrous
Flowers	pseudo-axillary on reduced side branches	terminal on short side branches	terminal at the ends of branchlets
Pedicels	2.5–5.5 mm long	2.0–3.0 mm long	± 1 mm long
	puberulous	pubescent	puberulous
Bracts and bracteoles	3	3	0–3
	remote	subapproximate	variable, bracteoles usually subremote
		Ciliolate	
Calyx	1:4 to 1:2 to corolla	1:2 to corolla	1:2–1:1 to corolla
	lobes lanceolate, acute	lobes lanceolate, acute	lobes ovate, subacute to obtuse
	mostly glabrous,		
Corolla	subcampanulate-	cyathiform	broad cyathiform
	cyathiform	pink to red	pink
	pink to red	glabrous	glabrous
	glabrous	2.0–2.5 mm long	1.5–2.5 mm long
	2.5–5 mm long		
Anthers	exserted	included	manifest to exserted
	scabrous	scabrous	scabrid
	muticous	muticous	muticous
	pores ¼–1/3 of anther	pores ½ of anther	pores 2/3 of anther
Ovary	glabrous	pubescent	pubescent
Style	exserted	exserted	exserted
	simple	simple	peltate
Nectaries	present	present	absent

For *Erica* sp. RDH183, the results pointed to a match with an existing species whilst identifying a need to update the character coding within the *Erica* ID aid. Phylogenetic analysis of this specimen showed a close relationship to a specimen (MP898) collected in the nearby area of Boosmansbos identified by E.G.H. Oliver as *E.albescens*. Morphologically, both specimens appear to correspond to E.albescensvaralbescens whilst deviating in flower shape coded for the species in the *Erica* ID aid, thereby excluding a strict match. However, using the “Probability” algorithm, *E.albescens* was recovered as the most likely species once the region was included as a character (see Table 2). The simplest conclusion from these results is to determine the specimen as E.albescensvaralbescens and to update the *Erica* ID aid accordingly.

In general, the results highlighted the importance for identification and species matching of factors such as geographic distribution and flowering time. These are clearly relevant for species boundaries in the context of the patterns of narrow endemism and shifts between pollinators within the Cape (Van der Niet et al. 2014; Musker et al. 2024). The differences between results based on strict and probabilistic matching are consistent with the different interpretations of character variation they imply. Otherwise similar taxa might be unambiguously distinguished with strict matching. As an example, *Ericapodophylla* is the second-most probable match for *Erica* sp. RDH181, yet it does not feature in the strict matching due to its hairy corolla (non-hairy in *Erica* sp. RDH181). For *Erica* sp. JV2988 there is no strict match at all, and probabilistic matching suggests comparison to *E.atricha* with the more common 8 stamens (as opposed to 6 stamens as in *Erica* sp. JV2988). The possibilities for unrecorded intraspecific variation and for simple error clearly support the use of both algorithms when attempting species identifications.

The new samples of known taxa and of newly discovered species bring the total representation of species diversity in the phylogenetic tree of *Erica* to around 66% of 854 species, as compared to 65% of 851 species in Pirie et al. (2024). Further increases in sampling will improve the power of phylogenetic comparisons in discovering and assessing putative species diversity in *Erica*. Testing results will provide further improvement, particularly when they are derived from single samples of specific taxa or if they seem unexpected from the morphologies and distributions of apparently closely related taxa (such as that of the ITS sequence of *E.flanaganii* Bolus MP631). Phylogenetic analysis of *Erica* taxa is mostly based on only a few Sanger-sequenced markers. As in the cases illustrated here, these markers can still be informative despite their limited variability and representation of genomes; however, they will not always deliver a conclusive result. High-throughput sequencing and phylogenomic approaches (Musker et al. 2024, 2025) may be needed to inform taxonomic decisions, particularly in species complexes.

Subgeneric classification is another area in which further research is needed. The classification used by Guthrie and Bolus (1905) to group similar species into sections has been maintained in subsequent work and adopted on iNaturalist. However, the sections are not monophyletic, often grouping distantly related taxa (Pirie et al. 2011, 2016). They cannot be assumed to have predictive value for combinations of traits in either known or undescribed taxa. This has made the attempt to place new species within the existing classification challenging and, arguably, arbitrary. Thus, the subgeneric placement of new species and the current classification should not be over-interpreted. Nonetheless, our hope is that the attempt will be useful as a bridge towards a more robust classification of *Erica*.

### ﻿Taxonomic treatment

**Figure 3.**
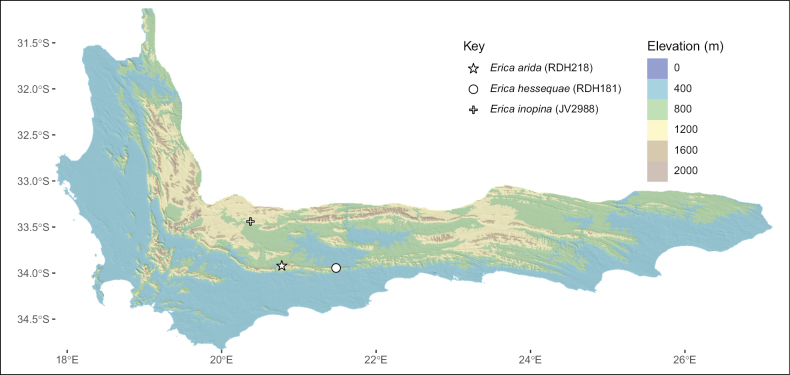
Map of the Cape Floristic Region, showing the distributions of the new taxa described in this study.

This section provides a taxonomic treatment for three new *Erica* taxa from the Western Cape, South Africa. As far as possible, taxonomic treatments were standardised in accordance with the methodology of E.G.H. Oliver, with minor morphological details maintained as subtle but key distinguishing characters in this large and complex genus. The treatment for *E.inopina* is reduced to follow Oliver’s (2000) methodology for species with indehiscent and partially dehiscent fruit, which includes less detail. Diagnoses are included above the descriptions as in the recommendations of Hassemer et al. (2020). We distinguish the new taxa from those that present a strict match according to the *Erica* ID aid without reiterating characters in Table 1; in one case without a strict match we report the characters that differ from the closest known relative. Further discussion of similar taxa, including close relatives, identification, and more variable characters (such as habit) are included under subsequent notes. Specimen localities are based on the quarter-degree grid cell system employed by the South African National Mapping Agency, National Geo-Spatial Information (2017), e.g. “3321CD”, in addition to precise coordinates. We also provide summary descriptions (Suppl. material 4) and geographic distributions (Fig. 3).

#### ﻿ Erica arida

Taxon classificationPlantaeEricalesEricaceae

R.D.Hoekstra sp. nov.

9651A820-52D4-5567-98C1-372F5A75DC57

urn:lsid:ipni.org:names:77362792-1

Fig. 4

##### Link.

WFO: https://list.worldfloraonline.org/wfo-1000079209.

##### Type.

South Africa • Western Cape, 3320DD (Warmwaterberg): Langeberg Range, Barrydale outskirts, on steep middle south-facing slopes north of the Doringrivier Catchment Area, 709 m, 33.922°S, 20.779°E, 14 February 2024, *R.D. Hoekstra 218* (NBG, holotype).

##### Diagnosis.

*Ericaarida* matches characters exhibited in *E.tenuicaulis*, from which it differs in leaf shape (saddle-shaped, as opposed to broadly linear in *E.tenuicaulis*), its straight style (curved in *E.tenuicaulis*) and staminal filaments approximately 3 times as long as the anthers (as long as the anthers in *E.tenuicaulis*).

##### Description.

Rounded to semi-spreading, resprouting shrublet up to 30 cm tall, rootstock thick. ***Branches*** twiggy, erect, glabrous; secondary branches erect or ascending, sparsely stipitate-glandular. ***Leaves*** 3-nate, erect to spreading, ovate to subcordate, saddle-shaped, blades 2.5–4.0 × 1.0–1.5 mm, acute; adaxially convex, glabrous, margins revolute, rarely sparsely ciliolate; abaxially deeply sulcate, densely hispidulous within sulcus with simple, eglandular hairs; petiole ± 1 mm long, occasionally loosely decurrent, margins ciliolate. ***Inflorescence*** of 3-nate flowers at ends of short side branches; pedicel 3.0–4.0 mm long, pale green to pink, viscid, sessile- and stipitate-glandular; bracteoles 2, opposite, median to submedian, oblong to shortly lanceolate, ± 0.75 mm long, subacute, sessile-glandular, glabrous but margins sparsely ciliolate at base, green and slightly sulcate towards abaxial apex; bract partially recaulescent, submedian, lanceolate, acute, sessile-glandular, margin sparsely ciliolate towards base. ***Calyx*** 4-lobed, sepals connate at base, lanceolate, 1.25–1.5 mm long, acute; abaxially glabrous, apex sulcate, the sulcus shortly villous; adaxially glabrous; margins sessile-glandular. ***Corolla*** campanulate to shortly urceolate with slightly constricted throat, 3.0–4.0 × 3.0–4.0 mm, white, glabrous, dry; lobes erect to spreading, ± 0.5 mm long, acute, minutely serrated. ***Stamens*** 8, free, included, filaments flat, ± 2.5 mm long, glabrous, white turning golden reddish distally; anthers ± 0.8 mm long, obcuneate, dorsifixed at base, bipartite, thecae erect, free, muticous, dark brown; pore ± 0.3 mm long, oval. ***Ovary*** 4-locular, shortly oblong, ± 0.8 mm long, slightly emarginate, red to purplish, hispid with long, simple, eglandular hairs; ovules ± 20 per locule; placenta apical; nectaries basal, green; style ± 3.5 mm long, glabrous, pale pink turning dark red or black towards stigma, exserted; stigma dark red to black, subcapitate. ***Fruit*** and ***seeds*** not seen. ***Flowering time***: December to February.

**Figure 4.**
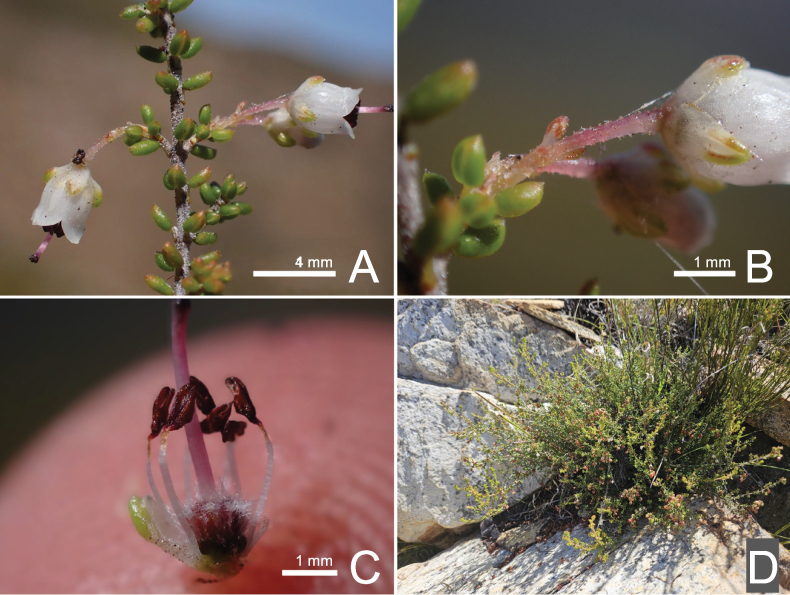
*Ericaarida* R.D.Hoekstra **A** incomplete inflorescences **B** glandular pedicel, bracteoles and calyx **C** dissected flower showing parts of the androecium and gynoecium **D** whole plant in situ. Voucher *R.D. Hoekstra 218* (NBG).

##### Distribution and habitat.

*Ericaarida* is known only from the mountains north of the Doringrivier Catchment Area east of Barrydale in the Western Cape. It has been recorded from a ridgeline at an altitude of 1190 m in Northern Langeberg Sandstone Fynbos (Mucina and Rutherford 2006), growing in association with *Ericabarrydalensis*, as well as on the middle south-facing slopes as low as 516 m growing in association with *Phylicamairei*, *Ericavestita* and *Cliffortiapulchella*. It has not been recorded from the north-facing slopes.

##### Threat status.

*Ericaarida* is only known from a few records on iNaturalist and our single collection from the Doringrivier Catchment Area. The three known subpopulations appear to be small and scattered within the catchment area, and our survey at the type locality revealed only 17 plants. *Ericaarida* was found to have an AOO of 12.00 km^2^ and an EOO of 12.00 km^2.^ Additionally, the marshland in the catchment area is heavily infested with invasive *Hakeasericea* with a significant encroachment up to the middle south-facing slopes where *E.arida* occurs. This poses a major threat to the two subpopulations on the middle slopes. The habitat of *E.arida* is remote and difficult to access and only limited botanical surveys have been performed around the catchment area. Therefore, there are not enough data to accurately estimate the population size of this species. However, the wide range in altitude between the known subpopulations and results from our survey suggest that additional small subpopulations may exist within this catchment area. Since all observed subpopulations have consisted of only a few plants we estimate a minimum total population of 100 plants. Based on this estimate, an EOO of 12.00 km^2^, ongoing decline in quality of habitat as a result of invasive alien plant species, and its restriction to a single known location, we recommend an IUCN (2012) category of Critically Endangered (CR) under criterion B1ab(iii) for *E.arida*.

##### Pollination syndrome.

*Ericaarida* has well-developed nectaries and small, open cup-shaped flowers, suggesting entomophily. The lack of a peltate stigma and the fact that pollen is not released in a cloud after in situ manipulation make anemophily highly unlikely, and the size and shape of the corolla make ornithophily unlikely.

##### Etymology.

*Ericaarida* is named for the seasonal aridity seen during its flowering months in the part of the Klein Karoo where it occurs; from the Latin, *arida*, for dry or arid.

##### Subgeneric classification.

The closest relatives of *E.arida* are classified in at least five different (non-monophyletic) sections, but most are in either *Ceramia* (glabrous, pitcher-shaped corollas and terminal inflorescences: consistent with *E.arida*) or *Arsace* (large peltate stigmas: inconsistent). Placement of the new species in *Ceramia* may be a useful provisional classification.

##### Notes on morphology and phylogeny.

The single strict match for *E.arida* with the *Erica* ID aid, *E.tenuicaulis*, diagnosed above, has yet to be analysed phylogenetically. Phylogenetic data place *E.arida* in a clade containing *E.cordata*, *E.lowryensis*, *E.flanaganii* (ITS only), *E.hispidula*, *E.turneri*, *E.leucopelta*, *E.natalitia*, *E.copiosa*, *E.tradouwensis* and *E.grata*, with which it is unlikely to be confused as it differs from all of these by its glabrous (as opposed to hairy) leaves. It further differs from *E.cordata*, *E.lowryensis* and *E.tenuicaulis* in leaf shape (saddle-shaped, as opposed to cordate in *E.cordata*, and lanceolate in *E.lowryensis*); from *E.turneri* and *E.copiosa* by its muticous (as opposed to awned) anthers; from *E.turneri* by its closed-backed (versus distinctly broad, open-backed) leaves, and glabrous (versus hairy) corolla, and from *E.copiosa*, *E.tradouwensis* and *E.grata* by its glabrous (as opposed to hairy) sepals. *Ericahispidula*, *E.leucopelta* and *E.natalitia* are clearly distinct wind-pollinated plants with peltate stigmas and absent nectaries, in contrast to *E.arida* which has a subcapitate stigma and conspicuous nectaries.

The placement of *Ericaflanaganii* is unexpected: it is geographically distant, from the Drakensberg Mountains more than 500 km from the eastern edge of the CFR, as well as morphologically dissimilar, possessing a hairy corolla, lanceolate imbricate leaves, a fully recaulescent bract and anther appendages. The result (from data presented by Pirie et al. 2011; 2024) is only shown by ITS and contradicted by plastid results. Additional data from different samples are needed.

#### ﻿ Erica hessequae

Taxon classificationPlantaeEricalesEricaceae

R.D.Hoekstra sp. nov.

345EC13E-C5A9-57F3-B393-93AA265D260F

urn:lsid:ipni.org:names:77362793-1

Fig. 5

##### Link.

WFO: https://list.worldfloraonline.org/wfo-1000079210.

##### Type.

South Africa • Western Cape, 3321CD (Sandkraal): Langeberg Range, Romanskraal north of Albertinia, north-facing slopes west of Skoorsteen Peak, 1165 m, 33.945°S, 21.482°E, 23 December 2023, *R.D. Hoekstra 181* (NBG, holotype; BG, isotype [2088580]).

##### Diagnosis.

*Ericahessequae* matches characteristics exhibited in *E.hispidula*, *E.oakesiorum*, *E.rivularis*, *E.tegetiformis*, *E.umbratica and E.woodii.* It can be distinguished from all these taxa by its ovate leaves (linear, linear-lanceolate or lanceolate in all). It is further distinguished by its glandular pedicel, sepals, bract and bracteoles (all eglandular in *E.magistrati*, *E.oakesiorum* and *E.tegetiformis*); by its subcapitate stigma (peltate in *E.hispidula*; simple in *E.rivularis* and *E.tegetiformis*), and by its densely lanate ovary (shortly hispid in *E.hispidula*, *E.oakesiorum*, *E.rivularis*, *E.woodii* and *E.umbratica*).

##### Description.

Semi-spreading shrublet up to 80 cm tall. ***Branches*** twiggy, glabrous to puberulous; secondary branches ascending, hispid with a mix of simple, eglandular hairs and stipitate glands. ***Leaves*** 3-nate, erect, ovate, blades 4.0–5.0 × 1.5–2.5 mm, acute, open-backed; adaxially slightly convex, hispidulous when young with simple, eglandular hairs mixed with stipitate and sessile glands, glabrescent, margins thickened, revolute; abaxially deeply sulcate, pale, densely hispidulous with simple, eglandular hairs; petiole 0.8–1.2 mm long, hispidulous and glandular as for adaxial leaf surface, not decurrent. ***Inflorescence*** of 3-nate flowers, terminal on secondary and side branches; pedicel 4.0–5.5 mm long, pale creamy green turning red-purplish with exposure to sun, stipitate-glandular, viscid; bracteoles 2, median, lanceolate, ± 1.5 × 0.5 mm, acute, creamy white to dark pink, leathery, margins densely sessile-glandular, abaxially sparsely hispid towards apex and along basal margin, adaxially glabrous; bract partially recaulescent, sub-median, ovate to lanceolate, ± 0.5 × 1.5 mm long, acute, creamy white to dark pink, leathery, margins densely sessile-glandular, abaxially sparsely hispid towards apex and along basal margin, adaxially glabrous. ***Calyx*** 4-lobed; sepals adpressed to corolla, ovate, 2.0–2.5 × 1.0–1.5 mm, creamy white/yellow with green tips, leathery; margins densely sessile-glandular, revolute towards apex; abaxially sulcate, with occasional stalked glands and sparsely hispidulous with simple, eglandular hairs within sulcus and at base, otherwise glabrous, viscid; adaxially glabrous, midrib slightly raised. ***Corolla*** 4-lobed, cyathiform to shortly urceolate, throat constricted, 3.5–4.5 × 3.0–4.0 mm, creamy white, glabrous, viscid, occasionally with stalked glands adjacent to sepals; lobes recurved, 0.75–1.25 mm long, obtuse, margins smooth. ***Stamens*** 8, free, manifest to exserted, filaments flat, 2.5–3.0 mm long, glabrous, white turning dark reddish pink towards apex, apically kyphotic; anthers cuneate, 0.7–0.8 mm long, brown, dorsifixed at base, bipartite, thecae erect, ventral surface golden brown and shortly scabrous; awns ± 0.4 mm long, thin, laterally fixed to apex of filaments, simple or rarely with one or two basal barbs, reddish brown; pores round, ± 0.4 mm long. ***Ovary*** 4-locular, turbinate, ± 1.0 mm long, dark purple, densely lanate with simple, white, eglandular hairs; ovules 15–20 per locule; placenta apical; nectaries basal, green to black; style ± 3.5 mm long, glabrous, pale pink above but white towards base, exserted; stigma dark pink to purplish, subcapitate. ***Fruit*** and ***seeds*** not seen. ***Flowering time***: December to January.

**Figure 5.**
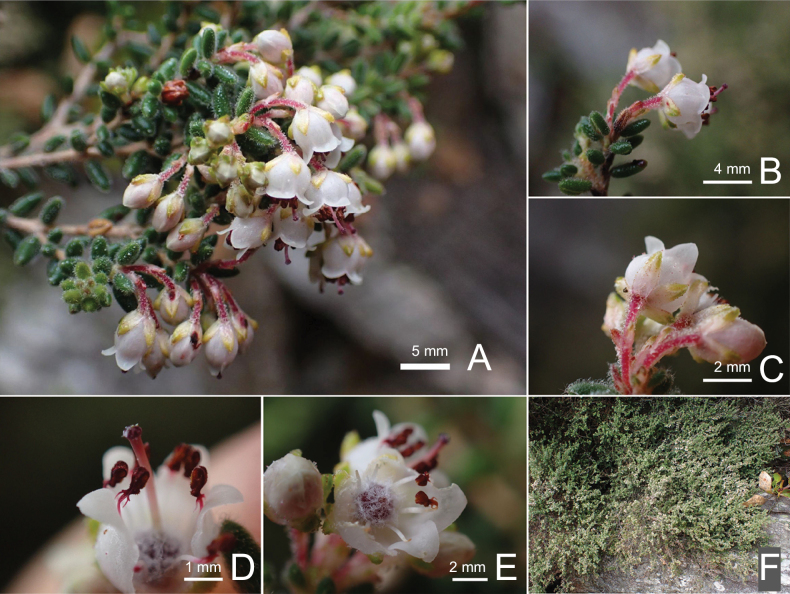
*Ericahessequae* R.D.Hoekstra **A** multiple inflorescences **B** single inflorescence **C** calyx and pedicel **D** dissected flower showing androecium **E** dissected flower showing ovary **F** whole plant in situ. Voucher *R.D. Hoekstra 181* (NBG).

##### Distribution and habitat.

*Ericahessequae* is only known from two localities, some 4 km apart, on the mountains surrounding Romanskraal north of Albertinia—one along the ridgeline leading up to a plateau mountain west of Skoorsteen Peak, the other on Langeberg Peak. It appears to be confined to high-elevation ridgelines where it grows amongst craggy rocks in North and South Langeberg Sandstone Fynbos (Mucina and Rutherford 2006). It has only been observed at altitudes above 1100 m.

##### Threat status.

*Ericahessequae* appears to be a naturally rare, range-restricted species. There are only two records of the species, one plant having been observed on Langeberg Peak and two west of Skoorsteen Peak. Limited surveys performed in the surrounding area have failed to reveal additional localities. While alien vegetation is present in the region, favouring middle, south-facing slopes, neither location is yet threatened by invasive species. The plants examined at both localities demonstrated a reseeding regeneration strategy, which may represent a vulnerability to too-frequent fires. *Ericahessequae* was found to have an AOO of 8.00 km^2^ and an EOO of 8.00 km^2^. Unfortunately, its habitat is remote and not easily accessible, making estimation of the population size difficult in the absence of more systematic surveys. Until more data can be collected, current conservative estimates put the total population size at fewer than 50 mature individuals. Based on this estimate, an IUCN (2012) category Critically Endangered (CR) under criterion D is recommended.

##### Pollination syndrome.

The small, open cup-shaped flowers of *Ericahessequae* have well-developed nectaries and anther appendages, suggesting entomophily. The corolla is remarkably viscid and thus likely to trap crawling and flying insects that would need to land on the surface to effectuate pollination. Therefore, small and medium-sized flying insects that avoid trapping by landing on the recurved adaxial lobes or by entering the corolla through the mouth are the most likely pollinators of this species, although no clear interaction with pollinators has been observed. The lack of a peltate stigma and the fact that pollen is not released in a cloud after manipulation make anemophily highly unlikely.

##### Etymology.

*Ericahessequae* is named for the Hessequa clan which occupied most of the modern-day Hessequa municipal area encompassing the Eastern Langeberg Range between the Tradouw Pass and Gourits River.

##### Subgeneric classification.

*Ericahessequae* could be placed in section Ceramia (Guthrie & Bolus, 1905) by its glabrous, pitcher-shaped corolla and terminal inflorescence.

##### Notes on morphology and phylogeny.

Phylogenetic data suggest that *E.macrophylla* and *E.ocellata* (sect. Ceramia) are closely related to *E.hessequae*. While there are morphological similarities among these three Langeberg-endemic taxa, the former two are readily distinguishable by their muticous anthers. In addition, *E.macrophylla* has a more elongate, urceolate or tubular corolla with a more constricted throat as compared to the stouter bell- to cup-shaped corolla of *E.hessequae*. *Ericaocellata* is distinct in having a capitate, 6- to 10-flowered inflorescence (terminal and 3-nate in *E.hessequae*) with significantly shorter pedicels and sepals as compared to *E.hessequae*.

Beyond character matching and phylogenetic relatedness, *Ericahessequae* bears an overall morphological similarity to *E.grata* and *E.cordata*, two other species found in the Langeberg. *Ericahessequae* and *E.grata* are restricted to the Langeberg Range whilst *E.cordata* has a wider distribution. *Ericahessequae* can be distinguished from *E.grata* by its viscid (as opposed to dry), creamy white (as opposed to light- to red-pink) corolla with a less pronounced constriction at the throat, and by its more densely hairy, lanate (as opposed to hispid) ovary; and from *E.cordata* by its awned (as opposed to muticous) anthers and subcapitate (as opposed to capitate to peltate) stigma. The leaves in *E.grata* are usually narrower and more lanceolate with less markedly revolute margins than those of *E.hessequae*, and it is usually a laxer, upright shrublet preferring wet, south-facing lower to middle slopes. *Ericacordata* is also mostly upright in habit with softer, shorter side branches. In contrast, *Ericahessequae* has hard ligneous stems and main branches, a semi-spreading, rounded habit, and preference for high-altitude craggy peaks.

##### Additional specimens examined.

Western Cape, 3321CD (Sandkraal): Langeberg Mtns, summit of Unnamed Peak (Trig Beacon 45) east of the Langkloof, 1506 m, 33°57.037'S, 21°26.257'E, 22 February 2003, *R.C. Turner 701* (NBG).

#### ﻿ Erica inopina

Taxon classificationPlantaeEricalesEricaceae

J.H.J.Vlok sp. nov.

DCE98308-BB4A-53A6-B260-3C8C2234A4D9

urn:lsid:ipni.org:names:77362794-1

Fig. 6

##### Link.

WFO: https://list.worldfloraonline.org/wfo-1000079208.

##### Type.

South Africa • Western Cape, 3320BB (Laingsburg): Laingsburg district, about 20 km south-east of Touws River, on Lettaskraal Farm, Brandhoek section, 840 m, -33.440°S, 20.373°E, 13 April 2021, *J.H.J. Vlok 2988* (NBG, holotype; BG, isotype [2088576]).

##### Diagnosis.

*Ericainopina* is closely related and morphologically similar to *E.subcapitata*. It can be distinguished by its glabrous (as opposed to hairy) sepals, the presence of 3 (as opposed to 0) bracts on the pedicel, and its smooth (as opposed to verrucose) 3-locular (as opposed to 1- or 2- locular) ovary.

##### Description.

Erect, densely branched, reseeding shrub to 50 cm tall. ***Branches*** erect, main branches with many secondary and tertiary flowering branches, pubescent when young with simple spreading hairs. ***Leaves*** 3-nate, erect, imbricate, clasping stems, narrowly oblong, blades 2.0–2.5 × 0.6–0.7 mm, apex acute, sulcate, glabrous; petiole 0.3–0.5 mm long, not decurrent. ***Inflorescence*** of 1- to 3-nate flowers, mostly axillary in upper leaves, sometimes terminal at the tips of small branches; pedicel ± 0.3 mm long, green, glabrous; bracteoles 2, adnate to calyx, lanceolate, 0.5 × 0.4 mm, with ciliate margins; bract adnate to calyx, lanceolate, 1.5–1.7 × ± 0.4 mm, glabrous with ciliate margins. ***Calyx*** equally 4-lobed; sepals adpressed to corolla, lanceolate, 0.7 × 0.4 mm, glabrous, green, margins entire. ***Corolla*** equally 4-lobed, urceolate, 1.2–1.7 × ± 1.7 mm, glabrous, yellow green, fading reddish, lobes half to three-quarters the length of corolla, apex obtuse, margins irregular. ***Stamens*** 6, connate, partially exserted; anthers golden brown, almost sessile, muticous; pore tear-shaped. ***Ovary*** 3-locular, often uniseriate septa, glabrous, smooth; 2 ovules per locule; style 2.0–2.5 mm long, glabrous, stigma exserted, ± 0.3 mm diam., peltate. ***Fruit*** and ***seeds*** not seen. ***Flowering time***: March to May.

**Figure 6.**
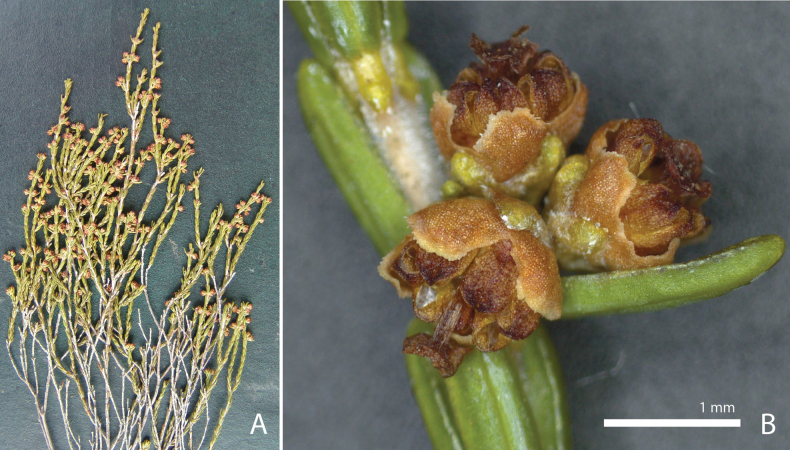
*Ericainopina* J.H.J.Vlok **A** flowering branch **B** single inflorescence. Voucher *J.H.J. Vlok 2988* (NBG).

##### Distribution and habitat.

This species is currently only known from the type locality, where it is locally abundant in deep sandy soil on the ecotone between Arid Fynbos and Renosterveld at the base of a south-facing slope.

##### Threat status.

*Ericainopina* is restricted to a single locality in the Laingsburg district, where fewer than 500 plants were observed with no immediate threats and no significant population decline expected. Based on this data, an IUCN (2012) category Vulnerable under criterion D is recommended.

##### Pollination syndrome.

The exserted style and peltate stigma, small, cup-shaped corolla and the absence of nectaries suggest anemophily.

##### Etymology.

The specific name refers to the surprise to find an *Erica* of this group in an arid inland area.

##### Subgeneric classification.

*Ericainopina* falls within the Coccospermad group (section Ventiflora) of *Erica* species that have a fully recaulescent bract and bracteoles (Oliver 2000).

##### Notes on phylogeny and morphology.

The phylogenetic placement of *E.inopina* suggests *E.subcapitata* as its closest relative. These species are also morphologically similar but differ in several features clarified in the diagnosis above. Strict matching with the *Erica* ID aid yielded no results for *E.inopina*, irrespective of region and flowering times, and *E.subcapitata* featured low (32^nd^) using the “probability” algorithm. This highlights the need to integrate multiple lines of evidence for alpha taxonomy in *Erica*.

Beyond the formal results, *E.inopina* is also morphologically similar to *E.didymocarpa* and *E.parviporandra*, from which it can be distinguished by its glabrous, 3-locular ovary (2- locular in *E.didymocarpa*; hairy, 1-locular in *E.parviporandra*) glabrous sepals (hairy in *E.didymocarpa*), 6 exserted anthers (8, mostly included anthers in *E.didymocarpa*) and fully recaulescent bract and bracteoles (*E.didymocarpa* is ebracteolate).

## Supplementary Material

XML Treatment for Erica arida

XML Treatment for Erica hessequae

XML Treatment for Erica inopina

## ﻿Additional information

### Author ORCIDs

Rendert D. Hoekstra https://orcid.org/0009-0001-1506-7107

Seth D. Musker https://orcid.org/0000-0002-1456-1373

Michael D. Pirie https://orcid.org/0000-0003-0403-4470

Jan H. J. Vlok https://orcid.org/0000-0002-5659-7550

### Data availability

Data that support the findings of this study have been archived on GenBank, iNaturalist and GBIF (PhytoKeys. An integrative approach to alpha taxonomy in Erica L. (Ericaceae) with three new species from the Western Cape, South Africa. Occurrence dataset https://doi.org/10.15468/abecqd accessed via GBIF.org on 2025-05-14.).

## Supplementary materials

Supplementary material 1 Observation data and GenBank accession numbers Data type docx https://binary.pensoft.net/file/1344864This dataset is made available under the Open Database License (http://opendatacommons.org/licenses/odbl/1.0/). The Open Database License (ODbL) is a license agreement intended to allow users to freely share, modify, and use this Dataset while maintaining this same freedom for others, provided that the original source and author(s) are credited.Rendert D. Hoekstra, Seth D. Musker, Michael D. Pirie, Jan H. J. Vlok

Supplementary material 2 Probabilistic identification using the *Erica* ID aid Data type pdf https://binary.pensoft.net/file/1344865This dataset is made available under the Open Database License (http://opendatacommons.org/licenses/odbl/1.0/). The Open Database License (ODbL) is a license agreement intended to allow users to freely share, modify, and use this Dataset while maintaining this same freedom for others, provided that the original source and author(s) are credited.Rendert D. Hoekstra, Seth D. Musker, Michael D. Pirie, Jan H. J. Vlok

Supplementary material 3 Full phylogenetic data and results Data type zip Explanation note Includes data matrices, analysis output, and pdf plots of the full phylogenetic trees: fig. S1: plastid tree; fig. S2: nuclear tree; fig. S3: combined. https://binary.pensoft.net/file/1344866This dataset is made available under the Open Database License (http://opendatacommons.org/licenses/odbl/1.0/). The Open Database License (ODbL) is a license agreement intended to allow users to freely share, modify, and use this Dataset while maintaining this same freedom for others, provided that the original source and author(s) are credited.Rendert D. Hoekstra, Seth D. Musker, Michael D. Pirie, Jan H. J. Vlok

Supplementary material 4 Summarised descriptions of new *Erica* taxa Data type docx https://binary.pensoft.net/file/1344867This dataset is made available under the Open Database License (http://opendatacommons.org/licenses/odbl/1.0/). The Open Database License (ODbL) is a license agreement intended to allow users to freely share, modify, and use this Dataset while maintaining this same freedom for others, provided that the original source and author(s) are credited.Rendert D. Hoekstra, Seth D. Musker, Michael D. Pirie, Jan H. J. Vlok

## References

[B1] Baldwin BG Sanderson MJ Porter JM Wojciechowski MF Campbell S Donoghue MJ Campbell CS Donoghue MJ (1995) The ITS region of nuclear ribosomal DNA: a valuable source of evidence on angiosperm phylogeny. Annals of the Missouri Botanical Garden 82: 247–277. 10.2307/2399880

[B2] Beentje HJ (2006) Ericaceae. In: Beentje HJ Ghazanfar SA (Eds) Flora of Tropical East Africa.Royal Botanic Gardens, Kew, 1–29.

[B3] Brown MJM Bachman SP Nic Lughadha E (2023) Three in four undescribed plant species are threatened with extinction. The New Phytologist 240: 1340–1344. 10.1111/nph.19214 37583098

[B4] Chernomor O Von Haeseler A Minh BQ (2016) Terrace aware data structure for phylogenomic inference from supermatrices. Systematic Biology 65: 997–1008. 10.1093/sysbio/syw037 27121966 PMC5066062

[B5] Dulfer H (1964) Revision der südafrikanischen Arten der Gattung *Erica* L. 1. Teil. Annalen des Naturhistorischen Museums in Wien 67: 79–147.

[B6] Dulfer H (1965) Revision der südafrikanischen Arten der Gattung *Erica* L. 2. Teil (Fortsetzung). Annalen des Naturhistorischen Museums in Wien 68: 25–177.

[B7] Elliott AC Bester SP Klopper RR Nelson EC Pirie MD (2024) Curating an online checklist for *Erica* L. (Ericaceae): Contributing to and supporting global conservation through the World Flora Online. PhytoKeys 243: 121–135. 10.3897/phytokeys.243.121555 38947554 PMC11214009

[B8] Goldblatt P Manning J (2000) Cape plants: a conspectus of the Cape flora of South Africa. Strelitzia 9. National Botanical Institute, Pretoria and Missouri Botanical Garden, St Louis, 743 pp.

[B9] Guindon S Dufayard J-F Lefort V Anisimova M Hordijk W Gascuel O (2010) New Algorithms and Methods to Estimate Maximum-Likelihood Phylogenies: Assessing the Performance of PhyML 3.0. Systematic Biology 59: 307–321. 10.1093/sysbio/syq010 20525638

[B10] Guthrie F Bolus H (1905) Erica. In: Thistleton-Dyer (Ed.) Flora Capensis.Reeve, London, 4–513. https://www.biodiversitylibrary.org/item/15242.

[B11] Hassemer G Prado J Baldini RM (2020) Diagnoses and descriptions in Plant Taxonomy: Are we making proper use of them? Taxon 69: 1–4. 10.1002/tax.12200

[B12] Hoang DT Chernomor O Von Haeseler A Minh BQ Vinh LS (2018) UFBoot2: Improving the Ultrafast Bootstrap Approximation. Molecular Biology and Evolution 35: 518–522. 10.1093/molbev/msx281 29077904 PMC5850222

[B13] IUCN (2012) IUCN Red List Categories and Criteria, Second ed. IUCN, Gland, Switzerland and Cambridge, UK.

[B14] Kalyaanamoorthy S Minh BQ Wong TKF Von Haeseler A Jermiin LS (2017) ModelFinder: Fast model selection for accurate phylogenetic estimates. Nature Methods 14: 587–589. 10.1038/nmeth.4285 28481363 PMC5453245

[B15] Linder HP (2003) The radiation of the Cape flora, southern Africa. Biological Reviews 78: 597–638. 10.1017/S1464793103006171 14700393

[B16] Minh BQ Schmidt HA Chernomor O Schrempf D Woodhams MD Von Haeseler A Lanfear R (2020) IQ-TREE 2: New Models and Efficient Methods for Phylogenetic Inference in the Genomic Era. In: Teeling E (Ed.) Molecular Biology and Evolution 37: 1530–1534. 10.1093/molbev/msaa015 32011700 PMC7182206

[B17] Mucina L Rutherford MC (2006) The vegetation of South Africa, Lesotho and Swaziland. Strelitzia 19. South African National Biodiversity Institute, Pretoria, 801 pp.

[B18] Mugrabi de Kuppler AL Fagúndez J Bellstedt DU Oliver EGH Léon J Pirie MD (2015) Testing reticulate versus coalescent origins of *Ericalusitanica* using a species phylogeny of the northern heathers (Ericeae, Ericaceae). Molecular Phylogenetics and Evolution 88: 121–131. 10.1016/j.ympev.2015.04.005 25888972

[B19] Musker SD Pirie MD Nürk NM (2024) Pollinator shifts despite hybridisation in the Cape’s hyperdiverse heathers (*Erica*, Ericaceae). Molecular Ecology 17505. 10.1111/mec.17505 39188071

[B20] Musker SD Nürk NM Pirie MD (2025) Maximising informativeness for target capture-based phylogenomics in Erica (Ericaceae). PhytoKeys 251: 87–118. 10.3897/phytokeys.251.136373 39867481 PMC11758362

[B21] Nelson EC (2011) Hardy heathers from the northern hemisphere: *Calluna*, *Daboecia*, *Erica*. Royal botanic gardens Kew, Richmond.

[B22] Nelson EC Pirie MD Bellstedt DU (2024) Redefining the megagenus Erica L. (Ericaceae): the contributions of E. G. H. Oliver and I. M. Oliver (née Nitzsche) to taxonomy and nomenclature. PhytoKeys 244: 39–55. 10.3897/phytokeys.244.121705 39006939 PMC11245645

[B23] Oliver EGH (2000) Systematics of Ericaceae (Ericeae-Ericoideae): species with indehiscent and partially dehiscent fruits. Contributions from the Bolus Herbarium 19, 483 pp.

[B24] Oliver EGH Oliver IM (2002) The genus *Erica* (Ericaceae) in southern Africa: Taxonomic notes 1. Bothalia 32: 37–61. 10.4102/abc.v32i1.461

[B25] Oliver EGH Oliver IM (2005) The genus *Erica* (Ericaceae) in southern Africa: Taxonomic notes 2. Bothalia 35: 121–148. 10.4102/abc.v35i2.388

[B26] Oliver EGH Forshaw N Oliver IM Vlok F Schumann AWS Dorr LJ Hoekstra RD Musker SD Nürk NM Pirie MD Rebelo AG (2024a) Genus *Erica*: An Identification Aid Version 4.00. ARPHA Preprints. 10.3897/arphapreprints.e117930 PMC11063621 38699680

[B27] Oliver EGH Forshaw N Oliver IM Volk F Schumann AWS Dorr LJ Hoekstra RD Musker SD Nürk NM Pirie MD Rebelo AG (2024b) Genus *Erica*: An identification aid version 4.00. PhytoKeys 241: 143–154. 10.3897/phytokeys.241.117604 38699680 PMC11063621

[B28] Pirie MD Humphreys AM Barker NP Linder HP (2009) Reticulation, data combination, and inferring evolutionary history: An example from Danthonioideae (Poaceae). Systematic Biology 58: 612–628. 10.1093/sysbio/syp068 20525613

[B29] Pirie MD Oliver EGH Bellstedt DU (2011) A densely sampled ITS phylogeny of the Cape flagship genus *Erica* L. suggests numerous shifts in floral macro-morphology. Molecular Phylogenetics and Evolution 61: 593–601. 10.1016/j.ympev.2011.06.007 21722743

[B30] Pirie MD Oliver EGH Mugrabi de Kuppler A Gehrke B Le Maitre NC Kandziora M Bellstedt DU (2016) The biodiversity hotspot as evolutionary hot-bed: Spectacular radiation of *Erica* in the Cape Floristic Region. BMC Evolutionary Biology 16: 190. 10.1186/s12862-016-0764-3 PMC5027107 27639849

[B31] Pirie MD Oliver EGH Gehrke B Heringer L Mugrabi de Kuppler A Le Maitre NC Bellstedt DU (2017) Underestimated regional species diversity in the Cape Floristic Region revealed by phylogenetic analysis of the *Erica abietina/E.viscaria* clade (Ericaceae). Botanical Journal of the Linnean Society 184: 185–203. 10.1093/botlinnean/box021

[B32] Pirie MD Bellstedt DU Bouman RW Fagúndez J Gehrke B Kandziora M Le Maitre NC Musker SD Newman E Nürk NM Oliver EGH Pipins S van der Niet T Forest F (2024) Spatial decoupling of taxon richness, phylogenetic diversity and threat status in the megagenus *Erica* (Ericaceae). PhytoKeys 244: 127–150. 10.3897/phytokeys.244.124565 39027483 PMC11255470

[B33] Raimondo D Van Staden L Foden W Victor JE Helme NA Turner RC Kamundi DA Manyama PA (2009) Strelitzia No. 25 Red list of South African plants 2009. South African National Biodiversity Institute, Pretoria, [ix +] 668 pp. http://hdl.handle.net/20.500.12143/260

[B34] Shaw J Lickey EB Schilling EE Small RL (2007) Comparison of whole chloroplast genome sequences to choose noncoding regions for phylogenetic studies in angiosperms: The Tortoise and the hare III. American Journal of Botany 94: 275–288. 10.3732/ajb.94.3.275 21636401

[B35] South African National Mapping Agency (2017) 1:50 000 medium scale index. National Geo-Spatial Information. [Retrieved February 6, 2025] https://ngi.dalrrd.gov.za/index.php/what-we-do/maps-and-geospatial-information/35-map-products/51-1-50-000-topographical-maps

[B36] Taberlet P Gielly L Pautou G Bouvet J (1991) Universal primers for amplification of three non-coding regions of chloroplast DNA. Plant Molecular Biology 17: 1105–1109. 10.1007/BF00037152 1932684

[B37] Thiers B (2018) Index Herbariorum: a Global Directory of Public Herbaria and Associated Staff. New York botanical Garden’s virtual herbarium. http://sweetgum.nybg.org/ih

[B38] Van der Niet T Pirie MD Shuttleworth A Johnson SD Midgley JJ (2014) Do pollinator distributions underlie the evolution of pollination ecotypes in the Cape shrub *Ericaplukenetii*? Annals of Botany 113: 301–316. 10.1093/aob/mct193 PMC3890384 24071499

