# Supplementary material for: ﻿An integrative approach to alpha taxonomy in Erica L. (Ericaceae) with three new species from the Western Cape, South Africa
Source: PhytoKeys. 2025 Jun 4;257:95–117. doi: 10.3897/phytokeys.257.139457 (PMC12159662; doi:10.3897/phytokeys.257.139457)
Supplement: Supplementary material 1 — Observation data and GenBank accession numbers [file phytokeys-257-095_article-139457__-s001.docx]

**Supplementary Material 1: Observation data and GenBank accession numbers**

| Collector | No. | Dups | Species | Country | Major area | Locality notes | Coordinates | ITS_GB | TRNL_GB | Link to iNaturalist.org observation |
| --- | --- | --- | --- | --- | --- | --- | --- | --- | --- | --- |
| Vlok, J.H.J. | 2988 | BG | *Erica* sp. "inopina" | South Africa | Western Cape | Touws River, on farm Lettaskraal, in Brandhoek part of farm | 33°26′25″S, 20°22′24″E | PV173805 | PV188032 | - |
| Hoekstra, R.D. | 42 | NBG | *Erica zitzikammensis* Dulfer | South Africa | Western Cape | Uniondale Division, Upper northern slopes around Quaggasberg | 33°35′9.93″S, 23°35′39.49″E | PV173806 | PV188033 | https://www.inaturalist.org/observations/189121495 |
| Hoekstra, R.D. | 77 | NBG | *Erica omninoglabra* H.A.Baker | South Africa | Western Cape | Swellendam Division, Twaalfuurkop ascent | 33°59′1.20″S, 20°26′55.69″E | PV173803 | PV188030 | https://www.inaturalist.org/observations/190225419 |
| Hoekstra, R.D. | 139 | NBG | *Erica pilaarkopensis* H.A.Baker | South Africa | Western Cape | Caledon Division, Pilaarkop ascent | 34°3′39.05″S, 19°50′35.33″E | PV173804 | PV188031 | https://www.inaturalist.org/observations/192859389 |
| Hoekstra, R.D. | 180 | BG, NBG | *Erica macrophylla* Klotzsch ex Benth. | South Africa | Western Cape | Riversdale Division, Sleeping Beauty Peak | 33°57′56.98″S, 21°11′41.85″E | PV173801 | PV188028 | https://www.inaturalist.org/observations/197918938 |
| Hoekstra, R.D. | 181 | BG, NBG | *Erica* sp. "hessequae" | South Africa | Western Cape | Langeberg Range, Romanskraal, north-facing slopes west of Skoorsteen Peak | 33°56′42.92″S, 21°28′55.49″E | PV173807 | PV188034 | https://www.inaturalist.org/observations/199590945 |
| Hoekstra, R.D. | 183 | NBG | *Erica albescens* Klotzsch ex Benth. *var. albescens* Bolus | South Africa | Western Cape | Riversdale Division, Mosambiekkop ("Sleeping Beauty") north of Riversdale, south-facing slopes on the outcrop | 33°57′57.22″S, 21°11′42.26″E | PV173808 | PV188035 | https://www.inaturalist.org/observations/198724327 |
| Hoekstra, R.D. | 184 | BG, NBG | *Erica* *nudiflora* x *peltata* | South Africa | Western Cape | Bredasdorp Division, Soetmuisberg, lower north-facing slopes close to Napier | 34°29′39.10″S, 19°53′41.38″E | PV173809, PV173810 | PV188036 | https://www.inaturalist.org/observations/199590917 |
| Hoekstra, R.D. | 187 | NBG | *Erica mucronata* Andrews | South Africa | Western Cape | Riversdale Division, Boet-se-pad trail from Garcia's Pass | 33°57′37.85″S, 21°9′50.14″E | PV173802 | PV188029 | https://www.inaturalist.org/observations/194922445 |
| Hoekstra, R.D. | 218 | NBG | *Erica* sp. "arida" | South Africa | Western Cape | Overberg, Barrydale outskirts. On steep middle south-facing slopes north of the Doringrivier catchment area | 33°55′19.97″S, 20°46′44.35″E | PV173811 | PV188037 | https://www.inaturalist.org/observations/203756788 |
