# Supplementary material for: ﻿An integrative approach to alpha taxonomy in Erica L. (Ericaceae) with three new species from the Western Cape, South Africa
Source: PhytoKeys. 2025 Jun 4;257:95–117. doi: 10.3897/phytokeys.257.139457 (PMC12159662; doi:10.3897/phytokeys.257.139457)
Supplement: Supplementary material 2 — Probabilistic identification using the Erica ID aid [file phytokeys-257-095_article-139457__-s002.pdf]

Appendix 3: Probabilistic Identification using the Erica ID Aid

Erica sp. RDH181

Region and flowering month excluded		Region included and flowering month excluded		All characters included	
**Species**	**Probability**	**Species**	**Probability**	**Species**	**Probability**
woodii	57,224	heterophylla	57,964	heterophylla	60,976
galantha	55,795	woodii	57,224	podophylla	58,519
setosa	55,795	galantha	55,795	woodii	57,224
andreaei	54,141	setosa	55,795	galantha	55,795
lehmannii	52,765	podophylla	55,507	setosa	55,795
wittebergensis	52,235	andreaei	54,141	lehmannii	55,777
setacea	52,198	brevifolia	52,783	andreaei	54,141
heterophylla	50,770	lehmannii	52,765	condensata	53,869
atherstonei	49,741	wittebergensis	52,235	brevifolia	52,783
involucrata	49,125	setacea	52,198	atherstonei	52,754
diotiflora	48,313	condensata	50,857	wittebergensis	52,235
fausta	48,313	atherstonei	49,741	setacea	52,198
podophylla	48,313	involucrata	49,125	involucrata	52,137
trichadenia	48,313	diotiflora	48,313	diotiflora	51,325
curvirostris	48,205	fausta	48,313	trichadenia	51,325
paniculata	48,095	trichadenia	48,313	flaccida	50,210
lucida	47,210	curvirostris	48,205	fausta	48,313
flaccida	47,198	paniculata	48,095	curvirostris	48,205
brachysepala	46,645	lucida	47,210	paniculata	48,095
pauciovulata	46,119	flaccida	47,198	amoena	48,026
brevifolia	45,589	brachysepala	46,645	capillaris	47,718
viscaria	45,578	pauciovulata	46,119	lucida	47,210
amoena	45,014	viscaria	45,578	pulchella	47,139
capillaris	44,706	amoena	45,014	brachysepala	46,645
gnaphaloides	44,127	capillaris	44,706	pauciovulata	46,119
pulchella	44,127	gnaphaloides	44,127	viscaria	45,578
viscaria subsp. gallorum	44,063	pulchella	44,127	gnaphaloides	44,127
carduifolia	44,017	viscaria subsp. gallorum	44,063	viscaria subsp. gallorum	44,063
aghillana	43,821	carduifolia	44,017	carduifolia	44,017
jacksoniana	43,711	aghillana	43,821	aghillana	43,821
campanularis	43,662	jacksoniana	43,711	jacksoniana	43,711
condensata	43,662	campanularis	43,662	campanularis	43,662
affinis	42,482	affinis	42,482	affinis	42,482
hispidula	26,921	hispidula	34,115	hispidula	37,127
tegetiformis	26,921	eremioides	32,879	eremioides	35,891
pseudocalycina	25,825	atropurpurea	30,229	adunca	32,668
atromontana	25,684	oophylla	30,229	intermedia	31,228
eremioides	25,684	langebergensis	29,731	intermedia subsp. intermedia	31,228
eremioides subsp. eglandula	25,684	melanthera	29,731	muscosa	31,219
floccifera	25,459	physantha	29,731	eremioides subsp. eremioides	30,866
viscidiflora	25,459	rhodella	29,731	albescens	30,673
argentea	25,348	adunca	29,656	dysantha	30,673
cymosa	25,300	chartacea	29,656	oxyandra	30,673
fimbriata	25,300	karooica	29,656	racemosa	30,457
saxicola	25,300	lasciva	29,656	atropurpurea	30,229
dolfiana	25,276	intermedia	28,216	oophylla	30,229
revoluta	25,276	intermedia subsp. intermedia	28,216	imbricata	30,175
schelpeorum	25,276	muscosa	28,207	tegetiformis	29,933
setociliata	25,276	poculiflora	28,159	langebergensis	29,731
strigosa	24,464	eremioides subsp. eremioides	27,854	melanthera	29,731
uysii	24,464	quadrifida	27,854	physantha	29,731
zebrensis	24,464	albescens	27,661	rhodella	29,731
anemodes	24,389	dysantha	27,661	chartacea	29,656
bruniifolia	24,389	oxyandra	27,661	karooica	29,656
crateriformis	24,389	rudolfii	27,661	lasciva	29,656
deflexa	24,389	tradouwensis	27,661	copiosa	29,645
diosmifolia	24,389	nutans	27,628	glomiflora	28,966
emarginata	24,389	leucodesmia	27,469	pseudocalycina	28,837
glumiflora	24,389	racemosa	27,445	eremioides subsp. eglandula	28,697
keeromsbergensis	24,389	cristiflora	27,199	plukenetii	28,660
lachnaeifolia	24,389	cordata	27,163	mucronata	28,639
pudens	24,389	imbricata	27,163	cymosa	28,312
sperata	24,389	macrophylla	27,163	saxicola	28,312
thodei	24,389	tegetiformis	26,921	revoluta	28,288
eremioides subsp. pubescens	24,256	gigantea	26,856	poculiflora	28,159
caprina	24,112	gillii	26,697	multumbellifera	28,024
curvistyla	23,920	copiosa	26,633	quadrifida	27,854
calycina	23,886	grata	26,633	latifolia	27,718
kingaensis subsp. rugegensis	23,886	afra	26,089	rudolfii	27,661
blaerioides	23,871	turneri	26,089	tradouwensis	27,661
blaerioides subsp. hirsuta	23,871	nivalis	26,040	penicilliformis	27,643
oreotragus	23,871	glomiflora	25,954	nutans	27,628
esteriana	23,764	elsieana	25,933	leucodesmia	27,469
esteriana subsp. esteriana	23,764	parviporandra	25,836	deflexa	27,401
villosa	23,565	pseudocalycina	25,825	outeniquae	27,343
roseoloba	23,373	crassisepala	25,734	urceolata	27,276
orientalis	23,294	atromontana	25,684	eremioides subsp. pubescens	27,268
atropurpurea	23,035	eremioides subsp. eglandula	25,684	cristiflora	27,199
coronanthera	23,035	plukenetii	25,648	cordata	27,163
oophylla	23,035	mucronata	25,627	macrophylla	27,163
patens	23,035	floccifera	25,459	caprina	27,124
thimifolia	23,035	viscidiflora	25,459	velatiflora	27,102
recurvifolia	22,960	anguliger	25,397	nematophylla	27,015
inamoena	22,891	rosacea	25,397	longimontana	26,980
amphigena	22,817	rosacea subsp. rosacea	25,397	curvistyla	26,932
subulata	22,817	argentea	25,348	calycina	26,898
cymosa subsp. cymosa	22,768	papyracea	25,341	blaerioides	26,883
sagittata	22,650	cymosa	25,300	blaerioides subsp. hirsuta	26,883
blaerioides subsp. blaerioides	22,635	fimbriata	25,300	oreotragus	26,883
langebergensis	22,537	saxicola	25,300	gigantea	26,856
lavandulifolia	22,537	dolfiana	25,276	esteriana	26,776
macrotrema	22,537	revoluta	25,276	esteriana subsp. esteriana	26,776
maritima	22,537	schelpeorum	25,276	gillii	26,697
melanthera	22,537	setociliata	25,276	grata	26,633
physantha	22,537	euryphylla	25,167	ixanthera	26,487
pilaarkopensis	22,537	multumbellifera	25,012	viscosissima	26,482
rhodella	22,537	sexfaria	24,986	orientalis	26,306
boucheri	22,528	syngenesia	24,937	afra	26,089
rigidula	22,528	comata	24,706	turneri	26,089
adunca	22,462	granulatifolia	24,706	nivalis	26,040
anomala	22,462	latifolia	24,706	recurvifolia	25,972
chartacea	22,462	mundii	24,706	elsieana	25,933
desmantha	22,462	pearsoniana	24,706	parviporandra	25,836
hebdomadalis	22,462	burchelliana	24,697	cymosa subsp. cymosa	25,780
karooica	22,462	penicilliformis	24,631	crassisepala	25,734
lasciva	22,462	pubigera	24,578	atromontana	25,684
magnisylvae	22,462	strigosa	24,464	blaerioides subsp. blaerioides	25,647
altiphila	22,335	uysii	24,464	plukenetii subsp. plukenetii	25,626
esteriana subsp. swartbergensis	22,191	zebrensis	24,464	discolor	25,598
cetrata	22,103	oliveranthus	24,435	discolor variant B 'speciosa'	25,598
banksii	22,017	anemodes	24,389	lavandulifolia	25,549
extrusa	21,932	bruniifolia	24,389	floccifera	25,459
nigrimontana	21,932	crateriformis	24,389	viscidiflora	25,459
wildii	21,932	deflexa	24,389	anguliger	25,397
harveiana	21,895	diosmifolia	24,389	rosacea	25,397
magistrati	21,895	emarginata	24,389	rosacea subsp. rosacea	25,397
oakesiorum	21,895	glumiflora	24,389	densifolia	25,387
rivularis	21,895	keeromsbergensis	24,389	argentea	25,348
tragulifera	21,895	lachnaeifolia	24,389	altiphila	25,347
umbratica	21,895	pudens	24,389	papyracea	25,341
intermedia subsp. albiflora	21,834	sperata	24,389	fimbriata	25,300
leucanthera	21,801	thodei	24,389	dolfiana	25,276
plumosa	21,799	outeniquae	24,331	schelpeorum	25,276
wyliei	21,414	urceolata	24,264	setociliata	25,276
navigatoris	21,355	eremioides subsp. pubescens	24,256	euryphylla	25,167
serrata	21,307	tenuicaulis	24,129	glandulosa	25,033
loganii	21,306	caprina	24,112	glandulosa subsp. glandulosa	25,033
xeranthemifolia	21,226	brachycentra	24,101	sexfaria	24,986
swaziensis	21,198	juniperina	24,101	sessiliflora	24,948
trichophylla	21,196	velatiflora	24,090	extrusa	24,944
cooperi	21,131	nematophylla	24,003	syngenesia	24,937
priorii	21,075	longimontana	23,968	rivularis	24,908
arachnocalyx	21,034	vallis-fluminis	23,968	umbratica	24,908
petrophila	21,034	curvistyla	23,920	intermedia subsp. albiflora	24,846
pogonanthera	21,034	calycina	23,886	comata	24,706
intermedia	21,022	kingaensis subsp. rugegensis	23,886	granulatifolia	24,706
intermedia subsp. intermedia	21,022	blaerioides	23,871	mundii	24,706
melastoma	21,022	blaerioides subsp. hirsuta	23,871	pearsoniana	24,706
melastoma subsp. melastoma	21,022	oreotragus	23,871	burchelliana	24,697
melastoma subsp. minor	21,022	esteriana	23,764	cubitans	24,669
muscosa	21,013	esteriana subsp. esteriana	23,764	pubigera	24,578
poculiflora	20,964	villosa	23,565	daphniflora	24,556
phacelanthera	20,907	madida	23,476	infundibuliformis	24,483
arcuata	20,800	ixanthera	23,475	vestita	24,479
unilateralis	20,800	viscosissima	23,470	strigosa	24,464
inconstans	20,665	ardens	23,422	uysii	24,464
angulosa	20,659	roseoloba	23,373	zebrensis	24,464
eremioides subsp. eremioides	20,659	orientalis	23,294	oliveranthus	24,435
fuscescens	20,659	ocellata	23,134	wyliei	24,426
quadrifida	20,659	coronanthera	23,035	anemodes	24,389
uberiflora	20,659	patens	23,035	bruniifolia	24,389

Erica sp. RDH183

Region and flowering month excluded		Region included and flowering month excluded		All characters included	
**Species**	**Probability**	**Species**	**Probability**	**Species**	**Probability**
viscaria	40,057	albescens	41,648	albescens	44,716
varderi	38,274	oxyandra	41,648	oxyandra	44,716
distorta	36,708	cristiflora	41,091	viscaria	43,124
trachysantha	35,824	viscaria	40,057	cristiflora	41,091
heleogena	34,709	mucronata	39,519	heterophylla	40,852
paludicola	34,709	cubica	38,571	podophylla	40,197
albescens	34,454	eremioides	38,351	distorta	39,776
oxyandra	34,454	eremioides subsp. eremioides	38,351	mucronata	39,519
carduifolia	34,251	afra	38,274	cubica	38,571
chrysocodon	34,179	varderi	38,274	eremioides	38,351
cristiflora	33,897	heterophylla	37,785	eremioides subsp. eremioides	38,351
zitzikammensis	33,897	mundii	37,264	afra	38,274
flanaganii	32,809	podophylla	37,130	varderi	38,274
ovina	32,652	distorta	36,708	heleogena	37,777
concordia	32,527	syngenesia	36,301	paludicola	37,777
cryptanthera	32,392	trachysantha	35,824	carduifolia	37,319
cymosa	32,392	heleogena	34,709	mundii	37,264
cymosa subsp. grandiflora	32,392	paludicola	34,709	syngenesia	36,301
galantha	32,392	carduifolia	34,251	flanaganii	35,877
myriocodon	32,392	chrysocodon	34,179	trachysantha	35,824
setosa	32,392	zitzikammensis	33,897	concordia	35,595
woodii	32,392	flanaganii	32,809	cymosa	35,460
mucronata	32,325	omninoglabra	32,802	cymosa subsp. grandiflora	35,460
hippurus	31,997	oliveranthus	32,667	woodii	35,460
limnophila	31,997	ovina	32,652	georgica	35,339
oresigena	31,997	concordia	32,527	copiosa	34,850
cubica	31,377	tetrathecoides	32,503	chrysocodon	34,179
eremioides	31,156	cryptanthera	32,392	zitzikammensis	33,897
eremioides subsp. eremioides	31,156	cymosa	32,392	tetragona	33,694
afra	31,080	cymosa subsp. grandiflora	32,392	natalitia	33,533
setulosa	30,883	galantha	32,392	lignosa	33,420
curvistyla	30,820	myriocodon	32,392	phillipsii	33,420
plumosa	30,761	setosa	32,392	umbrosa	33,420
abbottii	30,748	woodii	32,392	coacervata	33,263
heterophylla	30,591	georgica	32,271	cederbergensis	33,003
setacea	30,591	hippurus	31,997	trichadenia	33,003
natalitia	30,466	limnophila	31,997	wyliei	33,003
lignosa	30,352	oresigena	31,997	omninoglabra	32,802
phillipsii	30,352	melanthera	31,917	oliveranthus	32,667
umbrosa	30,352	copiosa	31,782	ovina	32,652
coacervata	30,195	papyracea	31,254	tetrathecoides	32,503
mundii	30,070	setulosa	30,883	cryptanthera	32,392
cederbergensis	29,935	curvistyla	30,820	galantha	32,392
fausta	29,935	plumosa	30,761	myriocodon	32,392
podophylla	29,935	abbottii	30,748	setosa	32,392
trichadenia	29,935	tenuicaulis	30,740	bracteolaris	32,197
wyliei	29,935	tetragona	30,627	hippurus	31,997
pudens	29,106	setacea	30,591	limnophila	31,997
syngenesia	29,106	natalitia	30,466	oresigena	31,997
viscaria subsp. gallorum	28,821	dysantha	30,412	melanthera	31,917
hottentotica	28,550	rudolfii	30,412	johnstoniana	31,618
johnstoniana	28,550	lignosa	30,352	parilis	31,493
parilis	28,425	phillipsii	30,352	canescens	31,358
canescens	28,291	umbrosa	30,352	longipedunculata	31,336
longipedunculata	28,268	coacervata	30,195	papyracea	31,254
diotiflora	28,134	filamentosa	30,184	condensata	31,121
eriocodon	28,009	brevifolia	29,980	monantha	31,076
monantha	28,009	cederbergensis	29,935	oophylla	31,023
oraria	27,766	fausta	29,935	setulosa	30,883
involucrata	27,555	trichadenia	29,935	oraria	30,833
amphigena	27,534	wyliei	29,935	curvistyla	30,820
tenella	27,462	comata	29,855	plumosa	30,761
cincta	27,305	quadrangularis	29,220	abbottii	30,748
petiolaris	27,273	anguliger	29,155	tenuicaulis	30,740
algida	27,195	bracteolaris	29,129	setacea	30,591
deflexa	27,045	pudens	29,106	tenella	30,529
goatcheriana	26,649	pubigera	29,072	dysantha	30,412
macroloma	26,649	tradouwensis	29,021	rudolfii	30,412
modesta	26,649	turneri	28,840	cincta	30,372
schumannii	26,649	viscaria subsp. gallorum	28,821	filamentosa	30,184
sphaerocephala	26,649	nivalis	28,611	deflexa	30,112
wildii	26,649	hottentotica	28,550	brevifolia	29,980
viscaria subsp. viscaria	26,624	johnstoniana	28,550	fausta	29,935
recta	26,620	parilis	28,425	nematophylla	29,908
stokoei	26,489	canescens	28,291	comata	29,855
lutea	26,194	longipedunculata	28,268	sphaerocephala	29,717
cumuliflora	26,168	trichophora	28,211	viscaria subsp. viscaria	29,691
triflora	25,964	diotiflora	28,134	stokoei	29,556
notoporina	25,889	condensata	28,053	lutea	29,261
physodes	25,889	eriocodon	28,009	quadrangularis	29,220
desmantha	25,789	monantha	28,009	anguliger	29,155
lusitanica	25,732	atropurpurea	27,955	pudens	29,106
andreaei	25,704	oophylla	27,955	muscosa	29,087
floccifera	25,704	oraria	27,766	pubigera	29,072
lateralis	25,660	involucrata	27,555	tradouwensis	29,021
viscidiflora	25,654	amphigena	27,534	notoporina	28,957
flaccida	25,628	tenella	27,462	turneri	28,840
omninoglabra	25,607	cincta	27,305	viscaria subsp. gallorum	28,821
argentea	25,472	petiolaris	27,273	lusitanica	28,800
oliveranthus	25,472	algida	27,195	lateralis	28,727
paniculata	25,472	deflexa	27,045	flaccida	28,696
pyxidiflora	25,400	nematophylla	26,841	nivalis	28,611
lowryensis	25,378	vallis-fluminis	26,719	hottentotica	28,550
acuta	25,309	crassisepala	26,684	longimontana	28,396
tetrathecoides	25,309	goatcheriana	26,649	trichophora	28,211
limosa	25,118	macroloma	26,649	salteri	28,185
salteri	25,118	modesta	26,649	diotiflora	28,134
georgica	25,077	schumannii	26,649	hispidula	28,132
mallotocalyx	25,005	sphaerocephala	26,649	velatiflora	28,106
velitaris	25,005	wildii	26,649	velitaris	28,072
glandulifera	24,848	viscaria subsp. viscaria	26,624	eriocodon	28,009
tegulifolia	24,848	recta	26,620	atropurpurea	27,955
trichroma	24,848	stokoei	26,489	glandulifera	27,915
transparens	24,794	lutea	26,194	transparens	27,862
melanthera	24,723	cumuliflora	26,168	atherstonei	27,655
atherstonei	24,588	cubitans	26,029	consobrina	27,655
consobrina	24,588	euryphylla	26,029	involucrata	27,555
copiosa	24,588	muscosa	26,019	amphigena	27,534
truncata	24,588	triflora	25,964	corifolia	27,459
xeranthemifolia	24,553	notoporina	25,889	petiolaris	27,273
corifolia	24,392	physodes	25,889	calycina	27,199
andevalensis	24,319	desmantha	25,789	algida	27,195
calycina	24,132	lusitanica	25,732	sitiens	27,155
sitiens	24,088	andreaei	25,704	equisetifolia	27,149
equisetifolia	24,082	floccifera	25,704	vestita	27,120
papyracea	24,060	lateralis	25,660	vogelpoelii	26,804
curvirostris	23,902	viscidiflora	25,654	obliqua	26,732
vogelpoelii	23,737	flaccida	25,628	vallis-fluminis	26,719
lehmannii	23,727	argentea	25,472	crassisepala	26,684
obliqua	23,664	paniculata	25,472	goatcheriana	26,649
passerina	23,627	pyxidiflora	25,400	macroloma	26,649
sagittata	23,567	lowryensis	25,378	modesta	26,649
tenuicaulis	23,546	longimontana	25,328	schumannii	26,649
laevigata	23,507	acuta	25,309	wildii	26,649
tetragona	23,432	limosa	25,118	recta	26,620
umbellata	23,332	salteri	25,118	laevigata	26,575
pauciovulata	23,316	ardens	25,065	cerinthoides	26,481
orculiflora	23,275	hispidula	25,065	cerinthoides var. cerinthoides	26,481
pulchella	23,247	velatiflora	25,039	orculiflora	26,343
blaerioides	23,218	mallotocalyx	25,005	glomiflora	26,330
blaerioides subsp. hirsuta	23,218	velitaris	25,005	ixanthera	26,330
dysantha	23,218	glandulifera	24,848	pulchella	26,315
oreotragus	23,218	tegulifolia	24,848	oreotragus	26,286
pubescens	23,218	trichroma	24,848	pubescens	26,286
rudolfii	23,218	transparens	24,794	tegetiformis	26,286
tegetiformis	23,218	puberuliflora	24,792	macrophylla	26,205
goatcheriana var. goatcheriana	23,203	atherstonei	24,588	cumuliflora	26,168
wittebergensis	23,197	consobrina	24,588	cubitans	26,029
intricata	23,015	truncata	24,588	euryphylla	26,029
tarantulae	23,015	xeranthemifolia	24,553	amoena	26,011
filamentosa	22,990	corifolia	24,392	triflora	25,964
amoena	22,943	stylaris	24,355	physodes	25,889
haematocodon	22,943	andevalensis	24,319	desmantha	25,789
mauritanica	22,943	calycina	24,132	andreaei	25,704
bicolor	22,786	sitiens	24,088	floccifera	25,704
brevifolia	22,786	equisetifolia	24,082	viscidiflora	25,654
holosericea	22,786	vestita	24,053	subdivaricata	25,555
lucida	22,786	curvirostris	23,902	argentea	25,472
whyteana	22,747	vogelpoelii	23,737	paniculata	25,472
campanularis	22,661	lehmannii	23,727	pyxidiflora	25,400
comata	22,661	obliqua	23,664	depressa	25,398

Erica sp. RDH184

Region and flowering month excluded		Region included and flowering month excluded		All characters included	
**Species**	**Probability**	**Species**	**Probability**	**Species**	**Probability**
cymosa	36,976	axilliflora	41,814	axilliflora	41,814
diotiflora	34,217	nudiflora	41,158	canescens	41,379
zitzikammensis	34,025	aghillana	39,696	nudiflora	41,158
galantha	33,748	canescens	38,311	pulchella	40,458
floccifera	33,715	uysii	37,465	cymosa	40,043
woodii	33,102	pulchella	37,391	aghillana	39,696
cristiflora	32,645	cymosa	36,976	brachysepala	37,635
axilliflora	32,381	elimensis	35,906	imbricata	37,582
heterophylla	32,143	anguliger	34,884	uysii	37,465
trichadenia	32,143	brachysepala	34,568	serrata	37,274
calycina	32,142	imbricata	34,515	woodii	36,169
eriocodon	32,127	diotiflora	34,217	plukenetii	36,067
tarantulae	31,999	serrata	34,206	elimensis	35,906
cymosa subsp. grandiflora	31,951	maritima	34,045	subdivaricata	35,899
eremioides	31,866	zitzikammensis	34,025	chonantha	35,696
nudiflora	31,724	parviflora	33,831	axillaris	35,653
setosa	31,673	galantha	33,748	melastoma	35,598
argentea	31,530	floccifera	33,715	melastoma subsp. melastoma	35,598
langebergensis	31,497	propinqua	33,218	calycina	35,210
lavandulifolia	31,497	woodii	33,102	heterophylla	35,210
pilaarkopensis	31,497	plukenetii	33,000	trichadenia	35,210
rhodella	31,497	subdivaricata	32,832	cymosa subsp. grandiflora	35,018
lehmannii	31,254	cristiflora	32,645	anguliger	34,884
wittebergensis	31,254	chonantha	32,629	saxicola	34,808
mucronata	31,073	axillaris	32,586	lavandulifolia	34,564
consobrina	31,040	melastoma	32,530	muscosa	34,523
lucida	31,040	melastoma subsp. melastoma	32,530	viscaria subsp. longifolia	34,385
viscaria	30,952	melastoma subsp. minor	32,530	diotiflora	34,217
deflexa	30,570	heterophylla	32,143	banksii	34,136
brevicaulis	30,537	trichadenia	32,143	consobrina	34,107
aghillana	30,262	calycina	32,142	maritima	34,045
viscidiflora	30,249	eriocodon	32,127	zitzikammensis	34,025
comata	30,151	tarantulae	31,999	viscaria	34,019
natalitia	30,151	cymosa subsp. grandiflora	31,951	parviflora	33,831
newdigateae	30,151	eremioides	31,866	galantha	33,748
physantha	30,106	berzelioides	31,859	mammosa	33,739
curvistyla	30,101	saxicola	31,741	floccifera	33,715
limosa	30,065	setosa	31,673	deflexa	33,638
wyliei	30,053	argentea	31,530	curviflora	33,282
andreaei	30,019	lasciva	31,514	natalitia	33,219
pyramidalis	29,954	langebergensis	31,497	propinqua	33,218
poculiflora	29,924	lavandulifolia	31,497	bredasiana	33,170
comorensis	29,676	pilaarkopensis	31,497	didymocarpa	33,170
salteri	29,596	rhodella	31,497	wyliei	33,120
viscaria subsp. gallorum	29,437	muscosa	31,455	plukenetii subsp. plukenetii	33,033
strigosa	29,422	viscaria subsp. longifolia	31,317	discolor	32,852
algida	29,403	lehmannii	31,254	salteri	32,663
chamissonis	29,056	wittebergensis	31,254	cristiflora	32,645
dissimulans	28,965	mucronata	31,073	melastoma subsp. minor	32,530
hexensis	28,965	banksii	31,068	brownii	32,218
canescens	28,877	consobrina	31,040	eriocodon	32,127
amoena	28,844	lucida	31,040	chamissonis	32,124
monantha	28,723	viscaria	30,952	dissimulans	32,033
heleogena	28,420	sperata	30,830	tarantulae	31,999
atherstonei	28,077	mammosa	30,671	amoena	31,911
bicolor	28,077	deflexa	30,570	eremioides	31,866
copiosa	28,077	brevicaulis	30,537	berzelioides	31,859
setacea	28,077	radicans	30,278	monantha	31,790
trachysantha	28,077	viscidiflora	30,249	glabella	31,773
uysii	28,031	curviflora	30,214	setosa	31,673
gnaphaloides	27,957	curtophylla	30,204	argentea	31,530
pulchella	27,957	comata	30,151	lasciva	31,514
carduifolia	27,847	natalitia	30,151	langebergensis	31,497
petiolaris	27,332	newdigateae	30,151	pilaarkopensis	31,497
cubica	27,205	physantha	30,106	rhodella	31,497
canaliculata	27,117	bredasiana	30,102	heleogena	31,488
tenuicaulis	27,117	didymocarpa	30,102	lehmannii	31,254
euryphylla	26,932	curvistyla	30,101	wittebergensis	31,254
eremioides subsp. eremioides	26,841	limosa	30,065	atherstonei	31,144
gossypioides	26,716	wyliei	30,053	copiosa	31,144
cederbergensis	26,648	gracilipes	30,052	mucronata	31,073
fausta	26,648	andreaei	30,019	lucida	31,040
podophylla	26,648	plukenetii subsp. plukenetii	29,965	carduifolia	30,914
cordata	26,472	pyramidalis	29,954	viscosissima	30,852
elimensis	26,472	poculiflora	29,924	sperata	30,830
granulatifolia	26,472	discolor	29,784	brevicaulis	30,537
longipedunculata	26,472	comorensis	29,676	regia	30,319
affinis	26,312	salteri	29,596	regia subsp. regia	30,319
jacksoniana	26,048	bruniifolia	29,439	radicans	30,278
paniculata	26,048	viscaria subsp. gallorum	29,437	viscidiflora	30,249
hispidula	26,002	strigosa	29,422	curtophylla	30,204
macrotrema	26,002	algida	29,403	canaliculata	30,185
melanthera	26,002	brownii	29,150	comata	30,151
campanularis	25,999	chamissonis	29,056	newdigateae	30,151
condensata	25,999	radicans subsp. schlechteri	29,048	discolor variant A 'discolor'	30,136
pauciovulata	25,999	capitata	28,983	physantha	30,106
oxysepala	25,915	calcicola	28,977	curvistyla	30,101
pyxidiflora	25,843	dissimulans	28,965	limosa	30,065
flaccida	25,687	hexensis	28,965	gracilipes	30,052
anguliger	25,450	amoena	28,844	andreaei	30,019
cymosa subsp. cymosa	25,270	ocellata	28,839	pyramidalis	29,954
rusticula	25,268	monantha	28,723	poculiflora	29,924
exleeana	25,242	glabella	28,705	gossypioides	29,784
brachysepala	25,134	glabella subsp. laevis	28,705	cederbergensis	29,716
imbricata	25,081	tristis	28,640	podophylla	29,716
latifolia	25,081	heleogena	28,420	corifolia	29,712
saptouensis	25,081	arenaria	28,336	comorensis	29,676
oligantha	25,043	atherstonei	28,077	cerinthoides	29,594
cryptanthera	25,027	bicolor	28,077	cerinthoides var. cerinthoides	29,594
myriocodon	25,027	copiosa	28,077	longipedunculata	29,539
montis-hominis	24,993	setacea	28,077	bruniifolia	29,439
pseudocalycina	24,907	trachysantha	28,077	viscaria subsp. gallorum	29,437
nemorosa	24,899	gnaphaloides	27,957	strigosa	29,422
nervata	24,851	riparia	27,930	algida	29,403
stokoei	24,811	carduifolia	27,847	hispidula	29,070
esteriana	24,772	viscosissima	27,784	condensata	29,067
esteriana subsp. esteriana	24,772	puberuliflora	27,746	radicans subsp. schlechteri	29,048
serrata	24,772	occulta	27,613	plukenetii subsp. lineata	29,000
thomensis	24,651	platycalyx	27,448	capitata	28,983
maritima	24,611	petiolaris	27,332	oxysepala	28,983
coronanthera	24,574	regia	27,251	calcicola	28,977
priorii	24,540	regia subsp. regia	27,251	hexensis	28,965
inamoena	24,430	cubica	27,205	ocellata	28,839
parviflora	24,397	canaliculata	27,117	flaccida	28,755
roseoloba	24,382	tenuicaulis	27,117	glabella subsp. laevis	28,705
revoluta	24,358	discolor variant A 'discolor'	27,068	regia subsp. mariae	28,698
papyracea	24,328	euryphylla	26,932	tristis	28,640
curvirostris	24,083	eremioides subsp. eremioides	26,841	arenaria	28,336
spumosa	23,940	gossypioides	26,716	exleeana	28,309
varderi	23,932	cederbergensis	26,648	bicolor	28,077
tegetiformis	23,928	fausta	26,648	setacea	28,077
brevifolia	23,924	podophylla	26,648	trachysantha	28,077
truncata	23,924	corifolia	26,644	coccinea	28,041
propendens	23,871	cerinthoides	26,526	coccinea subsp. uniflora	28,041
acuta	23,808	cerinthoides var. cerinthoides	26,526	pseudocalycina	27,975
propinqua	23,784	cordata	26,472	gnaphaloides	27,957
passerina	23,756	granulatifolia	26,472	riparia	27,930
marlothii	23,652	longipedunculata	26,472	stokoei	27,879
involucrata	23,613	affinis	26,312	oblongiflora	27,787
plukenetii	23,566	rubiginosa	26,307	puberuliflora	27,746
plukenetii subsp. bredensis	23,566	pulvinata	26,151	occulta	27,613
plukenetii subsp. penicillata	23,566	calcareophila	26,098	roseoloba	27,449
boucheri	23,536	jacksoniana	26,048	platycalyx	27,448
rigidula	23,536	paniculata	26,048	revoluta	27,425
terniflora	23,536	hispidula	26,002	petiolaris	27,332
anomala	23,471	macrotrema	26,002	tenella	27,228
crateriformis	23,471	melanthera	26,002	cubica	27,205
karooica	23,471	campanularis	25,999	tenuicaulis	27,117
magnisylvae	23,471	condensata	25,999	spumosa	27,008
aneimena	23,436	pauciovulata	25,999	tegetiformis	26,995
oliveranthus	23,422	plukenetii subsp. lineata	25,933	monadelphia	26,946
subdivaricata	23,398	oxysepala	25,915	euryphylla	26,932
abbottii	23,383	pyxidiflora	25,843	eremioides subsp. eremioides	26,841
altiphila	23,344	longiaristata	25,736	fausta	26,648
plumosa	23,338	flaccida	25,687	plukenetii subsp. bredensis	26,633
empetrina	23,315	regia subsp. mariae	25,631	plukenetii subsp. penicillata	26,633
abietina	23,312	cymosa subsp. cymosa	25,270	terniflora	26,604
thomae	23,312	rusticula	25,268	cordata	26,472
esteriana subsp. swartbergensis	23,200	agglutinans	25,253	granulatifolia	26,472
capillaris	23,195	vernicosa	25,253	coccinea subsp. coccinea	26,396
chonantha	23,195	exleeana	25,242	abietina	26,379

Erica sp. RDH218

Region and flowering month excluded		Region included and flowering month excluded		All characters included	
**Species**	**Probability**	**Species**	**Probability**	**Species**	**Probability**
tenuicaulis	39,425	tenuicaulis	46,619	tenuicaulis	46,619
argentea	37,351	oliveranthus	42,924	oliveranthus	42,924
oliveranthus	35,730	argentea	37,351	heterophylla	40,306
subdivaricata	35,706	heterophylla	36,460	capillaris	39,349
capillaris	35,503	comata	36,113	chonantha	39,349
chonantha	35,503	subdivaricata	35,706	natalitia	38,417
natalitia	34,571	cubica	35,561	plukenetii	38,385
equisetifolia	33,428	capillaris	35,503	copiosa	37,885
brevicaulis	33,092	chonantha	35,503	argentea	37,351
depressa	33,017	eremioides	35,259	equisetifolia	37,274
nivea	33,017	eremioides subsp. eremioides	35,259	daphniflora	37,245
campanularis	32,816	omninoglabra	34,914	depressa	36,863
viscidiflora	32,765	anguliger	34,607	plukenetii subsp. plukenetii	36,584
comorensis	32,105	natalitia	34,571	podophylla	36,456
paniculata	32,039	plukenetii	34,539	comata	36,113
pyxidiflora	31,553	copiosa	34,039	subdivaricata	35,706
viscaria	31,536	latifolia	33,597	cubica	35,561
calycina	31,376	cristiflora	33,582	eremioides	35,259
floccifera	31,376	mucronata	33,582	eremioides subsp. eremioides	35,259
curvistyla	31,068	equisetifolia	33,428	calycina	35,222
oxysepala	30,741	daphniflora	33,398	omninoglabra	34,914
lehmannii	30,648	albens	33,293	georgica	34,682
paludicola	30,625	brevicaulis	33,092	anguliger	34,607
tarantulae	30,039	depressa	33,017	paludicola	34,472
viscaria subsp. gallorum	30,021	nivea	33,017	condensata	34,005
galantha	29,947	campanularis	32,816	infundibuliformis	33,798
jacksoniana	29,582	viscidiflora	32,765	latifolia	33,597
myriocodon	29,447	plukenetii subsp. plukenetii	32,737	cristiflora	33,582
woodii	29,301	podophylla	32,610	mucronata	33,582
heterophylla	29,266	amicorum	32,169	multumbellifera	33,337
canaliculata	28,919	comorensis	32,105	albens	33,293
comata	28,919	quadrangularis	32,091	ixanthera	33,262
newdigateae	28,919	paniculata	32,039	hispidula	33,242
rusticula	28,803	conferta	31,878	brevicaulis	33,092
pauciovulata	28,617	pyxidiflora	31,553	tetragona	33,037
distorta	28,536	viscaria	31,536	nivea	33,017
amoena	28,433	calycina	31,376	bracteolaris	32,880
cubica	28,367	floccifera	31,376	campanularis	32,816
propendens	28,227	curvistyla	31,068	canaliculata	32,765
bakeri	28,176	polifolia	31,066	viscidiflora	32,765
comorensis subsp. anjurensis	28,128	georgica	30,836	intermedia	32,733
eremioides	28,065	oxysepala	30,741	intermedia subsp. intermedia	32,733
eremioides subsp. eremioides	28,065	lehmannii	30,648	distorta	32,382
aghillana	28,049	paludicola	30,625	amoena	32,279
setosa	27,873	condensata	30,159	amicorum	32,169
chamissonis	27,824	ocellata	30,134	comorensis	32,105
humifusa	27,794	tarantulae	30,039	quadrangularis	32,091
inamoena	27,794	viscaria subsp. gallorum	30,021	paniculata	32,039
omninoglabra	27,719	infundibuliformis	29,952	conferta	31,878
andreaei	27,681	galantha	29,947	sessiliflora	31,723
cymosa	27,681	pycnantha	29,703	chamissonis	31,670
cymosa subsp. grandiflora	27,681	jacksoniana	29,582	humifusa	31,640
pulchella	27,546	multumbellifera	29,491	pyxidiflora	31,553
setacea	27,499	myriocodon	29,447	viscaria	31,536
monantha	27,490	ixanthera	29,416	cymosa	31,527
anguliger	27,412	cordata	29,396	cymosa subsp. grandiflora	31,527
plukenetii	27,345	hispidula	29,396	pulchella	31,392
plukenetii subsp. bredensis	27,345	macrophylla	29,396	floccifera	31,376
plukenetii subsp. breviflora	27,345	woodii	29,301	steinbergiana	31,365
plukenetii subsp. lineata	27,345	tetrathecoides	29,262	praenitens	31,341
plukenetii subsp. penicillata	27,345	tetragona	29,191	monantha	31,337
zitzikammensis	27,298	papyracea	29,183	plukenetii subsp. bredensis	31,191
axilliflora	27,047	bracteolaris	29,034	plukenetii subsp. lineata	31,191
galgebergensis	26,916	canaliculata	28,919	plukenetii subsp. penicillata	31,191
atherstonei	26,844	newdigateae	28,919	curvistyla	31,068
copiosa	26,844	intermedia	28,887	polifolia	31,066
trachysantha	26,844	intermedia subsp. intermedia	28,887	oxysepala	30,741
deflexa	26,770	rusticula	28,803	atherstonei	30,690
petiolaris	26,755	pauciovulata	28,617	lehmannii	30,648
chiroptera	26,483	distorta	28,536	deflexa	30,616
multiflexuosa	26,483	amoena	28,433	chiroptera	30,329
latifolia	26,403	propendens	28,227	multiflexuosa	30,329
cristiflora	26,387	bakeri	28,176	costatisepala	30,221
mucronata	26,387	elsieana	28,166	ocellata	30,134
costatisepala	26,374	comorensis subsp. anjurensis	28,128	urceolata	30,085
krugeri	26,328	filamentosa	28,102	nematophylla	30,082
cryptanthera	26,252	brevifolia	28,084	imbricata	30,049
daphniflora	26,204	aghillana	28,049	vestita	30,047
thunbergii	26,204	dysantha	27,967	tarantulae	30,039
albens	26,099	sessiliflora	27,877	viscaria subsp. gallorum	30,021
coarctata	26,075	setosa	27,873	galantha	29,947
carduifolia	25,887	chamissonis	27,824	albescens	29,739
intricata	25,887	humifusa	27,794	oxyandra	29,739
eriocodon	25,870	inamoena	27,794	pycnantha	29,703
limosa	25,795	andreaei	27,681	salteri	29,641
salteri	25,795	cymosa	27,681	jacksoniana	29,582
gnaphaloides	25,744	cymosa subsp. grandiflora	27,681	glandulosa	29,493
diotiflora	25,689	benthamiana	27,643	glandulosa subsp. glandulosa	29,493
navigatoris	25,645	seriphiifolia	27,617	myriocodon	29,447
plukenetii subsp. plukenetii	25,543	pulchella	27,546	cordata	29,396
cederbergensis	25,416	steinbergiana	27,519	macrophylla	29,396
fausta	25,416	klotzschii	27,507	adunca	29,320
podophylla	25,416	setacea	27,499	woodii	29,301
trichadenia	25,416	praenitens	27,495	cederbergensis	29,262
revoluta	25,411	monantha	27,490	tetrathecoides	29,262
simulans	25,397	trichophora	27,372	trichadenia	29,262
azorica	25,262	plukenetii subsp. bredensis	27,345	muscosa	29,261
scoparia	25,262	plukenetii subsp. breviflora	27,345	revoluta	29,257
sylvainiana	25,160	plukenetii subsp. lineata	27,345	papyracea	29,183
flaccida	25,110	plukenetii subsp. penicillata	27,345	flaccida	28,956
bicolor	25,042	zitzikammensis	27,298	newdigateae	28,919
wittebergensis	24,997	dodii	27,116	rusticula	28,803
amicorum	24,975	axilliflora	27,047	chlorosepala	28,627
curvirostris	24,968	gillii	26,939	pauciovulata	28,617
quadrangularis	24,897	granulatifolia	26,939	carnea	28,530
carnea	24,684	langebergensis	26,939	oxycoccifolia	28,516
conferta	24,684	melanthera	26,939	racemosa	28,405
oxycoccifolia	24,670	mundii	26,939	velatiflora	28,367
astroites	24,658	pearsoniana	26,939	propendens	28,227
thomensis	24,651	poculiflora	26,939	rehmii	28,194
abbottii	24,607	rhodella	26,939	bakeri	28,176
ampullacea	24,559	heleophila	26,923	elsieana	28,166
brachysepala	24,556	galgebergensis	26,916	consobrina	28,159
shannonii	24,402	sexfaria	26,867	viscosissima	28,158
rehmii	24,347	karooica	26,864	comorensis subsp. anjurensis	28,128
consobrina	24,313	atherstonei	26,844	filamentosa	28,102
leucopelta	24,301	trachysantha	26,844	brevifolia	28,084
mauritanica	24,242	deflexa	26,770	aghillana	28,049
esteriana	24,167	petiolaris	26,755	heleogena	27,996
esteriana subsp. esteriana	24,167	tradouwensis	26,577	dysantha	27,967
esteriana subsp. swartbergensis	24,167	chiroptera	26,483	simii	27,878
heleogena	24,150	multiflexuosa	26,483	setosa	27,873
oreophila	24,098	costatisepala	26,374	leptopus	27,870
simii	24,032	krugeri	26,328	leucantha	27,870
leptopus	24,024	baueri	26,256	lutea	27,870
leucantha	24,024	baueri subsp. baueri	26,256	margaritacea	27,846
lutea	24,024	cryptanthera	26,252	inamoena	27,794
margaritacea	24,000	parviporandra	26,239	andreaei	27,681
cetrata	23,968	urceolata	26,239	benthamiana	27,643
guthriei	23,871	nematophylla	26,236	wyliei	27,641
hermani	23,871	thunbergii	26,204	seriphiifolia	27,617
polifolia	23,871	imbricata	26,203	penicilliformis	27,518
micrandra	23,843	nutans	26,203	klotzschii	27,507
capitata	23,826	vestita	26,201	setacea	27,499
wyliei	23,795	crassisepala	26,166	densifolia	27,418
leucanthera	23,776	coarctata	26,075	trichophora	27,372
totta	23,776	albescens	25,893	plukenetii subsp. breviflora	27,345
irbyana	23,747	oxyandra	25,893	spumosa	27,307
georgica	23,642	rudolfii	25,893	zitzikammensis	27,298
spumosa	23,460	turneri	25,893	umbratica	27,168
umbratica	23,322	carduifolia	25,887	dodii	27,116
mammosa	23,215	intricata	25,887	mammosa	27,061
nudiflora	23,195	eriocodon	25,870	axilliflora	27,047
trichostigma	23,109	limosa	25,795	gillii	26,939
involucrata	23,035	salteri	25,795	granulatifolia	26,939
lageniformis	23,009	gnaphaloides	25,744	langebergensis	26,939
condensata	22,965	madida	25,709	melanthera	26,939
elimensis	22,940	diotiflora	25,689	mundii	26,939
macrotrema	22,940	glandulosa	25,647	pearsoniana	26,939
nemorosa	22,940	glandulosa subsp. glandulosa	25,647	poculiflora	26,939
ocellata	22,940	navigatoris	25,645	rhodella	26,939

Erica sp. Vlok2988

Region and flowering month excluded		Region included and flowering month excluded		All characters included	
**Species**	**Probability**	**Species**	**Probability**	**Species**	**Probability**
binaria	149,015	binaria	149,015	binaria	149,015
atricha	134,005	atricha	134,005	heterophylla	136,833
heterophylla	131,807	heterophylla	131,807	atricha	134,005
densifolia	129,894	densifolia	129,894	thomae	131,753
mammosa	129,345	mammosa	129,345	densifolia	129,894
thomae	126,728	thomae	126,728	etheliae	129,521
etheliae	124,496	etheliae	124,496	mammosa	129,345
viscaria subsp. longifolia	122,342	viscaria subsp. longifolia	122,342	viscaria subsp. longifolia	127,367
x serratifolia	119,702	x serratifolia	119,702	x serratifolia	119,702
comorensis	46,767	cetrata	47,891	cetrata	47,891
comorensis subsp. comorensis	46,767	comorensis	46,767	woodii	47,881
equisetifolia	44,689	comorensis subsp. comorensis	46,767	comorensis	46,767
woodii	42,856	equisetifolia	44,689	comorensis subsp. comorensis	46,767
comorensis subsp. anjurensis	42,790	areolata	44,291	equisetifolia	44,689
artemisioides	42,080	parviporandra	44,291	areolata	44,291
platycalyx	41,810	terniflora	44,291	parviporandra	44,291
xeranthemifolia	41,504	woodii	42,856	terniflora	44,291
rakotozafyana	40,274	comorensis subsp. anjurensis	42,790	bredasiana	42,929
remota	40,274	melanomontana	42,670	comorensis subsp. anjurensis	42,790
filiformis	39,702	artemisioides	42,080	melanomontana	42,670
cetrata	39,488	blaerioides	41,826	artemisioides	42,080
paucifolia	39,134	blaerioides subsp. hirsuta	41,826	blaerioides	41,826
paucifolia subsp. ciliata	39,134	platycalyx	41,810	blaerioides subsp. hirsuta	41,826
paucifolia subsp. paucifolia	39,134	xeranthemifolia	41,504	platycalyx	41,810
paucifolia subsp. squarrosa	39,134	rakotozafyana	40,274	xeranthemifolia	41,504
subcapitata	38,653	remota	40,274	rakotozafyana	40,274
thomensis	38,472	filiformis	39,702	remota	40,274
bredasiana	37,904	paucifolia	39,134	filiformis	39,702
arachnocalyx	37,118	paucifolia subsp. ciliata	39,134	paucifolia	39,134
cereris	37,118	paucifolia subsp. paucifolia	39,134	paucifolia subsp. ciliata	39,134
areolata	35,888	paucifolia subsp. squarrosa	39,134	paucifolia subsp. paucifolia	39,134
parviporandra	35,888	subcapitata	38,653	paucifolia subsp. squarrosa	39,134
terniflora	35,888	thomensis	38,472	subcapitata	38,653
pleiotricha	35,611	bredasiana	37,904	thomensis	38,472
pleiotricha var. blaerioides	35,611	arachnocalyx	37,118	petraea	37,648
tragomontana	35,611	cereris	37,118	plukenetii	37,183
pilosiflora	35,316	pleiotricha	35,611	arachnocalyx	37,118
pilosiflora subsp. pilosiflora	35,316	pleiotricha var. blaerioides	35,611	cereris	37,118
pilosiflora subsp. purpurea	35,316	tragomontana	35,611	anguliger	36,624
benguelensis	34,811	pilosiflora	35,316	phaeocarpa	36,624
benguelensis var. albescens	34,811	pilosiflora subsp. pilosiflora	35,316	imbricata	35,846
benguelensis var. benguelensis	34,811	pilosiflora subsp. purpurea	35,316	albens	35,845
hexandra	34,811	umbelliflora	34,992	pleiotricha	35,611
outeniquae	34,383	benguelensis	34,811	pleiotricha var. blaerioides	35,611
melanomontana	34,267	benguelensis var. albescens	34,811	tragomontana	35,611
rossii	34,222	benguelensis var. benguelensis	34,811	plukenetii subsp. plukenetii	35,381
blaerioides	33,422	hexandra	34,811	pilosiflora	35,316
blaerioides subsp. hirsuta	33,422	outeniquae	34,383	pilosiflora subsp. pilosiflora	35,316
mafiensis	32,917	rossii	34,222	pilosiflora subsp. purpurea	35,316
bosseri	32,441	mafiensis	32,917	coarctata	35,230
humbertii	32,441	paniculata	32,623	umbelliflora	34,992
madagascariensis	32,441	petraea	32,623	benguelensis	34,811
capillaris	31,849	bosseri	32,441	benguelensis var. albescens	34,811
coarctata	30,205	humbertii	32,441	benguelensis var. benguelensis	34,811
inconstans	29,778	madagascariensis	32,441	hexandra	34,811
daphniflora	29,198	humifusa	32,440	outeniquae	34,383
alfredii	28,713	plukenetii	32,158	rossii	34,222
elimensis	28,697	pseudocalycina	31,881	mafiensis	32,917
leucantha	28,154	capillaris	31,849	paniculata	32,623
rhopalantha	28,154	anguliger	31,599	bosseri	32,441
leucanthera	27,076	phaeocarpa	31,599	humbertii	32,441
borboniifolia	27,069	imbricata	30,821	madagascariensis	32,441
dissimulans	26,589	sonderiana	30,821	humifusa	32,440
mundii	26,589	albens	30,819	cristiflora	32,205
oliveri	26,589	georgica	30,819	flaccida	32,185
umbelliflora	26,589	inamoena	30,606	pseudocalycina	31,881
wittebergensis	26,589	esteriana subsp. swartbergensis	30,427	capillaris	31,849
turbiniflora	26,509	adelopetala	30,356	dissimulans	31,614
caespitosa	25,359	plukenetii subsp. plukenetii	30,356	turbiniflora	31,534
azorica	25,270	coarctata	30,205	sonderiana	30,821
chiroptera	25,270	inconstans	29,778	georgica	30,819
scoparia	25,270	astroites	29,304	viridiflora	30,690
oreophila	25,001	andreaei	29,281	arcuata	30,660
campanularis	24,968	daphniflora	29,198	inamoena	30,606
azaleifolia	24,787	hispiduloides	28,986	esteriana subsp. swartbergensis	30,427
placentiflora	24,787	benthamiana	28,983	nudiflora	30,403
rubiginosa	24,787	carduifolia	28,927	adelopetala	30,356
glumiflora	24,514	rubens	28,745	hispidula	30,189
opulenta	24,514	alfredii	28,713	karooica	29,835
chartacea	24,219	elimensis	28,697	inconstans	29,778
desmantha	24,219	leucantha	28,154	astroites	29,304
hexensis	24,219	rhopalantha	28,154	andreaei	29,281
jacksoniana	24,219	glandulipila	27,937	daphniflora	29,198
lehmannii	24,219	phacelanthera	27,756	calcicola	29,065
magnisylvae	24,219	cristiflora	27,180	hispiduloides	28,986
paniculata	24,219	oresbia	27,180	benthamiana	28,983
petraea	24,219	flaccida	27,160	carduifolia	28,927
calcicola	24,040	leucanthera	27,076	versicolor	28,888
didymocarpa	24,040	borboniifolia	27,069	plukenetii subsp. lineata	28,780
trimera	24,040	nubigena	26,866	plukenetii subsp. penicillata	28,780
trimera subsp. trimera	24,040	dissimulans	26,589	rubens	28,745
tristis	24,040	mundii	26,589	alfredii	28,713
chonantha	24,037	oliveri	26,589	elimensis	28,697
humifusa	24,037	wittebergensis	26,589	orientalis	28,444
multiflexuosa	24,037	costatisepala	26,548	subulata	28,220
omninoglabra	24,037	toringbergensis	26,531	leucantha	28,154
polifolia	24,037	turbiniflora	26,509	rhopalantha	28,154
plukenetii	23,755	viridiflora	25,665	glandulipila	27,937
plukenetii subsp. lineata	23,755	arcuata	25,635	concordia	27,819
plukenetii subsp. penicillata	23,755	seriphiifolia	25,535	diaphana	27,793
alticola	23,714	montis-hominis	25,378	phacelanthera	27,756
aghillana	23,554	nudiflora	25,378	lepidota	27,443
pseudocalycina	23,477	caespitosa	25,359	penicilliformis	27,443
sexfaria	23,468	azorica	25,270	curviflora	27,243
curtophylla	23,419	chiroptera	25,270	pearsoniana	27,228
orientalis	23,419	scoparia	25,270	oresbia	27,180
nemorosa	23,254	hispidula	25,164	thodei	27,170
amphigena	23,195	melanthera	25,164	erigena	27,135
anguliger	23,195	kougabergensis	25,105	leucanthera	27,076
arborea	23,195	rosacea	25,105	borboniifolia	27,069
phaeocarpa	23,195	rosacea subsp. glabrata	25,105	nubigena	26,866
subulata	23,195	rosacea subsp. rosacea	25,105	coccinea	26,764
pauciovulata	23,166	oreophila	25,001	simii	26,695
pulchella	22,893	campanularis	24,968	zeyheriana	26,695
diotiflora	22,771	bolusanthus	24,810	recta	26,665
curvirostris	22,712	hebdomadalis	24,810	mundii	26,589
lucida	22,712	karooica	24,810	oliveri	26,589
thunbergii	22,522	viridiflora subsp. redacta	24,810	wittebergensis	26,589
arenaria	22,498	vlokii	24,810	subdivaricata	26,576
ebracteata	22,417	azaleifolia	24,787	costatisepala	26,548
imbricata	22,417	placentiflora	24,787	toringbergensis	26,531
lepidota	22,417	rubiginosa	24,787	viridiflora subsp. primulina	26,518
penicilliformis	22,417	glumiflora	24,514	uberiflora	26,204
sonderiana	22,417	opulenta	24,514	schelpeorum	26,174
albens	22,416	chartacea	24,219	umbratica	25,744
georgica	22,416	desmantha	24,219	revoluta	25,583
sylvainiana	22,238	hexensis	24,219	seriphiifolia	25,535
trimera subsp. elgonensis	22,238	jacksoniana	24,219	montis-hominis	25,378
blancheana	22,203	lehmannii	24,219	lutea	25,367
coronanthera	22,203	magnisylvae	24,219	caespitosa	25,359
galantha	22,203	calcicola	24,040	azorica	25,270
granulatifolia	22,203	didymocarpa	24,040	chiroptera	25,270
inamoena	22,203	trimera	24,040	scoparia	25,270
pearsoniana	22,203	trimera subsp. trimera	24,040	grandiflora	25,227
pogonanthera	22,203	tristis	24,040	depressa	25,185
tenuipes	22,203	chonantha	24,037	melanthera	25,164
villosa	22,203	multiflexuosa	24,037	atherstonei	25,154
macroloma	22,145	omninoglabra	24,037	kougabergensis	25,105
salax	22,145	polifolia	24,037	rosacea	25,105
strigosa	22,145	navigatoris	23,872	rosacea subsp. glabrata	25,105
thodei	22,145	versicolor	23,863	rosacea subsp. rosacea	25,105
utriculosa	22,145	plukenetii subsp. lineata	23,755	axillaris	25,075
erigena	22,110	plukenetii subsp. penicillata	23,755	oreophila	25,001
esteriana subsp. swartbergensis	22,024	alticola	23,714	campanularis	24,968
goudotiana	22,024	aghillana	23,554	bolusanthus	24,810
tenuicaulis	22,021	loganii	23,484	hebdomadalis	24,810
tetragona	22,004	sexfaria	23,468	viridiflora subsp. redacta	24,810
adelopetala	21,953	curtophylla	23,419	vlokii	24,810
berzelioides	21,953	orientalis	23,419	azaleifolia	24,787
plukenetii subsp. plukenetii	21,953	ostiaria	23,362	placentiflora	24,787
coccinea	21,739	mira	23,303	rubiginosa	24,787

gracilipes	20,470	thimifolia	23,035	crateriformis	24,389	newdigateae	22,661	passerina	23,627	lowryensis	25,378	villosa	23,183	latifolia	25,081	thomae	26,379	denticulata	22,939	physantha	25,548	heleophila	26,923	simii	21,670	nemorosa	23,254	glumiflora	24,514
albescens	20,467	stylaris	22,994	diosmifolia	24,389	longiaristata	22,487	sagittata	23,567	acuta	25,309	axillaris	23,152	saptouensis	25,081	affinis	26,312	lananthera	22,939	adunca	25,473	galgebergensis	26,916	zeyheriana	21,670	amphigena	23,195	opulenta	24,514
argyraea	20,467	recurvifolia	22,960	emarginata	24,389	subdivaricata	22,487	parviporandra	23,562	capillaris	25,222	cetrata	23,112	oligantha	25,043	rubiginosa	26,307	remota	22,930	chartacea	25,473	sexfaria	26,867	oxysepala	21,574	arborea	23,195	discolor	24,444
aspalathoides	20,467	inamoena	22,891	glumiflora	24,389	depressa	22,330	laevigata	23,507	tenuifolia	25,159	intermedia	23,096	cryptanthera	25,027	capillaris	26,262	anomala	22,865	lasciva	25,473	karooica	26,864	subdivaricata	21,550	subulata	23,195	chartacea	24,219
chionodes	20,467	amphigena	22,817	keeromsbergensis	24,389	nivea	22,330	albens	23,492	limosa	25,118	intermedia subsp. intermedia	23,096	myriocodon	25,027	intermedia	26,164	silvatica	22,770	cederbergensis	25,416	trachysantha	26,844	irbyana	21,471	pauciovulata	23,166	desmantha	24,219
dysantha	20,467	subulata	22,817	lachnaeifolia	24,389	labialis	22,232	heleophila	23,492	ardens	25,065	melastoma	23,096	placentiflora	25,027	intermedia subsp. intermedia	26,164	infundibuliformis	22,758	fausta	25,416	lananthera	26,785	orculiflora	21,393	zwartbergensis	23,089	hexensis	24,219
hirta	20,467	cymosa subsp. cymosa	22,768	pudens	24,389	capillaris	22,155	cerinthoides	23,413	urceolata	25,058	melastoma subsp. melastoma	23,096	regerminans	25,000	paludicola	26,152	truncata	22,692	trichadenia	25,416	petiolaris	26,755	brachysepala	21,322	pulchella	22,893	jacksoniana	24,219
lerouxiae	20,467	sagittata	22,650	sperata	24,389	tenuifolia	22,092	cerinthoides var. cerinthoides	23,413	mallotocalyx	25,005	melastoma subsp. minor	23,096	montis-hominis	24,993	pulvinata	26,151	algida	22,676	muscosa	25,415	tradouwensis	26,577	viscaria	21,307	concordia	22,794	lehmannii	24,219
marifolia	20,467	tetrathecoides	22,639	thodei	24,389	quadrangularis	22,026	umbellata	23,332	tegulifolia	24,848	paludicola	23,085	coccinea	24,974	calcareophila	26,098	whyteana	22,652	revoluta	25,411	intermedia subsp. albiflora	26,351	rusticula	21,179	kammanassieae	22,794	magnisylvae	24,219
oxyandra	20,467	blaerioides subsp. blaerioides	22,635	navigatoris	24,367	blaerioides subsp. blaerioides	21,982	pauciovulata	23,316	trichroma	24,848	coacervata	22,968	coccinea subsp. uniflora	24,974	jacksoniana	26,048	empetrina	22,646	simulans	25,397	krugeri	26,328	uberiflora	21,179	diotiflora	22,771	tenella	24,206
pubescens	20,467	plukenetii subsp. plukenetii	22,613	serrata	24,319	eremioides subsp. pubescens	21,982	orculiflora	23,275	adunca	24,815	gillii	22,968	pseudocalycina	24,907	paniculata	26,048	lucida	22,511	regerminans	25,315	baueri	26,256	gnaphaloides	21,092	diaphana	22,768	didymocarpa	24,040
rudolfii	20,467	discolor	22,586	cruenta	24,136	anguliger	21,961	glomiflora	23,263	plukenetii	24,815	schelpeorum	22,967	nemorosa	24,899	coacervata	26,036	barnettiana	22,510	azorica	25,262	baueri subsp. baueri	26,256	tegulifolia	21,092	atromontana	22,735	trimera	24,040
tradouwensis	20,467	discolor variant B 'speciosa'	22,586	tenuicaulis	24,129	bracteolaris	21,935	ixanthera	23,263	puberuliflora	24,792	abietina subsp. constantiana	22,839	nervata	24,851	macrotrema	26,002	pycnantha	22,509	scoparia	25,262	cryptanthera	26,252	wangfatiana	21,005	jananthus	22,735	trimera subsp. trimera	24,040
nutans	20,433	lavandulifolia	22,537	brachycentra	24,101	cinerea	21,935	leucodesmia	23,263	oxysepala	24,742	leucanthera	22,809	stokoei	24,811	melanthera	26,002	intermedia subsp. albiflora	22,505	sylvainiana	25,160	parviporandra	26,239	astroites	20,901	curvirostris	22,712	tristis	24,040
zwartbergensis	20,433	macrotrema	22,537	juniperina	24,101	greyi	21,878	pulchella	23,247	toringbergensis	24,698	alfredii	22,795	esteriana	24,772	campanularis	25,999	corydalis	22,450	flaccida	25,110	glomiflora	26,230	latifolia	20,884	lucida	22,712	chonantha	24,037
goatcheriana	20,311	maritima	22,537	arachnocalyx	24,046	pubigera	21,878	blaerioides	23,218	sessiliflora	24,686	multumbellifera	22,776	esteriana subsp. esteriana	24,772	pauciovulata	25,999	turbiniflora	22,379	bicolor	25,042	turbiniflora	26,225	silvatica	20,884	thunbergii	22,522	multiflexuosa	24,037
sphaerocephala	20,311	pilaarkopensis	22,537	petrophila	24,046	globulifera	21,862	blaerioides subsp. hirsuta	23,218	thomae	24,641	oreophila	22,776	oblongiflora	24,720	alfredii	25,862	sagittata	22,354	wittebergensis	24,997	thunbergii	26,204	andreaei	20,877	arenaria	22,498	omninoglabra	24,037
cymosa subsp. grandiflora	20,275	boucheri	22,528	melastoma	24,034	tradouwensis	21,827	oreotragus	23,218	chamissonis	24,633	oresigena	22,776	thomensis	24,651	multumbellifera	25,844	multumbellifera	22,297	curvirostris	24,968	nutans	26,203	condensata	20,797	ebracteata	22,417	polifolia	24,037
formosa	20,275	rigidula	22,528	melastoma subsp. melastoma	24,034	rusticula	21,779	pubescens	23,218	daphniflora	24,633	plumigera	22,776	coronanthera	24,574	pyxidiflora	25,843	viscaria subsp. macrosepala	22,275	chlorosepala	24,781	crassisepala	26,166	imerinensis	20,723	lepidota	22,417	whyteana	23,995
leucodesmia	20,275	anomala	22,462	vallis-fluminis	23,968	oxysepala	21,675	tegetiformis	23,218	truncata	24,588	tegulifolia	22,776	priorii	24,540	hendricksei	25,823	corifolia	22,222	outeniquae	24,734	coarctata	26,075	bracteolaris	20,614	penicilliformis	22,417	navigatoris	23,872
aestiva	20,251	desmantha	22,462	curviflora	23,953	turneri	21,646	goatcheriana var. goatcheriana	23,203	xeranthemifolia	24,553	hendricksei	22,756	inamoena	24,430	lignosa	25,820	ixanthera	22,222	quadrifida	24,694	corifolia	26,068	hispiduloides	20,582	oliveranthus	22,251	natalitia	23,802
gibbosa	20,251	hebdomadalis	22,462	unicolor	23,953	toringbergensis	21,631	wittebergensis	23,197	marifolia	24,484	lignosa	22,752	roseoloba	24,382	oraria	25,790	cordata	22,202	carnea	24,684	viridiflora subsp. redacta	26,048	benthamiana	20,580	sylvainiana	22,238	umbellata	23,802
racemosa	20,251	magnisylvae	22,462	unicolor subsp. unicolor	23,953	bolusiae	21,573	cordata	23,138	stylaris	24,355	eremioides subsp. eglandula	22,692	revoluta	24,358	longiaristata	25,736	hispidula	22,202	oxycoccifolia	24,670	canescens	25,996	revoluta	20,558	trimera subsp. elgonensis	22,238	alticola	23,714
scabriuscula	20,251	densifolia	22,375	phacelanthera	23,919	pannosa	21,573	karooica	23,138	rehmii	24,344	distorta	22,544	scytophylla	24,343	distorta	25,612	macrophylla	22,202	astroites	24,658	rudolfii	25,893	carduifolia	20,524	curviflora	22,218	corifolia	23,565
kingaensis	20,119	altiphila	22,335	kingaensis subsp. rugegensis	23,886	setociliata	21,573	macrophylla	23,138	andevalensis	24,319	zebrensis	22,537	papyracea	24,328	zebrensis	25,604	viridiflora subsp. redacta	22,202	thomensis	24,651	turneri	25,893	polycoma	20,524	blancheana	22,203	aghillana	23,554
terniflora	20,071	esteriana subsp. swartbergensis	22,191	arcuata	23,812	thomae	21,573	amicorum	23,065	carnea	24,299	depressa	22,511	excavata	24,290	depressa	25,578	canescens	22,150	abbottii	24,607	carduifolia	25,887	sphaerocephala	20,524	coronanthera	22,203	pannosa	23,509
cristiflora	20,005	cetrata	22,103	coccinea subsp. uniflora	23,671	chamissonis	21,566	intricata	23,015	barrydalensis	24,030	nivea	22,511	melanacme	24,184	oophylla	25,567	alfredii	22,126	ampullacea	24,559	intricata	25,887	velitaris	20,500	galantha	22,203	loganii	23,484
dissimulans	20,005	puberuliflora	22,041	uberiflora	23,671	gnaphaloides	21,445	tarantulae	23,015	curvirostris	23,902	atropurpurea	22,499	irregularis	24,160	versicolor	25,492	inconstans	22,092	racemosa	24,559	eriocodon	25,870	nyassana	20,450	granulatifolia	22,203	sexfaria	23,468
hexensis	20,005	benthamiana	22,034	versicolor	23,671	argyraea	21,416	amoena	22,943	brachysepala	23,898	oophylla	22,499	tenella	24,160	globiceps	25,445	caprina	22,086	brachysepala	24,556	limosa	25,795	junonia	20,447	pearsoniana	22,203	patersonii	23,440
recurvata	19,983	glandulosa	22,021	villosa	23,565	aspalathoides	21,416	haematocodon	22,943	chonantha	23,882	patens	22,499	curvirostris	24,083	similis	25,445	tetrathecoides	22,067	velatiflora	24,521	gnaphaloides	25,744	junonia var. minor	20,447	pogonanthera	22,203	curtophylla	23,419
cordata	19,969	glandulosa subsp. glandulosa	22,021	seriphiifolia	23,542	cedromontana	21,416	mauritanica	22,943	discolor	23,873	holosericea	22,496	albertyniae	24,023	tubercularis	25,387	tetragona	21,997	macilenta	24,466	madida	25,709	marlothii	20,401	tenuipes	22,203	ostiaria	23,362
imbricata	19,969	banksii	22,017	marifolia	23,479	chionodes	21,416	bicolor	22,786	discolor variant B 'speciosa'	23,873	comorensis subsp. anjurensis	22,476	spumosa	23,940	vogelpoelii	25,303	papyracea	21,989	obconica	24,407	diotiflora	25,689	suffulta	20,401	villosa	22,203	parviflora	23,352
lowryensis	19,969	sessiliflora	21,936	pubescens	23,479	hirta	21,416	holosericea	22,786	multumbellifera	23,873	chrysocodon	22,472	varderi	23,932	cymosa subsp. cymosa	25,270	montis-hominis	21,959	umbelliflora	24,407	navigatoris	25,645	carnea	20,376	macroloma	22,145	coccinea subsp. coccinea	23,317
macrophylla	19,969	extrusa	21,932	madida	23,476	lerouxiae	21,416	lucida	22,786	pearsoniana	23,748	berzelioides	22,425	tegetiformis	23,928	rusticula	25,268	bracteolaris	21,840	shannonii	24,402	stokoei	25,623	brachycentra	20,343	salax	22,145	leucotrachela	23,317
natalitia	19,969	nigrimontana	21,932	chlorosepala	23,475	marifolia	21,416	whyteana	22,747	lehmannii	23,727	ciliaris	22,425	brevifolia	23,924	agglutinans	25,253	stokoei	21,777	rehmii	24,347	serrata	25,556	setacea	20,343	strigosa	22,145	leucotrachela subsp. leucotrachela	23,317
ostiaria	19,969	wildii	21,932	ardens	23,422	nivalis	21,416	campanularis	22,661	densifolia	23,663	versicolor	22,425	truncata	23,924	vernicosa	25,253	serrata	21,710	consobrina	24,313	physantha	25,548	erasmia	20,341	thodei	22,145	vestita	23,317
simulans	19,969	albens	21,903	roseoloba	23,373	amalophylla	21,291	newdigateae	22,661	passerina	23,627	filamentosa	22,370	monadelphia	23,878	adunca	25,147	austronyassana	21,701	viscosissima	24,312	chartacea	25,473	leptopus	20,341	utriculosa	22,145	mira	23,303
viridiflora subsp. redacta	19,969	klotzschii	21,897	goatcheriana	23,323	philippioides	21,291	longiaristata	22,487	sagittata	23,567	obconica	22,368	propendens	23,871	hebdomadalis	25,147	intermedia	21,693	leucopelta	24,301	lasciva	25,473	lutea	20,341	erigena	22,110	pumila	23,283
baroniana	19,770	harveiana	21,895	sphaerocephala	23,323	cunoniensis	21,276	subdivaricata	22,487	parviporandra	23,562	intricata	22,352	acuta	23,808	latifolia	25,081	intermedia subsp. intermedia	21,693	puberuliflora	24,274	fausta	25,416	rubens	20,341	goudotiana	22,024	nemorosa	23,254
ingeana	19,770	magistrati	21,895	cymosa subsp. grandiflora	23,287	rehmii	21,276	quadrifida	22,438	albens	23,492	tubercularis	22,319	passerina	23,756	saptouensis	25,081	junonia	21,672	pubigera	24,272	rhopalantha	25,413	vogelpoelii	20,341	tenuicaulis	22,021	amphigena	23,195
kammanassieae	19,728	oakesiorum	21,895	aestiva	23,263	carnea	21,231	depressa	22,330	heleophila	23,492	fimbriata	22,307	marlothii	23,652	oligantha	25,043	junonia var. minor	21,672	mauritanica	24,242	rubens	25,413	microdonta	20,320	tetragona	22,004	arborea	23,195
pulvinata	19,710	rivularis	21,895	scabriuscula	23,263	jacksoniana	21,089	nivea	22,330	umbellata	23,332	saxicola	22,307	involucrata	23,613	cryptanthera	25,027	ioniana	21,644	esteriana	24,167	vogelpoelii	25,413	columnaris	20,317	monsoniana	21,969	pauciovulata	23,166
axillaris	19,686	tragulifera	21,895	ocellata	23,134	trichophora	21,016	labialis	22,232	pauciovulata	23,316	dolfiana	22,283	dispar	23,608	myriocodon	25,027	bredasiana	21,635	esteriana subsp. esteriana	24,167	simulans	25,397	mauritiensis	20,220	berzelioides	21,953	zwartbergensis	23,089
monsoniana	19,681	umbratica	21,895	coronanthera	23,035	galgebergensis	20,939	capillaris	22,155	glandulosa	23,308	setociliata	22,283	plukenetii subsp. bredensis	23,566	placentiflora	25,027	tristis	21,635	esteriana subsp. swartbergensis	24,167	coccinea subsp. uniflora	25,347	depressa	20,160	discolor variant B 'speciosa'	21,788	barrydalensis	22,980
gigantea	19,661	heleophila	21,850	patens	23,035	condensata	20,859	pycnantha	22,152	glandulosa subsp. glandulosa	23,308	mauritanica	22,280	plukenetii subsp. penicillata	23,566	regerminans	25,000	erasmia	21,567	heleogena	24,150	regerminans	25,315	micrandra	20,160	coccinea	21,739	passerinoides	22,894
pyxidiflora	19,661	intermedia subsp. albiflora	21,834	thimifolia	23,035	brachysepala	20,830	tenuifolia	22,092	racemosa	23,295	klotzschii	22,272	boucheri	23,536	montis-hominis	24,993	leptantha	21,567	oreophila	24,098	viscaria subsp. pustulata	25,285	nivea	20,160	leucopelta	21,699	pulchella	22,893
bredasiana	19,660	leucanthera	21,801	dissimulans	23,017	chonantha	20,814	gigantea	22,079	leucodesmia	23,263	binaria	22,241	rigidula	23,536	melanomontana	24,983	rhopalantha	21,567	afra	24,091	azorica	25,262	atherstonei	20,128	simii	21,670	kammanassieae	22,794
caespitosa	19,660	plumosa	21,799	stylaris	22,994	atropurpurea	20,761	outeniquae	22,057	blaerioides	23,218	vogelpoelii	22,235	terniflora	23,536	doliiformis	24,951	rubens	21,567	simii	24,032	scoparia	25,262	fausta	20,128	zeyheriana	21,670	diotiflora	22,771
calcareophila	19,657	quadrangularis	21,720	natalitia	22,981	coronanthera	20,761	urceolata	21,990	blaerioides subsp. hirsuta	23,218	bojeri	22,168	banksii subsp. purpurea	23,512	nemorosa	24,899	vogelpoelii	21,567	leptopus	24,024	sylvainiana	25,160	oakesiorum	20,128	recta	21,640	atromontana	22,735
vallis-gratiae	19,657	cubitans	21,657	simulans	22,981	oophylla	20,761	blaerioides subsp. blaerioides	21,982	goatcheriana var. goatcheriana	23,203	parviporandra	22,108	baueri	23,484	nervata	24,851	columnaris	21,543	leucantha	24,024	bicolor	25,042	oophylla	20,128	oxysepala	21,574	jananthus	22,735
ioniana	19,581	omninoglabra	21,597	viridiflora subsp. redacta	22,981	planifolia	20,761	eremioides subsp. pubescens	21,982	wittebergensis	23,197	adunca	22,080	baueri subsp. baueri	23,484	esteriana	24,772	occulta	21,543	lutea	24,024	wittebergensis	24,997	oxyandra	20,128	esteriana	21,564	curvirostris	22,712
gillii	19,502	daphniflora	21,544	inamoena	22,891	taylorii	20,761	cinerea	21,935	cordata	23,138	chartacea	22,080	anomala	23,471	esteriana subsp. esteriana	24,772	virginalis	21,543	margaritacea	24,000	retorta	24,983	podophylla	20,128	madida	21,564	lucida	22,712
comorensis	19,493	glandulipila	21,491	discolor variant A 'discolor'	22,882	atromontana	20,591	elsieana	21,908	karooica	23,138	desmantha	22,080	crateriformis	23,471	thomensis	24,651	viscaria subsp. pustulata	21,439	cetrata	23,968	curvirostris	24,968	setosa	20,128	rigidula	21,564	thunbergii	22,522
eriocodon	19,487	infundibuliformis	21,471	amphigena	22,817	cooperi	20,478	greyi	21,878	amicorum	23,065	hebdomadalis	22,080	karooica	23,471	coronanthera	24,574	pyramidalis	21,426	leucodesmia	23,899	pseudocalycina	24,953	trichadenia	20,128	urceolata	21,564	arenaria	22,498
banksii subsp. comptonii	19,485	vestita	21,467	subulata	22,817	leucopelta	20,196	globulifera	21,862	intricata	23,015	lasciva	22,080	magnisylvae	23,471	priorii	24,540	phacelanthera	21,310	miniscula	23,891	versicolor	24,936	axillaris	20,049	subdivaricata	21,550	ebracteata	22,417
adnata	19,438	wyliei	21,414	ingeana	22,782	beatricis	20,183	rusticula	21,779	tarantulae	23,015	arachnocalyx	22,042	aneimena	23,436	intermedia subsp. albiflora	24,519	obtusata	21,294	cubitans	23,890	dracomontana	24,818	trimera subsp. keniensis	19,949	viridiflora subsp. primulina	21,493	banksii	22,286
arenaria	19,438	navigatoris	21,355	axillaris	22,698	montis-hominis	20,159	adunca	21,747	imbricata	23,013	orthiocola	22,042	oliveranthus	23,422	extrusa	24,463	chrysocodon	21,239	guthriei	23,871	procaviana	24,818	perspicua	19,937	irbyana	21,471	banksii subsp. comptonii	22,286
bicolor	19,438	serrata	21,307	caespitosa	22,672	altiphila	20,061	chartacea	21,747	penicilliformis	23,013	petrophila	22,042	abbottii	23,383	lachnaeifolia	24,463	jasminiflora	21,207	hermani	23,871	glabripes	24,785	perspicua subsp. latifolia	19,937	orculiflora	21,393	banksii subsp. purpurea	22,286
copiosa	19,438	loganii	21,306	sagittata	22,650	esteriana	20,061	lasciva	21,747	haematocodon	22,943	muscosa	22,021	altiphila	23,344	inamoena	24,430	cylindrica	21,137	micrandra	23,843	outeniquae	24,734	perspicua subsp. perspicua	19,937	brachysepala	21,322	coccinea subsp. uniflora	22,286
elimensis	19,438	xeranthemifolia	21,226	tetrathecoides	22,639	esteriana subsp. esteriana	20,061	plukenetii	21,747	mauritanica	22,943	sylvainiana	21,965	plumosa	23,338	diaphana	24,397	retorta	21,137	stylaris	23,837	lignosa	24,726	duthieae	19,930	viscaria	21,307	intermedia	22,286
grata	19,438	swaziensis	21,198	macrotrema	22,537	cetrata	19,951	oxysepala	21,675	esterhuyseniae	22,839	melanomontana	21,915	coccinea subsp. coccinea	23,329	phillipsii	24,391	pseudocalycina	21,106	capitata	23,826	fairii	24,710	mauritanica	19,930	rusticula	21,179	intermedia subsp. intermedia	22,286
humansdorpensis	19,438	trichophylla	21,196	maritima	22,537	villosa	19,900	toringbergensis	21,631	canaliculata	22,816	phacelanthera	21,915	empetrina	23,315	umbrosa	24,391	genistifolia	21,063	wyliei	23,795	quadrifida	24,694	petricola	19,868	uberiflora	21,179	plukenetii subsp. bredensis	22,286
keetii	19,438	cooperi	21,131	pilaarkopensis	22,537	brevicaulis	19,873	sessiliflora	21,618	bicolor	22,786	armandiana	21,891	abietina	23,312	scytophylla	24,343	rubiginosa	21,063	leucanthera	23,776	astroites	24,658	viscaria subsp. gallorum	19,792	nervata	21,173	oliveranthus	22,251
onusta	19,438	cruenta	21,124	boucheri	22,528	affinis	19,801	bolusiae	21,573	holosericea	22,786	abietina subsp. diabolis	21,883	thomae	23,312	glomiflora	24,329	dracomontana	20,972	totta	23,776	longimontana	24,655	manipuliflora	19,773	roseoloba	21,173	sylvainiana	22,238
parviflora	19,438	priorii	21,075	rigidula	22,528	esterhuyseniae	19,772	pannosa	21,573	lucida	22,786	doliiformis	21,883	ampullacea	23,258	papyracea	24,328	elsieana	20,972	irbyana	23,747	thomensis	24,651	multumbellifera	19,773	schelpeorum	21,149	trimera subsp. elgonensis	22,238
tenuipes	19,438	umbelliflora	21,067	anomala	22,462	canaliculata	19,748	setociliata	21,573	discolor variant A 'discolor'	22,778	viscaria subsp. longifolia	21,883	esteriana subsp. swartbergensis	23,200	excavata	24,290	procaviana	20,972	burchelliana	23,737	concordia	24,619	vagans	19,773	setulosa	21,149	fairii	22,207
trachysantha	19,438	garciae	21,052	desmantha	22,462	comorensis	19,739	thomae	21,573	infundibuliformis	22,759	kammanassieae	21,803	capillaris	23,195	palliiflora	24,217	angulosa	20,968	penicilliformis	23,672	abbottii	24,607	serrata	19,654	syngenesia	21,115	vallis-aranearum	22,207
krugeri	19,428	arachnocalyx	21,034	hebdomadalis	22,462	nematophylla	19,647	chamissonis	21,566	whyteana	22,747	nubigena	21,681	villosa	23,183	equisetifolia	24,188	glabripes	20,939	rosacea	23,628	ampullacea	24,559	shannonii	19,583	gnaphaloides	21,092	blancheana	22,203
salteri	19,413	petrophila	21,034	magnisylvae	22,462	viscaria subsp. longifolia	19,647	daphniflora	21,566	viscaria subsp. longifolia	22,714	banksii	21,634	cetrata	23,112	permutata	24,188	filamentosa	20,908	rosacea subsp. rosacea	23,628	brachysepala	24,556	austronyassana	19,558	tegulifolia	21,092	coronanthera	22,203
glaphyra	19,375	pogonanthera	21,034	bracteolaris	22,458	viscaria subsp. macrosepala	19,647	glandulipila	21,512	viscaria subsp. macrosepala	22,714	intermedia subsp. albiflora	21,452	intermedia	23,096	melanacme	24,184	varderi	20,898	densifolia	23,572	uberiflora	24,541	glandulipila	19,534	wangfatiana	21,005	galantha	22,203
petraea	19,364	melastoma	21,022	georgica	22,458	vallis-fluminis	19,525	gnaphaloides	21,445	campanularis	22,661	trichophora	21,411	intermedia subsp. intermedia	23,096	irregularis	24,160	brevifolia	20,890	spumosa	23,460	macilenta	24,466	affinis	19,447	latifolia	20,884	granulatifolia	22,203
salax	19,364	melastoma subsp. melastoma	21,022	tenuipes	22,451	capitata	19,489	argyraea	21,416	newdigateae	22,661	anemodes	21,396	paludicola	23,085	curvirostris	24,083	lignosa	20,880	atropurpurea	23,436	obconica	24,407	phacelanthera	19,352	silvatica	20,884	pogonanthera	22,203
aspalathifolia	19,179	melastoma subsp. minor	21,022	salteri	22,425	crassisepala	19,489	aspalathoides	21,416	cruenta	22,533	diosmifolia	21,396	venustiflora	23,077	macrophylla	24,045	bokkeveldia	20,865	oophylla	23,436	umbelliflora	24,407	cabernetea	19,291	condensata	20,797	tenuipes	22,203
eustacei	19,179	curviflora	20,941	macilenta	22,405	aghillana	19,468	cedromontana	21,416	longiaristata	22,487	emarginata	21,396	venustiflora subsp. glandulosa	23,077	pearsoniana	24,045	strigosa	20,865	dispar	23,331	shannonii	24,402	savileae	19,282	imerinensis	20,723	villosa	22,203
glabella	19,124	unicolor	20,941	cerinthoides	22,340	nudiflora	19,468	chionodes	21,416	brachycentra	22,483	extrusa	21,396	venustiflora subsp. venustiflora	23,077	peltata	24,045	fairii	20,864	umbratica	23,322	borboniifolia	24,328	savileae var. grandiflora	19,282	quadrifida	20,719	tubercularis	22,181
glabella subsp. glabella	19,124	unicolor subsp. unicolor	20,941	cerinthoides var. cerinthoides	22,340	propendens	19,444	hirta	21,416	quadrifida	22,438	keeromsbergensis	21,396	coacervata	22,968	viridiflora subsp. redacta	24,045	wangfatiana	20,840	mammosa	23,215	gysbertii	24,328	savileae var. mutica	19,282	tegetiformis	20,719	macroloma	22,145
glabella subsp. laevis	19,124	phacelanthera	20,907	barrydalensis	22,332	thimifolia	19,370	lerouxiae	21,416	coccinea subsp. uniflora	22,358	lachnaeifolia	21,396	gillii	22,968	jasminiflora	24,030	manipuliflora	20,831	nudiflora	23,195	viscaria subsp. pendula	24,320	savileae var. savileae	19,282	umbratica	20,719	salax	22,145
phaeocarpa	19,124	arcuata	20,800	tetragona	22,275	abietina subsp. constantiana	19,214	marifolia	21,416	intermedia	22,358	nigrimontana	21,396	schelpeorum	22,967	albertyniae	24,023	vagans	20,831	syngenesia	23,169	gracilis	24,306	denticulata	19,256	bracteolaris	20,614	strigosa	22,145
silvatica	19,087	unilateralis	20,800	aspalathifolia	22,191	axilliflora	19,214	rosacea	21,372	intermedia subsp. intermedia	22,358	pudens	21,396	abietina subsp. constantiana	22,839	oreotragus	23,946	flavicoma	20,829	trichostigma	23,109	leucopelta	24,301	gerhardii	19,256	dodii	20,607	utriculosa	22,145
russakiana	19,051	inconstans	20,665	esteriana subsp. swartbergensis	22,191	pyramidalis	19,214	rosacea subsp. rosacea	21,372	nivea	22,330	sperata	21,396	leucanthera	22,809	varderi	23,932	loganii	20,829	grata	23,062	regia	24,281	afrorum	19,247	revoluta	20,558	sessiliflora	22,026
greyi	19,005	angulosa	20,659	cetrata	22,103	doliiformis	19,116	amalophylla	21,291	labialis	22,232	thodei	21,396	alfredii	22,795	brevifolia	23,924	pulvinata	20,805	involucrata	23,035	regia subsp. regia	24,281	tenella	19,181	polycoma	20,524	goudotiana	22,024
afra	18,895	coccinea subsp. uniflora	20,659	silvatica	22,099	ingeana	19,116	philippioides	21,291	curviflora	22,229	wildii	21,396	multumbellifera	22,776	truncata	23,924	amphigena	20,790	juniperina	23,016	puberuliflora	24,274	euryphylla	19,067	sphaerocephala	20,524	tenuicaulis	22,021
turneri	18,895	fuscescens	20,659	russakiana	22,063	latiflora	19,116	cunoniensis	21,276	unicolor	22,229	petrusiana	21,380	oreophila	22,776	savileae	23,872	arborea	20,790	lageniformis	23,009	pubigera	24,272	lusitanica	19,024	velitaris	20,500	tetragona	22,004
leucopelta	18,873	uberiflora	20,659	puberuliflora	22,041	oatesii	19,116	rehmii	21,276	unicolor subsp. unicolor	22,229	diaphana	21,330	oresigena	22,776	savileae var. grandiflora	23,872	diosmifolia	20,790	elimensis	22,940	mauritanica	24,242	whyteana	18,970	nyassana	20,450	agglutinans	22,000
bakeri	18,861	versicolor	20,659	benthamiana	22,034	pillansii	19,116	carnea	21,231	doliiformis	22,184	hottentotica	21,324	plumigera	22,776	savileae var. mutica	23,872	subulata	20,790	macrotrema	22,940	esteriana	24,167	glauca	18,826	junonia	20,447	monsoniana	21,969
costatisepala	18,861	miniscula	20,620	unicolor subsp. mutica	22,026	pillansii subsp. fervida	19,116	juniperina	21,217	ingeana	22,184	phillipsii	21,324	tegulifolia	22,776	savileae var. savileae	23,872	concordia	20,773	nemorosa	22,940	esteriana subsp. esteriana	24,167	glauca var. glauca	18,826	junonia var. minor	20,447	berzelioides	21,953
pleiotricha	18,861	obconica	20,602	banksii	22,017	tetralix	19,116	jacksoniana	21,089	pillansii	22,184	umbrosa	21,324	hendricksei	22,756	propendens	23,871	dysantha	20,773	denticulata	22,939	esteriana subsp. swartbergensis	24,167	pycnantha	18,826	marlothii	20,401	baccans	21,920
pleiotricha var. blaerioides	18,861	seriphiifolia	20,530	nigrimontana	21,932	valida	19,116	macilenta	21,035	valida	22,184	atromontana	21,301	lignosa	22,752	conspicua	23,848	uberiflora	20,695	lananthera	22,939	oreophila	24,098	bokkeveldia	18,809	suffulta	20,401	bolusiae	21,888
pleiotricha var. pleiotricha	18,861	gracilipes	20,470	wildii	21,932	parviflora	18,959	barrydalensis	20,963	pycnantha	22,152	eremioides subsp. pubescens	21,263	flavicoma	22,722	infundibuliformis	23,848	sessiliflora	20,683	remota	22,930	afra	24,091	anomala	18,776	carnea	20,376	discolor variant B 'speciosa'	21,788
acuta	18,849	argyraea	20,467	albens	21,903	velutina	18,959	galgebergensis	20,939	gigantea	22,079	glomiflora	21,261	oraria	22,722	unicolor	23,848	gracilipes	20,677	anomala	22,865	tegetiformis	23,973	bombycina	18,776	brachycentra	20,343	canaliculata	21,785
cedromontana	18,846	aspalathoides	20,467	klotzschii	21,897	frigida	18,906	brachysepala	20,830	outeniquae	22,057	mammosa	21,237	eremioides subsp. eglandula	22,692	acuta	23,808	hispiduloides	20,581	silvatica	22,770	cetrata	23,968	calycina	18,776	setacea	20,343	kraussiana	21,785
cryptanthera	18,846	chionodes	20,467	harveiana	21,895	cereris	18,834	chonantha	20,814	blaerioides subsp. blaerioides	21,982	oatesii	21,237	lambertii	22,669	passerina	23,756	roseoloba	20,581	truncata	22,692	axillaris	23,935	cristiflora	18,776	erasmia	20,341	peltata	21,785
myriocodon	18,846	hirta	20,467	magistrati	21,895	cubitans	18,834	discolor	20,806	eremioides subsp. pubescens	21,982	permutata	21,121	fascicularis	22,645	caespitosa	23,736	eburnea	20,577	algida	22,676	leucodesmia	23,899	longistyla	18,776	leptopus	20,341	consobrina	21,727
nivalis	18,846	lerouxiae	20,467	oakesiorum	21,895	euryphylla	18,834	discolor variant B 'speciosa'	20,806	frigida	21,973	equisetifolia	21,120	fascicularis var. fascicularis	22,645	turbiniflora	23,658	longiaristata	20,560	whyteana	22,652	miniscula	23,891	macrotrema	18,776	lutea	20,341	subverticillaris	21,727
ovina	18,846	marifolia	20,467	tragulifera	21,895	leptoclada	18,834	garciae	20,806	versicolor	21,947	caprina	21,119	distorta	22,544	marlothii	23,652	borboniifolia	20,482	empetrina	22,646	cubitans	23,890	mucronata	18,776	vogelpoelii	20,341	leucopelta	21,699
propinqua	18,825	pubescens	20,467	costatisepala	21,873	oligantha	18,834	grata	20,806	cinerea	21,935	thimifolia	21,108	zebrensis	22,537	involucrata	23,613	gerhardii	20,482	lucida	22,511	guthriei	23,871	natalitia	18,776	microdonta	20,320	oxysepala	21,574
esterhuyseniae	18,822	chlorosepala	20,462	heleophila	21,850	artemisioides	18,825	multumbellifera	20,806	elsieana	21,908	adnata	20,977	depressa	22,511	dispar	23,608	gysbertii	20,482	barnettiana	22,510	hermani	23,871	oresbia	18,776	columnaris	20,317	esteriana	21,564
pannosa	18,822	zwartbergensis	20,433	esterhuyseniae	21,834	muscosa	18,825	coronanthera	20,761	greyi	21,878	blancheana	20,977	nivea	22,511	urceolata	23,603	walkeri	20,482	intermedia subsp. albiflora	22,505	micrandra	23,843	umbellata	18,776	mauritiensis	20,220	madida	21,564
andevalensis	18,789	pycnantha	20,388	leucanthera	21,801	rigidula	18,825	planifolia	20,761	globulifera	21,862	lowryensis	20,977	atropurpurea	22,499	boucheri	23,536	viscaria subsp. pendula	20,474	nivalis	22,470	stylaris	23,837	flaccida	18,757	annalis	20,202	rigidula	21,564
binaria	18,775	goatcheriana	20,311	plumosa	21,799	alopecurus	18,676	taylorii	20,761	rusticula	21,779	macrophylla	20,977	oophylla	22,499	rigidula	23,536	benthamiana	20,449	corydalis	22,450	capitata	23,826	racemosa	18,757	grandiflora	20,202	urceolata	21,564
bombycina	18,769	sphaerocephala	20,311	dracomontana	21,751	holtii	18,676	granulatifolia	20,681	chartacea	21,747	mundii	20,977	patens	22,499	banksii subsp. purpurea	23,512	regia	20,435	glomiflora	22,384	leucanthera	23,776	brevifolia	18,722	grandiflora subsp. grandiflora	20,202	irbyana	21,471
radicans	18,769	cymosa subsp. grandiflora	20,275	exleeana	21,751	pectinifolia	18,676	inclusa	20,681	lasciva	21,747	pearsoniana	20,977	holosericea	22,496	baueri	23,484	regia subsp. regia	20,435	turbiniflora	22,379	totta	23,776	plumigera	18,722	depressa	20,160	adunca	21,432
radicans subsp. radicans	18,769	formosa	20,275	procaviana	21,751	strigilifolia	18,676	langebergensis	20,681	pectinifolia	21,744	peltata	20,977	comorensis subsp. anjurensis	22,476	baueri subsp. baueri	23,484	seriphiifolia	20,423	euryphylla	22,374	ferrea	23,768	tenuifolia	18,697	micrandra	20,160	glabella	21,432
glomiflora	18,760	aestiva	20,251	mammosa	21,748	inaequalis	18,134	pearsoniana	20,681	bolusiae	21,573	simulans	20,977	chrysocodon	22,472	anomala	23,471	lowryensis	20,400	sagittata	22,354	laeta	23,768	junonia var. junonia	18,645	nivea	20,160	glabella subsp. laevis	21,432
kirstenii	18,760	gibbosa	20,251	regia	21,748	inflaticalyx	18,134	rhodella	20,681	pannosa	21,573	sparsa	20,977	ciliaris	22,425	crateriformis	23,471	comorensis subsp. comorensis	20,399	viscaria subsp. macrosepala	22,275	tenuifolia	23,768	corifolia	18,540	atherstonei	20,128	lasciva	21,432
dracomontana	18,739	scabriuscula	20,251	regia subsp. regia	21,748	longimontana	18,134	densifolia	20,595	setociliata	21,573	tenuipes	20,977	versicolor	22,425	karooica	23,471	steinbergiana	20,325	corifolia	22,222	irbyana	23,747	ixanthera	18,540	fausta	20,128	vernicosa	21,432
elsieana	18,739	amicorum	20,243	thomae	21,748	bruniades	18,099	atromontana	20,591	glandulipila	21,512	viridiflora subsp. redacta	20,977	globiceps	22,378	magnisylvae	23,471	kammanassieae	20,313	viridiflora subsp. redacta	22,202	burchelliana	23,737	rhodopis	18,540	oakesiorum	20,128	orculiflora	21,393
exleeana	18,739	kingaensis	20,119	bolusanthus	21,745	marlothii	18,099	nutans	20,532	gnaphaloides	21,445	jasminiflora	20,963	similis	22,378	aneimena	23,436	klotzschii	20,313	vallis-fluminis	22,200	rosacea	23,628	filialis	18,484	oophylla	20,128	brachysepala	21,322
procaviana	18,739	terniflora	20,071	quadrangularis	21,720	ostiaria	18,099	cooperi	20,478	argyraea	21,416	lageniformis	20,963	filamentosa	22,370	oliveranthus	23,422	praenitens	20,301	canescens	22,150	rosacea subsp. rosacea	23,628	pannosa	18,484	oxyandra	20,128	viscaria	21,307
beatricis	18,736	dissimulans	20,005	omninoglabra	21,597	suffulta	18,099	miniscula	20,461	aspalathoides	21,416	blaerioides	20,878	obconica	22,368	discolor variant B 'speciosa'	23,418	trichophora	20,178	alfredii	22,126	abietina	23,626	setociliata	18,484	podophylla	20,128	rusticula	21,179
mammosa	18,736	hexensis	20,005	bruniades	21,552	anemodes	17,870	sexfaria	20,300	cedromontana	21,416	blaerioides subsp. hirsuta	20,878	intricata	22,352	abbottii	23,383	magistrati	20,127	similis	22,100	rhodopis	23,611	dulcis	18,473	setosa	20,128	nervata	21,173
regia	18,736	recurvata	19,983	concordia	21,552	ardens	17,870	glandulosa	20,241	chionodes	21,416	oreotragus	20,878	tubercularis	22,319	altiphila	23,344	oakesiorum	20,127	inconstans	22,092	lavandulifolia	23,591	nubigena	18,463	trichadenia	20,128	roseoloba	21,173
regia subsp. regia	18,736	lowryensis	19,969	glandulipila	21,491	corydalis	17,870	glandulosa subsp. glandulosa	20,241	hirta	21,416	haematocodon	20,851	fimbriata	22,307	plumosa	23,338	tegetiformis	20,127	caprina	22,086	peltata	23,591	patersonii	18,415	passerina	20,078	setulosa	21,149
thomae	18,736	natalitia	19,969	plukenetii subsp. bredensis	21,466	crateriformis	17,870	racemosa	20,227	lerouxiae	21,416	bombycina	20,844	dolfiana	22,283	verticillata	23,330	zwartbergensis	20,127	montis-hominis	21,959	areolata	23,582	argyraea	18,327	axillaris	20,049	syngenesia	21,115
bolusanthus	18,733	ostiaria	19,969	plukenetii subsp. penicillata	21,466	cymosa subsp. cymosa	17,870	leucopelta	20,196	rosacea	21,372	radicans	20,844	setociliata	22,283	empetrina	23,315	axillaris	20,089	stokoei	21,777	cincta	23,519	aspalathoides	18,327	trimera subsp. keniensis	19,949	gnaphaloides	21,092
occulta	18,715	simulans	19,969	melanomontana	21,462	diosmifolia	17,870	beatricis	20,183	rosacea subsp. rosacea	21,372	radicans subsp. radicans	20,844	mauritanica	22,280	bergiana	23,288	hillburttii	20,058	serrata	21,710	azaleifolia	23,518	parviflora	18,327	perspicua	19,937	tegulifolia	21,092
virginalis	18,715	viridiflora subsp. redacta	19,969	loganii	21,306	emarginata	17,870	montis-hominis	20,159	amalophylla	21,291	xeranthemifolia	20,844	klotzschii	22,272	globiceps subsp. globiceps	23,270	marojejyensis	20,055	austronyassana	21,701	atropurpurea	23,436	coccinea subsp. coccinea	18,292	perspicua subsp. latifolia	19,937	wangfatiana	21,005
parviporandra	18,642	conferta	19,952	drakensbergensis	21,281	fimbriata	17,870	altiphila	20,061	philippioides	21,291	savileae	20,804	binaria	22,241	ampullacea	23,258	caledonica	20,052	junonia	21,672	oophylla	23,436	leucotrachela	18,292	perspicua subsp. perspicua	19,937	latifolia	20,884
orthiocola	18,577	discolor variant A 'discolor'	19,870	xeranthemifolia	21,226	hispidula	17,870	esteriana	20,061	cunoniensis	21,276	savileae var. grandiflora	20,804	vogelpoelii	22,235	esteriana subsp. swartbergensis	23,200	glauca	20,052	junonia var. minor	21,672	bolusanthus	23,421	leucotrachela subsp. leucotrachela	18,292	duthieae	19,930	silvatica	20,884
bruniades	18,540	baroniana	19,770	swaziensis	21,198	keeromsbergensis	17,870	esteriana subsp. esteriana	20,061	juniperina	21,217	savileae var. mutica	20,804	bojeri	22,168	villosa	23,183	glauca var. glauca	20,052	ardens	21,654	drakensbergensis	23,399	vestita	18,292	mauritanica	19,930	glomiflora	20,807
capitata	18,540	ingeana	19,770	trichophylla	21,196	kirstenii	17,870	cetrata	19,951	inaequalis	21,202	savileae var. savileae	20,804	parviporandra	22,108	cetrata	23,112	perrieri	19,977	ioniana	21,644	dispar	23,331	incarnata	18,258	petricola	19,868	condensata	20,797
concordia	18,540	kammanassieae	19,728	cooperi	21,131	lachnaeifolia	17,870	imbricata	19,945	viscosissima	21,121	aristata	20,780	adunca	22,080	venustiflora	23,077	dodii	19,922	bredasiana	21,635	curviflora	23,292	multiflora	18,258	viscaria subsp. gallorum	19,792	albertyniae	20,770
crassisepala	18,540	pulvinata	19,710	turbiniflora	21,101	lambertii	17,870	penicilliformis	19,945	jacksoniana	21,089	conspicua	20,780	chartacea	22,080	venustiflora subsp. glandulosa	23,077	duthieae	19,922	tristis	21,635	unicolor	23,292	pumila	18,258	manipuliflora	19,773	imerinensis	20,723
marlothii	18,540	axillaris	19,686	priorii	21,075	magistrati	17,870	villosa	19,900	macilenta	21,035	curviflora	20,780	desmantha	22,080	venustiflora subsp. venustiflora	23,077	ferrea	19,922	erasmia	21,567	unicolor subsp. mutica	23,292	brevicaulis	18,144	multumbellifera	19,773	quadrifida	20,719
petiolaris	18,540	monsoniana	19,681	umbelliflora	21,067	oakesiorum	17,870	brevicaulis	19,873	chlorosepala	20,961	infundibuliformis	20,780	hebdomadalis	22,080	gillii	22,968	laeta	19,922	leptantha	21,567	nudiflora	23,195	costatisepala	18,144	vagans	19,773	tegetiformis	20,719
suffulta	18,540	pyxidiflora	19,661	garciae	21,052	odorata	17,870	affinis	19,801	galgebergensis	20,939	unicolor	20,780	arachnocalyx	22,042	schelpeorum	22,967	pilulifera	19,922	rhopalantha	21,567	syngenesia	23,169	cyathiformis	18,128	maximilianii	19,772	intermedia subsp. albiflora	20,642
plukenetii	18,454	bredasiana	19,660	pogonanthera	21,034	recurvifolia	17,870	esterhuyseniae	19,772	lachnaeifolia	20,938	benguelensis	20,778	orthiocola	22,042	subulata	22,891	tenuifolia	19,922	rubens	21,567	trichostigma	23,109	quadrangularis	18,128	leucodesmia	19,666	oblongiflora	20,642
plukenetii subsp. bredensis	18,454	caespitosa	19,660	cederbergensis	21,022	richardii	17,870	canaliculata	19,748	saxicola	20,938	benguelensis var. benguelensis	20,778	petrophila	22,042	valida	22,876	gibbosa	19,875	vogelpoelii	21,567	grata	23,062	toringbergensis	18,128	serrata	19,654	bracteolaris	20,614
plukenetii subsp. penicillata	18,454	calcareophila	19,657	coacervata	21,022	salax	17,870	comorensis	19,739	umbratica	20,938	curtophylla	20,770	sylvainiana	21,965	sphaerocephala	22,843	junonia var. junonia	19,871	columnaris	21,543	involucrata	23,035	bodkinii	17,560	shannonii	19,583	dodii	20,607
viridiflora subsp. primulina	18,454	vallis-gratiae	19,657	melastoma subsp. minor	21,022	saxicola	17,870	discolor variant A 'discolor'	19,711	garciae	20,806	rupicola	20,747	melanomontana	21,915	abietina subsp. constantiana	22,839	petricola	19,833	occulta	21,543	didymocarpa	23,024	arborescens	17,546	austronyassana	19,558	polycoma	20,524
melanomontana	18,450	ioniana	19,581	planifolia	21,022	sociorum	17,870	umbelliflora	19,695	grata	20,806	glauca	20,720	phacelanthera	21,915	dracomontana	22,815	stokoeanthus	19,827	virginalis	21,543	exleeana	23,016	baroniana	17,546	affinis	19,447	sphaerocephala	20,524
mucronata	18,433	comorensis	19,493	viridiflora	20,935	sperata	17,870	infundibuliformis	19,691	coronanthera	20,761	glauca var. glauca	20,720	armandiana	21,891	procaviana	22,815	abietina subsp. constantiana	19,805	gigantea	21,524	juniperina	23,016	hermani	17,543	discolor	19,418	velitaris	20,500
armandiana	18,426	eriocodon	19,487	viridiflora subsp. viridiflora	20,935	thodei	17,870	viscaria subsp. longifolia	19,647	planifolia	20,761	quadrangularis	20,707	abietina subsp. diabolis	21,883	leucanthera	22,809	abietina	19,780	coccinea subsp. uniflora	21,501	lageniformis	23,009	krugeri	17,543	jugicola	19,418	nyassana	20,450
empetrina	18,356	banksii subsp. comptonii	19,485	corydalis	20,914	umbratica	17,870	viscaria subsp. macrosepala	19,647	taylorii	20,761	bredasiana	20,668	doliiformis	21,883	oreophila	22,776	rhodopis	19,765	viscaria subsp. pustulata	21,439	tenella	22,992	tetrathecoides	17,543	selaginifolia	19,313	junonia	20,447
hispiduloides	18,348	bracteolaris	19,446	mira	20,878	velatiflora	17,845	capitata	19,489	granulatifolia	20,681	caespitosa	20,668	innovans	21,847	oresigena	22,776	platycalyx	19,747	pyramidalis	21,426	elimensis	22,940	embothriifolia	17,509	cabernetea	19,291	junonia var. minor	20,447
totta	18,348	georgica	19,446	orculiflora	20,878	axillaris	17,604	aghillana	19,468	inclusa	20,681	didymocarpa	20,668	kammanassieae	21,803	plumigera	22,776	blancheana	19,745	phacelanthera	21,310	macrotrema	22,940	banksii	17,261	savileae	19,282	cerinthoides	20,436
milanjiana	18,324	adnata	19,438	oresigena	20,830	serrata	17,604	nudiflora	19,468	langebergensis	20,681	goudotiana	20,668	nubigena	21,681	tegulifolia	22,776	gillii	19,745	obtusata	21,294	nemorosa	22,940	banksii subsp. comptonii	17,261	savileae var. grandiflora	19,282	cerinthoides var. cerinthoides	20,436
afrorum	18,269	arenaria	19,438	gracilis	20,813	puberuliflora	17,598	cruenta	19,465	rhodella	20,681	swaziensis	20,662	intermedia subsp. albiflora	21,452	solandri	22,770	granulatifolia	19,745	inclusa	21,271	denticulata	22,939	banksii subsp. purpurea	17,261	savileae var. mutica	19,282	ceraria	20,405
drakensbergensis	18,269	bicolor	19,438	lignosa	20,806	empetrina	17,544	madida	19,451	axillaris	20,672	pogonanthera	20,651	trichophora	21,411	flavicoma	22,722	langebergensis	19,745	chrysocodon	21,239	remota	22,930	coccinea subsp. uniflora	17,261	savileae var. savileae	19,282	marlothii	20,401
recta	18,269	elimensis	19,438	lusitanica	20,804	flacca	17,443	propendens	19,444	serrata	20,672	gracilipes	20,618	anemodes	21,396	eremioides subsp. eglandula	22,692	lavandulifolia	19,745	jasminiflora	21,207	orculiflora	22,909	intermedia	17,261	denticulata	19,256	suffulta	20,401
anguliger	18,202	humansdorpensis	19,438	unilateralis	20,800	curvifolia	17,387	brachycentra	19,415	atromontana	20,591	outeniquae	20,602	diosmifolia	21,396	malmesburiensis	22,681	melanthera	19,745	cylindrica	21,137	thomae	22,888	intermedia subsp. intermedia	17,261	gerhardii	19,256	carnea	20,376
inflaticalyx	18,202	keetii	19,438	gossypioides	20,768	michellensis	17,315	thimifolia	19,370	nutans	20,532	turbiniflora	20,591	emarginata	21,396	lambertii	22,669	mundii	19,745	retorta	21,137	palliiflora	22,873	plukenetii subsp. bredensis	17,261	afrorum	19,247	pillansii	20,373
rosacea	18,202	onusta	19,438	lateralis	20,731	pillansii subsp. pillansii	17,315	coccinea subsp. uniflora	19,290	flacca	20,511	ioniana	20,590	extrusa	21,396	fascicularis	22,645	pearsoniana	19,745	pseudocalycina	21,106	anomala	22,865	plukenetii subsp. breviflora	17,261	tenella	19,181	brachycentra	20,343
rosacea subsp. glabrata	18,202	parviflora	19,438	tenella	20,731	ribisaria	17,315	intermedia	19,290	cooperi	20,478	urceolata	20,535	keeromsbergensis	21,396	fascicularis var. fascicularis	22,645	peltata	19,745	versicolor	21,090	cruenta	22,862	viridiflora	17,261	blaerioides subsp. blaerioides	19,099	setacea	20,343
rosacea subsp. rosacea	18,202	tenuipes	19,438	inconstans	20,665	solandri	17,315	intermedia subsp. intermedia	19,290	miniscula	20,461	plukenetii subsp. plukenetii	20,531	lachnaeifolia	21,396	concordia	22,616	pilaarkopensis	19,745	genistifolia	21,063	discolor	22,862	arcuata	17,231	fimbriata	19,099	erasmia	20,341
arborescens	18,198	trachysantha	19,438	angulosa	20,659	sparrmannii	17,315	latifolia	19,290	michellensis	20,382	kougabergensis	20,475	nigrimontana	21,396	hermani	22,582	poculiflora	19,745	rubiginosa	21,063	discolor variant B 'speciosa'	22,862	grisbrookii	17,207	oreotragus	19,099	leptopus	20,341
tristis	18,198	krugeri	19,428	fuscescens	20,659	tumida	17,315	physantha	19,290	pillansii subsp. pillansii	20,382	discolor	20,350	pudens	21,396	nivea	22,511	priorii	19,745	dracomontana	20,972	zeyheriana	22,854	fairii	17,182	dolfiana	19,075	vogelpoelii	20,341
papyracea	18,146	salteri	19,413	miniscula	20,620	abietina	17,190	abietina subsp. constantiana	19,214	ribisaria	20,382	discolor variant B 'speciosa'	20,350	thodei	21,396	atropurpurea	22,499	rhodella	19,745	procaviana	20,972	lusitanica	22,835	vallis-aranearum	17,182	lignosa	19,075	microdonta	20,320
turbiniflora	18,089	macilenta	19,393	obconica	20,602	abietina subsp. diabolis	17,190	axilliflora	19,214	solandri	20,382	leucosiphon	20,350	wildii	21,396	patens	22,499	sparsa	19,745	angulosa	20,968	frigida	22,807	tubercularis	17,156	euryphylla	19,067	columnaris	20,317
mafiensis	18,041	glaphyra	19,375	malmesburiensis	20,551	stylaris	17,160	pyramidalis	19,214	sparrmannii	20,382	verticillata	20,262	petrusiana	21,380	holosericea	22,496	tenuipes	19,745	glabripes	20,939	cerinthoides	22,799	feminarum	17,136	lusitanica	19,024	banksii subsp. banksii	20,270
cederbergensis	18,010	petraea	19,364	longipedunculata	20,524	vestita	16,859	curviflora	19,161	steinbergiana	20,374	selaginifolia	20,245	diaphana	21,330	perspicua	22,491	areolata	19,736	varderi	20,898	cerinthoides var. cerinthoides	22,799	seriphiifolia	17,132	whyteana	18,970	mauritiensis	20,220
coacervata	18,010	salax	19,364	peltata	20,524	senilis	16,808	unicolor	19,161	unicolor subsp. mutica	20,302	bergiana	20,221	hottentotica	21,324	perspicua subsp. perspicua	22,491	boucheri	19,736	lignosa	20,880	silvatica	22,770	eugenea	17,024	afra	18,917	annalis	20,202
hippurus	18,010	cerinthoides	19,328	saptouensis	20,524	aspalathifolia	16,775	unicolor subsp. unicolor	19,161	sexfaria	20,300	bruniifolia	20,005	phillipsii	21,324	comorensis subsp. anjurensis	22,476	rigidula	19,736	bokkeveldia	20,865	intonsa	22,754	sessiliflora	17,000	chionodes	18,917	grandiflora subsp. grandiflora	20,202
planifolia	18,010	cerinthoides var. cerinthoides	19,328	areolata	20,515	eustacei	16,775	doliiformis	19,116	abietina	20,257	glumiflora	20,005	umbrosa	21,324	chrysocodon	22,472	heleophila	19,729	strigosa	20,865	melanomontana	22,699	adnata	16,975	glauca	18,826	notholeeana	20,202
opulenta	17,976	barrydalensis	19,320	gracilipes	20,470	pseudocalycina	16,775	ingeana	19,116	conferta	20,257	recurvifolia	19,967	atromontana	21,301	ciliaris	22,425	erigena	19,705	fairii	20,864	truncata	22,692	agglutinans	16,975	glauca var. glauca	18,826	procaviana	20,202
euryphylla	17,972	dispar	19,293	argyraea	20,467	inconstans	16,640	latiflora	19,116	leucopelta	20,196	turgida	19,933	eremioides subsp. pubescens	21,263	haemastoma	22,410	cincta	19,673	wangfatiana	20,840	algida	22,676	dominans	16,975	pycnantha	18,826	radicans subsp. schlechteri	20,202

maximilianii	17,923	tetragona	19,263	aspalathoides	20,467
viridiflora	17,923	aspalathifolia	19,179	chionodes	20,467
viridiflora subsp. viridiflora	17,923	eustacei	19,179	hirta	20,467
petrusiana	17,915	polifolia	19,140	lerouxiae	20,467
thunbergii	17,913	glabella	19,124	filiformis	20,449
corydalis	17,902	glabella subsp. glabella	19,124	involvens	20,449
scytophylla	17,902	glabella subsp. laevis	19,124	sonderiana	20,449
bokkeveldia	17,866	phaeocarpa	19,124	zwartbergensis	20,433
mira	17,866	silvatica	19,087	pycnantha	20,388
nemorosa	17,866	russakiana	19,051	rhopalantha	20,289
orculiflora	17,866	inclusa	19,038	vogelpoelii	20,289
tarantulae	17,866	unicolor subsp. mutica	19,014	formosa	20,275
amidae	17,849	greyi	19,005	gibbosa	20,251
berzelioides	17,849	leucopelta	18,873	amicorum	20,243
leucosiphon	17,849	bakeri	18,861	didymocarpa	20,215
sociorum	17,849	costatisepala	18,861	abbottii	20,213
multumbellifera	17,818	pleiotricha	18,861	flanaganii	20,213
oreophila	17,818	pleiotricha var. blaeriodes	18,861	kingaensis	20,119
oresigena	17,818	pleiotricha var. pleiotricha	18,861	terniflora	20,071
plumigera	17,818	acuta	18,849	hexensis	20,005
tegulifolia	17,818	cedromontana	18,846	plukenetii subsp. breviflora	20,004
alexandri	17,794	cryptanthera	18,846	recurvata	19,983
alexandri subsp. acockii	17,794	myriocodon	18,846	lowryensis	19,969
alexandri subsp. alexandri	17,794	ovina	18,846	ostiaria	19,969
filialis	17,794	propinqua	18,825	conferta	19,952
lignosa	17,794	esterhuyseniae	18,822	amalophylla	19,931
lusitanica	17,791	pannosa	18,822	consobrina	19,919
sexfaria	17,791	andevalensis	18,789	schumannii	19,919
gossypioides	17,756	binaria	18,775	spumosa	19,919
syngenesia	17,743	bombycina	18,769	utriculosa	19,919
lateralis	17,719	radicans	18,769	plukenetii subsp. lineata	19,893
tenella	17,719	radicans subsp. radicans	18,769	inaequalis	19,786
verecunda	17,719	kirstenii	18,760	baroniana	19,770
malmesburiensis	17,539	baueri	18,743	inordinata	19,765
radicans subsp. schlechteri	17,539	baueri subsp. baueri	18,743	nubigena	19,734
blancheana	17,512	dracomontana	18,739	kammanassieae	19,728
comata	17,512	exleeana	18,739	pulvinata	19,710
granulatifolia	17,512	procaviana	18,739	monsoniana	19,681
latifolia	17,512	beatricis	18,736	pyxidiflora	19,661
longipedunculata	17,512	mammosa	18,736	bredasiana	19,660
mundii	17,512	regia	18,736	calcareophila	19,657
newdigateae	17,512	regia subsp. regia	18,736	vallis-gratiae	19,657
pearsoniana	17,512	thomae	18,736	brownleeae	19,636
peltata	17,512	bolusanthus	18,733	ioniana	19,581
saptouensis	17,512	occulta	18,715	comorensis	19,493
sparsa	17,512	virginalis	18,715	eriocodon	19,487
areolata	17,503	orthiocola	18,577	banksii subsp. comptonii	19,485
burchelliana	17,503	bruniades	18,540	adnata	19,438
accommodata	17,437	capitata	18,540	arenaria	19,438
ebracteata	17,437	concordia	18,540	bicolor	19,438
filiformis	17,437	marlothii	18,540	elimensis	19,438
involvens	17,437	petiolaris	18,540	humansdorpensis	19,438
lepidota	17,437	suffulta	18,540	keetii	19,438
penicilliformis	17,437	plukenetii subsp. bredensis	18,454	onusta	19,438
sonderiana	17,437	plukenetii subsp. penicillata	18,454	parviflora	19,438
vlokii	17,437	viridiflora subsp. primulina	18,454	trachysantha	19,438
pubigera	17,384	melanomontana	18,450	riparia	19,433
erasmia	17,277	armandiana	18,426	chamissonis	19,428
leptantha	17,277	empetrina	18,356	krugeri	19,428
oxysepala	17,277	hispiduloides	18,348	brevicaulis	19,416
rhopalantha	17,277	totta	18,348	limnophila	19,401
vogelpoelii	17,277	milanjiana	18,324	etheliae	19,399
senilis	17,250	afrorum	18,269	distorta	19,375
austronyassana	17,240	drakensbergensis	18,269	glaphyra	19,375
oliveranthus	17,240	recta	18,269	petraea	19,364
goatcheriana var. petrensis	17,224	inflaticalyx	18,202	salax	19,364
subdivaricata	17,216	rosacea subsp. glabrata	18,202	depressa	19,342
didymocarpa	17,203	arborescens	18,198	equisetifolia	19,342
goudotiana	17,203	tristis	18,198	micrandra	19,342
abbottii	17,201	cubica	18,192	altevivens	19,293
bolusiae	17,201	turbiniflora	18,089	dispar	19,293
flanaganii	17,201	similis	18,063	oraria	19,293
outeniquae	17,137	mafiensis	18,041	michellensis	19,291
urceolata	17,070	cederbergensis	18,010	pectinifolia	19,224
florifera	17,058	coacervata	18,010	strigilifolia	19,224
banksii subsp. banksii	16,992	hippurus	18,010	petricola	19,219
plukenetii subsp. breviflora	16,992	planifolia	18,010	lasiocarpa	19,185
hillburttii	16,972	opulenta	17,976	stokoei	19,185
guthriei	16,971	blenna	17,931	eustacei	19,179
tenuicaulis	16,934	maximilianii	17,923	polifolia	19,140
amalophylla	16,919	viridiflora	17,923	glabella	19,124
philippioides	16,919	viridiflora subsp. viridiflora	17,923	glabella subsp. glabella	19,124
brachycentra	16,907	petrusiana	17,915	glabella subsp. laevis	19,124
consobrina	16,907	thunbergii	17,913	phaeocarpa	19,124
dominans	16,907	corydalis	17,902	caterviflora	19,110
juniperina	16,907	scytophylla	17,902	flacca	19,095
kougabergensis	16,907	bokkeveldia	17,866	monantha	19,095
macroloma	16,907	mira	17,866	inclusa	19,038
schumannii	16,907	nemorosa	17,866	steinbergiana	19,016
spumosa	16,907	orculiflora	17,866	greyi	19,005
trichoclada	16,907	tarantulae	17,866	praenitens	18,992
utriculosa	16,907	amidae	17,849	azaleifolia	18,987
eriophoros	16,895	berzelioides	17,849	erinus	18,979
setulosa	16,895	leucosiphon	17,849	frigida	18,974
velatiflora	16,895	sociorum	17,849	remota	18,942
plukenetii subsp. lineata	16,881	oreophila	17,818	artemisioides	18,894
nematophylla	16,809	oresigena	17,818	rehmii	18,879
inaequalis	16,774	plumigera	17,818	leucopelta	18,873
longimontana	16,774	tegulifolia	17,818	bakeri	18,861
vallis-fluminis	16,774	dodii	17,801	pleiotricha	18,861
inordinata	16,753	gracilis	17,801	pleiotricha var. blaeriodes	18,861
nubigena	16,722	regerminans	17,801	pleiotricha var. pleiotricha	18,861
filago	16,630	alexandri	17,794	acuta	18,849
rusticula	16,630	alexandri subsp. acockii	17,794	cedromontana	18,846
brownleeae	16,624	alexandri subsp. alexandri	17,794	cryptanthera	18,846
stokoeanthus	16,555	filialis	17,794	myriocodon	18,846
ericoides	16,483	lignosa	17,794	ovina	18,846
riparia	16,421	lusitanica	17,791	propinqua	18,825
chamissonis	16,416	gossypioides	17,756	tysonii	18,824
brevicaulis	16,404	lateralis	17,719	pannosa	18,822
limnophila	16,389	tenella	17,719	andevalensis	18,789
velutina	16,389	verecunda	17,719	binaria	18,775
etheliae	16,387	malmesburiensis	17,539	bombycina	18,769
hottentotica	16,365	radicans subsp. schlechteri	17,539	radicans	18,769
johnstoniana	16,365	blancheana	17,512	radicans subsp. radicans	18,769
phillipsii	16,365	longipedunculata	17,512	kirstenii	18,760
psittacina	16,365	newdigateae	17,512	baueri	18,743
turgida	16,365	peltata	17,512	baueri subsp. baueri	18,743
umbrosa	16,365	saptouensis	17,512	beatricis	18,736
distorta	16,363	sparsa	17,512	occulta	18,715
arborea	16,330	areolata	17,503	virginalis	18,715
depressa	16,330	accommodata	17,437	thamnoides	18,683
equisetifolia	16,330	ebracteata	17,437	tenuifolia	18,644
micrandra	16,330	filiformis	17,437	orthiocola	18,577
nivea	16,330	involvens	17,437	kraussiana	18,554
lithophila	16,303	lepidota	17,437	capitata	18,540
taylorii	16,303	sonderiana	17,437	marlothii	18,540
madida	16,282	vlokii	17,437	petiolaris	18,540
altevivens	16,281	erasmia	17,277	suffulta	18,540
flavicoma	16,281	leptantha	17,277	permutata	18,490
ixanthera	16,281	oxysepala	17,277	sicifolia	18,490
lycopodiastrum	16,281	rhopalantha	17,277	viridiflora subsp. primulina	18,454
oraria	16,281	vogelpoelii	17,277	armandiana	18,426
triflora	16,281	senilis	17,250	jugicola	18,404
michellensis	16,279	austronyassana	17,240	viscaria subsp. longifolia	18,392
oatesii	16,279	goatcheriana var. petrensis	17,224	passerinoides	18,364
jonasiana	16,276	subdivaricata	17,216	empetrina	18,356
viscosissima	16,276	didymocarpa	17,203	hispiduloides	18,348
hendricksei	16,253	goudotiana	17,203	totta	18,348
cuscutiformis	16,245	abbottii	17,201	subverticillaris	18,347
ardens	16,228	bolusiae	17,201	milanjiana	18,324
grisbrookii	16,228	flanaganii	17,201	melanacme	18,298
lambertii	16,228	florifera	17,058	bergiana	18,274
odorata	16,228	banksii subsp. banksii	16,992	velitaris	18,274
pectinifolia	16,212	plukenetii subsp. breviflora	16,992	afrorum	18,269
strigilifolia	16,212	hillburttii	16,972	recta	18,269
petricola	16,207	guthriei	16,971	inflaticalyx	18,202
agglutinans	16,201	filamentosa	16,924	rosacea subsp. glabrata	18,202
lasiocarpa	16,173	amalophylla	16,919	arborescens	18,198
reenensis	16,173	philippioides	16,919	articularis	18,198
schlechteri	16,173	consobrina	16,907	tristis	18,198
stokoei	16,173	dominans	16,907	cubica	18,192
manipuliflora	16,171	kougabergensis	16,907	multiflexuosa	18,105
vagans	16,171	macroloma	16,907	similis	18,063
ustulescens	16,147	schumannii	16,907	mafiensis	18,041
alopecurus	16,106	spumosa	16,907	hippurus	18,010

eremioides subsp. eglandula	16,634	oatesii	19,116	beatricis	20,183
galioides	16,615	pillansii	19,116	montis-hominis	20,159
altevivens	16,530	pillansii subsp. fervida	19,116	plukenetii subsp. plukenetii	20,100
flavicoma	16,530	tetralix	19,116	altiphila	20,061
loganii	16,530	valida	19,116	esteriana	20,061
recurvata	16,530	obconica	19,109	esteriana subsp. esteriana	20,061
scytophylla	16,530	ocellata	19,109	cetrata	19,951
zwartbergensis	16,530	poculiflora	19,109	villosa	19,900
viscaria subsp. pendula	16,504	seriphiifolia	19,036	brevicaulis	19,873
bruniifolia	16,480	parviflora	18,959	aspalathifolia	19,843
glumiflora	16,480	velutina	18,959	pseudocalycina	19,843
petraea	16,480	frigida	18,906	affinis	19,801
eriophoros	16,454	versicolor	18,879	comorensis	19,739
parviporandra	16,368	cereris	18,834	umbelliflora	19,695
terniflora	16,368	leptoclada	18,834	viscaria subsp. pendula	19,572
riparia	16,353	oligantha	18,834	petraea	19,547
albens	16,298	artemisioides	18,825	capitata	19,489
heleophila	16,298	rigidula	18,825	aghillana	19,468
leptopus	16,298	baueri	18,705	nudiflora	19,468
monsoniana	16,298	baueri subsp. baueri	18,705	madida	19,451
navigatoris	16,298	alopecurus	18,676	propendens	19,444
subulata	16,298	holtii	18,676	terniflora	19,436
trichophylla	16,298	pectinifolia	18,676	thimifolia	19,370
dolfiana	16,226	strigilifolia	18,676	leptopus	19,366
revoluta	16,226	inaequalis	18,134	monsoniana	19,366
ventricosa	16,226	inflaticalyx	18,134	subulata	19,366
cerinthoides	16,219	bruniades	18,099	gracilis	19,316
cerinthoides var. barbertona	16,219	marlothii	18,099	revoluta	19,293
cerinthoides var. cerinthoides	16,219	ostiaria	18,099	ventricosa	19,293
amidae	16,069	suffulta	18,099	latifolia	19,290
berzelioides	16,069	viscosissima	18,054	physantha	19,290
bodkinii	16,069	dodii	18,050	abietina subsp. constantiana	19,214
formosa	16,069	blenna	17,893	axilliflora	19,214
glomiflora	16,069	chlorosepala	17,893	pyramidalis	19,214
grisbrookii	16,069	anemodes	17,870	rivularis	19,136
ixanthera	16,069	corydalis	17,870	latiflora	19,116
leucodesmia	16,069	crateriformis	17,870	oatesii	19,116
leucosiphon	16,069	cymosa subsp. cymosa	17,870	pillansii subsp. fervida	19,116
rivularis	16,069	diosmifolia	17,870	tetralix	19,116
tragulifera	16,069	emarginata	17,870	obconica	19,109
bojeri	15,960	fimbriata	17,870	ocellata	19,109
anomala	15,944	keeromsbergensis	17,870	poculiflora	19,109
arachnocalyx	15,944	kirstenii	17,870	seriphiifolia	19,036
cordata	15,944	lachnaeifolia	17,870	roseoloba	19,011
hispiduloides	15,944	lambertii	17,870	viridiflora subsp. redacta	19,011
karooica	15,944	magistrati	17,870	psittacina	18,991
lageniformis	15,944	oakesiorum	17,870	parviflora	18,959
macrophylla	15,944	odorata	17,870	velutina	18,959
magnisylvae	15,944	recurvifolia	17,870	viscaria subsp. pustulata	18,917
petrophila	15,944	richardii	17,870	cereris	18,834
roseoloba	15,944	salax	17,870	leptoclada	18,834
simulans	15,944	saxicola	17,870	oligantha	18,834
totta	15,944	sociorum	17,870	artemisioides	18,825
viridiflora subsp. redacta	15,944	sperata	17,870	rigidula	18,825
vlokii	15,944	thodei	17,870	baueri	18,705
psittacina	15,924	umbratica	17,870	baueri subsp. baueri	18,705
turgida	15,924	gillii	17,768	alopecurus	18,676
amicorum	15,871	axillaris	17,604	holtii	18,676
petrusiana	15,849	serrata	17,604	strigilifolia	18,676
viscaria subsp. pustulata	15,849	empetrina	17,544	extrusa	18,481
comorensis subsp. anjurensis	15,762	burchelliana	17,479	polycoma	18,481
humidicola	15,742	flacca	17,443	sicifolia	18,481
intervallaris	15,742	curvifolia	17,387	utriculosa	18,481
purgatoriensis	15,742	michellensis	17,315	abietina subsp. abietina	18,455
thomensis	15,480	pillansii subsp. pillansii	17,315	grandiflora subsp. perfoliosa	18,455
arenaria	15,413	ribisaria	17,315	verticillata	18,455
cabernetea	15,413	solandri	17,315	orientalis	18,452
elimensis	15,413	sparrmannii	17,315	uberiflora	18,311
extrusa	15,413	tumida	17,315	similis	18,247
keetii	15,413	steinbergiana	17,307	inflaticalyx	18,134
lithophila	15,413	unicolor subsp. mutica	17,234	multiflexuosa	18,130
melanacme	15,413	abietina	17,190	bruniades	18,099
nigrimontana	15,413	abietina subsp. diabolis	17,190	marlothii	18,099
onusta	15,413	conferta	17,190	ostiaria	18,099
oreophila	15,413	plukenetii subsp. plukenetii	17,032	suffulta	18,099
patens	15,413	senilis	16,808	dodii	18,050
polycoma	15,413	aspalathifolia	16,775	drakensbergensis	18,041
sicifolia	15,413	eustacei	16,775	inordinata	18,041
strigosa	15,413	pseudocalycina	16,775	leucantha	18,025
tenuipes	15,413	inconstans	16,640	blenna	17,893
trichoclada	15,413	eremioides subsp. eglandula	16,634	anemodes	17,870
utriculosa	15,413	benthamiana	16,618	corydalis	17,870
viridiflora	15,413	galioides	16,615	crateriformis	17,870
viridiflora subsp. viridiflora	15,413	altevivens	16,530	cymosa subsp. cymosa	17,870
abietina subsp. abietina	15,388	flavicoma	16,530	diosmifolia	17,870
grandiflora subsp. perfoliosa	15,388	loganii	16,530	emarginata	17,870
situshiemalis	15,388	recurvata	16,530	fimbriata	17,870
verticillata	15,388	scytophylla	16,530	keeromsbergensis	17,870
xanthina	15,388	zwartbergensis	16,530	kirstenii	17,870
orientalis	15,384	viscaria subsp. pendula	16,504	lambertii	17,870
sylvainiana	15,326	klotzschii	16,482	magistrati	17,870
fuscescens	15,244	bruniifolia	16,480	oakesiorum	17,870
quadrifida	15,244	glumiflora	16,480	odorata	17,870
uberiflora	15,244	petraea	16,480	recurvifolia	17,870
benguelensis	15,224	eriophoros	16,454	richardii	17,870
benguelensis var. albescens	15,224	dispar	16,409	salax	17,870
benguelensis var. benguelensis	15,224	polifolia	16,377	sociorum	17,870
caprina	15,062	terniflora	16,368	sperata	17,870
lateriflora	15,062	riparia	16,353	thodei	17,870
multiflexuosa	15,062	leptopus	16,298	etheliae	17,796
afrorum	14,973	monsoniana	16,298	dracomontana	17,781
drakensbergensis	14,973	navigatoris	16,298	evansii	17,781
inordinata	14,973	subulata	16,298	procaviana	17,781
arborea	14,958	trichophylla	16,298	gillii	17,768
atricha	14,958	gracilis	16,248	fairii	17,721
austronyassana	14,958	regerminans	16,248	margaritacea	17,721
bakeri	14,958	dolfiana	16,226	turbiniflora	17,721
leucantha	14,958	revoluta	16,226	hebdomadalis	17,620
pycnantha	14,958	ventricosa	16,226	plukenetii subsp. bredensis	17,620
filipendula	14,885	cerinthoides var. barbertona	16,219	plukenetii subsp. penicillata	17,620
gigantea	14,885	amidae	16,069	costatisepala	17,564
outeniquae	14,863	berzelioides	16,069	empetrina	17,544
schelpeorum	14,835	bodkinii	16,069	caterviflora	17,491
urceolata	14,796	formosa	16,069	maderi	17,491
etheliae	14,728	grisbrookii	16,069	mammosa	17,491
lycopodiastrum	14,728	leucosiphon	16,069	regia	17,491
andringitrensis	14,723	rivularis	16,069	regia subsp. regia	17,491
dracomontana	14,714	tragulifera	16,069	scabriuscula	17,491
elsieana	14,714	bojeri	15,960	verecunda	17,491
evansii	14,714	anomala	15,944	burchelliana	17,479
procaviana	14,714	arachnocalyx	15,944	oxycoccifolia	17,439
harveiana	14,678	hispiduloides	15,944	praenitens	17,389
calcareophila	14,653	lageniformis	15,944	curvifolia	17,387
fairii	14,653	magnisylvae	15,944	tysonii	17,386
hameriana	14,653	petrophila	15,944	fontana	17,366
margaritacea	14,653	roseoloba	15,944	hendricksei	17,366
salicina	14,653	simulans	15,944	lananthera	17,366
turbiniflora	14,653	totta	15,944	tumida	17,315
vallis-gratiae	14,653	viridiflora subsp. redacta	15,944	jasminiflora	17,209
adunca	14,553	vlokii	15,944	abietina subsp. diabolis	17,190
chartacea	14,553	psittacina	15,924	zebrensis	17,090
hebdomadalis	14,553	turgida	15,924	annalis	17,064
lasciva	14,553	petrusiana	15,849	leptantha	16,909
plukenetii	14,553	viscaria subsp. pustulata	15,849	rubens	16,909
plukenetii subsp. bredensis	14,553	comorensis subsp. anjurensis	15,762	subverticillaris	16,909
plukenetii subsp. penicillata	14,553	humidicola	15,742	insolitanthera	16,836
pogonanthera	14,553	intervallaris	15,742	supranubia	16,836
costatisepala	14,496	purgatoriensis	15,742	senilis	16,808
goatcheriana var. drakensteinensis	14,496	thomensis	15,480	eustacei	16,775
sicula	14,434	arenaria	15,413	articularis	16,700
sicula subsp. sicula	14,434	cabernetea	15,413	cameronii	16,679
aestiva	14,424	elimensis	15,413	cristata	16,679
caterviflora	14,424	extrusa	15,413	hirtiflora	16,679
colorans	14,424	keetii	15,413	jugicola	16,679
gibbosa	14,424	lithophila	15,413	rufescens	16,679
maderi	14,424	melanacme	15,413	savileae	16,679
mammosa	14,424	nigrimontana	15,413	savileae var. grandiflora	16,679
regia	14,424	onusta	15,413	savileae var. mutica	16,679
regia subsp. regia	14,424	oreophila	15,413	savileae var. savileae	16,679
scabriuscula	14,424	patens	15,413	spumosa	16,679
sessiliflora	14,424	polycoma	15,413	straussiana	16,679
verecunda	14,424	sicifolia	15,413	arcuata	16,650
daphniflora	14,371	strigosa	15,413	inconstans	16,640
leucanthera	14,371	tenuipes	15,413	eremioides subsp. eglandula	16,634
oxycoccifolia	14,371	trichoclada	15,413	benthamiana	16,618
curtophylla	14,318	utriculosa	15,413	galioides	16,615

leucopelta	19,882	glomiflora	21,261	rehmii	22,400
amphigena	19,824	oatesii	21,237	filamentosa	22,370
subulata	19,824	filipendula	21,183	obconica	22,368
tumida	19,809	filipendula subsp. filipendula	21,183	georgica	22,352
valida	19,809	filipendula subsp. parva	21,183	intricata	22,352
goatcheriana	19,775	palliiflora	21,150	glabella subsp. glabella	22,339
sphaerocephala	19,775	permutata	21,121	discolor variant C 'hebecalyx'	22,322
dracomontana	19,747	equisetifolia	21,120	inordinata	22,322
elsieana	19,747	caprina	21,119	fimbriata	22,307
madida	19,747	thimifolia	21,108	ingeana	22,301
procaviana	19,747	globulifera	21,077	dolfiana	22,283
jonasiana	19,741	adnata	20,977	setociliata	22,283
brownii	19,716	blancheana	20,977	mauritanica	22,280
solandri	19,703	lowryensis	20,977	klotzschii	22,272
glandulosa subsp. fourcadei	19,685	macrophylla	20,977	binaria	22,241
trimera	19,676	mundii	20,977	retorta	22,227
trimera subsp. trimera	19,676	pearsoniana	20,977	squarrosa	22,227
blaerioides subsp. blaerioides	19,642	peltata	20,977	supranubia	22,227
malmesburiensis	19,614	simulans	20,977	ventricosa	22,227
radicans subsp. schlechteri	19,614	sparsa	20,977	banksii subsp. comptonii	22,170
galgebergensis	19,605	tenuipes	20,977	bojeri	22,168
ostiaria	19,586	viridiflora subsp. redacta	20,977	parviporandra	22,108
intervallaris	19,559	flexistyla	20,967	chartacea	22,080
capitata	19,549	jasminiflora	20,963	desmantha	22,080
cereris	19,549	lageniformis	20,963	arachnocalyx	22,042
concordia	19,549	blaerioides	20,878	orthiocola	22,042
crassisepala	19,549	blaerioides subsp. hirsuta	20,878	petrophila	22,042
dysantha	19,549	oreotragus	20,878	rufescens	21,989
leptoclada	19,549	haematocodon	20,851	orientalis	21,977
calcicola	19,543	bombycina	20,844	rivularis	21,970
hermani	19,515	radicans subsp. radicans	20,844	umbratica	21,970
polifolia	19,515	xeranthemifolia	20,844	sylvainiana	21,965
kingaensis subsp. rugegensis	19,502	interrupta	20,806	etheliae	21,956
viridiflora	19,462	savileae	20,804	vestita	21,916
viridiflora subsp. primulina	19,462	savileae var. grandiflora	20,804	phacelanthera	21,915
perspicua	19,424	savileae var. mutica	20,804	armandiana	21,891
perspicua subsp. perspicua	19,424	savileae var. savileae	20,804	glandulifera	21,884
ocellata	19,405	aristata	20,780	abietina subsp. diabolis	21,883
baroniana	19,388	conspicua	20,780	laevigata	21,850
boutonii	19,360	infundibuliformis	20,780	innovans	21,847
hispiduloides	19,356	unicolor	20,780	kammanassieae	21,803
totta	19,356	benguelensis	20,778	nubigena	21,681
leucotrachela	19,352	benguelensis var. benguelensis	20,778	areolata	21,579
leucotrachela subsp. leucotrachela	19,352	rupicola	20,747	bolusanthus	21,418
haemastoma	19,342	glauca	20,720	trichophora	21,411
rehmii	19,332	glauca var. glauca	20,720	anemodes	21,396
georgica	19,285	quadrangularis	20,707	diosmifolia	21,396
platycodon	19,285	caespitosa	20,668	emarginata	21,396
glabella	19,271	goudotiana	20,668	keeromsbergensis	21,396
glabella subsp. glabella	19,271	swaziensis	20,662	nigrimontana	21,396
glabella subsp. laevis	19,271	pogonanthera	20,651	pudens	21,396
discolor variant C 'hebecalyx'	19,255	outeniquae	20,602	thodei	21,396
inordinata	19,255	turbiniflora	20,591	wildii	21,396
ingeana	19,234	ioniana	20,590	petrusiana	21,380
macilenta	19,232	urceolata	20,535	hottentotica	21,324
arborescens	19,206	kougabergensis	20,475	atromontana	21,301
cryptoclada	19,206	discolor variant B 'speciosa'	20,350	eremioides subsp. pubescens	21,263
tristis	19,206	leucosiphon	20,350	oatesii	21,237
retorta	19,159	verticillata	20,262	flacca	21,225
squarrosa	19,159	selaginifolia	20,245	filipendula	21,183
supranubia	19,159	bergiana	20,221	filipendula subsp. filipendula	21,183
ventricosa	19,159	globiceps subsp. globiceps	20,203	filipendula subsp. parva	21,183
banksii subsp. comptonii	19,103	glumiflora	20,005	caprina	21,119
guthriei	19,046	recurvifolia	19,967	erinus	21,109
krugeri	19,046	turgida	19,933	thimifolia	21,108
rufescens	18,922	leucopelta	19,882	globulifera	21,077
thunbergii	18,921	amphigena	19,824	humifusa	21,010
orientalis	18,910	subulata	19,824	racemosa	21,009
arenaria	18,902	tumida	19,809	adnata	20,977
grata	18,902	valida	19,809	blancheana	20,977
humansdorpensis	18,902	goatcheriana	19,775	lowryensis	20,977
keetii	18,902	sphaerocephala	19,775	mundii	20,977
magistrati	18,902	dracomontana	19,747	simulans	20,977
oakesiorum	18,902	elsieana	19,747	sparsa	20,977
onusta	18,902	madida	19,747	tenuipes	20,977
rivularis	18,902	procaviana	19,747	parilis	20,971
tragulifera	18,902	jonasiana	19,741	velatiflora	20,971
umbratica	18,902	solandri	19,703	flexistyla	20,967
etheliae	18,888	glandulosa subsp. fourcadei	19,685	lageniformis	20,963
vestita	18,849	trimera	19,676	grandiflora	20,885
glaphyra	18,839	trimera subsp. trimera	19,676	nematophylla	20,885
glandulifera	18,816	blaerioides subsp. blaerioides	19,642	blaerioides	20,878
laevigata	18,783	malmesburiensis	19,614	blaerioides subsp. hirsuta	20,878
fastigiata	18,690	galgebergensis	19,605	haematocodon	20,851
areolata	18,511	ostiaria	19,586	bombycina	20,844
riparia	18,496	intervallaris	19,559	radicans subsp. radicans	20,844
alnea	18,446	cereris	19,549	xeranthemifolia	20,844
dominans	18,446	concordia	19,549	pellucida	20,813
ebracteata	18,446	crassisepala	19,549	praenitens	20,813
lepidota	18,446	dysantha	19,549	interrupta	20,806
vlokii	18,446	leptoclada	19,549	aristata	20,780
spiculifolia	18,408	hermani	19,515	benguelensis	20,778
australis	18,384	polifolia	19,515	benguelensis var. benguelensis	20,778
navigatoris	18,362	kingaensis subsp. rugegensis	19,502	gysbertii	20,765
angulosa	18,350	viridiflora	19,462	densifolia	20,750
bolusanthus	18,350	viridiflora subsp. primulina	19,462	rupicola	20,747
viscosissima	18,350	perspicua	19,424	glauca	20,720
loganii	18,313	perspicua subsp. perspicua	19,424	glauca var. glauca	20,720
puberuliflora	18,313	baroniana	19,388	quadrangularis	20,707
sagittata	18,266	boutonii	19,360	kraussiana	20,684
trichophylla	18,203	hispiduloides	19,356	goudotiana	20,668
occulta	18,179	totta	19,356	swaziensis	20,662
virginalis	18,179	leucotrachela	19,352	pogonanthera	20,651
filago	18,169	leucotrachela subsp. leucotrachela	19,352	outeniquae	20,602
bruniades	18,158	haemastoma	19,342	ioniana	20,590
flacca	18,158	rehmii	19,332	albescens	20,541
suffulta	18,158	georgica	19,285	marifolia	20,541
tradouwensis	18,158	platycodon	19,285	oxyandra	20,541
cooperi	18,138	glabella subsp. glabella	19,271	pubescens	20,541
nutans	18,124	discolor variant C 'hebecalyx'	19,255	kougabergensis	20,475
erinus	18,041	inordinata	19,255	embothriifolia	20,455
mafiensis	18,041	ingeana	19,234	mira	20,398
platycalyx	18,014	macilenta	19,232	orculiflora	20,398
hanekomii	17,961	arborescens	19,206	leucosiphon	20,350
humifusa	17,943	cryptoclada	19,206	insolitanthera	20,344
milanjiana	17,942	retorta	19,159	scabriuscula	20,325
racemosa	17,942	squarrosa	19,159	notholeeana	20,283
amalophylla	17,928	supranubia	19,159	rubens	20,278
cubitans	17,928	ventricosa	19,159	selaginifolia	20,245
philippioides	17,928	banksii subsp. comptonii	19,103	russakiana	20,192
parilis	17,904	guthriei	19,046	accommodata	20,122
setulosa	17,904	krugeri	19,046	filiformis	20,122
velatiflora	17,904	rufescens	18,922	involvens	20,122
grandiflora	17,817	thunbergii	18,921	penicilliformis	20,122
nematophylla	17,817	orientalis	18,910	petraea	20,122
regia	17,817	grata	18,902	ferrea	20,095
regia subsp. regia	17,817	humansdorpensis	18,902	sitiens	20,095
rakotozafyana	17,816	keetii	18,902	toringbergensis	20,095
pellucida	17,746	magistrati	18,902	longistyla	20,051
praenitens	17,746	oakesiorum	18,902	glumiflora	20,005
leucoclada	17,725	onusta	18,902	recurvifolia	19,967
gysbertii	17,697	rivularis	18,902	turgida	19,933
densifolia	17,683	tragulifera	18,902	leucopelta	19,882
inconstans	17,672	umbratica	18,902	amphigena	19,824
rosacea	17,666	etheliae	18,888	tumida	19,809
rosacea subsp. rosacea	17,666	vestita	18,849	leptantha	19,808
barnettiana	17,655	glaphyra	18,839	rhopalantha	19,808
discolor variant A 'discolor'	17,634	glandulifera	18,816	goatcheriana	19,775
kraussiana	17,617	laevigata	18,783	monsoniana	19,755
plena	17,587	fastigiata	18,690	elsieana	19,747
brachyphylla	17,586	areolata	18,511	madida	19,747
harveiana	17,512	alnea	18,446	jonasiana	19,741
albescens	17,474	dominans	18,446	glandulosa subsp. fourcadei	19,685
argyraea	17,474	ebracteata	18,446	trimera	19,676
aspalathoides	17,474	lepidota	18,446	trimera subsp. trimera	19,676
chionodes	17,474	vlokii	18,446	blaerioides subsp. blaerioides	19,642
hippurus	17,474	spiculifolia	18,408	galgebergensis	19,605
hirta	17,474	australis	18,384	ostiaria	19,586
lerouxiae	17,474	navigatoris	18,362	intervallaris	19,559
marifolia	17,474	angulosa	18,350	cereris	19,549
oxyandra	17,474	bolusanthus	18,350	crassisepala	19,549
planifolia	17,474	loganii	18,313	dysantha	19,549
pubescens	17,474	sagittata	18,266	leptoclada	19,549
rudolfii	17,474	trichophylla	18,203	polifolia	19,515
zwartbergensis	17,441	virginalis	18,179	kingaensis subsp. rugegensis	19,502
embothriifolia	17,387	filago	18,169	grandiflora subsp. grandiflora	19,494

accommodata	19,672	manipuliflora	20,831	whyteana	22,652
azaleifolia	19,672	vagans	20,831	empetrina	22,646
sexfaria	19,672	flavicoma	20,829	hansfordii	22,609
crateriformis	19,670	loganii	20,829	pubescens	22,545
karooica	19,670	longimontana	20,809	lucida	22,511
magnisylvae	19,670	pulvinata	20,805	barnettiana	22,510
vlokii	19,670	amphigena	20,790	nivalis	22,470
haematocodon	19,619	arborea	20,790	corydalis	22,450
bolusanthus	19,575	diosmifolia	20,790	euryphylla	22,374
drakensbergensis	19,553	subulata	20,790	sagittata	22,354
berzelioides	19,548	concordia	20,773	saxicola	22,352
altiphila	19,543	uberiflora	20,695	embothriifolia	22,326
holosericea	19,462	gracilipes	20,677	caespitosa	22,286
tradouwensis	19,382	blenna	20,590	passerinoides	22,284
villosa	19,382	hispiduloides	20,581	viscaria subsp. macrosepala	22,275
curvifolia	19,353	roseoloba	20,581	vallis-fluminis	22,200
banksii	19,296	eburnea	20,577	subverticillaris	22,179
banksii subsp. banksii	19,296	longiaristata	20,560	rivularis	22,171
filipendula	19,290	borboniifolia	20,482	alfredii	22,126
affinis	19,245	gerhardii	20,482	similis	22,100
cuscutiformis	19,244	gysbertii	20,482	pumila	22,096
binaria	19,178	walkeri	20,482	inconstans	22,092
didymocarpa	19,178	viscaria subsp. pendula	20,474	caprina	22,086
goudotiana	19,178	gracilis	20,460	perspicua	21,966
trimera	19,178	regia	20,435	perspicua subsp. perspicua	21,966
trimera subsp. trimera	19,178	regia subsp. regia	20,435	toringbergensis	21,966
exleeana	19,170	lowryensis	20,400	montis-hominis	21,959
atricha	19,169	comorensis subsp. comorensis	20,399	inordinata	21,884
trichophylla	19,169	kammanassieae	20,313	longipedunculata	21,789
schelpeorum	19,166	magistrati	20,127	articularis	21,778
amalophylla	19,152	oakesiorum	20,127	pellucida	21,749
philippioides	19,152	tegetiformis	20,127	austronyassana	21,701
tenella	19,145	zwartbergensis	20,127	junonia	21,672
eriophoros	19,131	axillaris	20,089	junonia var. minor	21,672
setulosa	19,128	hillburttii	20,058	ardens	21,654
orculiflora	19,063	marojejyensis	20,055	ioniana	21,644
baueri	19,062	caledonica	20,052	barbigeroides	21,642
baueri subsp. baueri	19,062	glauca	20,052	bredasiana	21,635
parviporandra	19,045	glauca var. glauca	20,052	tristis	21,635
urceolata	19,045	perrieri	19,977	erasmia	21,567
nematophylla	19,042	duthieae	19,922	leptantha	21,567
thomae	19,042	ferrea	19,922	columnaris	21,543
palliiflora	19,027	laeta	19,922	occulta	21,543
imbricata	19,009	pilulifera	19,922	virginalis	21,543
nutans	19,009	tenuifolia	19,922	gigantea	21,524
ostiaria	19,009	gibbosa	19,875	viscaria subsp. longifolia	21,459
zeyheriana	19,008	junonia var. junonia	19,871	pyramidalis	21,426
vestita	19,006	petricola	19,833	subcapitata	21,403
lusitanica	18,988	stokoeanthus	19,827	barrydalensis	21,398
crassisepala	18,971	abietina subsp. constantiana	19,805	notoporina	21,371
frigida	18,961	abietina	19,780	coccinea	21,348
intonsa	18,908	rhodopis	19,765	phacelanthera	21,310
curtophylla	18,897	platycalyx	19,747	obtusata	21,294
eremioides subsp. eglandula	18,891	blancheana	19,745	inclusa	21,271
viridiflora subsp. primulina	18,885	lavandulifolia	19,745	aspalathifolia	21,257
melanomontana	18,853	peltata	19,745	chrysocodon	21,239
rupicola	18,787	pilaarkopensis	19,745	caterviflora	21,229
hansfordii	18,763	priorii	19,745	unicolor subsp. unicolor	21,217
kingaensis subsp. rugegensis	18,736	sparsa	19,745	jasminiflora	21,207
albescens	18,698	tenuipes	19,745	cylindrica	21,137
greyi	18,698	areolata	19,736	supranubia	21,133
oxyandra	18,698	boucheri	19,736	oblongiflora	21,094
pubescens	18,698	rigidula	19,736	genistifolia	21,063
rudolfii	18,698	glandulipila	19,722	rubiginosa	21,063
turneri	18,698	erigena	19,705	dissimulans	21,059
alnea	18,606	cincta	19,673	longistyla	21,059
kougabergensis	18,602	accommodata	19,672	haemastoma	20,976
madida	18,515	azaleifolia	19,672	angulosa	20,968
fimbriata	18,506	crateriformis	19,670	oreotragus	20,924
saxicola	18,506	magnisylvae	19,670	varderi	20,898
perplexa	18,499	vlokii	19,670	bokkeveldia	20,865
dolfiana	18,482	haematocodon	19,619	strigosa	20,865
setociliata	18,482	bolusanthus	19,575	wangfatiana	20,840
embothriifolia	18,480	drakensbergensis	19,553	manipuliflora	20,831
glandulosa	18,453	berzelioides	19,548	vagans	20,831
glandulosa subsp. glandulosa	18,453	altiphila	19,543	flavicoma	20,829
trimera subsp. elgonensis	18,442	holosericea	19,462	loganii	20,829
arborescens	18,440	curviflora	19,446	pulvinata	20,805
caespitosa	18,440	unicolor	19,446	amphigena	20,790
passerinoides	18,438	unicolor subsp. mutica	19,446	arborea	20,790
patersonii	18,407	villosa	19,382	diosmifolia	20,790
vallis-aranearum	18,407	curvifolia	19,353	subulata	20,790
cabernetea	18,372	banksii	19,296	flacca	20,771
melanacme	18,372	banksii subsp. banksii	19,296	marifolia	20,743
maritima	18,354	filipendula	19,290	melastoma	20,685
physantha	18,354	affinis	19,245	melastoma subsp. melastoma	20,685
macroloma	18,333	cuscutiformis	19,244	gracilipes	20,677
subverticillaris	18,333	binaria	19,178	evansii	20,665
pleiotricha	18,325	didymocarpa	19,178	blenna	20,590
pleiotricha var. pleiotricha	18,325	goudotiana	19,178	hispiduloides	20,581
rivularis	18,325	trimera	19,178	roseoloba	20,581
tragulifera	18,325	trimera subsp. trimera	19,178	eburnea	20,577
cereris	18,316	exleeana	19,170	longiaristata	20,560
coronanthera	18,316	atricha	19,169	velitaris	20,535
leptoclada	18,316	trichophylla	19,169	scabriuscula	20,527
oligantha	18,316	schelpeorum	19,166	parilis	20,518
adunca	18,279	amalophylla	19,152	gerhardii	20,482
chartacea	18,279	philippioides	19,152	walkeri	20,482
desmantha	18,279	tenella	19,145	laevigata	20,416
hebdomadalis	18,279	brachycentra	19,140	lowryensis	20,400
lasciva	18,279	eriophoros	19,131	comorensis subsp. comorensis	20,399
oresbia	18,279	setulosa	19,128	erinus	20,397
petraea	18,279	orculiflora	19,063	filiformis	20,324
florifera	18,255	thomae	19,042	involvens	20,324
eugenea	18,250	palliiflora	19,027	kammanassieae	20,313
pumila	18,250	cruenta	19,016	transparens	20,276
arachnocalyx	18,241	discolor	19,016	discolor variant A 'discolor'	20,146
petrophila	18,241	discolor variant B 'speciosa'	19,016	magistrati	20,127
viridiflora	18,230	ostiaria	19,009	oakesiorum	20,127
muscosa	18,220	zeyheriana	19,008	zwartbergensis	20,127
adelopetala	18,157	lusitanica	18,988	hillburttii	20,058
nubigena	18,149	frigida	18,961	marojejyensis	20,055
lateriflora	18,126	cerinthoides	18,953	caledonica	20,052
nervata	18,124	cerinthoides var. cerinthoides	18,953	glauca	20,052
baccans	18,120	intonsa	18,908	glauca var. glauca	20,052
blandfordii	18,120	curtophylla	18,897	perrieri	19,977
regerminans	18,120	eremioides subsp. eglandula	18,891	duthieae	19,922
rimarum	18,120	viridiflora subsp. primulina	18,885	pilulifera	19,922
toringbergensis	18,120	melanomontana	18,853	gibbosa	19,875
perspicua	18,119	rupicola	18,787	junonia var. junonia	19,871
perspicua subsp. perspicua	18,119	hansfordii	18,763	petricola	19,833
artemisioides	18,115	kingaensis subsp. rugegensis	18,736	notholeeana	19,829
plena	18,084	greyi	18,698	stokoeanthus	19,827
inordinata	18,037	pubescens	18,698	oraria	19,821
ustulescens	18,028	alnea	18,606	abietina subsp. constantiana	19,805
adnata	17,943	kougabergensis	18,602	platycalyx	19,747
longipedunculata	17,943	fimbriata	18,506	blancheana	19,745
calcicola	17,942	saxicola	18,506	pilaarkopensis	19,745
articularis	17,932	perplexa	18,499	priorii	19,745
pellucida	17,903	dolfiana	18,482	sparsa	19,745
ebracteata	17,868	setociliata	18,482	tenuipes	19,745
lepidota	17,868	embothriifolia	18,480	boucheri	19,736
barbigeroides	17,796	trimera subsp. elgonensis	18,442	rigidula	19,736
glabella	17,781	arborescens	18,440	glandulipila	19,722
glabella subsp. glabella	17,781	caespitosa	18,440	mira	19,714
glabella subsp. laevis	17,781	passerinoides	18,438	erigena	19,705
betsileana	17,766	patersonii	18,407	arcuata	19,685
arenaria	17,670	vallis-aranearum	18,407	accommodata	19,672
keetii	17,670	cabernetea	18,372	crateriformis	19,670
onusta	17,670	melanacme	18,372	magnisylvae	19,670
vallis-gratiae	17,630	maritima	18,354	vlokii	19,670
viscaria subsp. longifolia	17,613	macroloma	18,333	haematocodon	19,619
anemodes	17,595	subverticillaris	18,333	sitiens	19,569
emarginata	17,595	pleiotricha	18,325	berzelioides	19,548
keeromsbergensis	17,595	pleiotricha var. pleiotricha	18,325	altiphila	19,543
lachnaeifolia	17,595	rivularis	18,325	holosericea	19,462
pudens	17,595	tragulifera	18,325	tubercularis	19,438
salax	17,595	cereris	18,316	villosa	19,382
scytophylla	17,595	coronanthera	18,316	curvifolia	19,353
sperata	17,595	leptoclada	18,316	massonii	19,331
thodei	17,595	oligantha	18,316	squarrosa	19,331
chlorosepala	17,587	desmantha	18,279	banksii	19,296
bruniades	17,580	hebdomadalis	18,279	banksii subsp. banksii	19,296
marlothii	17,580	oresbia	18,279	filipendula	19,290
suffulta	17,580	petraea	18,279	flanaganii	19,279
subcapitata	17,557	florifera	18,255	affinis	19,245

montis-hominis	16,975	bokkeveldia	18,809	micrandra	20,160
nudiflora	16,975	anomala	18,776	nivea	20,160
spumosa	16,975	bombycina	18,776	fausta	20,128
baccans	16,895	calycina	18,776	oakesiorum	20,128
blandfordii	16,895	longistyla	18,776	oophylla	20,128
regerminans	16,895	macrotrema	18,776	oxyandra	20,128
rimarum	16,895	mucronata	18,776	podophylla	20,128
bolusiae	16,863	natalitia	18,776	setosa	20,128
canaliculata	16,760	umbellata	18,776	trichadenia	20,128
floccifera	16,760	racemosa	18,757	oraria	20,106
gillii	16,760	brevifolia	18,722	passerina	20,078
hispidula	16,760	plumigera	18,722	trimera subsp. keniensis	19,949
kraussiana	16,760	tenuifolia	18,697	perspicua	19,937
langebergensis	16,760	junonia var. junonia	18,645	perspicua subsp. latifolia	19,937
melanthera	16,760	corifolia	18,540	perspicua subsp. perspicua	19,937
orthiocola	16,760	ixanthera	18,540	duthieae	19,930
peltata	16,760	rhodopis	18,540	mauritanica	19,930
physantha	16,760	filialis	18,484	petricola	19,868
plumosa	16,760	pannosa	18,484	viscaria subsp. gallorum	19,792
priorii	16,760	setociliata	18,484	dispar	19,787
simulans	16,760	dulcis	18,473	hansfordii	19,787
viscosissima	16,760	patersonii	18,415	manipuliflora	19,773
steinbergiana	16,729	argyraea	18,327	multumbellifera	19,773
cedromontana	16,706	aspalathoides	18,327	vagans	19,773
formosa	16,706	parviflora	18,327	maximilianii	19,772
nivalis	16,706	discolor variant C 'hebecalyx'	18,323	greyi	19,711
ovina	16,706	inordinata	18,323	sicifolia	19,711
straussiana	16,706	coccinea subsp. coccinea	18,292	leucodesmia	19,666
anemodes	16,702	leucotrachela	18,292	serrata	19,654
argentea	16,702	leucotrachela subsp. leucotrachela	18,292	involvens	19,630
consobrina	16,702	vestita	18,292	galpinii	19,622
deflexa	16,702	incarnata	18,258	shannonii	19,583
emarginata	16,702	multiflora	18,258	austronyassana	19,558
extrusa	16,702	pumila	18,258	affinis	19,447
kougabergensis	16,702	brevicaulis	18,144	jugicola	19,418
lachnaeifolia	16,702	cyathiformis	18,128	macrophylla	19,416
rosacea	16,702	quadrangularis	18,128	maritima	19,416
rosacea subsp. glabrata	16,702	gossypioides	18,013	tradouwensis	19,416
rosacea subsp. rosacea	16,702	eustacei	18,003	glandulosa	19,389
scytophylla	16,702	barrydalensis	17,955	glandulosa subsp. glandulosa	19,389
subverticillaris	16,702	passerinoides	17,869	sperata	19,357
trichoclada	16,702	kirstenii	17,583	selaginifolia	19,313
involucrata	16,682	lithophila	17,583	cabernetea	19,291
turgida	16,682	taylorii	17,583	savileae	19,282
octonaria	16,671	bodkinii	17,560	savileae var. grandiflora	19,282
baueri	16,613	valida	17,559	savileae var. mutica	19,282
baueri subsp. baueri	16,613	arborescens	17,546	savileae var. savileae	19,282
baueri subsp. gouriquae	16,613	baroniana	17,546	denticulata	19,256
sacciflora	16,613	hermani	17,543	gerhardii	19,256
transparens	16,465	krugeri	17,543	afrorum	19,247
marojejyensis	16,460	tetrathecoides	17,543	blaerioides subsp. blaerioides	19,099
caledonica	16,457	embothriifolia	17,509	fimbriata	19,099
adunca	16,407	ingeana	17,454	oreotragus	19,099
bolusanthus	16,407	wendlandiana	17,402	dolfiana	19,075
crateriformis	16,407	esterhuyseniae	17,273	lignosa	19,075
eriocephala	16,407	banksii	17,261	euryphylla	19,067
glabella	16,407	banksii subsp. comptonii	17,261	lusitanica	19,024
glabella subsp. glabella	16,407	banksii subsp. purpurea	17,261	afra	18,917
glabella subsp. laevis	16,407	coccinea subsp. uniflora	17,261	chionodes	18,917
hebdomadalis	16,407	intermedia	17,261	haemastoma	18,864
juniperina	16,407	intermedia subsp. intermedia	17,261	unicolor	18,840
karooica	16,407	plukenetii subsp. bredensis	17,261	unicolor subsp. mutica	18,840
karwyderi	16,407	plukenetii subsp. breviflora	17,261	drakensbergensis	18,830
lasciva	16,407	grisbrookii	17,207	glauca	18,826
obconica	16,407	fairii	17,182	glauca var. glauca	18,826
pilaarkopensis	16,407	vallis-aranearum	17,182	pycnantha	18,826
radicans	16,407	tubercularis	17,156	bokkeveldia	18,809
radicans subsp. radicans	16,407	feminarum	17,136	anomala	18,776
vernicosa	16,407	strigilifolia	17,032	bombycina	18,776
viridiflora subsp. redacta	16,407	eugenea	17,024	calycina	18,776
vlokii	16,407	sessiliflora	17,000	longistyla	18,776
pilulifera	16,327	adnata	16,975	macrotrema	18,776
monadelphia	16,166	agglutinans	16,975	mucronata	18,776
lageniformis	16,028	dominans	16,975	racemosa	18,757
x flavisepala	15,905	spumosa	16,975	brevifolia	18,722
conferta	15,899	baccans	16,895	plumigera	18,722
albertyniae	15,745	blandfordii	16,895	tenuifolia	18,697
reunionensis	15,745	regerminans	16,895	amatolensis	18,695
inclusa	15,640	rimarum	16,895	junonia var. junonia	18,645
amoena	15,630	bolusiae	16,863	abietina	18,625
conspicua	15,616	canaliculata	16,760	evansii	18,581
intermedia subsp. albiflora	15,616	floccifera	16,760	globiceps	18,557
oblongiflora	15,616	gillii	16,760	globiceps subsp. gracilis	18,557
glaphyra	15,606	kraussiana	16,760	interrupta	18,557
cryptoclada	15,530	langebergensis	16,760	similis	18,557
navigatoris	15,469	orthiocola	16,760	ixanthera	18,540
versicolor	15,459	peltata	16,760	rhodopis	18,540
ceraria	15,380	physantha	16,760	filialis	18,484
foliacea	15,380	plumosa	16,760	setociliata	18,484
foliacea subsp. foliacea	15,380	priorii	16,760	dulcis	18,473
foliacea subsp. fulgens	15,380	simulans	16,760	obliqua	18,461
ecklonii	15,371	viscosissima	16,760	cruenta	18,410
pillansii	15,348	steinbergiana	16,729	rehmii	18,339
ustulescens	15,330	cedromontana	16,706	argyraea	18,327
banksii subsp. banksii	15,245	formosa	16,706	aspalathoides	18,327
sociorum	15,187	nivalis	16,706	discolor variant C 'hebecalyx'	18,323
viridiflora subsp. viridiflora	15,187	ovina	16,706	inordinata	18,323
boucheri	15,177	straussiana	16,706	aristifolia	18,304
erinus	15,177	anemodes	16,702	tysonii	18,262
malmesburiensis	15,177	argentea	16,702	incarnata	18,258
notholeeana	15,177	consobrina	16,702	multiflora	18,258
procaviana	15,177	deflexa	16,702	dracomontana	18,186
pulchelliflora	15,177	emarginata	16,702	brevicaulis	18,144
radicans subsp. schlechteri	15,177	extrusa	16,702	cyathiformis	18,128
gracilipes	15,174	lachnaeifolia	16,702	quadrangularis	18,128
guthriei	15,174	scytophylla	16,702	gossypioides	18,013
parilis	15,116	subverticillaris	16,702	eustacei	18,003
acuta	15,081	trichoclada	16,702	glandulosa subsp. fourcadei	17,745
altevivens	15,081	involucrata	16,682	kirstenii	17,583
goatcheriana	15,081	turgida	16,682	lithophila	17,583
loganii	15,081	octonaria	16,671	taylorii	17,583
oraria	15,081	baueri	16,613	bodkinii	17,560
intervallaris	15,061	baueri subsp. baueri	16,613	valida	17,559
purgatoriensis	15,061	baueri subsp. gouriquae	16,613	arborescens	17,546
irregularis	15,057	sacciflora	16,613	baroniana	17,546
propinqua	15,057	transparens	16,465	hermani	17,543
virginalis	15,057	marojejyensis	16,460	krugeri	17,543
curvifolia	15,008	caledonica	16,457	tetrathecoides	17,543
jonasiana	14,958	adunca	16,407	embothriifolia	17,509
nutans	14,958	crateriformis	16,407	ingeana	17,454
obtusata	14,958	eriocephala	16,407	wendlandiana	17,402
ostiaria	14,958	glabella	16,407	viscaria subsp. pustulata	17,392
subimbricata	14,958	glabella subsp. glabella	16,407	copiosa	17,341
cinerea	14,900	glabella subsp. laevis	16,407	fuscescens	17,341
mira	14,900	juniperina	16,407	hippurus	17,341
melastoma	14,892	karwyderi	16,407	longimontana	17,341
melastoma subsp. melastoma	14,892	lasciva	16,407	filipendula	17,334
melastoma subsp. minor	14,892	obconica	16,407	filipendula subsp. filipendula	17,334
rupicola	14,786	pilaarkopensis	16,407	esterhuyseniae	17,273
barbigeroides	14,762	radicans	16,407	plukenetii subsp. breviflora	17,261
dispar	14,762	radicans subsp. radicans	16,407	retorta	17,219
hansfordii	14,762	vernicosa	16,407	albospicata	17,209
ioniana	14,762	pilulifera	16,327	articularis	17,209
piquetbergensis	14,762	monadelphia	16,166	grisbrookii	17,207
brachyphylla	14,693	lageniformis	16,028	feminarum	17,136
caprina	14,686	saxigena	15,939	laevigata	17,071
greyi	14,686	x flavisepala	15,905	strigilifolia	17,032
keetii	14,686	conferta	15,899	eugenea	17,024
kingaensis	14,686	glomiflora	15,782	adnata	16,975
kingaensis subsp. rugegensis	14,686	albertyniae	15,745	dominans	16,975
sicifolia	14,686	reunionensis	15,745	spumosa	16,975
zwartbergensis	14,686	inclusa	15,640	glabripes	16,946
accommodata	14,605	amoena	15,630	blandfordii	16,895
alnea	14,605	conspicua	15,616	regerminans	16,895
genistifolia	14,605	intermedia subsp. albiflora	15,616	rimarum	16,895
involvens	14,605	oblongiflora	15,616	abietina subsp. atrorosea	16,824
stokoeanthus	14,605	glaphyra	15,606	regia	16,824
galpinii	14,597	cryptoclada	15,530	regia subsp. regia	16,824
comata	14,391	cerinthoides	15,411	unilateralis	16,814
concordia	14,391	cerinthoides var. cerinthoides	15,411	blenna	16,789
cordata	14,391	ceraria	15,380	fascicularis	16,765
dysantha	14,391	foliacea	15,380	fascicularis var. fascicularis	16,765
flacca	14,391	foliacea subsp. foliacea	15,380	floccifera	16,760
kammanassieae	14,391	foliacea subsp. fulgens	15,380	gillii	16,760
klotzschii	14,391	ecklonii	15,371	langebergensis	16,760
lavandulifolia	14,391	pillansii	15,348	orthiocola	16,760

holtii	16,106	trichoclada	16,907	opulenta	17,976
caterviflora	16,098	utriculosa	16,907	blenna	17,931
cereris	16,083	eriophoros	16,895	maximilianii	17,923
flacca	16,083	setulosa	16,895	petrusiana	17,915
leptoclada	16,083	plukenetii subsp. lineata	16,881	thunbergii	17,913
monantha	16,083	inaequalis	16,774	scytophylla	17,902
oligantha	16,083	inordinata	16,753	bokkeveldia	17,866
calcicola	16,077	nubigena	16,722	nemorosa	17,866
obtusata	16,008	filago	16,630	tarantulae	17,866
subimbricata	16,008	rusticula	16,630	cincta	17,865
azaleifolia	15,975	brownleeae	16,624	doliiformis	17,862
erinus	15,967	stokoeanthus	16,555	valida	17,862
frigida	15,962	ericoides	16,483	amidae	17,849
ocellata	15,939	riparia	16,421	berzelioides	17,849
platycalyx	15,939	chamissonis	16,416	leucosiphon	17,849
remota	15,930	brevicaulis	16,404	sociorum	17,849
boutonii	15,894	limnophila	16,389	oreophila	17,818
nervata	15,891	velutina	16,389	plumigera	17,818
zitzikammensis	15,891	etheliae	16,387	tegulifolia	17,818
artemisioides	15,882	hottentotica	16,365	dodii	17,801
aneimena	15,867	johnstoniana	16,365	regerminans	17,801
rehmii	15,867	phillipsii	16,365	alexandri	17,794
genistifolia	15,865	psittacina	16,365	alexandri subsp. acockii	17,794
jananthus	15,865	turgida	16,365	alexandri subsp. alexandri	17,794
rubiginosa	15,865	umbrosa	16,365	filialis	17,794
curtophylla	15,812	distorta	16,363	canescens	17,756
tysonii	15,812	arborea	16,330	atricha	17,721
stylaris	15,800	depressa	16,330	corifolia	17,721
glauca	15,762	equisetifolia	16,330	leptopus	17,721
glauca var. glauca	15,762	micrandra	16,330	leucantha	17,721
cryptoclada	15,741	nivea	16,330	lutea	17,721
rakotozafyana	15,741	lithophila	16,303	palliiflora	17,721
montis-hominis	15,671	taylorii	16,303	verecunda	17,719
thamnoides	15,671	altevivens	16,281	margaritacea	17,697
benguelensis	15,665	flavicoma	16,281	chiroptera	17,641
benguelensis var. albescens	15,665	lycopodiastrum	16,281	intervallaris	17,613
benguelensis var. benguelensis	15,665	oraria	16,281	evansii	17,598
galioides	15,665	triflora	16,281	maderi	17,595
tenuifolia	15,632	michellensis	16,279	ventricosa	17,595
lanceolifera	15,619	oatesii	16,279	radicans subsp. schlechteri	17,539
trichostigma	15,596	jonasiana	16,276	notoporina	17,538
kraussiana	15,542	hendricksei	16,253	salicina	17,538
eglandulosa	15,480	cuscutiformis	16,245	sitiens	17,538
botryoides	15,478	grisbrookii	16,228	blancheana	17,512
permutata	15,478	lambertii	16,228	newdigateae	17,512
sicifolia	15,478	odorata	16,228	sparsa	17,512
tetrathecoides	15,445	pectinifolia	16,212	canaliculata	17,489
plukenetii subsp. plukenetii	15,419	strigilifolia	16,212	humifusa	17,489
discolor	15,392	petricola	16,207	amatolensis	17,453
discolor variant B 'speciosa'	15,392	agglutinans	16,201	parilis	17,450
excavata	15,392	lasiocarpa	16,173	accommodata	17,437
jugicola	15,392	reenensis	16,173	ebracteata	17,437
viscaria subsp. longifolia	15,380	schlechteri	16,173	lepidota	17,437
passerinoides	15,352	stokoei	16,173	vlokii	17,437
subverticillaris	15,334	manipuliflora	16,171	chonantha	17,415
varderi	15,294	vagans	16,171	oresbia	17,415
melanacme	15,286	ustulescens	16,147	abietina	17,364
polycoma	15,286	alopecurus	16,106	shannonii	17,362
selaginifolia	15,286	holtii	16,106	diaphana	17,308
truncata	15,286	caterviflora	16,098	discolor variant C 'hebecalyx'	17,308
algida	15,270	cereris	16,083	erasmia	17,277
bergiana	15,262	flacca	16,083	leptantha	17,277
irregularis	15,262	leptoclada	16,083	oxysepala	17,277
velitaris	15,262	monantha	16,083	senilis	17,250
fastigiata	15,225	oligantha	16,083	austronyassana	17,240
viscaria subsp. macrosepala	15,188	calcicola	16,077	goatcheriana var. petrensis	17,224
articularis	15,186	obtusata	16,008	subdivaricata	17,216
densifolia	15,181	subimbricata	16,008	goudotiana	17,203
tragomontana	15,168	steinbergiana	16,004	bolusiae	17,201
lateriflora	15,127	praenitens	15,980	borboniifolia	17,129
multiflexuosa	15,093	azaleifolia	15,975	florifera	17,058
alnea	14,980	erinus	15,967	banksii subsp. banksii	16,992
karwyderi	14,980	trichophora	15,965	cameronii	16,975
nudiflora	14,980	frigida	15,962	hillburttii	16,972
umbellata	14,980	platycalyx	15,939	guthriei	16,971
robynsiana	14,925	remota	15,930	filamentosa	16,924
cincta	14,853	boutonii	15,894	philippioides	16,919
doliiformis	14,850	nervata	15,891	dominans	16,907
latiflora	14,850	zitzikammensis	15,891	kougabergensis	16,907
tetralix	14,850	artemisioides	15,882	macroloma	16,907
tumida	14,850	aneimena	15,867	trichoclada	16,907
valida	14,850	rehmii	15,867	eriophoros	16,895
puberuliflora	14,847	genistifolia	15,865	setulosa	16,895
benthamiana	14,840	jananthus	15,865	cumuliflora	16,873
glandulosa	14,827	rubiginosa	15,865	glandulifera	16,870
glandulosa subsp. glandulosa	14,827	curtophylla	15,812	hirtiflora	16,870
australis	14,817	tysonii	15,812	straussiana	16,870
galgebergensis	14,814	glauca	15,762	laevigata	16,836
bodkinii	14,799	glauca var. glauca	15,762	embothriifolia	16,831
richardii	14,799	cryptoclada	15,741	notholeeana	16,762
passerina	14,796	rakotozafyana	15,741	viscaria subsp. pendula	16,738
humidicola	14,793	montis-hominis	15,671	physophylla	16,726
pilosiflora	14,772	thamnoides	15,671	goatcheriana var. goatcheriana	16,653
pilosiflora subsp. pilosiflora	14,772	benguelensis	15,665	filago	16,630
canescens	14,744	benguelensis var. albescens	15,665	rusticula	16,630
ribisaria	14,744	benguelensis var. benguelensis	15,665	stokoeanthus	16,555
solandri	14,744	galioides	15,665	longistyla	16,530
filipendula	14,742	tenuifolia	15,632	ericoides	16,483
filipendula subsp. parva	14,742	lanceolifera	15,619	coccinea	16,477
sessiliflora	14,742	trichostigma	15,596	transparens	16,443
albens	14,709	kraussiana	15,542	zeyheriana	16,411
atricha	14,709	eglandulosa	15,480	velutina	16,389
corifolia	14,709	botryoides	15,478	tubercularis	16,371
leptopus	14,709	permutata	15,478	hottentotica	16,365
leucantha	14,709	sicifolia	15,478	johnstoniana	16,365
lutea	14,709	excavata	15,392	phillipsii	16,365
palliiflora	14,709	jugicola	15,392	psittacina	16,365
klotzschii	14,703	viscaria subsp. longifolia	15,380	turgida	16,365
margaritacea	14,685	passerinoides	15,352	umbrosa	16,365
goatcheriana var. drakensteinensis	14,656	subverticillaris	15,334	arborea	16,330
heleophila	14,656	varderi	15,294	nivea	16,330
hexandra	14,637	melanacme	15,286	lithophila	16,303
chiroptera	14,629	polycoma	15,286	taylorii	16,303
collina	14,601	selaginifolia	15,286	flavicoma	16,281
intervallaris	14,601	truncata	15,286	lycopodiastrum	16,281
evansii	14,586	algida	15,270	triflora	16,281
colorans	14,583	bergiana	15,262	oatesii	16,279
maderi	14,583	irregularis	15,262	jonasiana	16,276
ventricosa	14,583	velitaris	15,262	hendricksei	16,253
notoporina	14,526	fastigiata	15,225	cuscutiformis	16,245
physodes	14,526	viscaria subsp. macrosepala	15,188	sparrmannii	16,241
quadrangularis	14,526	articularis	15,186	grisbrookii	16,228
salicina	14,526	tragomontana	15,168	lambertii	16,228
sitiens	14,526	lateriflora	15,127	odorata	16,228
canaliculata	14,477	multiflexuosa	15,093	agglutinans	16,201
humifusa	14,477	alnea	14,980	reenensis	16,173
cubitans	14,462	karwyderi	14,980	schlechteri	16,173
banksii subsp. purpurea	14,460	nudiflora	14,980	manipuliflora	16,171
jasminiflora	14,460	umbellata	14,980	vagans	16,171
lageniformis	14,460	robynsiana	14,925	ustulescens	16,147
amatolensis	14,441	cincta	14,853	alopecurus	16,106
parilis	14,438	doliiformis	14,850	holtii	16,106
chonantha	14,403	latiflora	14,850	glabripes	16,098
omninoglabra	14,403	tetralix	14,850	cereris	16,083
oresbia	14,403	tumida	14,850	leptoclada	16,083
limosa	14,388	valida	14,850	oligantha	16,083
abietina	14,352	australis	14,817	calcicola	16,077
grandiflora	14,352	galgebergensis	14,814	eburnea	16,076
grandiflora subsp. grandiflora	14,352	bodkinii	14,799	obtusata	16,008
adelopetala	14,350	richardii	14,799	subimbricata	16,008
daphniflora	14,350	passerina	14,796	trichophora	15,965
shannonii	14,350	humidicola	14,793	platycalyx	15,939
diaphana	14,296	pilosiflora	14,772	annalis	15,935
discolor variant C 'hebecalyx'	14,296	pilosiflora subsp. pilosiflora	14,772	perspicua	15,933
glandulipila	14,296	canescens	14,744	perspicua subsp. perspicua	15,933
cremea	14,277	ribisaria	14,744	paludicola	15,914
infundibuliformis	14,277	solandri	14,744	abietina subsp. atrorosea	15,902
vestita	14,273	filipendula	14,742	viscaria subsp. pustulata	15,902
leucoclada	14,259	filipendula subsp. parva	14,742	boutonii	15,894
axilliflora	14,246	atricha	14,709	nervata	15,891
brachyphylla	14,120	corifolia	14,709	zitzikammensis	15,891
borboniifolia	14,117	leptopus	14,709	aneimena	15,867
cameronii	13,963	leucantha	14,709	genistifolia	15,865
rufescens	13,963	lutea	14,709	jananthus	15,865
wendlandiana	13,963	palliiflora	14,709	rubiginosa	15,865
cruenta	13,930	margaritacea	14,685	fontana	15,861

glandulipila	14,318	viridiflora	15,413	dissimulans	16,554
nubigena	14,318	viridiflora subsp. viridiflora	15,413	lavandulifolia	16,554
tysonii	14,318	abietina subsp. abietina	15,388	peltata	16,554
ampullacea	14,299	grandiflora subsp. perfoliosa	15,388	tubercularis	16,554
fontana	14,299	situshiemalis	15,388	areolata	16,545
hendricksei	14,299	verticillata	15,388	altevivens	16,530
lananthera	14,299	xanthina	15,388	flavicoma	16,530
rosacea	14,177	orientalis	15,384	loganii	16,530
rosacea subsp. rosacea	14,177	sylvainiana	15,326	recurvata	16,530
hebeclada	14,158	fuscescens	15,244	scytophylla	16,530
parkeri	14,158	uberiflora	15,244	zwartbergensis	16,530
perhispida	14,158	benguelensis	15,224	klotzschii	16,482
ebracteata	14,142	benguelensis var. albescens	15,224	bruniifolia	16,480
jasminiflora	14,142	benguelensis var. benguelensis	15,224	glumiflora	16,480
lepidota	14,142	similis	15,179	eriophoros	16,454
obtusata	14,142	caprina	15,062	dispar	16,409
viridiflora subsp. primulina	14,142	lateriflora	15,062	bolusanthus	16,384
gracilipes	14,023	multiflexuosa	15,062	polifolia	16,377
juniperina	14,023	afrorum	14,973	riparia	16,353
kougabergensis	14,023	drakensbergensis	14,973	navigatoris	16,298
uysii	14,023	inordinata	14,973	trichophylla	16,298
zebrensis	14,023	arborea	14,958	banksii	16,280
annalis	13,997	atricha	14,958	banksii subsp. comptonii	16,280
bokkeveldia	13,841	austronyassana	14,958	regerminans	16,248
caledonica	13,841	bakeri	14,958	dolfiana	16,226
cuscutiformis	13,841	leucantha	14,958	palliiflora	16,223
goatcheriana var. petrensis	13,841	filipendula	14,885	cerinthoides var. barbertona	16,219
inamoena	13,841	schelpeorum	14,835	bredasiana	16,209
leptantha	13,841	etheliae	14,728	caespitosa	16,209
macilenta	13,841	lycopodiastrum	14,728	chiroptera	16,203
nemorosa	13,841	andringitrensis	14,723	amidae	16,069
rubens	13,841	dracomontana	14,714	berzelioides	16,069
subverticillaris	13,841	evansii	14,714	bodkinii	16,069
barrydalensis	13,769	procaviana	14,714	formosa	16,069
filialis	13,769	harveiana	14,678	grisbrookii	16,069
insolitanthera	13,769	calcareophila	14,653	leucosiphon	16,069
irregularis	13,769	fairii	14,653	tragulifera	16,069
saxigena	13,769	hameriana	14,653	plukenetii subsp. lineata	16,048
schlechteri	13,769	margaritacea	14,653	passerinoides	16,015
supranubia	13,769	salicina	14,653	exleeana	15,979
swaziensis	13,769	turbiniflora	14,653	intermedia subsp. albiflora	15,976
silvatica	13,671	vallis-gratiae	14,653	bojeri	15,960
articularis	13,633	prolata	14,575	anomala	15,944
adnata	13,612	hebdomadalis	14,553	arachnocalyx	15,944
cameronii	13,612	plukenetii subsp. bredensis	14,553	hispiduloides	15,944
cristata	13,612	plukenetii subsp. penicillata	14,553	lageniformis	15,944
discolor	13,612	pogonanthera	14,553	magnisylvae	15,944
discolor variant B 'speciosa'	13,612	costatisepala	14,496	petrophila	15,944
dominans	13,612	goatcheriana var. drakensteinensis	14,496	simulans	15,944
excavata	13,612	sicula	14,434	totta	15,944
garciae	13,612	sicula subsp. sicula	14,434	vlokii	15,944
grata	13,612	aestiva	14,424	turgida	15,924
hirtiflora	13,612	caterviflora	14,424	thamnoides	15,854
humansdorpensis	13,612	colorans	14,424	petrusiana	15,849
jugicola	13,612	gibbosa	14,424	accommodata	15,819
lanuginosa	13,612	maderi	14,424	filiformis	15,819
maximilianii	13,612	mammosa	14,424	involvens	15,819
multumbellifera	13,612	regia	14,424	comorensis subsp. anjurensis	15,762
plumigera	13,612	regia subsp. regia	14,424	humidicola	15,742
rufescens	13,612	scabriuscula	14,424	intervallaris	15,742
savileae	13,612	verecunda	14,424	purgatoriensis	15,742
savileae var. grandiflora	13,612	leucanthera	14,371	subcapitata	15,679
savileae var. mutica	13,612	oxycoccifolia	14,371	perspicua	15,637
savileae var. savileae	13,612	praenitens	14,322	perspicua subsp. perspicua	15,637
selaginifolia	13,612	curtophylla	14,318	diaphana	15,584
spumosa	13,612	nubigena	14,318	discolor variant C 'hebecalyx'	15,584
straussiana	13,612	tysonii	14,318	rhopalantha	15,568
wendlandiana	13,612	ampullacea	14,299	retorta	15,565
arcuata	13,583	fontana	14,299	brachialis	15,496
unilateralis	13,583	hendricksei	14,299	thomensis	15,480
kingaensis subsp. rugegensis	13,567	lananthera	14,299	irrorata	15,449
blancheana	13,487	hebeclada	14,158	arenaria	15,413
dissimulans	13,487	parkeri	14,158	cabernetea	15,413
granulatifolia	13,487	perhispida	14,158	elimensis	15,413
hexensis	13,487	ebracteata	14,142	keetii	15,413
inclusa	13,487	jasminiflora	14,142	lithophila	15,413
irbyana	13,487	lepidota	14,142	melanacme	15,413
langebergensis	13,487	obtusata	14,142	nigrimontana	15,413
lavandulifolia	13,487	viridiflora subsp. primulina	14,142	onusta	15,413
macrotrema	13,487	gracilipes	14,023	oreophila	15,413
nervata	13,487	kougabergensis	14,023	patens	15,413
orthiocola	13,487	uysii	14,023	strigosa	15,413
pearsoniana	13,487	zebrensis	14,023	tenuipes	15,413
peltata	13,487	annalis	13,997	trichoclada	15,413
pilaarkopensis	13,487	bokkeveldia	13,841	viridiflora	15,413
rhodella	13,487	caledonica	13,841	viridiflora subsp. viridiflora	15,413
sparsa	13,487	cuscutiformis	13,841	situshiemalis	15,388
tubercularis	13,487	goatcheriana var. petrensis	13,841	xanthina	15,388
areolata	13,478	inamoena	13,841	haemastoma	15,339
boucheri	13,478	leptantha	13,841	taxifolia	15,339
phacelanthera	13,478	nemorosa	13,841	sylvainiana	15,326
angulosa	13,442	rubens	13,841	notholeeana	15,324
densifolia	13,401	subverticillaris	13,841	botryoides	15,288
nutans	13,337	filialis	13,769	permutata	15,288
bolusanthus	13,317	insolitanthera	13,769	ferrea	15,264
pulvinata	13,313	irregularis	13,769	glabripes	15,264
miniscula	13,266	saxigena	13,769	laeta	15,264
stokoeanthus	13,260	schlechteri	13,769	fuscescens	15,244
banksii	13,212	supranubia	13,769	benguelensis	15,224
banksii subsp. banksii	13,212	swaziensis	13,769	benguelensis var. albescens	15,224
banksii subsp. comptonii	13,212	silvatica	13,671	benguelensis var. benguelensis	15,224
banksii subsp. purpurea	13,212	articularis	13,633	longistyla	15,214
plukenetii subsp. breviflora	13,212	adnata	13,612	coccinea	15,163
lanipes	13,156	cameronii	13,612	melastoma	15,163
micrandra	13,156	cristata	13,612	melastoma subsp. melastoma	15,163
palliiflora	13,156	dominans	13,612	atrovinosa	15,107
pleiotricha	13,156	excavata	13,612	mira	15,107
pleiotricha var. pleiotricha	13,156	hirtiflora	13,612	physophylla	15,107
bredasiana	13,141	humansdorpensis	13,612	rhodopis	15,107
caespitosa	13,141	jugicola	13,612	urna-viridis	15,107
esteriana subsp. swartbergensis	13,141	lanuginosa	13,612	caprina	15,062
perplexa	13,141	maximilianii	13,612	lateriflora	15,062
chiroptera	13,135	plumigera	13,612	bergiana	15,034
manipuliflora	13,106	rufescens	13,612	glutinosa	15,034
sexfaria	13,106	savileae	13,612	lasiocarpa	15,034
vagans	13,106	savileae var. grandiflora	13,612	reenensis	15,034
filipendula subsp. parva	13,083	savileae var. mutica	13,612	brownleeae	15,005
ioniana	13,063	savileae var. savileae	13,612	humifusa	14,982
glandulosa	13,047	selaginifolia	13,612	afrorum	14,973
glandulosa subsp. glandulosa	13,047	spumosa	13,612	arborea	14,958
racemosa	13,033	straussiana	13,612	atricha	14,958
thunbergii	13,031	wendlandiana	13,612	austronyassana	14,958
jananthus	12,981	arcuata	13,583	bakeri	14,958
krugeri	12,981	unilateralis	13,583	conspicua	14,909
plukenetii subsp. lineata	12,981	kingaensis subsp. rugegensis	13,567	cygnea	14,909
glaphyra	12,978	blancheana	13,487	cyrilliflora	14,909
hillburttii	12,947	dissimulans	13,487	gossypioides	14,909
passerinoides	12,947	hexensis	13,487	gysbertii	14,909
exleeana	12,912	irbyana	13,487	intonsa	14,909
petricola	12,912	lavandulifolia	13,487	oblongiflora	14,909
intermedia subsp. albiflora	12,908	macrotrema	13,487	filipendula	14,885
eburnea	12,852	nervata	13,487	ericoides	14,864
florifera	12,852	orthiocola	13,487	schelpeorum	14,835
rosacea subsp. glabrata	12,787	peltata	13,487	lycopodiastrum	14,728
thamnoides	12,787	pilaarkopensis	13,487	andringitrensis	14,723
hexandra	12,767	sparsa	13,487	harveiana	14,678
accommodata	12,751	tubercularis	13,487	calcareophila	14,653
filiformis	12,751	areolata	13,478	hameriana	14,653
imbricata	12,751	boucheri	13,478	salicina	14,653
involvens	12,751	phacelanthera	13,478	vallis-gratiae	14,653
penicilliformis	12,751	angulosa	13,442	prolata	14,575
sonderiana	12,751	bolusanthus	13,317	pogonanthera	14,553
subimbricata	12,751	pulvinata	13,313	goatcheriana var. drakensteinensis	14,496
denticulata	12,727	stokoeanthus	13,260	azaleifolia	14,478
plena	12,727	banksii	13,212	erigena	14,454
opulenta	12,682	banksii subsp. banksii	13,212	sicula	14,434
subcapitata	12,611	banksii subsp. comptonii	13,212	sicula subsp. sicula	14,434
genistifolia	12,570	banksii subsp. purpurea	13,212	aestiva	14,424
perspicua	12,570	plukenetii subsp. breviflora	13,212	colorans	14,424
perspicua subsp. perspicua	12,570	lanipes	13,156	gibbosa	14,424
rubiginosa	12,570	micrandra	13,156	leucanthera	14,371
shannonii	12,570	palliiflora	13,156	curtophylla	14,318
diaphana	12,516	pleiotricha	13,156	nubigena	14,318
discolor variant A 'discolor'	12,516	pleiotricha var. pleiotricha	13,156	ampullacea	14,299
discolor variant C 'hebecalyx'	12,516	bredasiana	13,141	glabella	14,282
astroites	12,501	caespitosa	13,141	glabella subsp. glabella	14,282

maximilianii	17,387	bruniades	18,158	arcuata	19,484
viridiflora subsp. viridiflora	17,387	flacca	18,158	viridiflora	19,462
bokkeveldia	17,330	suffulta	18,158	viridiflora subsp. primulina	19,462
mira	17,330	tradouwensis	18,158	abietina subsp. abietina	19,456
orculiflora	17,330	cooperi	18,138	grandiflora subsp. perfoliosa	19,456
junonia	17,316	nutans	18,124	utriculosa	19,438
junonia var. junonia	17,316	erinus	18,041	abietina subsp. atrorosea	19,423
formosa	17,282	mafiensis	18,041	viscaria subsp. pustulata	19,423
leucodesmia	17,282	baueri subsp. gouriquae	17,990	baroniana	19,388
insolitanthera	17,277	penduliflora	17,990	boutonii	19,360
aestiva	17,258	hanekomii	17,961	hispiduloides	19,356
filialis	17,258	humifusa	17,943	totta	19,356
gibbosa	17,258	milanjiana	17,942	leucotrachela	19,352
scabriuscula	17,258	racemosa	17,942	leucotrachela subsp. leucotrachela	19,352
notholeeana	17,216	amalophylla	17,928	viscaria subsp. viscaria	19,351
petricola	17,216	cubitans	17,928	uberiflora	19,343
atricha	17,210	philippioides	17,928	platycodon	19,285
corifolia	17,210	parilis	17,904	viscaria subsp. macrosepala	19,264
rubens	17,210	setulosa	17,904	aspalathifolia	19,254
phaeocarpa	17,197	velatiflora	17,904	macilenta	19,232
mollis	17,171	grandiflora	17,817	arborescens	19,206
russakiana	17,124	nematophylla	17,817	cryptoclada	19,206
burchelliana	17,120	rakotozafyana	17,816	guthriei	19,046
cerinthoides	17,093	pellucida	17,746	krugeri	19,046
cerinthoides var. cerinthoides	17,093	praenitens	17,746	transparens	19,000
accommodata	17,055	leucoclada	17,725	costatisepala	18,936
filiformis	17,055	gysbertii	17,697	thunbergii	18,921
involvens	17,055	densifolia	17,683	glandulosa	18,903
juniperina	17,055	inconstans	17,672	glandulosa subsp. glandulosa	18,903
karwyderi	17,055	rosacea	17,666	grata	18,902
penicilliformis	17,055	rosacea subsp. rosacea	17,666	humansdorpensis	18,902
petraea	17,055	barnettiana	17,655	keetii	18,902
sonderiana	17,055	kraussiana	17,617	magistrati	18,902
umbellata	17,055	plena	17,587	oakesiorum	18,902
ferrea	17,028	brachyphylla	17,586	onusta	18,902
sitiens	17,028	harveiana	17,512	tragulifera	18,902
toringbergensis	17,028	albescens	17,474	esterhuyseniae	18,897
obtusata	17,017	argyraea	17,474	johnstoniana	18,897
recurvata	16,990	aspalathoides	17,474	glaphyra	18,839
longistyla	16,984	chionodes	17,474	michellensis	18,810
oliveri	16,984	hippurus	17,474	borboniifolia	18,723
tetrathecoides	16,984	hirta	17,474	simii	18,711
remota	16,939	lerouxiae	17,474	chiroptera	18,705
artemisioides	16,890	marifolia	17,474	lasiocarpa	18,705
genistifolia	16,873	oxyandra	17,474	reenensis	18,705
rubiginosa	16,873	planifolia	17,474	fastigiata	18,690
humbertii	16,866	pubescens	17,474	inflata	18,690
seriphiifolia	16,801	rudolfii	17,474	evansii	18,662
silvatica	16,778	zwartbergensis	17,441	azaleifolia	18,660
erasmia	16,741	embothriifolia	17,387	cameronii	18,569
leptantha	16,741	maximilianii	17,387	alnea	18,446
rhopalantha	16,741	viridiflora subsp. viridiflora	17,387	dominans	18,446
pulvinata	16,717	bokkeveldia	17,330	ebracteata	18,446
hexandra	16,712	mira	17,330	lepidota	18,446
goatcheriana var. petrensis	16,688	orculiflora	17,330	vlokii	18,446
monsoniana	16,688	junonia	17,316	daphniflora	18,426
galioides	16,674	junonia var. junonia	17,316	spiculifolia	18,408
minutifolia	16,674	formosa	17,282	australis	18,384
parkeri	16,674	leucodesmia	17,282	navigatoris	18,362
perhispida	16,674	insolitanthera	17,277	conspicua subsp. conspicua	18,353
gigantea	16,669	aestiva	17,258	quadrisulcata	18,353
calcareophila	16,664	filialis	17,258	unicolor subsp. mutica	18,353
vallis-gratiae	16,664	gibbosa	17,258	angulosa	18,350
banksii subsp. banksii	16,609	scabriuscula	17,258	drakensbergensis	18,343
plukenetii subsp. breviflora	16,609	notholeeana	17,216	tysonii	18,343
eriophoros	16,513	petricola	17,216	loganii	18,313
plukenetii subsp. lineata	16,499	atricha	17,210	hansfordii	18,296
eglandulosa	16,489	rubens	17,210	sagittata	18,266
hillburttii	16,436	phaeocarpa	17,197	trichophylla	18,203
grandiflora subsp. grandiflora	16,426	mollis	17,171	virginalis	18,179
arcuata	16,416	russakiana	17,124	filago	18,169
unilateralis	16,416	burchelliana	17,120	tenuifolia	18,164
abietina subsp. abietina	16,389	accommodata	17,055	bruniades	18,158
grandiflora subsp. perfoliosa	16,389	filiformis	17,055	suffulta	18,158
latiflora	16,389	involvens	17,055	tradouwensis	18,158
situshiemalis	16,389	juniperina	17,055	cooperi	18,138
xanthina	16,389	karwyderi	17,055	nutans	18,124
macroloma	16,371	penicilliformis	17,055	annalis	18,065
salax	16,371	petraea	17,055	brachycentra	18,048
schumannii	16,371	sonderiana	17,055	mafiensis	18,041
trichoclada	16,371	umbellata	17,055	sicifolia	18,010
utriculosa	16,371	ferrea	17,028	baueri subsp. gouriquae	17,990
abietina subsp. atrorosea	16,355	sitiens	17,028	penduliflora	17,990
viscaria subsp. pustulata	16,355	toringbergensis	17,028	hanekomii	17,961
longiaristata	16,302	obtusata	17,017	milanjiana	17,942
viscaria subsp. viscaria	16,283	recurvata	16,990	amalophylla	17,928
fuscescens	16,276	longistyla	16,984	cubitans	17,928
inflaticalyx	16,276	oliveri	16,984	philippioides	17,928
quadrifida	16,276	tetrathecoides	16,984	jugicola	17,923
rosacea subsp. glabrata	16,276	remota	16,939	inaequalis	17,914
uberiflora	16,276	artemisioides	16,890	longimontana	17,914
vallis-fluminis	16,238	genistifolia	16,873	setulosa	17,904
regia subsp. mariae	16,197	humbertii	16,866	passerinoides	17,883
viscaria subsp. macrosepala	16,197	seriphiifolia	16,801	lusitanica	17,866
aspalathifolia	16,186	silvatica	16,778	subverticillaris	17,866
eustacei	16,186	erasmia	16,741	sparrmannii	17,835
lawsonii	16,159	leptantha	16,741	polycoma	17,818
unicolor subsp. georgensis	16,125	rhopalantha	16,741	rakotozafyana	17,816
opulenta	16,050	hexandra	16,712	viscaria subsp. pendula	17,802
greyi	16,012	goatcheriana var. petrensis	16,688	lateralis	17,794
transparens	15,932	monsoniana	16,688	velitaris	17,794
afra	15,902	galioides	16,674	verecunda	17,794
turneri	15,902	minutifolia	16,674	leucoclada	17,725
bakeri	15,868	parkeri	16,674	inconstans	17,672
costatisepala	15,868	perhispida	16,674	rosacea	17,666
pleiotricha	15,868	gigantea	16,669	rosacea subsp. rosacea	17,666
pleiotricha var. pleiotricha	15,868	vallis-gratiae	16,664	pulchelliflora	17,656
alexandri	15,867	banksii subsp. banksii	16,609	barnettiana	17,655
alexandri subsp. acockii	15,867	plukenetii subsp. breviflora	16,609	plena	17,587
alexandri subsp. alexandri	15,867	eriophoros	16,513	brachyphylla	17,586
cedromontana	15,853	eglandulosa	16,489	harveiana	17,512
limnophila	15,853	hillburttii	16,436	psittacina	17,506
nivalis	15,853	grandiflora subsp. grandiflora	16,426	argyraea	17,474
ovina	15,853	arcuata	16,416	aspalathoides	17,474
velutina	15,853	unilateralis	16,416	chionodes	17,474
glandulosa	15,835	abietina subsp. abietina	16,389	hippurus	17,474
glandulosa subsp. glandulosa	15,835	grandiflora subsp. perfoliosa	16,389	hirta	17,474
esterhuyseniae	15,829	latiflora	16,389	lerouxiae	17,474
johnstoniana	15,829	situshiemalis	16,389	planifolia	17,474
pannosa	15,829	xanthina	16,389	rudolfii	17,474
spinifera	15,825	macroloma	16,371	zwartbergensis	17,441
agglutinans	15,819	salax	16,371	maximilianii	17,387
eriocephala	15,819	schumannii	16,371	viridiflora subsp. viridiflora	17,387
paucifolia	15,819	trichoclada	16,371	bokkeveldia	17,330
paucifolia subsp. ciliata	15,819	utriculosa	16,371	junonia	17,316
paucifolia subsp. paucifolia	15,819	abietina subsp. atrorosea	16,355	junonia var. junonia	17,316
paucifolia subsp. squarrosa	15,819	viscaria subsp. pustulata	16,355	formosa	17,282
vernicosa	15,819	viscaria subsp. viscaria	16,283	leucodesmia	17,282
andevalensis	15,796	fuscescens	16,276	flanaganii	17,276
kirstenii	15,767	inflaticalyx	16,276	ribisaria	17,276
lithophila	15,767	quadrifida	16,276	aestiva	17,258
taylorii	15,767	rosacea subsp. glabrata	16,276	filialis	17,258
michellensis	15,743	uberiflora	16,276	gibbosa	17,258
kingaensis	15,735	vallis-fluminis	16,238	multiflexuosa	17,243
cuscutiformis	15,709	viscaria subsp. macrosepala	16,197	barbigeroides	17,241
borboniifolia	15,656	aspalathifolia	16,186	ericoides	17,241
saxigena	15,656	eustacei	16,186	petricola	17,216
simii	15,643	lawsonii	16,159	atricha	17,210
chiroptera	15,637	unicolor subsp. georgensis	16,125	phaeocarpa	17,197
lasiocarpa	15,637	opulenta	16,050	alticola	17,176
reenensis	15,637	greyi	16,012	mollis	17,171
schlechteri	15,637	transparens	15,932	burchelliana	17,120
stokoeanthus	15,637	afra	15,902	subcapitata	17,090
subimbricata	15,626	turneri	15,902	juniperina	17,055
inflata	15,623	bakeri	15,868	karwyderi	17,055
evansii	15,595	costatisepala	15,868	sonderiana	17,055
azaleifolia	15,593	pleiotricha	15,868	umbellata	17,055
placentiflora	15,593	pleiotricha var. pleiotricha	15,868	obtusata	17,017
cyathiformis	15,566	alexandri	15,867	cincta	17,002
dodii	15,566	alexandri subsp. acockii	15,867	recurvata	16,990
regerminans	15,566	alexandri subsp. alexandri	15,867	oliveri	16,984
coccinea	15,540	cedromontana	15,853	tetrathecoides	16,984
coccinea subsp. uniflora	15,540	limnophila	15,853	remota	16,939
cameronii	15,502	nivalis	15,853	fontana	16,925
cavartica	15,502	ovina	15,853	macowanii	16,925
sexfaria	15,482	velutina	15,853	macowanii subsp. macowanii	16,925

outeniquae	17,540	eugenea	18,250	cuscutiformis	19,244
notoporina	17,525	pumila	18,250	binaria	19,178
salicina	17,525	arachnocalyx	18,241	goudotiana	19,178
lanipes	17,512	petrophila	18,241	trimera	19,178
coccinea	17,502	viridiflora	18,230	trimera subsp. trimera	19,178
atromontana	17,500	adelopetala	18,157	atricha	19,169
fuscescens	17,500	nubigena	18,149	trichophylla	19,169
quadrifida	17,500	lateriflora	18,126	schelpeorum	19,166
filipendula subsp. parva	17,488	nervata	18,124	amalophylla	19,152
eremioides subsp. pubescens	17,462	baccans	18,120	philippioides	19,152
eglandulosa	17,455	blandfordii	18,120	brachycentra	19,140
innovans	17,428	rimarum	18,120	eriophoros	19,131
aspalathifolia	17,411	toringbergensis	18,120	setulosa	19,128
eustacei	17,411	perspicua	18,119	esterhuyseniae	19,098
caterviflora	17,382	perspicua subsp. perspicua	18,119	ostiaria	19,009
milanjiana	17,364	artemisioides	18,115	doliiformis	19,002
racemosa	17,364	plena	18,084	straussiana	18,988
cumuliflora	17,327	inordinata	18,037	extrusa	18,984
velatiflora	17,327	ustulescens	18,028	utriculosa	18,984
cygnea	17,287	adnata	17,943	orientalis	18,955
supranubia	17,287	longipedunculata	17,943	fontana	18,928
terniflora	17,279	calcicola	17,942	obliqua	18,914
macilenta	17,272	articularis	17,932	curtophylla	18,897
riparia	17,263	pellucida	17,903	eremioides subsp. eglandula	18,891
oblongiflora	17,248	ebracteata	17,868	viridiflora subsp. primulina	18,885
dissimulans	17,213	lepidota	17,868	gossypioides	18,810
hexensis	17,213	barbigeroides	17,796	rupicola	18,787
longistyla	17,213	glabella	17,781	physophylla	18,757
obconica	17,213	glabella subsp. glabella	17,781	globiceps	18,752
oliveri	17,213	glabella subsp. laevis	17,781	globiceps subsp. consors	18,752
umbelliflora	17,213	betsileana	17,766	globiceps subsp. gracilis	18,752
haemastoma	17,130	arenaria	17,670	kingaensis subsp. rugegensis	18,736
viscosissima	17,118	keetii	17,670	ventricosa	18,736
aristata	17,106	onusta	17,670	greyi	18,698
plumosa	17,080	vallis-gratiae	17,630	abietina subsp. atrorosea	18,629
puberuliflora	17,080	viscaria subsp. longifolia	17,613	alnea	18,606
blaerioides	17,078	anemodes	17,595	kougabergensis	18,602
blaerioides subsp. hirsuta	17,078	emarginata	17,595	fimbriata	18,506
oreotragus	17,078	keeromsbergensis	17,595	perplexa	18,499
pubigera	17,078	lachnaeifolia	17,595	dolfiana	18,482
senilis	17,056	pudens	17,595	setociliata	18,482
pannosa	17,054	salax	17,595	trimera subsp. elgonensis	18,442
baroniana	17,049	scytophylla	17,595	arborescens	18,440
xeranthemifolia	17,043	sperata	17,595	patersonii	18,407
x flavisepala	17,031	thodei	17,595	vallis-aranearum	18,407
passerina	17,029	bruniades	17,580	cabernetea	18,372
cunoniensis	17,000	marlothii	17,580	melanacme	18,372
kirstenii	16,991	suffulta	17,580	maritima	18,354
harveiana	16,934	subcapitata	17,557	macroloma	18,333
pleiotricha var. blaeriodes	16,934	barrydalensis	17,552	pleiotricha	18,325
flacca	16,925	notoporina	17,525	pleiotricha var. pleiotricha	18,325
afra	16,897	salicina	17,525	tragulifera	18,325
argyraea	16,897	lanipes	17,512	cereris	18,316
aspalathoides	16,897	coccinea	17,502	coronanthera	18,316
chionodes	16,897	atromontana	17,500	leptoclada	18,316
hirta	16,897	fuscescens	17,500	oligantha	18,316
lerouxiae	16,897	filipendula subsp. parva	17,488	coacervata	18,286
marifolia	16,897	garciae	17,481	desmantha	18,279
trimera subsp. keniensis	16,896	eremioides subsp. pubescens	17,462	hebdomadalis	18,279
multiflora	16,859	eglandulosa	17,455	oresbia	18,279
pogonanthera	16,851	innovans	17,428	petraea	18,279
melastoma	16,839	aspalathifolia	17,411	florifera	18,255
melastoma subsp. melastoma	16,839	eustacei	17,411	eugenea	18,250
melastoma subsp. minor	16,839	caterviflora	17,382	arachnocalyx	18,241
ecklonii	16,833	unicolor subsp. unicolor	17,371	petrophila	18,241
filipendula subsp. filipendula	16,833	milanjiana	17,364	kraussiana	18,240
nana	16,833	cumuliflora	17,327	viridiflora	18,230
lanuginosa	16,821	cygnea	17,287	alticola	18,184
evansii	16,819	supranubia	17,287	adelopetala	18,157
banksii subsp. comptonii	16,764	terniflora	17,279	nubigena	18,149
banksii subsp. purpurea	16,764	riparia	17,263	lateriflora	18,126
aneimena	16,709	oblongiflora	17,248	etheliae	18,125
filago	16,707	dissimulans	17,213	nervata	18,124
jonasiana	16,707	hexensis	17,213	baccans	18,120
formosa	16,704	longistyla	17,213	blandfordii	18,120
leucodesmia	16,704	oliveri	17,213	rimarum	18,120
miniscula	16,697	haemastoma	17,130	artemisioides	18,115
cubitans	16,695	aristata	17,106	plena	18,084
velitaris	16,688	plumosa	17,080	ustulescens	18,028
aestiva	16,680	blaerioides	17,078	lateralis	17,995
andevalensis	16,680	blaerioides subsp. hirsuta	17,078	adnata	17,943
scabriuscula	16,680	oreotragus	17,078	calcicola	17,942
microdonta	16,679	senilis	17,056	coccinea subsp. coccinea	17,902
parilis	16,671	pannosa	17,054	conspicua	17,899
dulcis	16,656	baroniana	17,049	tysonii	17,889
stylaris	16,642	xeranthemifolia	17,043	ebracteata	17,868
baueri subsp. gouriquae	16,605	x flavisepala	17,031	lepidota	17,868
ceraria	16,605	passerina	17,029	glabella	17,781
foliacea	16,605	cunoniensis	17,000	glabella subsp. glabella	17,781
foliacea subsp. foliacea	16,605	kirstenii	16,991	glabella subsp. laevis	17,781
foliacea subsp. fulgens	16,605	harveiana	16,934	betsileana	17,766
petrusiana	16,585	pleiotricha var. blaeriodes	16,934	arenaria	17,670
aristifolia	16,570	flacca	16,925	keetii	17,670
laevigata	16,570	argyraea	16,897	onusta	17,670
saptouensis	16,552	aspalathoides	16,897	vallis-gratiae	17,630
erinus	16,551	chionodes	16,897	anemodes	17,595
abietina subsp. abietina	16,549	hirta	16,897	emarginata	17,595
burchelliana	16,543	lerouxiae	16,897	keeromsbergensis	17,595
odorata	16,534	marifolia	16,897	lachnaeifolia	17,595
filiformis	16,477	trimera subsp. keniensis	16,896	pudens	17,595
involvens	16,477	multiflora	16,859	salax	17,595
penicilliformis	16,477	pogonanthera	16,851	scytophylla	17,595
sonderiana	16,477	melastoma	16,839	sperata	17,595
leucotrachela	16,475	melastoma subsp. melastoma	16,839	thodei	17,595
leucotrachela subsp. leucotrachela	16,475	melastoma subsp. minor	16,839	bruniades	17,580
rosacea	16,434	ecklonii	16,833	marlothii	17,580
rosacea subsp. rosacea	16,434	filipendula subsp. filipendula	16,833	suffulta	17,580
transparens	16,429	nana	16,833	sicifolia	17,556
densifolia	16,378	lanuginosa	16,821	salicina	17,525
opulenta	16,279	evansii	16,819	lanipes	17,512
uysii	16,279	banksii subsp. comptonii	16,764	atromontana	17,500
zebrensis	16,279	banksii subsp. purpurea	16,764	fuscescens	17,500
platycodon	16,247	aneimena	16,709	filipendula subsp. parva	17,488
platycodon subsp. platycodon	16,247	filago	16,707	garciae	17,481
atropurpurea	16,241	jonasiana	16,707	cristata	17,473
hippurus	16,241	formosa	16,704	eremioides subsp. pubescens	17,462
oophylla	16,241	velitaris	16,688	inaequalis	17,461
patens	16,241	aestiva	16,680	eglandulosa	17,455
planifolia	16,241	andevalensis	16,680	innovans	17,428
leucoclada	16,234	scabriuscula	16,680	eustacei	17,411
bruniifolia	16,205	microdonta	16,679	milanjiana	17,364
glumiflora	16,205	parilis	16,671	sphaerocephala	17,364
jananthus	16,205	dulcis	16,656	cumuliflora	17,327
recurvifolia	16,167	baueri subsp. gouriquae	16,605	cygnea	17,287
viridiflora subsp. viridiflora	16,155	ceraria	16,605	terniflora	17,279
dispar	16,136	foliacea	16,605	riparia	17,263
piquetbergensis	16,136	foliacea subsp. foliacea	16,605	inflata	17,256
galioides	16,097	foliacea subsp. fulgens	16,605	hexensis	17,213
bodkinii	16,083	petrusiana	16,585	oliveri	17,213
sociorum	16,080	aristifolia	16,570	malmesburiensis	17,202
oresigena	16,049	laevigata	16,570	pulchelliflora	17,202
filialis	16,025	saptouensis	16,552	aristata	17,106
hameriana	16,010	erinus	16,551	plumosa	17,080
cryptoclada	15,983	abietina subsp. abietina	16,549	blaerioides	17,078
notholeeana	15,983	odorata	16,534	blaerioides subsp. hirsuta	17,078
altevivens	15,975	filiformis	16,477	senilis	17,056
cymosa subsp. cymosa	15,975	involvens	16,477	pannosa	17,054
oraria	15,975	sonderiana	16,477	baroniana	17,049
syngenesia	15,975	leucotrachela	16,475	xeranthemifolia	17,043
grata	15,868	leucotrachela subsp. leucotrachela	16,475	x flavisepala	17,031
humansdorpensis	15,868	transparens	16,429	passerina	17,029
mira	15,868	discolor variant A 'discolor'	16,300	regia subsp. mariae	17,008
parviflora	15,868	opulenta	16,279	cunoniensis	17,000
dianthifolia	15,867	uysii	16,279	kirstenii	16,991
penduliflora	15,867	zebrensis	16,279	harveiana	16,934
blaerioides subsp. blaerioides	15,842	platycodon	16,247	pleiotricha var. blaeriodes	16,934
arcuata	15,839	platycodon subsp. platycodon	16,247	valida	16,928
unilateralis	15,839	hippurus	16,241	argyraea	16,897
juniperina	15,822	patens	16,241	aspalathoides	16,897
karwyderi	15,822	planifolia	16,241	chionodes	16,897
umbellata	15,822	leucoclada	16,234	hirta	16,897
intervallaris	15,798	bruniifolia	16,205	lerouxiae	16,897
reenensis	15,798	glumiflora	16,205	trimera subsp. keniensis	16,896
orthiocola	15,784	jananthus	16,205	multiflora	16,859
cyathiformis	15,723	recurvifolia	16,167	pogonanthera	16,851
sitiens	15,723	viridiflora subsp. viridiflora	16,155	melastoma subsp. minor	16,839

leptoclada	14,391	ustulescens	15,330	physantha	16,760
macrophylla	14,391	banksii subsp. banksii	15,245	plumosa	16,760
maritima	14,391	sociorum	15,187	priorii	16,760
monantha	14,391	viridiflora subsp. viridiflora	15,187	simulans	16,760
newdigateae	14,391	boucheri	15,177	viscosissima	16,760
ocellata	14,391	erinus	15,177	dregei	16,755
oligantha	14,391	malmesburiensis	15,177	steinbergiana	16,729
petrophila	14,391	notholeeana	15,177	axilliflora	16,718
poculiflora	14,391	procaviana	15,177	cedromontana	16,706
puberuliflora	14,391	pulchelliflora	15,177	formosa	16,706
rhodella	14,391	radicans subsp. schlechteri	15,177	nivalis	16,706
sparsa	14,391	gracilipes	15,174	ovina	16,706
tradouwensis	14,391	guthriei	15,174	straussiana	16,706
diaphana	14,364	parilis	15,116	anemodes	16,702
glandulosa	14,364	acuta	15,081	argentea	16,702
glandulosa subsp. glandulosa	14,364	altevivens	15,081	deflexa	16,702
atromontana	14,332	goatcheriana	15,081	emarginata	16,702
bruniifolia	14,332	oraria	15,081	extrusa	16,702
diosmifolia	14,332	intervallaris	15,061	lachnaeifolia	16,702
jananthus	14,332	purgatoriensis	15,061	scytophylla	16,702
keeromsbergensis	14,332	irregularis	15,057	trichoclada	16,702
nigrimontana	14,332	propinqua	15,057	involucrata	16,682
pudens	14,332	virginalis	15,057	turgida	16,682
schumannii	14,332	curvifolia	15,008	eglandulosa	16,671
sperata	14,332	jonasiana	14,958	muscosa	16,671
thamnoides	14,332	nutans	14,958	octonaria	16,671
wildii	14,332	obtusata	14,958	globulifera	16,660
perrieri	14,297	subimbricata	14,958	gracilis	16,660
venustiflora	14,232	cinerea	14,900	sitiens	16,660
venustiflora subsp. glandulosa	14,232	melastoma	14,892	baueri	16,613
venustiflora subsp. venustiflora	14,232	melastoma subsp. melastoma	14,892	baueri subsp. baueri	16,613
jasminiflora	14,226	melastoma subsp. minor	14,892	baueri subsp. gouriquae	16,613
platycodon	14,121	rupicola	14,786	sacciflora	16,613
platycodon subsp. platycodon	14,121	barbigeroides	14,762	australis	16,551
labialis	14,086	dispar	14,762	transparens	16,465
fastigiata	13,996	hansfordii	14,762	marojejyensis	16,460
plena	13,996	ioniana	14,762	caledonica	16,457
flavicoma	13,848	piquetbergensis	14,762	crateriformis	16,407
leptantha	13,848	brachyphylla	14,693	eriocephala	16,407
oliveranthus	13,848	caprina	14,686	glabella subsp. glabella	16,407
haemastoma	13,839	greyi	14,686	juniperina	16,407
halicacaba	13,839	keetii	14,686	karwyderi	16,407
margaritacea	13,824	kingaensis	14,686	obconica	16,407
pulvinata	13,824	kingaensis subsp. rugegensis	14,686	pilaarkopensis	16,407
aristata	13,815	sicifolia	14,686	radicans	16,407
conspicua subsp. conspicua	13,815	accommodata	14,605	radicans subsp. radicans	16,407
curviflora	13,815	alnea	14,605	globiceps subsp. globiceps	16,382
hibbertii	13,815	genistifolia	14,605	pilulifera	16,327
infundibuliformis	13,815	involvens	14,605	ardens	16,221
nevillei	13,815	stokoeanthus	14,605	monadelphia	16,166
pellucida	13,815	galpinii	14,597	notoporina	16,092
pinea	13,815	comata	14,391	physodes	16,092
praenitens	13,815	cordata	14,391	lageniformis	16,028
unicolor	13,815	dysantha	14,391	cylindrica	15,986
unicolor subsp. mutica	13,815	flacca	14,391	velatiflora	15,969
drakensbergensis	13,805	klotzschii	14,391	saxigena	15,939
lecomtei	13,728	lavandulifolia	14,391	verecunda	15,911
amatolensis	13,670	leptoclada	14,391	x flavisepala	15,905
abietina	13,600	macrophylla	14,391	conferta	15,899
brachialis	13,600	maritima	14,391	reunionensis	15,745
conspicua subsp. roseoflora	13,600	monantha	14,391	wyliei	15,720
latiflora	13,600	newdigateae	14,391	discolor variant A 'discolor'	15,694
nematophylla	13,600	ocellata	14,391	inclusa	15,640
petrusiana	13,600	oligantha	14,391	amoena	15,630
monsoniana	13,566	petrophila	14,391	conspicua	15,616
evansii	13,556	poculiflora	14,391	glaphyra	15,606
boutonii	13,532	puberuliflora	14,391	hirta	15,539
globiceps	13,532	rhodella	14,391	filipendula subsp. parva	15,532
globiceps subsp. consors	13,532	sparsa	14,391	cryptoclada	15,530
globiceps subsp. gracilis	13,532	tradouwensis	14,391	foliacea	15,380
interrupta	13,532	glandulosa	14,364	foliacea subsp. foliacea	15,380
similis	13,532	glandulosa subsp. glandulosa	14,364	foliacea subsp. fulgens	15,380
bakeri	13,453	bruniifolia	14,332	ecklonii	15,371
sagittata	13,453	diosmifolia	14,332	ustulescens	15,330
viscidiflora	13,453	keeromsbergensis	14,332	bibax	15,203
cameronii	13,443	nigrimontana	14,332	kogelbergensis	15,203
gigantea	13,436	pudens	14,332	regia subsp. mariae	15,203
obliqua	13,436	schumannii	14,332	viscaria subsp. pendula	15,203
cruenta	13,385	sperata	14,332	sociorum	15,187
discolor variant B 'speciosa'	13,385	thamnoides	14,332	viridiflora subsp. viridiflora	15,187
maderensis	13,385	wildii	14,332	boucheri	15,177
cumuliflora	13,314	perrieri	14,297	erinus	15,177
rehmii	13,314	venustiflora	14,232	malmesburiensis	15,177
riparia	13,300	venustiflora subsp. glandulosa	14,232	pulchelliflora	15,177
chamissonis	13,295	venustiflora subsp. venustiflora	14,232	gracilipes	15,174
leucopelta	13,295	jasminiflora	14,226	guthriei	15,174
aristifolia	13,279	platycodon	14,121	colorans	15,144
lycopodiastrum	13,279	platycodon subsp. platycodon	14,121	parilis	15,116
palliiflora	13,279	labialis	14,086	acuta	15,081
triflora	13,279	fastigiata	13,996	altevivens	15,081
heleophila	13,271	plena	13,996	goatcheriana	15,081
macilenta	13,271	flavicoma	13,848	intervallaris	15,061
odorata	13,271	leptantha	13,848	purgatoriensis	15,061
andevalensis	13,255	haemastoma	13,839	irregularis	15,057
lateralis	13,255	halicacaba	13,839	propinqua	15,057
recta	13,237	margaritacea	13,824	virginalis	15,057
tysonii	13,237	pulvinata	13,824	curvifolia	15,008
recurvata	13,170	aristata	13,815	jonasiana	14,958
altiphila	13,161	conspicua subsp. conspicua	13,815	nutans	14,958
dracomontana	13,161	hibbertii	13,815	obtusata	14,958
elsieana	13,161	infundibuliformis	13,815	subimbricata	14,958
esteriana	13,161	nevillei	13,815	cinerea	14,900
esteriana subsp. esteriana	13,161	pellucida	13,815	melastoma	14,892
jumellei	13,161	pinea	13,815	melastoma subsp. melastoma	14,892
madida	13,161	praenitens	13,815	melastoma subsp. minor	14,892
rigidula	13,161	unicolor	13,815	rupicola	14,786
urceolata	13,161	unicolor subsp. mutica	13,815	barbigeroides	14,762
amicorum	13,158	drakensbergensis	13,805	ioniana	14,762
corydalis	13,099	lecomtei	13,728	piquetbergensis	14,762
ciliaris	13,090	amatolensis	13,670	brachyphylla	14,693
viridiflora subsp. primulina	13,090	abietina	13,600	caprina	14,686
modesta	13,065	brachialis	13,600	keetii	14,686
pubigera	13,065	conspicua subsp. roseoflora	13,600	kingaensis	14,686
saxicola	13,065	latiflora	13,600	kingaensis subsp. rugegensis	14,686
phillipsii	13,041	nematophylla	13,600	aspalathifolia	14,625
williamsiorum	13,002	petrusiana	13,600	accommodata	14,605
ericoides	12,960	evansii	13,556	alnea	14,605
pleiotricha var. pleiotricha	12,884	boutonii	13,532	genistifolia	14,605
amalophylla	12,770	globiceps	13,532	stokoeanthus	14,605
cubitans	12,770	globiceps subsp. consors	13,532	abietina subsp. abietina	14,454
eriocodon	12,770	globiceps subsp. gracilis	13,532	grandiflora subsp. perfoliosa	14,454
limosa	12,770	interrupta	13,532	urna-viridis	14,419
nervata	12,770	similis	13,532	unicolor subsp. unicolor	14,395
oxycoccifolia	12,770	bakeri	13,453	comata	14,391
philippioides	12,770	sagittata	13,453	cordata	14,391
roseoloba	12,770	viscidiflora	13,453	dysantha	14,391
salteri	12,770	cameronii	13,443	flacca	14,391
totta	12,770	gigantea	13,436	klotzschii	14,391
zitzikammensis	12,770	obliqua	13,436	lavandulifolia	14,391
lananthera	12,763	cruenta	13,385	leptoclada	14,391
walkeri	12,763	maderensis	13,385	monantha	14,391
aneimena	12,746	cumuliflora	13,314	newdigateae	14,391
schelpeorum	12,746	rehmii	13,314	ocellata	14,391
setulosa	12,746	riparia	13,300	oligantha	14,391
glandulosa subsp. fourcadei	12,719	chamissonis	13,295	petrophila	14,391
cymosa	12,711	aristifolia	13,279	poculiflora	14,391
cymosa subsp. cymosa	12,711	lycopodiastrum	13,279	puberuliflora	14,391
intricata	12,711	palliiflora	13,279	rhodella	14,391
melanacme	12,711	triflora	13,279	sparsa	14,391
oresigena	12,711	heleophila	13,271	bruniifolia	14,332
syngenesia	12,711	macilenta	13,271	diosmifolia	14,332
trichophylla	12,711	odorata	13,271	keeromsbergensis	14,332
truncata	12,711	andevalensis	13,255	nigrimontana	14,332
occulta	12,687	lateralis	13,255	pudens	14,332
russakiana	12,687	tysonii	13,237	schumannii	14,332
angulosa	12,589	recurvata	13,170	thamnoides	14,332
bruniades	12,589	altiphila	13,161	wildii	14,332
capitata	12,589	dracomontana	13,161	perrieri	14,297
crassisepala	12,589	elsieana	13,161	chionophila	14,290
filago	12,589	esteriana subsp. esteriana	13,161	venustiflora	14,232
longipedunculata	12,589	jumellei	13,161	venustiflora subsp. glandulosa	14,232
lowryensis	12,589	amicorum	13,158	venustiflora subsp. venustiflora	14,232
permutata	12,589	corydalis	13,099	jasminiflora	14,226
petiolaris	12,589	ciliaris	13,090	platycodon	14,121
saptouensis	12,589	modesta	13,065	platycodon subsp. platycodon	14,121
rivularis	12,530	pubigera	13,065	labialis	14,086

labialis	13,880	goatcheriana var. drakensteinensis	14,656	ampullacea	15,827	erasmia	12,501	esteriana subsp. swartbergensis	13,141	albospicata	14,243	irbyana	15,468	glandulosa	15,835	intonsa	16,891	phaeocarpa	15,706	piquetbergensis	16,136	ecklonii	16,833	spiculifolia	12,447	saxicola	13,065	canescens	14,076
cinerea	13,872	hexandra	14,637	oxycoccifolia	15,827	glauca	12,501	perplexa	13,141	pinea	14,224	stylaris	15,418	glandulosa subsp. glandulosa	15,835	oxycoccifolia	16,891	perspicua subsp. latifolia	15,662	galioides	16,097	filipendula subsp. filipendula	16,833	viscaria subsp. pustulata	12,367	phillipsii	13,041	schlechteri	14,076
umbelliflora	13,872	chiroptera	14,629	curtophylla	15,812	glauca var. glauca	12,501	chiroptera	13,135	russakiana	14,209	azorica	15,411	esterhuyseniae	15,829	pinea	16,891	armandiana	15,634	bodkinii	16,083	nana	16,833	albescens	12,316	williamsiorum	13,002	fastigiata	13,996
cumuliflora	13,861	collina	14,601	carnea	15,770	junonia	12,501	manipuliflora	13,106	hebeclada	14,158	omninoglabra	15,411	johnstoniana	15,829	artemisioides	16,890	venustiflora	15,606	sociorum	16,080	ingeana	16,822	atropurpurea	12,316	ericoides	12,960	plena	13,996
garciae	13,857	intervallaris	14,601	glauca	15,762	junonia var. minor	12,501	vagans	13,106	parkeri	14,158	scoparia	15,411	pannosa	15,829	genistifolia	16,873	venustiflora subsp. glandulosa	15,606	oresigena	16,049	lanuginosa	16,821	cederbergensis	12,316	pleiotricha var. pleiotricha	12,884	hirtiflora	13,918
glandulifera	13,857	evansii	14,586	glauca var. glauca	15,762	rhopalantha	12,501	filipendula subsp. parva	13,083	perhispida	14,158	umbelliflora	15,411	spinifera	15,825	humbertii	16,866	venustiflora subsp. venustiflora	15,606	filialis	16,025	banksii subsp. comptonii	16,764	copiosa	12,316	amalophylla	12,770	penduliflora	13,911
hirtiflora	13,857	colorans	14,583	heleogena	15,755	umbelliflora	12,501	ioniana	13,063	ebracteata	14,142	daphniflora	15,358	eriocephala	15,819	bracteolaris	16,858	tubercularis	15,592	hameriana	16,010	banksii subsp. purpurea	16,764	curvistyla	12,316	cubitans	12,770	scabriuscula	13,894
holosericea	13,857	maderi	14,583	irrorata	15,745	cremea	12,497	thunbergii	13,031	lepidota	14,142	shannonii	15,358	paucifolia	15,819	thamnoides	16,811	beatricis	15,576	cryptoclada	15,983	aneimena	16,709	distorta	12,316	eriocodon	12,770	flavicoma	13,848
modesta	13,857	ventricosa	14,583	regia subsp. mariae	15,743	infundibuliformis	12,497	jananthus	12,981	obtusata	14,142	inclusa	15,310	paucifolia subsp. ciliata	15,819	seriphiifolia	16,801	massonii	15,485	notholeeana	15,983	filago	16,707	eremioides	12,316	limosa	12,770	leptantha	13,848
straussiana	13,857	prolata	14,575	albospicata	15,741	leucotrachela	12,497	krugeri	12,981	viridiflora subsp. primulina	14,142	nabea	15,310	paucifolia subsp. paucifolia	15,819	silvatica	16,778	squarrosa	15,485	altevivens	15,975	jonasiana	16,707	eremioides subsp. eglandula	12,316	oxycoccifolia	12,770	halicacaba	13,839
alfredii	13,835	notoporina	14,526	cryptoclada	15,741	leucotrachela subsp. leucotrachela	12,497	plukenetii subsp. lineata	12,981	melanomontana	14,088	odorata	15,310	paucifolia subsp. squarrosa	15,819	erasmia	16,741	bojeri	15,441	cymosa subsp. cymosa	15,975	formosa	16,704	eremioides subsp. eremioides	12,316	philippioides	12,770	margaritacea	13,824
chrysocodon	13,834	physodes	14,526	rakotozafyana	15,741	retorta	12,497	glaphyra	12,978	monadelphia	14,068	conspicua subsp. conspicua	15,286	andevalensis	15,796	cygnea	16,732	minutifolia	15,441	oraria	15,975	aestiva	16,680	eremioides subsp. pubescens	12,316	salteri	12,770	pulvinata	13,824
aristifolia	13,824	salicina	14,526	montis-hominis	15,671	barnettiana	12,445	hillburttii	12,947	gracilipes	14,023	cremea	15,286	kirstenii	15,767	hexandra	16,712	bolusiae	15,433	humansdorpensis	15,868	andevalensis	16,680	fuscescens	12,316	totta	12,770	aristata	13,815
cabernetea	13,824	sitiens	14,526	retorta	15,668	brachialis	12,428	passerinoides	12,947	kougabergensis	14,023	croceovirens	15,286	lithophila	15,767	goatcheriana var. petrensis	16,688	flanaganii	15,433	mira	15,868	microdonta	16,679	hippurus	12,316	zitzikammensis	12,770	conspicua subsp. conspicua	13,815
laevigata	13,824	canaliculata	14,477	benguelensis	15,665	filipendula subsp. filipendula	12,428	exleeana	12,912	uysii	14,023	hibbertii	15,286	taylorii	15,767	tetragona	16,675	dominans	15,411	parviflora	15,868	dulcis	16,656	inaequalis	12,316	lananthera	12,763	hibbertii	13,815
embothriifolia	13,819	humifusa	14,477	benguelensis var. albescens	15,665	propinqua	12,428	petricola	12,912	borboniifolia	13,923	oblongiflora	15,286	michellensis	15,743	galioides	16,674	insolitanthera	15,404	dianthifolia	15,867	diaphana	16,647	inflaticalyx	12,316	walkeri	12,763	infundibuliformis	13,815
maderensis	13,819	banksii subsp. purpurea	14,460	benguelensis var. benguelensis	15,665	irrorata	12,382	intermedia subsp. albiflora	12,908	bokkeveldia	13,841	quadrisulcata	15,286	kingaensis	15,735	minutifolia	16,674	cedromontana	15,276	penduliflora	15,867	baueri subsp. gouriquae	16,605	lateriflora	12,316	aneimena	12,746	nevillei	13,815
savileae	13,771	jasminiflora	14,460	galioides	15,665	kingaensis	12,331	eburnea	12,852	caledonica	13,841	unicolor subsp. mutica	15,286	cuscutiformis	15,709	parkeri	16,674	nivalis	15,276	blaerioides subsp. blaerioides	15,842	ceraria	16,605	longimontana	12,316	glandulosa subsp. fourcadei	12,719	pellucida	13,815
savileae var. grandiflora	13,771	lageniformis	14,460	lanceolifera	15,619	aristifolia	12,271	florifera	12,852	cuscutiformis	13,841	benguelensis var. albescens	15,283	borboniifolia	15,656	perhispida	16,674	ovina	15,276	arcuata	15,839	foliacea	16,605	magistrati	12,316	cymosa	12,711	pinea	13,815
savileae var. mutica	13,771	amatolensis	14,441	trichostigma	15,596	cruenta	12,271	rosacea subsp. glabrata	12,787	goatcheriana var. petrensis	13,841	afrorum	15,276	saxigena	15,656	gigantea	16,669	esterhuyseniae	15,252	unilateralis	15,839	foliacea subsp. foliacea	16,605	onusta	12,316	cymosa subsp. cymosa	12,711	praenitens	13,815
savileae var. savileae	13,771	parilis	14,438	pulchelliflora	15,526	haemastoma	12,271	thamnoides	12,787	inamoena	13,841	drakensbergensis	15,276	simii	15,643	vallis-gratiae	16,664	reunionensis	15,247	karwyderi	15,822	foliacea subsp. fulgens	16,605	patens	12,316	intricata	12,711	lecomtei	13,728
notholeeana	13,750	chonantha	14,403	subcapitata	15,496	taxifolia	12,271	hexandra	12,767	nemorosa	13,841	tysonii	15,276	chiroptera	15,637	botryoides	16,619	agglutinans	15,241	umbellata	15,822	petrusiana	16,585	planifolia	12,316	melanacme	12,711	massonii	13,616
spinifera	13,750	oresbia	14,403	eglandulosa	15,480	binaria	12,257	accommodata	12,751	filialis	13,769	hansfordii	15,228	lasiocarpa	15,637	banksii subsp. banksii	16,609	glomiflora	15,189	intervallaris	15,798	brownii	16,577	pubescens	12,316	oresigena	12,711	brachialis	13,600
curviflora	13,747	limosa	14,388	botryoides	15,478	madida	12,257	filiformis	12,751	irregularis	13,769	hebeclada	15,159	reenensis	15,637	plukenetii subsp. breviflora	16,609	mauritiensis	15,188	reenensis	15,798	globiceps subsp. globiceps	16,577	quadrifida	12,316	trichophylla	12,711	conspicua subsp. roseoflora	13,600
fascicularis	13,747	abietina	14,352	excavata	15,392	notholeeana	12,257	involvens	12,751	saxigena	13,769	danguyana	15,139	schlechteri	15,637	eriophoros	16,513	euryphylla	15,180	orthiocola	15,784	aristifolia	16,570	recurvifolia	12,316	truncata	12,711	latiflora	13,600
fascicularis var. fascicularis	13,747	grandiflora	14,352	junonia	15,369	botryoides	12,221	sonderiana	12,751	schlechteri	13,769	amicorum	15,123	stokoeanthus	15,637	eglandulosa	16,489	abietina subsp. diabolis	15,156	cyathiformis	15,723	michellensis	16,554	rudolfii	12,316	occulta	12,687	nematophylla	13,600
unicolor	13,747	grandiflora subsp. grandiflora	14,352	junonia var. junonia	15,369	brachycentra	12,221	subimbricata	12,751	swaziensis	13,769	tenuifolia	15,096	subimbricata	15,626	cruenta	16,461	doliiformis	15,156	sitiens	15,723	saptouensis	16,552	tarantulae	12,316	russakiana	12,687	petrusiana	13,600
unicolor subsp. unicolor	13,747	adelopetala	14,350	cristata	15,354	permutata	12,221	denticulata	12,727	didymocarpa	13,752	lanceolifera	15,083	inflata	15,623	hillburttii	16,436	latiflora	15,156	phaeocarpa	15,706	abietina subsp. abietina	16,549	tegetiformis	12,316	angulosa	12,589	boutonii	13,532
eriocephala	13,744	shannonii	14,350	haemastoma	15,321	cernua	12,196	plena	12,727	simii	13,752	annalis	14,998	evansii	15,595	unilateralis	16,416	straussiana	15,142	odorata	15,662	perspicua subsp. latifolia	16,534	thimifolia	12,316	bruniades	12,589	globiceps subsp. consors	13,532
paucifolia	13,744	diaphana	14,296	haematosiphon	15,297	columnaris	12,196	opulenta	12,682	embothriifolia	13,716	brachycentra	14,980	azaleifolia	15,593	hirtiflora	16,389	trichroma	15,142	armandiana	15,634	pectinifolia	16,488	trachysantha	12,316	capitata	12,589	bakeri	13,453
paucifolia subsp. ciliata	13,744	discolor variant C 'hebecalyx'	14,296	varderi	15,294	ferrea	12,196	subcapitata	12,611	haematosiphon	13,694	sicifolia	14,942	cyathiformis	15,566	latiflora	16,389	extrusa	15,138	venustiflora	15,606	sonderiana	16,477	turneri	12,316	crassisepala	12,589	sagittata	13,453
paucifolia subsp. paucifolia	13,744	cremea	14,277	polycoma	15,286	flexistyla	12,196	genistifolia	12,570	inflata	13,694	corydalis	14,909	dodii	15,566	situshiemalis	16,389	nigrimontana	15,138	venustiflora subsp. glandulosa	15,606	leucotrachela	16,475	umbratica	12,316	filago	12,589	viscidiflora	13,453
paucifolia subsp. squarrosa	13,744	leucoclada	14,259	selaginifolia	15,286	glabripes	12,196	perspicua	12,570	silvatica	13,671	scytophylla	14,909	cameronii	15,502	straussiana	16,389	schumannii	15,138	venustiflora subsp. venustiflora	15,606	leucotrachela subsp. leucotrachel	16,475	uysii	12,316	longipedunculata	12,589	cameronii	13,443
vernicosa	13,744	axilliflora	14,246	truncata	15,286	laeta	12,196	perspicua subsp. perspicua	12,570	alfredii	13,641	cumuliflora	14,870	cavartica	15,502	xanthina	16,389	trichoclada	15,138	tubercularis	15,592	brachialis	16,471	vallis-fluminis	12,316	lowryensis	12,589	gigantea	13,436
viscaria subsp. pendula	13,726	brachyphylla	14,120	algida	15,270	nana	12,196	rubiginosa	12,570	adnata	13,612	amidae	14,856	sexfaria	15,482	macroloma	16,371	utriculosa	15,138	beatricis	15,576	lasiocarpa	16,449	zebrensis	12,316	permutata	12,589	maderensis	13,385
intricata	13,714	borboniifolia	14,117	rhodopis	15,264	occulta	12,196	shannonii	12,570	dominans	13,612	excavata	14,856	irbyana	15,468	salax	16,371	wildii	15,138	massonii	15,485	permutata	16,438	filipendula	12,309	petiolaris	12,589	cumuliflora	13,314
physophylla	13,714	cameronii	13,963	rubens	15,264	patersonii	12,196	diaphana	12,516	excavata	13,612	jugicola	14,856	stylaris	15,418	schumannii	16,371	orientalis	15,109	squarrosa	15,485	thamnoides	16,358	filipendula subsp. filipendula	12,309	saptouensis	12,589	riparia	13,300
chionophila	13,641	rufescens	13,963	irregularis	15,262	pilulifera	12,196	discolor variant C 'hebecalyx'	12,516	humansdorpensis	13,612	sociorum	14,856	azorica	15,411	trichoclada	16,371	recta	15,109	bojeri	15,441	cameronii	16,314	nana	12,309	rivularis	12,530	chamissonis	13,295
clavisepala	13,641	wendlandiana	13,963	fastigiata	15,225	vallis-aranearum	12,196	astroites	12,501	lanuginosa	13,612	inaequalis	14,847	omninoglabra	15,411	ixanthera	16,356	fontana	15,082	minutifolia	15,441	opulenta	16,279	cubica	12,299	spiculifolia	12,447	lycopodiastrum	13,279
goatcheriana var. goatcheriana	13,641	labialis	13,880	haematocodon	15,225	virginalis	12,196	erasmia	12,501	maximilianii	13,612	longimontana	14,847	scoparia	15,411	pectinifolia	16,286	cernua	15,068	bolusiae	15,433	uysii	16,279	viguieri	12,213	viscaria subsp. pustulata	12,367	palliflora	13,279
mallotocalyx	13,641	cinerea	13,872	urna-viridis	15,211	longistyla	12,146	glauca	12,501	plumigera	13,612	passerinoides	14,816	umbelliflora	15,411	unicolor subsp. unicolor	16,278	flexistyla	15,068	flanaganii	15,433	zebrensis	16,279	dodii	12,203	albescens	12,316	triflora	13,279
mauritanica	13,641	cumuliflora	13,861	obliqua	15,191	oliveri	12,146	glauca var. glauca	12,501	selaginifolia	13,612	astroites	14,807	daphniflora	15,358	fuscescens	16,276	obliqua	15,068	dominans	15,411	platycodon	16,247	annectens	12,194	atropurpurea	12,316	heleophila	13,271
longistyla	13,518	glandulifera	13,857	simii	15,190	priorii	12,146	junonia	12,501	wendlandiana	13,612	lusitanica	14,799	shannonii	15,358	inflaticalyx	16,276	subimbricata	15,049	insolitanthera	15,404	platycodon subsp. platycodon	16,247	retorta	12,194	cederbergensis	12,316	macilenta	13,271
oliveri	13,518	hirtiflora	13,857	viscaria subsp. macrosepala	15,188	coccinea	12,096	junonia var. minor	12,501	hermani	13,591	subverticillaris	14,799	inclusa	15,310	quadrifida	16,276	inflaticalyx	15,043	cedromontana	15,276	hippurus	16,241	albospicata	12,184	copiosa	12,316	odorata	13,271
placentiflora	13,518	holosericea	13,857	tragomontana	15,168	coccinea subsp. uniflora	12,096	rhopalantha	12,501	unilateralis	13,583	sparrmannii	14,768	nabea	15,310	rosacea subsp. glabrata	16,276	rosacea subsp. glabrata	15,043	ovina	15,276	patens	16,241	articularis	12,184	curvistyla	12,316	andevalensis	13,255
ciliaris	13,465	modesta	13,857	brownii	15,159	intermedia	12,096	cremea	12,497	zeyheriana	13,582	melanacme	14,750	odorata	15,310	physophylla	16,245	vallis-fluminis	15,005	esterhuyseniae	15,252	planifolia	16,241	brownleeae	12,160	distorta	12,316	lateralis	13,255
coccinea	13,465	straussiana	13,857	glutinosa	15,138	intermedia subsp. intermedia	12,096	leucotrachela	12,497	kingaensis subsp. rugegensis	13,567	polycoma	14,750	conspicua subsp. conspicua	15,286	vallis-fluminis	16,238	forsteri	14,986	reunionensis	15,247	leucoclada	16,234	galgebergensis	12,152	eremioides	12,316	recurvata	13,170
coccinea subsp. uniflora	13,465	alfredii	13,835	supranubia	15,138	karwyderi	12,096	leucotrachela subsp. leucotrachela	12,497	blancheana	13,487	syngenesia	14,750	cremea	15,286	eustacei	16,186	kingaensis	14,968	agglutinans	15,241	bruniifolia	16,205	densata	12,108	eremioides subsp. eglandula	12,316	altiphila	13,161
versicolor	13,465	chrysocodon	13,834	lateriflora	15,127	latifolia	12,096	retorta	12,497	hexensis	13,487	viscaria subsp. pendula	14,735	croceovirens	15,286	caterviflora	16,173	gossypioides	14,964	mauritiensis	15,188	glumiflora	16,205	ibityensis	12,084	eremioides subsp. eremioides	12,316	elsieana	13,161
transparens	13,431	aristifolia	13,824	barbigeroides	15,111	maritima	12,096	barnettiana	12,445	irbyana	13,487	irregularis	14,726	hibbertii	15,286	lawsonii	16,159	globulifera	14,925	abietina subsp. diabolis	15,156	jananthus	16,205	laevigata	12,046	eremioides subsp. pubescens	12,316	esteriana subsp. esteriana	13,161
miniscula	13,426	cabernetea	13,824	ferrea	15,081	melastoma	12,096	brachialis	12,428	macrotrema	13,487	lateralis	14,726	quadrisulcata	15,286	unicolor subsp. georgensis	16,125	physophylla	14,911	doliiformis	15,156	recurvifolia	16,167	perplexa	12,041	fuscescens	12,316	jumellei	13,161
obconica	13,408	laevigata	13,824	toringbergensis	15,081	melastoma subsp. melastoma	12,096	filipendula subsp. filipendula	12,428	nervata	13,487	tenella	14,726	unicolor subsp. mutica	15,286	zeyheriana	16,084	globiceps	14,906	latiflora	15,156	viridiflora subsp. viridiflora	16,155	ampullacea	12,014	hippurus	12,316	amicorum	13,158
humbertii	13,401	embothriifolia	13,819	irbyana	15,015	melastoma subsp. minor	12,096	propinqua	12,428	orthiocola	13,487	velitaris	14,726	benguelensis var. albescens	15,283	opulenta	16,050	globiceps subsp. consors	14,906	straussiana	15,142	piquetbergensis	16,136	intonsa	12,014	inaequalis	12,316	corydalis	13,099
zeyheriana	13,399	maderensis	13,819	alnea	14,980	physantha	12,096	irrorata	12,382	pilaarkopensis	13,487	verecunda	14,726	afrorum	15,276	frigida	16,037	globiceps subsp. gracilis	14,906	trichroma	15,142	conspicua subsp. conspicua	16,097	viscaria subsp. macrosepala	11,980	inflaticalyx	12,316	ciliaris	13,090
tubercularis	13,359	savileae	13,771	karwyderi	14,980	atrovinosa	12,039	kingaensis	12,331	sparsa	13,487	coventryi	14,697	drakensbergensis	15,276	greyi	16,012	interrupta	14,906	extrusa	15,138	galioides	16,097	glabripes	11,921	lateriflora	12,316	modesta	13,065
seriphiifolia	13,335	savileae var. grandiflora	13,771	nudiflora	14,980	incarnata	12,039	aristifolia	12,271	boucheri	13,478	albertyniae	14,589	tysonii	15,276	globiceps subsp. consors	16,011	similis	14,906	nigrimontana	15,138	quadrisulcata	16,097	papyracea	11,921	longimontana	12,316	pubigera	13,065
madagascariensis	13,326	savileae var. mutica	13,771	umbellata	14,980	maderensis	12,039	haemastoma	12,271	phacelanthera	13,478	pulchelliflora	14,589	hansfordii	15,228	globiceps subsp. gracilis	16,011	ventricosa	14,889	schumannii	15,138	bodkinii	16,083	abietina subsp. atrorosea	11,799	magistrati	12,316	saxicola	13,065
astroites	13,268	savileae var. savileae	13,771	robynsiana	14,925	mira	12,039	taxifolia	12,271	angulosa	13,442	pleiotricha var. blaeriodes	14,477	hebeclada	15,159	afra	15,902	thimifolia	14,851	trichoclada	15,138	sociorum	16,080	annalis	11,799	onusta	12,316	phillipsii	13,041
chlorosepala	13,268	notholeeana	13,750	latiflora	14,850	physophylla	12,039	binaria	12,257	pumila	13,412	benthamiana	14,457	danguyana	15,139	turneri	15,902	abietina subsp. atrorosea	14,783	utriculosa	15,138	oresigena	16,049	grandiflora	11,799	patens	12,316	williamsiorum	13,002
pillansii	13,229	spinifera	13,750	tetralix	14,850	rhodopis	12,039	notholeeana	12,257	hansfordii	13,337	monadelphia	14,444	amicorum	15,123	bakeri	15,868	grandiflora	14,783	wildii	15,138	filialis	16,025	grandiflora subsp. grandiflora	11,799	planifolia	12,316	ericoides	12,960
pillansii subsp. fervida	13,229	fascicularis	13,747	tumida	14,850	urna-viridis	12,039	botryoides	12,221	pulvinata	13,313	psittacina	14,438	tenuifolia	15,096	pleiotricha	15,868	albertyniae	14,749	orientalis	15,109	hameriana	16,010	macowanii	11,799	pubescens	12,316	pleiotricha var. pleiotricha	12,884
pillansii subsp. pillansii	13,229	fascicularis var. fascicularis	13,747	coccinea subsp. coccinea	14,832	baroniana	11,982	permutata	12,221	stokoeanthus	13,260	pubigera	14,391	lanceolifera	15,083	pleiotricha var. pleiotricha	15,868	halicacaba	14,732	recta	15,109	cryptoclada	15,983	macowanii subsp. macowanii	11,799	recurvifolia	12,316	sparrmannii	12,833
sparrmannii	13,229	eriocephala	13,744	conspicua	14,832	abelii	11,967	cernua	12,196	banksii subsp. banksii	13,212	pilosiflora	14,390	annalis	14,998	alexandri	15,867	labialis	14,723	fontana	15,082	altevivens	15,975	regia	11,799	rudolfii	12,316	amalophylla	12,770
bojeri	13,208	paucifolia	13,744	conspicua subsp. conspicua	14,832	bergiana	11,967	columnaris	12,196	banksii subsp. purpurea	13,212	pilosiflora subsp. pilosiflora	14,390	brachycentra	14,980	alexandri subsp. acockii	15,867	cremea	14,708	cernua	15,068	cymosa subsp. cymosa	15,975	regia subsp. regia	11,799	tarantulae	12,316	cubitans	12,770
minutifolia	13,208	paucifolia subsp. ciliata	13,744	oblongiflora	14,832	chionophila	11,967	ferrea	12,196	plukenetii subsp. breviflora	13,212	pilosiflora subsp. purpurea	14,390	sicifolia	14,942	alexandri subsp. alexandri	15,867	pinea	14,708	flexistyla	15,068	humansdorpensis	15,868	unilateralis	11,788	thimifolia	12,316	eriocodon	12,770
parkeri	13,208	paucifolia subsp. paucifolia	13,744	quadrisulcata	14,832	clavisepala	11,967	flexistyla	12,196	lanipes	13,156	beatricis	14,352	corydalis	14,909	cedromontana	15,853	benguelensis	14,706	obliqua	15,068	parviflora	15,868	atrovinosa	11,764	trachysantha	12,316	limosa	12,770
perhispida	13,208	paucifolia subsp. squarrosa	13,744	australis	14,817	curviflora	11,967	glabripes	12,196	micrandra	13,156	dregei	14,335	cumuliflora	14,870	limnophila	15,853	benguelensis var. albescens	14,706	subimbricata	15,049	dianthifolia	15,867	blenna	11,764	turneri	12,316	oxycoccifolia	12,770
lanipes	13,194	vernicosa	13,744	galgebergensis	14,814	fascicularis	11,967	laeta	12,196	pleiotricha	13,156	madagascariensis	14,335	amidae	14,856	nivalis	15,853	benguelensis var. benguelensis	14,706	inflaticalyx	15,043	penduliflora	15,867	goatcheriana var. drakensteinensis	11,764	uysii	12,316	philippioides	12,770
pycnantha	13,194	viscaria subsp. pendula	13,726	bodkinii	14,799	fascicularis var. fascicularis	11,967	nana	12,196	pleiotricha var. pleiotricha	13,156	tetralix	14,314	jugicola	14,856	ovina	15,853	afrorum	14,698	rosacea subsp. glabrata	15,043	blaerioides subsp. blaerioides	15,842	taxifolia	11,764	vallis-fluminis	12,316	salteri	12,770
glandulosa subsp. fourcadei	13,182	intricata	13,714	richardii	14,799	glutinosa	11,967	occulta	12,196	esteriana subsp. swartbergensis	13,141	humidicola	14,257	sociorum	14,856	velutina	15,853	recurvata	14,652	forsteri	14,986	unilateralis	15,839	platycodon subsp. maderincola	11,751	zebrensis	12,316	totta	12,770
reunionensis	13,173	physophylla	13,714	passerina	14,796	kogelbergensis	11,967	patersonii	12,196	perplexa	13,141	jumellei	14,252	inaequalis	14,847	pannosa	15,829	limnophila	14,621	kingaensis	14,968	karwyderi	15,822	fascicularis	11,740	filipendula	12,309	zitzikammensis	12,770
trimera subsp. elgonensis	13,173	chionophila	13,641	humidicola	14,793	lasiocarpa	11,967	pilulifera	12,196	conspicua subsp. conspicua	13,108	austronyassana	14,247	longimontana	14,847	spinifera	15,825	turgida	14,599	gossypioides	14,964	umbellata	15,822	fascicularis var. fascicularis	11,740	filipendula subsp. filipendula	12,309	lananthera	12,763
purgatoriensis	13,172	clavisepala	13,641	hansfordii	14,775	mollis	11,967	vallis-aranearum	12,196	grandiflora	13,108	bolusiae	14,208	passerinoides	14,816	eriocephala	15,819	phillipsii	14,597	globulifera	14,925	annalis	15,810	haematosiphon	11,740	nana	12,309	walkeri	12,763
mauritiensis	13,142	goatcheriana var. goatcheriana	13,641	pilosiflora	14,772	reenensis	11,967	virginalis	12,196	macowanii	13,108	flanaganii	14,208	astroites	14,807	paucifolia	15,819	umbrosa	14,597	physophylla	14,911	intervallaris	15,798	dregei	11,730	cubica	12,299	aneimena	12,746
glabripes	13,085	mallotocalyx	13,641	pilosiflora subsp. pilosiflora	14,772	unicolor	11,967	longistyla	12,146	macowanii subsp. macowanii	13,108	ribisaria	14,208	lusitanica	14,799	paucifolia subsp. ciliata	15,819	jumellei	14,592	globiceps	14,906	reenensis	15,798	axilliflora	11,693	viguieri	12,213	cymosa	12,711
danguyana	13,065	mauritanica	13,641	ribisaria	14,744	unicolor subsp. unicolor	11,967	oliveri	12,146	massonii	13,108	multiflexuosa	14,175	subverticillaris	14,799	paucifolia subsp. paucifolia	15,819	rakotozafyana	14,592	globiceps subsp. consors	14,906	orthiocola	15,784	passerina	11,675	annectens	12,194	cymosa subsp. cymosa	12,711
cyathiformis	13,064	longistyla	13,518	solandri	14,744	brownleeae	11,938	priorii	12,146	quadrisulcata	13,108	barbigeroides	14,174	sparrmannii	14,768	paucifolia subsp. squarrosa	15,819	bombycina	14,586	globiceps subsp. gracilis	14,906	cyathiformis	15,723	stylaris	11,651	retorta	12,194	intricata	12,711
eburnea	13,064	oliveri	13,518	filipendula	14,742	humifusa	11,914	coccinea	12,096	regia subsp. mariae	13,108	dispar	14,174	polycoma	14,750	chlorosepala	15,800	eriocephala	14,586	interrupta	14,906	phaeocarpa	15,706	eglandulosa	11,645	albospicata	12,184	melanacme	12,711
whyteana	13,064	placentiflora	13,518	filipendula subsp. parva	14,742	obconica	11,914	karwyderi	12,096	squarrosa	13,108	ericoides	14,174	syngenesia	14,750	andevalensis	15,796	paucifolia	14,586	ventricosa	14,889	jugicola	15,668	leucoclada	11,645	articularis	12,184	oresigena	12,711
amicorum	13,049	ciliaris	13,465	goatcheriana var. drakensteinensi	14,656	ocellata	11,914	maritima	12,096	manipuliflora	13,106	alticola	14,109	viscaria subsp. pendula	14,735	kirstenii	15,767	paucifolia subsp. ciliata	14,586	thimifolia	14,851	perspicua subsp. latifolia	15,662	muscosa	11,645	brownleeae	12,160	trichophylla	12,711
hameriana	13,011	coccinea	13,465	hexandra	14,637	poculiflora	11,914	melastoma	12,096	vagans	13,106	banksii subsp. purpurea	14,078	lateralis	14,726	lithophila	15,767	paucifolia subsp. paucifolia	14,586	abietina subsp. atrorosea	14,783	armandiana	15,634	globulifera	11,634	galgebergensis	12,152	truncata	12,711
abietina subsp. abietina	12,923	transparens	13,431	collina	14,601	rupicola	11,914	melastoma subsp. melastoma	12,096	filipendula subsp. parva	13,083	collina	14,065	velitaris	14,726	taylorii	15,767	paucifolia subsp. squarrosa	14,586	grandiflora	14,783	venustiflora	15,606	gracilis	11,634	densata	12,108	occulta	12,687
abietina subsp. diabolis	12,923	humbertii	13,401	colorans	14,583	eglandulosa	11,905	melastoma subsp. minor	12,096	ioniana	13,063	florifera	14,065	verecunda	14,726	pillansii	15,761	radicans	14,586	albertyniae	14,749	venustiflora subsp. glandulosa	15,606	sitiens	11,634	ibityensis	12,084	russakiana	12,687
annalis	12,923	zeyheriana	13,399	prolata	14,575	remota	11,905	atrovinosa	12,039	thunbergii	13,031	baueri	14,050	coventryi	14,697	pillansii subsp. pillansii	15,761	radicans subsp. radicans	14,586	halicacaba	14,732	venustiflora subsp. venustiflora	15,606	cygnea	11,626	laevigata	12,046	trichroma	12,685
grandiflora subsp. perfoliosa	12,923	tubercularis	13,359	fairii	14,561	conspicua	11,842	incarnata	12,039	jananthus	12,981	baueri subsp. baueri	14,050	pulchelliflora	14,589	kingaensis	15,735	vernicosa	14,586	labialis	14,723	sparrmannii	15,580	pageana	11,626	perplexa	12,041	angulosa	12,589
situshiemalis	12,923	madagascariensis	13,326	physodes	14,526	cygnea	11,842	maderensis	12,039	krugeri	12,981	subcapitata	14,022	pleiotricha var. blaeriodes	14,477	cuscutiformis	15,709	lithophila	14,534	cremea	14,708	beatricis	15,576	supranubia	11,626	ampullacea	12,014	bruniades	12,589
xanthina	12,923	astroites	13,268	banksii subsp. purpurea	14,460	cyrilliflora	11,842	mira	12,039	glaphyra	12,978	oresbia	14,020	benthamiana	14,457	saxigena	15,656	taylorii	14,534	pinea	14,708	bergiana	15,538	ventricosa	11,626	intonsa	12,014	capitata	12,589
perspicua	12,921	pillansii	13,229	jasminiflora	14,460	fastigiata	11,842	physophylla	12,039	hillburttii	12,947	adelopetala	13,967	psittacina	14,438	schlechteri	15,637	oatesii	14,510	benguelensis	14,706	brownleeae	15,509	australis	11,526	viscaria subsp. macrosepala	11,980	crassisepala	12,589
perspicua subsp. perspicua	12,921	pillansii subsp. fervida	13,229	lageniformis	14,460	gossypioides	11,842	rhodopis	12,039	petricola	12,912	cincta	13,934	pubigera	14,391	stokoeanthus	15,637	imerinensis	14,470	benguelensis var. albescens	14,706	bojeri	15,441	insolitanthera	11,526	glabripes	11,921	filago	12,589
paludicola	12,902	pillansii subsp. pillansii	13,229	limosa	14,388	gysbertii	11,842	urna-viridis	12,039	eburnea	12,852	perspicua subsp. latifolia	13,929	pilosiflora	14,390	subimbricata	15,626	ardens	14,459	benguelensis var. benguelensis	14,706	minutifolia	15,441	armandiana	11,516	papyracea	11,921	longipedunculata	12,589
abietina subsp. atrorosea	12,890	sparrmannii	13,229	grandiflora	14,352	intonsa	11,842	baroniana	11,982	florifera	12,852	coccinea subsp. coccinea	13,895	pilosiflora subsp. pilosiflora	14,390	glabripes	15,617	lambertii	14,459	afrorum	14,698	bolusiae	15,433	bojeri	11,516	abietina subsp. atrorosea	11,799	lowryensis	12,589
viscaria subsp. pustulata	12,890	bojeri	13,208	grandiflora subsp. grandiflora	14,352	oblongiflora	11,842	abelii	11,967	amatolensis	12,822	recta	13,885	pilosiflora subsp. purpurea	14,390	amatolensis	15,581	monsoniana	14,459	recurvata	14,652	dominans	15,411	minutifolia	11,516	macowanii	11,799	permutata	12,589
fontana	12,849	minutifolia	13,208	adelopetala	14,350	pageana	11,842	bergiana	11,967	rosacea subsp. glabrata	12,787	fontana	13,857	beatricis	14,352	cyathiformis	15,566	coacervata	14,440	limnophila	14,621	insolitanthera	15,404	paludicola	11,513	macowanii subsp. macowanii	11,799	petiolaris	12,589
hanekomii	12,849	parkeri	13,208	cremea	14,277	seriphiifolia	11,842	chionophila	11,967	hexandra	12,767	macowanii	13,857	dregei	14,335	dodii	15,566	calcareophila	14,435	turgida	14,599	cedromontana	15,276	nabea	11,469	regia	11,799	saptouensis	12,589
leucotrachela	12,849	perhispida	13,208	leucoclada	14,259	turrisbabylonica	11,842	clavisepala	11,967	sonderiana	12,751	macowanii subsp. macowanii	13,857	madagascariensis	14,335	cavartica	15,502	schlechteri	14,405	phillipsii	14,597	ovina	15,276	cremea	11,445	regia subsp. regia	11,799	rivularis	12,530
leucotrachela subsp. leucotrachela	12,849	lanipes	13,194	pellucida	14,255	arborescens	11,801	fascicularis	11,967	subimbricata	12,751	ustulescens	13,838	tetralix	14,314	nevillei	15,500	swaziensis	14,405	umbrosa	14,597	reunionensis	15,247	croceovirens	11,445	unilateralis	11,788	spiculifolia	12,447
viscaria subsp. viscaria	12,818	glandulosa subsp. fourcadei	13,182	axilliflora	14,246	tristis	11,801	fascicularis var. fascicularis	11,967	denticulata	12,727	ampullacea	13,824	humidicola	14,257	sexfaria	15,482	kraussiana	14,394	jumellei	14,592	agglutinans	15,241	pyramidalis	11,445	atrovinosa	11,764	cristata	12,403
ampullacea	12,815	reunionensis	13,173	lananthera	14,206	ericoides	11,797	glutinosa	11,967	plena	12,727	intonsa	13,824	jumellei	14,252	irbyana	15,468	williamsiorum	14,376	rakotozafyana	14,592	mauritiensis	15,188	quadrisulcata	11,445	blenna	11,764	albescens	12,316
oxycoccifolia	12,815	trimera subsp. elgonensis	13,173	brachyphylla	14,120	pleiotricha var. blaeriodes	11,765	kogelbergensis	11,967	malmesburiensis	12,697	oxycoccifolia	13,824	austronyassana	14,247	brachialis	15,463	purgatoriensis	14,369	bombycina	14,586	abietina subsp. diabolis	15,156	flanaganii	11,420	goatcheriana var. drakensteinensis	11,764	atropurpurea	12,316
pinea	12,815	purgatoriensis	13,172	rufescens	13,963	alnea	11,685	lasiocarpa	11,967	pulchelliflora	12,697	pinea	13,824	bolusiae	14,208	carnea	15,443	brachyphylla	14,362	eriocephala	14,586	latiflora	15,156	maximilianii	11,369	taxifolia	11,764	cederbergensis	12,316
platycodon	12,782	mauritiensis	13,142	wendlandiana	13,963	cavartica	11,685	mollis	11,967	opulenta	12,682	andringitrensis	13,817	flanaganii	14,208	conferta	15,443	maximilianii	14,353	paucifolia	14,586	trichroma	15,142	burchelliana	11,359	platycodon subsp. maderincola	11,751	curvistyla	12,316
platycodon subsp. platycodon	12,782	glabripes	13,085	globiceps	13,881	ciliaris	11,685	reenensis	11,967	pellucida	12,652	viguieri	13,807	ribisaria	14,208	stylaris	15,418	nyassana	14,352	paucifolia subsp. ciliata	14,586	nigrimontana	15,138	exleeana	11,359	fascicularis	11,740	distorta	12,316
carnea	12,758	danguyana	13,065	globiceps subsp. consors	13,881	nabea	11,685	brownleeae	11,938	genistifolia	12,570	albens	13,790	multiflexuosa	14,175	azorica	15,411	rossii	14,347	paucifolia subsp. paucifolia	14,586	schumannii	15,138	spinifera	11,359	fascicularis var. fascicularis	11,740	eremioides	12,316
coarctata	12,758	cyathiformis	13,064	globiceps subsp. gracilis	13,881	versicolor	11,685	humifusa	11,914	rubiginosa	12,570	bracteolaris	13,790	barbigeroides	14,174	omninoglabra	15,411	alticola	14,338	paucifolia subsp. squarrosa	14,586	trichoclada	15,138	brownii	11,357	haematosiphon	11,740	eremioides subsp. eglandula	12,316
conferta	12,758	eburnea	13,064	labialis	13,880	oresbia	11,640	rupicola	11,914	shannonii	12,570	platycodon subsp. maderincola	13,790	ericoides	14,174	scoparia	15,411	cooperi	14,338	radicans	14,586	wildii	15,138	globiceps subsp. globiceps	11,357	dregei	11,730	eremioides subsp. eremioides	12,316
heleogena	12,743	whyteana	13,064	cinerea	13,872	lanceolifera	11,594	eglandulosa	11,905	astroites	12,501	platycodon subsp. platycodon	13,790	alticola	14,109	umbelliflora	15,411	gigantea	14,330	radicans subsp. radicans	14,586	recta	15,109	holosericea	11,263	axilliflora	11,693	eremioides subsp. pubescens	12,316
irrorata	12,733	hameriana	13,011	brachialis	13,868	agglutinans	11,515	remota	11,905	erasmia	12,501	coarctata	13,766	collina	14,065	shannonii	15,358	physodes	14,330	vernicosa	14,586	cernua	15,068	leucodesmia	11,263	stylaris	11,651	inaequalis	12,316
regia subsp. mariae	12,731	abietina subsp. abietina	12,923	holosericea	13,857	pilosiflora	11,515	conspicua	11,842	glauca	12,501	glandulipila	13,760	florifera	14,065	steinbergiana	15,343	coccinea subsp. uniflora	14,307	prolata	14,575	flexistyla	15,068	abietina subsp. diabolis	11,231	eglandulosa	11,645	inflaticalyx	12,316
albospicata	12,729	abietina subsp. diabolis	12,923	modesta	13,857	pilosiflora subsp. pilosiflora	11,515	cygnea	11,842	glauca var. glauca	12,501	feminarum	13,751	subcapitata	14,022	erigena	15,319	galpinii	14,282	lithophila	14,534	subimbricata	15,049	doliiformis	11,231	leucoclada	11,645	lateriflora	12,316
discolor variant A 'discolor'	12,676	annalis	12,923	alfredii	13,835	baueri	11,511	cyrilliflora	11,842	junonia	12,501	thamnoides	13,744	oresbia	14,020	inclusa	15,310	amidae	14,278	taylorii	14,534	inflaticalyx	15,043	fontana	11,231	muscosa	11,645	magistrati	12,316
retorta	12,656	grandiflora subsp. perfoliosa	12,923	chrysocodon	13,834	baueri subsp. baueri	11,511	fastigiata	11,842	junonia var. minor	12,501	heleophila	13,737	adelopetala	13,967	nabea	15,310	etheliae	14,278	oatesii	14,510	rosacea subsp. glabrata	15,043	innovans	11,200	globulifera	11,634	onusta	12,316
albertyniae	12,514	situshiemalis	12,923	aristifolia	13,824	cyathiformis	11,511	gossypioides	11,842	cremea	12,497	cunoniensis	13,718	cincta	13,934	odorata	15,310	leucosiphon	14,278	imerinensis	14,470	forsteri	14,986	ardens	11,196	gracilis	11,634	patens	12,316
pulchelliflora	12,514	xanthina	12,923	cabernetea	13,824	dianthifolia	11,511	gysbertii	11,842	leucotrachela	12,497	coruscans	13,665	perspicua subsp. latifolia	13,929	brownleeae	15,307	plumigera	14,247	lambertii	14,459	kingaensis	14,968	goatcheriana var. petrensis	11,196	sitiens	11,634	planifolia	12,316
subcapitata	12,484	perspicua	12,921	maderensis	13,819	penduliflora	11,511	intonsa	11,842	leucotrachela subsp. leucotrachela	12,497	cygnea	13,665	recta	13,885	cyrilliflora	15,304	tegulifolia	14,247	monsoniana	14,459	globulifera	14,925	lambertii	11,196	cygnea	11,626	pubescens	12,316
trichroma	12,396	perspicua subsp. perspicua	12,921	savileae	13,771	trichostigma	11,511	oblongiflora	11,842	barnettiana	12,445	pageana	13,665	fontana	13,857	cremea	15,286	lycopodiastrum	14,173	coacervata	14,440	interrupta	14,906	calcareophila	11,172	pageana	11,626	recurvifolia	12,316
monadelphia	12,370	paludicola	12,902	savileae var. grandiflora	13,771	ustulescens	11,461	pageana	11,842	filipendula subsp. filipendula	12,428	venustiflora	13,644	macowanii	13,857	croceovirens	15,286	triflora	14,173	calcareophila	14,435	maderi	14,859	vallis-gratiae	11,172	supranubia	11,626	rudolfii	12,316
junonia	12,357	abietina subsp. atrorosea	12,890	savileae var. mutica	13,771	azaleifolia	11,411	turrisbabylonica	11,842	propinqua	12,428	venustiflora subsp. glandulosa	13,644	macowanii subsp. macowanii	13,857	hibbertii	15,286	lateralis	14,149	schlechteri	14,405	thimifolia	14,851	heleogena	11,125	ventricosa	11,626	tarantulae	12,316
junonia var. junonia	12,357	viscaria subsp. pustulata	12,890	savileae var. savileae	13,771	glandulosa subsp. fourcadei	11,402	arborescens	11,801	alticola	12,339	venustiflora subsp. venustiflora	13,644	ustulescens	13,838	benguelensis var. albescens	15,283	verecunda	14,149	swaziensis	14,405	grandiflora	14,783	hillburttii	11,086	australis	11,526	thimifolia	12,316
junonia var. minor	12,357	fontana	12,849	spinifera	13,750	coarctata	11,386	tristis	11,801	kingaensis	12,331	tetragona	13,608	intonsa	13,824	afrorum	15,276	inclusa	14,077	kraussiana	14,394	albertyniae	14,749	cavartica	11,074	insolitanthera	11,526	trachysantha	12,316
cristata	12,342	hanekomii	12,849	fascicularis	13,747	dulcis	11,386	ericoides	11,797	barbigeroides	12,283	botryoides	13,551	oxycoccifolia	13,824	lananthera	15,270	coccinea subsp. coccinea	14,055	williamsiorum	14,376	halicacaba	14,732	cernua	11,067	armandiana	11,516	turneri	12,316
lanuginosa	12,342	leucotrachela	12,849	fascicularis var. fascicularis	13,747	erigena	11,386	pleiotricha var. blaeriodes	11,765	aristifolia	12,271	labialis	13,498	pinea	13,824	massonii	15,270	conspicua	14,053	purgatoriensis	14,369	labialis	14,723	ferrea	11,067	bojeri	11,516	uysii	12,316
abelii	12,318	leucotrachela subsp. leucotrachela	12,849	eriocephala	13,744	halicacaba	11,229	alnea	11,685	binaria	12,257	wendlandiana	13,427	andringitrensis	13,817	irrorata	15,265	glaphyra	14,043	brachyphylla	14,362	russakiana	14,713	flexistyla	11,067	minutifolia	11,516	vallis-fluminis	12,316
insolitanthera	12,318	viscaria subsp. viscaria	12,818	paucifolia	13,744	platycodon	11,229	cavartica	11,685	kraussiana	12,251	jananthus	13,408	viguieri	13,807	albospicata	15,260	tysonii	14,043	maximilianii	14,353	cremea	14,708	mallotocalyx	11,067	paludicola	11,513	zebrensis	12,316
pilosiflora subsp. purpurea	12,315	ampullacea	12,815	paucifolia subsp. ciliata	13,744	platycodon subsp. platycodon	11,229	ciliaris	11,685	cernua	12,196	cruenta	13,394	albens	13,790	articularis	15,260	placentiflora	14,020	nyassana	14,352	pinea	14,708	notoporina	11,067	nabea	11,469	nana	12,309
haemastoma	12,309	oxycoccifolia	12,815	paucifolia subsp. paucifolia	13,744	glabella	11,214	nabea	11,685	columnaris	12,196	arborea	13,337	bracteolaris	13,790	hebeclada	15,159	ciliaris	13,896	rossii	14,347	nevillei	14,707	physodes	11,067	cremea	11,445	cubica	12,299
taxifolia	12,309	pinea	12,815	paucifolia subsp. squarrosa	13,744	glabella subsp. glabella	11,214	oresbia	11,640	flexistyla	12,196	cinerea	13,337	platycodon subsp. maderincola	13,790	danguyana	15,139	versicolor	13,896	alticola	14,338	benguelensis	14,706	salicina	11,067	croceovirens	11,445	lasiocarpa	12,274
comorensis subsp. anjurensis	12,293	platycodon	12,782	vernicosa	13,744	glabella subsp. laevis	11,214	lanceolifera	11,594	nana	12,196	micrandra	13,337	platycodon subsp. platycodon	13,790	amicorum	15,123	lanceolifera	13,850	cooperi	14,338	benguelensis var. albescens	14,706	amidae	11,015	pyramidalis	11,445	solandri	12,274
filipendula subsp. filipendula	12,285	platycodon subsp. platycodon	12,782	intricata	13,714	phaeocarpa	11,214	agglutinans	11,515	occulta	12,196	garciae	13,322	coarctata	13,766	lanceolifera	15,083	humbertii	13,832	physodes	14,330	benguelensis var. benguelensis	14,706	discolor	11,015	quadrisulcata	11,445	viguieri	12,213
haematosiphon	12,285	carnea	12,758	inflata	13,676	adelopetala	11,179	pilosiflora	11,515	patersonii	12,196	hirtiflora	13,322	glandulipila	13,760	corydalis	14,909	annectens	13,825	galpinii	14,282	afrorum	14,698	excavata	11,015	flanaganii	11,420	annectens	12,194
bracteolaris	12,252	coarctata	12,758	alticola	13,655	albospicata	11,176	pilosiflora subsp. pilosiflora	11,515	pilulifera	12,196	modesta	13,322	feminarum	13,751	cristata	14,874	coruscans	13,825	amidae	14,278	macowanii	14,669	jugicola	11,015	burchelliana	11,359	brownleeae	12,160
georgica	12,252	heleogena	12,743	chionophila	13,641	pinea	11,157	cyathiformis	11,511	vallis-aranearum	12,196	straussiana	13,322	thamnoides	13,744	cumuliflora	14,870	ignita	13,825	etheliae	14,278	macowanii subsp. macowanii	14,669	leucosiphon	11,015	exleeana	11,359	galgebergensis	12,152
rhodopis	12,252	irrorata	12,733	clavisepala	13,641	russakiana	11,142	dianthifolia	11,511	virginalis	12,196	altevivens	13,288	heleophila	13,737	amidae	14,856	pilosiflora	13,813	leucosiphon	14,278	recurvata	14,652	cylindrica	10,961	spinifera	11,359	densata	12,108
rubens	12,252	regia subsp. mariae	12,731	mallotocalyx	13,641	hanekomii	11,106	penduliflora	11,511	oliveri	12,146	aristifolia	13,288	cunoniensis	13,718	sociorum	14,856	pilosiflora subsp. pilosiflora	13,813	plumigera	14,247	limnophila	14,621	eriophoros	10,944	brownii	11,357	ibityensis	12,084
columnaris	12,228	albospicata	12,729	mauritanica	13,641	milanjiana	11,106	trichostigma	11,511	priorii	12,146	cabernetea	13,288	coruscans	13,665	pumila	14,840	viguieri	13,807	tegulifolia	14,247	turgida	14,599	milanjiana	10,944	globiceps subsp. globiceps	11,357	perplexa	12,041
microdonta	12,219	retorta	12,656	blandfordii	13,619	australis	11,037	ustulescens	11,461	karwyderi	12,096	flavicoma	13,288	cygnea	13,665	taxifolia	14,840	platycodon subsp. maderincola	13,790	lycopodiastrum	14,173	phillipsii	14,597	velatiflora	10,944	holosericea	11,263	ampullacea	12,014
haematocodon	12,213	albertyniae	12,514	laeta	13,619	melanomontana	11,021	azaleifolia	11,411	maritima	12,096	ixanthera	13,288	pageana	13,665	haematosiphon	14,816	madagascariensis	13,757	triflora	14,173	umbrosa	14,597	physophylla	10,909	abietina subsp. diabolis	11,231	intonsa	12,014
mollis	12,213	pulchelliflora	12,514	oliveri	13,518	monadelphia	11,001	glandulosa subsp. fourcadei	11,402	melastoma subsp. minor	12,096	lycopodiastrum	13,288	tetragona	13,608	sessiliflora	14,816	sicifolia	13,710	lateralis	14,149	jumellei	14,592	selaginifolia	10,909	doliiformis	11,231	viscaria subsp. macrosepala	11,980
atrovinosa	12,199	subcapitata	12,484	placentiflora	13,518	chloroloma	10,872	coarctata	11,386	incarnata	12,039	oraria	13,288	botryoides	13,551	astroites	14,807	danguyana	13,677	verecunda	14,149	rakotozafyana	14,592	bergiana	10,885	fontana	11,231	papyracea	11,921
incarnata	12,199	trichroma	12,396	ciliaris	13,465	bombycina	10,860	dulcis	11,386	maderensis	12,039	triflora	13,288	labialis	13,498	leptopus	14,783	hexandra	13,677	coccinea subsp. coccinea	14,055	bombycina	14,586	verecunda	10,885	innovans	11,200	macowanii	11,799
macilenta	12,199	monadelphia	12,370	macowanii	13,404	eriocephala	10,860	erigena	11,386	baroniana	11,982	maderensis	13,283	wendlandiana	13,427	leucantha	14,783	parkeri	13,640	conspicua	14,053	eriocephala	14,586	lanceolifera	10,866	ardens	11,196	macowanii subsp. macowanii	11,799
multiflora	12,199	junonia	12,357	macowanii subsp. macowanii	13,404	paucifolia	10,860	halicacaba	11,229	abelii	11,967	tragomontana	13,241	jananthus	13,408	lutea	14,783	perhispida	13,640	glaphyra	14,043	paucifolia	14,586	barnettiana	10,851	goatcheriana var. petrensis	11,196	atrovinosa	11,764
urna-viridis	12,199	junonia var. junonia	12,357	humbertii	13,401	paucifolia subsp. ciliata	10,860	platycodon	11,229	chionophila	11,967	ardens	13,235	cruenta	13,394	rhodopis	14,783	cristata	13,626	tysonii	14,043	paucifolia subsp. ciliata	14,586	blaerioides subsp. blaerioides	10,695	lambertii	11,196	goatcheriana var. drakensteinensi	11,764
globulifera	12,179	junonia var. minor	12,357	pumila	13,394	paucifolia subsp. paucifolia	10,860	platycodon subsp. platycodon	11,229	clavisepala	11,967	grisbrookii	13,235	arborea	13,337	margaritacea	14,759	inaequalis	13,614	placentiflora	14,020	paucifolia subsp. paucifolia	14,586	cryptanthera	10,695	calcareophila	11,172	taxifolia	11,764
obliqua	12,179	cristata	12,342	intonsa	13,370	paucifolia subsp. squarrosa	10,860	glabella	11,214	fascicularis	11,967	lambertii	13,235	cinerea	13,337	syngenesia	14,750	longimontana	13,614	ciliaris	13,896	paucifolia subsp. squarrosa	14,586	cuscutiformis	10,695	vallis-gratiae	11,172	platycodon subsp. maderincola	11,751
jumellei	12,178	lanuginosa	12,342	nevillei	13,370	radicans	10,860	glabella subsp. glabella	11,214	fascicularis var. fascicularis	11,967	pectinifolia	13,219	micrandra	13,337	atrovinosa	14,730	glandulosa subsp. fourcadei	13,613	lanceolifera	13,850	radicans	14,586	cymosa subsp. grandiflora	10,695	heleogena	11,125	haematosiphon	11,740
simii	12,178	abelii	12,318	madagascariensis	13,326	radicans subsp. radicans	10,860	glabella subsp. laevis	11,214	kogelbergensis	11,967	strigilifolia	13,219	garciae	13,322	urna-viridis	14,730	ericoides	13,597	humbertii	13,832	radicans subsp. radicans	14,586	fimbriata	10,695	hillburttii	11,086	chloroloma	11,679
brownii	12,147	insolitanthera	12,318	astroites	13,268	vernicosa	10,860	phaeocarpa	11,214	mollis	11,967	fascicularis	13,211	hirtiflora	13,322	obliqua	14,711	acuta	13,518	annectens	13,825	vernicosa	14,586	limnophila	10,695	cavartica	11,074	stylaris	11,651
cerinthoides	12,134	pilosiflora subsp. purpurea	12,315	pillansii	13,229	viscosissima	10,860	adelopetala	11,179	rupicola	11,914	fascicularis var. fascicularis	13,211	modesta	13,322	coventryi	14,697	goatcheriana	13,518	coruscans	13,825	prolata	14,575	myriocodon	10,695	cernua	11,067	leucoclada	11,645
cerinthoides var. barbertona	12,134	haemastoma	12,309	pillansii subsp. fervida	13,229	borboniifolia	10,856	albospicata	11,176	eglandulosa	11,905	unicolor subsp. unicolor	13,211	straussiana	13,322	barrydalensis	14,658	polycoma	13,518	ignita	13,825	discolor variant C 'hebecalyx'	14,572	oreotragus	10,695	ferrea	11,067	cygnea	11,626
cerinthoides var. cerinthoides	12,134	taxifolia	12,309	pillansii subsp. pillansii	13,229	dodii	10,856	pinea	11,157	remota	11,905	physophylla	13,178	altevivens	13,288	glutinosa	14,658	sphaerocephala	13,518	pilosiflora	13,813	lithophila	14,534	wyliei	10,695	flexistyla	11,067	pageana	11,626
barrydalensis	12,126	comorensis subsp. anjurensis	12,293	squarrosa	13,211	duthieae	10,856	russakiana	11,142	fastigiata	11,842	alopecurus	13,113	aristifolia	13,288	maderi	14,658	irregularis	13,494	pilosiflora subsp. pilosiflora	13,813	taylorii	14,534	chrysocodon	10,671	mallotocalyx	11,067	supranubia	11,626
glutinosa	12,126	filipendula subsp. filipendula	12,285	bojeri	13,208	microdonta	10,847	hanekomii	11,106	pageana	11,842	holtii	13,113	cabernetea	13,288	notoporina	14,601	propinqua	13,494	viguieri	13,807	oatesii	14,510	dolfiana	10,671	notoporina	11,067	ventricosa	11,626
supranubia	12,126	haematosiphon	12,285	minutifolia	13,208	filago	10,803	milanjiana	11,106	turrisbabylonica	11,842	caterviflora	13,105	ixanthera	13,288	pleiotricha var. blaeriodes	14,477	hendricksei	13,461	platycodon subsp. maderincola	13,790	imerinensis	14,470	lignosa	10,671	physodes	11,067	insolitanthera	11,526
barbigeroides	12,099	rhodopis	12,252	parkeri	13,208	blenna	10,699	australis	11,037	arborescens	11,801	chionophila	13,105	lycopodiastrum	13,288	benthamiana	14,457	inflata	13,410	madagascariensis	13,757	albospicata	14,467	umbrosa	10,671	salicina	11,067	armandiana	11,516
dispar	12,099	rubens	12,252	perhispida	13,208	chlorosepala	10,699	melanomontana	11,021	tristis	11,801	clavisepala	13,105	triflora	13,288	pubigera	14,391	blenna	13,396	sicifolia	13,710	lambertii	14,459	discolor variant A 'discolor'	10,669	amidae	11,015	bojeri	11,516
cernua	12,069	columnaris	12,228	lanipes	13,194	eugenea	10,699	monadelphia	11,001	abietina subsp. atrorosea	11,767	goatcheriana var. goatcheriana	13,105	maderensis	13,283	pilosiflora	14,390	incarnata	13,396	danguyana	13,677	monsoniana	14,459	lanipes	10,531	excavata	11,015	minutifolia	11,516

ferrea	12,069	microdonta	12,219	glandulosa subsp. fourcadei	13,182	junonia var. junonia	10,699	chloroloma	10,872	pleiotricha var. blaeriodes	11,765	mallotocalyx	13,105	tragomontana	13,241	pilosiflora subsp. pilosiflora	14,390	grandiflora subsp. grandiflora	13,392	hexandra	13,677	calcareophila	14,435	afra	10,514	leucosiphon	11,015	paludicola	11,513
flexistyla	12,069	haematocodon	12,213	reunionensis	13,173	didymocarpa	10,684	bombycina	10,860	coccinea subsp. coccinea	11,717	zeyheriana	13,016	ardens	13,235	pilosiflora subsp. purpurea	14,390	malmesburiensis	13,356	parkeri	13,640	schlechteri	14,405	bicolor	10,514	cylindrica	10,961	nabea	11,469
tetragona	12,069	mollis	12,213	trimera subsp. elgonensis	13,173	goudotiana	10,684	eriocephala	10,860	grandiflora subsp. grandiflora	11,717	frigida	12,969	grisbrookii	13,235	beatricis	14,352	pulchelliflora	13,356	perhispida	13,640	swaziensis	14,405	botryoides	10,514	eriophoros	10,944	cremea	11,445
toringbergensis	12,069	atrovinosa	12,199	purgatoriensis	13,172	leucoclada	10,684	paucifolia	10,860	alnea	11,685	comorensis subsp. comorensis	12,945	pectinifolia	13,219	dregei	14,335	radicans subsp. schlechteri	13,356	cristata	13,626	williamsiorum	14,376	chionodes	10,514	milanjiana	10,944	croceovirens	11,445
cavartica	12,036	incarnata	12,199	mauritiensis	13,142	simii	10,684	paucifolia subsp. ciliata	10,860	cavartica	11,685	globiceps	12,944	strigilifolia	13,219	madagascariensis	14,335	grandiflora subsp. perfoliosa	13,354	inaequalis	13,614	purgatoriensis	14,369	coacervata	10,514	velatiflora	10,944	pyramidalis	11,445
thomensis	12,011	multiflora	12,199	danguyana	13,065	embothriifolia	10,649	paucifolia subsp. paucifolia	10,860	ciliaris	11,685	globiceps subsp. consors	12,944	unicolor subsp. unicolor	13,211	tetralix	14,314	situshiemalis	13,354	glandulosa subsp. fourcadei	13,613	brachyphylla	14,362	grata	10,514	physophylla	10,909	quadrisulcata	11,445
irbyana	12,003	urna-viridis	12,199	cyathiformis	13,064	multiflora	10,649	paucifolia subsp. squarrosa	10,860	nabea	11,685	globiceps subsp. gracilis	12,944	physophylla	13,178	humidicola	14,257	xanthina	13,354	ericoides	13,597	maximilianii	14,353	harveiana	10,514	bergiana	10,885	flanaganii	11,420
azorica	11,946	globulifera	12,179	whyteana	13,064	haematosiphon	10,626	radicans	10,860	oresbia	11,640	similis	12,944	alopecurus	13,113	jumellei	14,252	cinerea	13,337	acuta	13,518	nyassana	14,352	hirta	10,514	verecunda	10,885	burchelliana	11,359
hermani	11,946	obliqua	12,179	hameriana	13,011	inflata	10,626	radicans subsp. radicans	10,860	lanceolifera	11,594	densata	12,930	holtii	13,113	austronyassana	14,247	chionophila	13,266	goatcheriana	13,518	rossii	14,347	humansdorpensis	10,514	lanceolifera	10,866	exleeana	11,359
polifolia	11,946	jumellei	12,178	abietina subsp. abietina	12,923	minutifolia	10,612	vernicosa	10,860	agglutinans	11,515	bosseri	12,906	caterviflora	13,105	bolusiae	14,208	gracilis	13,266	polycoma	13,518	cooperi	14,338	lerouxiae	10,514	barnettiana	10,851	spinifera	11,359
scoparia	11,946	simii	12,178	abietina subsp. diabolis	12,923	alexandri	10,576	borboniifolia	10,856	pilosiflora	11,515	senilis	12,866	chionophila	13,105	collina	14,065	spiculifolia	13,254	sphaerocephala	13,518	physodes	14,330	marifolia	10,514	cryptanthera	10,695	brownii	11,357
comorensis subsp. comorensis	11,937	brownii	12,147	grandiflora subsp. perfoliosa	12,923	alexandri subsp. acockii	10,576	duthieae	10,856	pilosiflora subsp. pilosiflora	11,515	myriadenia	12,820	clavisepala	13,105	florifera	14,065	viscaria subsp. viscaria	13,249	irregularis	13,494	galpinii	14,282	tragulifera	10,514	cuscutiformis	10,695	pectinifolia	11,284
inclusa	11,844	cerinthoides var. barbertona	12,134	situshiemalis	12,923	alexandri subsp. alexandri	10,576	microdonta	10,847	cyathiformis	11,511	reunionensis	12,790	goatcheriana var. goatcheriana	13,105	oresbia	14,020	tragomontana	13,241	propinqua	13,494	amidae	14,278	filipendula subsp. parva	10,507	cymosa subsp. grandiflora	10,695	holosericea	11,263
nabea	11,844	glutinosa	12,126	xanthina	12,923	alfredii	10,574	filago	10,803	dianthifolia	11,511	trimera subsp. elgonensis	12,790	mallotocalyx	13,105	adelopetala	13,967	monadelphia	13,212	hendricksei	13,461	leucosiphon	14,278	eburnea	10,401	limnophila	10,695	abietina subsp. diabolis	11,231
curvifolia	11,840	supranubia	12,126	hanekomii	12,849	azorica	10,574	eugenea	10,699	penduliflora	11,511	wangfatiana	12,790	zeyheriana	13,016	perspicua subsp. latifolia	13,929	regia subsp. mariae	13,162	inflata	13,410	plumigera	14,247	gysbertii	10,393	myriocodon	10,695	doliiformis	11,231
aristata	11,820	barbigeroides	12,099	leucotrachela	12,849	gillii	10,574	junonia var. junonia	10,699	trichostigma	11,511	mauritiensis	12,760	frigida	12,969	williamsiorum	13,909	australis	13,119	incarnata	13,396	tegulifolia	14,247	miniscula	10,239	wyliei	10,695	fontana	11,231
coccinea subsp. coccinea	11,820	cernua	12,069	leucotrachela subsp. leucotrachela	12,849	scoparia	10,574	didymocarpa	10,684	ustulescens	11,461	chlorosepala	12,732	comorensis subsp. comorensis	12,945	recta	13,885	boutonii	13,102	grandiflora subsp. grandiflora	13,392	lycopodiastrum	14,173	bibax	10,178	chrysocodon	10,671	innovans	11,200
conspicua	11,820	ferrea	12,069	viscaria subsp. viscaria	12,818	comorensis subsp. comorensis	10,565	goudotiana	10,684	glandulosa subsp. fourcadei	11,402	pillansii	12,693	globiceps subsp. consors	12,944	ustulescens	13,838	tetralix	13,082	malmesburiensis	13,356	triflora	14,173	filamentosa	10,178	umbrosa	10,671	goatcheriana var. petrensis	11,196
conspicua subsp. conspicua	11,820	flexistyla	12,069	pinea	12,815	trimera subsp. keniensis	10,565	leucoclada	10,684	coarctata	11,386	pillansii subsp. fervida	12,693	globiceps subsp. gracilis	12,944	andringitrensis	13,817	valida	13,082	pulchelliflora	13,356	verecunda	14,149	kogelbergensis	10,178	discolor variant A 'discolor'	10,669	lambertii	11,196
croceovirens	11,820	toringbergensis	12,069	platycodon	12,782	robynsiana	10,526	simii	10,684	dulcis	11,386	pillansii subsp. pillansii	12,693	densata	12,930	viguieri	13,807	richardii	13,031	radicans subsp. schlechteri	13,356	glandulifera	14,133	regia subsp. mariae	10,178	lanipes	10,531	alopecurus	11,178
hibbertii	11,820	cavartica	12,036	platycodon subsp. platycodon	12,782	guthriei	10,524	embothriifolia	10,649	halicacaba	11,229	purgatoriensis	12,636	bosseri	12,906	albens	13,790	ingeana	12,976	grandiflora subsp. perfoliosa	13,354	hirtiflora	14,133	verticillata	10,178	bicolor	10,514	calcareophila	11,172
oblongiflora	11,820	thomensis	12,011	coarctata	12,758	hermani	10,524	multiflora	10,649	platycodon	11,229	trichostigma	12,603	senilis	12,866	platycodon subsp. maderincola	13,790	robynsiana	12,927	situshiemalis	13,354	urna-viridis	14,047	viscaria subsp. pendula	10,178	botryoides	10,514	vallis-gratiae	11,172
pyramidalis	11,820	irbyana	12,003	denticulata	12,634	zeyheriana	10,514	haematosiphon	10,626	platycodon subsp. platycodon	11,229	piquetbergensis	12,602	myriadenia	12,820	platycodon subsp. platycodon	13,790	alexandri	12,833	xanthina	13,354	glaphyra	14,043	colorans	10,119	coacervata	10,514	heleogena	11,125
quadrisulcata	11,820	azorica	11,946	albertyniae	12,514	aneimena	10,451	inflata	10,626	glabella subsp. laevis	11,214	isaloensis	12,559	reunionensis	12,790	coarctata	13,766	alexandri subsp. acockii	12,833	cinerea	13,337	haematosiphon	14,023	dianthifolia	10,119	grata	10,514	hillburttii	11,086
unicolor subsp. mutica	11,820	hermani	11,946	trichroma	12,396	jonasiana	10,449	minutifolia	10,612	phaeocarpa	11,214	glabripes	12,550	trimera subsp. elgonensis	12,790	glandulipila	13,760	alexandri subsp. alexandri	12,833	chionophila	13,266	placentiflora	14,020	fascicularis var. imperialis	10,119	harveiana	10,514	cavartica	11,074
rupicola	11,787	scoparia	11,946	monadelphia	12,370	collina	10,395	alexandri	10,576	adelopetala	11,179	amatolensis	12,514	wangfatiana	12,790	feminarum	13,751	velutina	12,819	spiculifolia	13,254	ciliaris	13,896	inflata	10,119	hirta	10,514	cernua	11,067
hansfordii	11,763	comorensis subsp. comorensis	11,937	junonia var. minor	12,357	fascicularis var. imperialis	10,395	alexandri subsp. acockii	10,576	erinus	11,125	miniscula	12,507	mauritiensis	12,760	heleophila	13,737	diaphana	12,801	viscaria subsp. viscaria	13,249	lanceolifera	13,850	cunoniensis	10,106	humansdorpensis	10,514	ferrea	11,067
viguieri	11,732	nabea	11,844	lanuginosa	12,342	rimarum	10,395	alexandri subsp. alexandri	10,576	hanekomii	11,106	perplexa	12,507	chlorosepala	12,732	cunoniensis	13,718	hottentotica	12,795	tragomontana	13,241	humbertii	13,832	discolor variant C 'hebecalyx'	9,920	lerouxiae	10,514	flexistyla	11,067
hebeclada	11,693	curvifolia	11,840	abelii	12,318	viridimontana	10,395	alfredii	10,574	milanjiana	11,106	nevillei	12,433	pillansii	12,693	annectens	13,698	humidicola	12,795	monadelphia	13,212	annectens	13,825	inordinata	9,920	marifolia	10,514	mallotocalyx	11,067
galpinii	11,624	aristata	11,820	insolitanthera	12,318	viridimontana subsp. nivicola	10,395	azorica	10,574	globiceps	11,053	innovans	12,414	pillansii subsp. fervida	12,693	coruscans	13,665	johnstoniana	12,795	regia subsp. mariae	13,162	coruscans	13,825	andringitrensis	9,895	tragulifera	10,514	salicina	11,067
venustiflora	11,569	coccinea subsp. coccinea	11,820	pilosiflora subsp. purpurea	12,315	viridimontana subsp. viridimontana	10,395	scoparia	10,574	globiceps subsp. consors	11,053	brachialis	12,395	pillansii subsp. pillansii	12,693	pageana	13,665	spinifera	12,790	australis	13,119	ignita	13,825	laeta	9,833	filipendula subsp. parva	10,507	amidae	11,015
venustiflora subsp. glandulosa	11,569	conspicua	11,820	taxifolia	12,309	pumila	10,344	comorensis subsp. comorensis	10,565	globiceps subsp. gracilis	11,053	carnea	12,376	purgatoriensis	12,636	labialis	13,498	brownii	12,731	boutonii	13,102	pilosiflora	13,813	pyxidiflora	9,833	eburnea	10,401	excavata	11,015
venustiflora subsp. venustiflora	11,569	conspicua subsp. conspicua	11,820	comorensis subsp. anjurensis	12,293	saptouensis	10,294	trimera subsp. keniensis	10,565	australis	11,037	conferta	12,376	trichostigma	12,603	wendlandiana	13,427	globiceps subsp. globiceps	12,731	tetralix	13,082	pilosiflora subsp. pilosiflora	13,813	abietina subsp. constantiana	9,824	gysbertii	10,393	leucosiphon	11,015
baueri	11,549	croceovirens	11,820	filipendula subsp. filipendula	12,285	burchelliana	10,285	robynsiana	10,526	chloroloma	10,872	halicacaba	12,275	piquetbergensis	12,602	jananthus	13,408	michellensis	12,708	valida	13,082	viguieri	13,807	coruscans	9,824	miniscula	10,239	eriophoros	10,944
baueri subsp. baueri	11,549	hibbertii	11,820	columnaris	12,228	hansfordii	10,270	guthriei	10,524	bombycina	10,860	steinbergiana	12,275	isaloensis	12,559	arborea	13,337	goatcheriana var. drakensteinensis	12,658	richardii	13,031	platycodon subsp. maderincola	13,790	ignita	9,824	maesta	10,205	milanjiana	10,944
dianthifolia	11,549	oblongiflora	11,820	microdonta	12,219	walkeri	10,270	hermani	10,524	eriocephala	10,860	erigena	12,251	glabripes	12,550	cinerea	13,337	grisbrookii	12,658	ingeana	12,976	madagascariensis	13,757	squarrosa	9,824	bibax	10,178	physophylla	10,909
fairii	11,549	pyramidalis	11,820	mollis	12,213	perspicua subsp. latifolia	10,113	zeyheriana	10,514	paucifolia	10,860	brownleeae	12,240	amatolensis	12,514	micrandra	13,337	pectinifolia	12,641	robynsiana	12,927	danguyana	13,677	chlorosepala	9,748	filamentosa	10,178	bergiana	10,885
penduliflora	11,549	quadrisulcata	11,820	atrovinosa	12,199	platycodon subsp. maderincola	10,113	aneimena	10,451	paucifolia subsp. ciliata	10,860	cyrilliflora	12,236	miniscula	12,507	garciae	13,322	strigilifolia	12,641	alexandri	12,833	hexandra	13,677	cincta	9,735	kogelbergensis	10,178	lanceolifera	10,866
perplexa	11,499	rupicola	11,787	incarnata	12,199	steinbergiana	10,113	jonasiana	10,449	paucifolia subsp. paucifolia	10,860	kogelbergensis	12,236	perplexa	12,507	modesta	13,322	brachialis	12,625	alexandri subsp. acockii	12,833	parkeri	13,640	abelii	9,724	regia subsp. mariae	10,178	barnettiana	10,851
verticillata	11,302	hansfordii	11,763	multiflora	12,199	aristata	10,040	collina	10,395	paucifolia subsp. squarrosa	10,860	turrisbabylonica	12,236	nevillei	12,433	altevivens	13,288	collina	12,603	alexandri subsp. alexandri	12,833	perhispida	13,640	danguyana	9,714	verticillata	10,178	pillansii subsp. pillansii	10,759
halicacaba	11,267	viguieri	11,732	globulifera	12,179	conspicua subsp. conspicua	10,040	fascicularis var. imperialis	10,395	radicans	10,860	lananthera	12,203	brachialis	12,395	aristifolia	13,288	lasiocarpa	12,603	velutina	12,819	glandulosa subsp. fourcadei	13,613	galioides	9,714	viscaria subsp. pendula	10,178	cryptanthera	10,695
erigena	11,243	hebeclada	11,693	jumellei	12,178	coruscans	10,040	rimarum	10,395	radicans subsp. radicans	10,860	massonii	12,203	carnea	12,376	cabernetea	13,288	permutata	12,592	diaphana	12,801	ericoides	13,597	parkeri	9,714	colorans	10,119	cuscutiformis	10,695
pellucida	11,243	galpinii	11,624	cerinthoides var. barbertona	12,134	croceovirens	10,040	viridimontana	10,395	vernicosa	10,860	irrorata	12,197	conferta	12,376	lycopodiastrum	13,288	andringitrensis	12,585	hottentotica	12,795	acuta	13,518	perhispida	9,714	dianthifolia	10,119	cymosa subsp. grandiflora	10,695
ecklonii	11,223	venustiflora	11,569	cernua	12,069	feminarum	10,040	viridimontana subsp. nivicola	10,395	duthieae	10,856	albospicata	12,193	halicacaba	12,275	triflora	13,288	alopecurus	12,536	humidicola	12,795	goatcheriana	13,518	collina	9,618	fascicularis var. imperialis	10,119	limnophila	10,695
lananthera	11,194	venustiflora subsp. glandulosa	11,569	flexistyla	12,069	grandiflora	10,040	viridimontana subsp. viridimontana	10,395	microdonta	10,847	articularis	12,193	steinbergiana	12,275	maderensis	13,283	holtii	12,536	johnstoniana	12,795	polycoma	13,518	cyrilliflora	9,610	inflata	10,119	myriocodon	10,695
plena	11,084	venustiflora subsp. venustiflora	11,569	cavartica	12,036	hibbertii	10,040	pumila	10,344	filago	10,803	whyteana	12,146	erigena	12,251	tragomontana	13,241	glandulipila	12,528	spinifera	12,790	irregularis	13,494	gossypioides	9,610	cunoniensis	10,106	chrysocodon	10,671
chloroloma	11,031	dianthifolia	11,549	thomensis	12,011	macowanii	10,040	saptouensis	10,294	eugenea	10,699	lateriflora	12,134	brownleeae	12,240	ardens	13,235	thamnoides	12,511	brownii	12,731	propinqua	13,494	hendricksei	9,610	andringitrensis	9,895	umbrosa	10,671
cubica	10,998	fairii	11,549	azorica	11,946	macowanii subsp. macowanii	10,040	hansfordii	10,270	junonia var. junonia	10,699	ignita	12,093	cyrilliflora	12,236	grisbrookii	13,235	cameronii	12,468	globiceps subsp. globiceps	12,731	hendricksei	13,461	trichophora	9,610	laeta	9,833	lanipes	10,531
dregei	10,869	penduliflora	11,549	hermani	11,946	massonii	10,040	walkeri	10,270	goudotiana	10,684	marojejyensis	12,006	kogelbergensis	12,236	strigilifolia	13,219	cavartica	12,468	michellensis	12,708	incarnata	13,396	turrisbabylonica	9,610	pyxidiflora	9,833	bicolor	10,514
globiceps	10,869	perplexa	11,499	scoparia	11,946	quadrisulcata	10,040	perspicua subsp. latifolia	10,113	leucoclada	10,684	robynsiana	11,932	turrisbabylonica	12,236	gracilis	13,139	fastigiata	12,432	goatcheriana var. drakensteinensis	12,658	grandiflora subsp. grandiflora	13,392	aspalathifolia	9,600	abietina subsp. constantiana	9,824	botryoides	10,514
globiceps subsp. consors	10,869	verticillata	11,302	comorensis subsp. comorensis	11,937	regia subsp. mariae	10,040	platycodon subsp. maderincola	10,113	multiflora	10,649	trichroma	11,860	lananthera	12,203	laeta	13,139	pageana	12,432	grisbrookii	12,658	radicans subsp. schlechteri	13,356	eustacei	9,600	coruscans	9,824	coacervata	10,514
globiceps subsp. gracilis	10,869	halicacaba	11,267	nabea	11,844	squarrosa	10,040	aristata	10,040	minutifolia	10,612	junonia var. minor	11,821	massonii	12,203	alopecurus	13,113	lawsonii	12,397	pectinifolia	12,641	grandiflora subsp. perfoliosa	13,354	algida	9,576	ignita	9,824	grata	10,514
similis	10,869	erigena	11,243	curvifolia	11,840	unicolor subsp. mutica	10,040	conspicua subsp. conspicua	10,040	alexandri	10,576	bodkinii	11,806	irrorata	12,197	holtii	13,113	octonaria	12,397	strigilifolia	12,641	situshiemalis	13,354	barrydalensis	9,551	squarrosa	9,824	harveiana	10,514
brachialis	10,856	pellucida	11,243	aristata	11,820	madagascariensis	10,031	coruscans	10,040	alexandri subsp. acockii	10,576	cristata	11,806	albospicata	12,193	chionophila	13,105	nabea	12,275	brachialis	12,625	xanthina	13,354	hameriana	9,551	chlorosepala	9,748	humansdorpensis	10,514
blenna	10,737	ecklonii	11,223	croceovirens	11,820	conferta	9,995	croceovirens	10,040	alexandri subsp. alexandri	10,576	lanuginosa	11,806	articularis	12,193	clavisepala	13,105	savileae	12,275	collina	12,603	cinerea	13,337	passerinoides	9,465	cincta	9,735	lerouxiae	10,514
caledonica	10,737	lananthera	11,194	hibbertii	11,820	plukenetii subsp. plukenetii	9,838	feminarum	10,040	azorica	10,574	richardii	11,806	whyteana	12,146	goatcheriana var. goatcheriana	13,105	savileae var. grandiflora	12,275	lasiocarpa	12,603	amatolensis	13,326	empetrina	9,438	abelii	9,724	marifolia	10,514
eugenea	10,737	plena	11,084	pyramidalis	11,820	amatolensis	9,755	grandiflora	10,040	scoparia	10,574	manipuliflora	11,787	lateriflora	12,134	mallotocalyx	13,105	savileae var. mutica	12,275	permutata	12,592	chionophila	13,266	abietina subsp. abietina	9,429	danguyana	9,714	tragulifera	10,514
sylvainiana	10,716	chloroloma	11,031	rupicola	11,787	calcicola	9,680	hibbertii	10,040	comorensis subsp. comorensis	10,565	vagans	11,787	ignita	12,093	comorensis subsp. comorensis	12,945	savileae var. savileae	12,275	andringitrensis	12,585	spiculifolia	13,254	grandiflora subsp. perfoliosa	9,429	galioides	9,714	eburnea	10,401
trimera	10,716	dregei	10,869	gysbertii	11,749	malmesburiensis	9,630	macowanii	10,040	trimera subsp. keniensis	10,565	abelii	11,782	marojejyensis	12,006	densata	12,930	conspicua subsp. conspicua	12,251	alopecurus	12,536	viscaria subsp. viscaria	13,249	hanekomii	9,429	parkeri	9,714	gysbertii	10,393
trimera subsp. trimera	10,716	globiceps	10,869	massonii	11,749	pulchelliflora	9,630	macowanii subsp. macowanii	10,040	robynsiana	10,526	pumila	11,773	robynsiana	11,932	bosseri	12,906	croceovirens	12,251	holtii	12,536	tragomontana	13,241	situshiemalis	9,429	perhispida	9,714	miniscula	10,239
wangfatiana	10,716	globiceps subsp. consors	10,869	viguieri	11,732	radicans subsp. schlechteri	9,630	massonii	10,040	guthriei	10,524	taxifolia	11,773	trichroma	11,860	senilis	12,866	curviflora	12,251	thamnoides	12,511	monadelphia	13,212	xanthina	9,429	collina	9,618	maesta	10,205
nana	10,713	globiceps subsp. gracilis	10,869	globiceps subsp. globiceps	11,706	coventryi	9,584	quadrisulcata	10,040	aneimena	10,451	filipendula	11,749	junonia var. minor	11,821	myriadenia	12,820	hibbertii	12,251	cameronii	12,468	australis	13,119	urna-viridis	9,394	cyrilliflora	9,610	filamentosa	10,178
kogelbergensis	10,698	brachialis	10,856	hebeclada	11,693	cylindrica	9,584	regia subsp. mariae	10,040	jonasiana	10,449	filipendula subsp. filipendula	11,749	bodkinii	11,806	reunionensis	12,790	quadrisulcata	12,251	cavartica	12,468	boutonii	13,102	unicolor subsp. unicolor	9,370	hendricksei	9,610	verticillata	10,178
saxigena	10,698	caledonica	10,737	galpinii	11,624	pellucida	9,584	squarrosa	10,040	collina	10,395	filipendula subsp. parva	11,749	cristata	11,806	trimera subsp. elgonensis	12,790	unicolor	12,251	fastigiata	12,432	tetralix	13,082	viscaria subsp. viscaria	9,323	trichophora	9,610	dianthifolia	10,119
forsteri	10,668	eugenea	10,737	venustiflora	11,569	benthamiana	9,424	madagascariensis	10,031	fascicularis var. imperialis	10,395	haematosiphon	11,749	lanuginosa	11,806	wangfatiana	12,790	unicolor subsp. mutica	12,251	pageana	12,432	richardii	13,031	caterviflora	9,265	turrisbabylonica	9,610	fascicularis var. imperialis	10,119
inflata	10,664	sylvainiana	10,716	venustiflora subsp. glandulosa	11,569	armandiana	9,376	amatolensis	9,755	rimarum	10,395	sessiliflora	11,749	richardii	11,806	mauritiensis	12,760	hebeclada	12,124	lawsonii	12,397	irrorata	13,009	chionophila	9,265	aspalathifolia	9,600	inflata	10,119
alticola	10,643	trimera	10,716	venustiflora subsp. venustiflora	11,569	boutonii	9,376	calcicola	9,680	viridimontana	10,395	leptopus	11,716	manipuliflora	11,787	pillansii subsp. fervida	12,693	modesta	12,089	octonaria	12,397	robynsiana	12,927	clavisepala	9,265	algida	9,576	cunoniensis	10,106
baccans	10,607	trimera subsp. trimera	10,716	dianthifolia	11,549	brachyphylla	9,344	malmesburiensis	9,630	viridimontana subsp. nivicola	10,395	leucantha	11,716	vagans	11,787	purgatoriensis	12,636	goatcheriana var. petrensis	12,002	nabea	12,275	alexandri	12,833	goatcheriana var. goatcheriana	9,265	hameriana	9,551	andringitrensis	9,895
blandfordii	10,607	wangfatiana	10,716	penduliflora	11,549	cryptoclada	9,344	pulchelliflora	9,630	viridimontana subsp. viridimontana	10,395	lutea	11,716	abelii	11,782	trichostigma	12,603	annalis	11,964	savileae	12,275	alexandri subsp. acockii	12,833	kirstenii	9,180	empetrina	9,438	laeta	9,833
dodii	10,607	nana	10,713	perplexa	11,499	trimera	9,344	radicans subsp. schlechteri	9,630	nevillei	10,376	palliiflora	11,716	pumila	11,773	piquetbergensis	12,602	brachycentra	11,946	savileae var. grandiflora	12,275	alexandri subsp. alexandri	12,833	lithophila	9,180	abietina subsp. abietina	9,429	pyxidiflora	9,833
duthieae	10,607	kogelbergensis	10,698	verticillata	11,302	trimera subsp. trimera	9,344	coventryi	9,584	saptouensis	10,294	rhodopis	11,716	taxifolia	11,773	isaloensis	12,559	hanekomii	11,889	savileae var. mutica	12,275	velutina	12,819	richardii	9,180	grandiflora subsp. perfoliosa	9,429	abietina subsp. constantiana	9,824
gracilis	10,607	saxigena	10,698	halicacaba	11,267	jumellei	9,294	cylindrica	9,584	walkeri	10,270	columnaris	11,692	haematosiphon	11,749	miniscula	12,507	mallotocalyx	11,873	savileae var. savileae	12,275	hottentotica	12,795	taylorii	9,180	hanekomii	9,429	coruscans	9,824
laeta	10,607	forsteri	10,668	erigena	11,243	kammanassieae	9,287	pellucida	9,584	annectens	10,195	margaritacea	11,692	sessiliflora	11,749	perplexa	12,507	mollis	11,837	conspicua subsp. conspicua	12,251	humidicola	12,795	beatricis	9,156	situshiemalis	9,429	ignita	9,824
pilulifera	10,607	inflata	10,664	ecklonii	11,223	klotzschii	9,287	armandiana	9,376	perspicua subsp. latifolia	10,113	atrovinosa	11,663	leptopus	11,716	halicacaba	12,275	cruenta	11,821	croceovirens	12,251	johnstoniana	12,795	oatesii	9,156	xanthina	9,429	squarrosa	9,824
regerminans	10,607	alticola	10,643	plena	11,084	alticola	9,271	boutonii	9,376	platycodon subsp. maderincola	10,113	goatcheriana var. drakensteinensis	11,663	leucantha	11,716	kogelbergensis	12,236	discolor	11,821	hibbertii	12,251	spinifera	12,790	tetralix	9,156	urna-viridis	9,394	chlorosepala	9,748
rimarum	10,607	baccans	10,607	chloroloma	11,031	mafiensis	9,221	brachyphylla	9,344	aristata	10,040	incarnata	11,663	lutea	11,716	turrisbabylonica	12,236	discolor variant B 'speciosa'	11,821	quadrisulcata	12,251	goatcheriana var. drakensteinensis	12,658	valida	9,156	unicolor subsp. unicolor	9,370	cincta	9,735
fascicularis var. imperialis	10,554	blandfordii	10,607	dregei	10,869	densata	9,219	cryptoclada	9,344	coruscans	10,040	urna-viridis	11,663	rhodopis	11,716	whyteana	12,146	excavata	11,821	hebeclada	12,124	grisbrookii	12,658	abbottii	9,050	viscaria subsp. viscaria	9,323	abelii	9,724
patersonii	10,554	duthieae	10,607	caledonica	10,737	barbigeroides	9,215	trimera	9,344	croceovirens	10,040	globulifera	11,643	columnaris	11,692	lateriflora	12,134	jugicola	11,821	modesta	12,089	strigilifolia	12,641	canescens	9,050	caterviflora	9,265	danguyana	9,714
piquetbergensis	10,527	laeta	10,607	eugenea	10,737	dispar	9,215	trimera subsp. trimera	9,344	feminarum	10,040	obliqua	11,643	margaritacea	11,692	ignita	12,093	maderensis	11,821	goatcheriana var. petrensis	12,002	collina	12,603	haematocodon	9,050	chionophila	9,265	galioides	9,714
trimera subsp. keniensis	10,508	pilulifera	10,607	sylvainiana	10,716	kraussiana	9,183	jumellei	9,294	hibbertii	10,040	cerinthoides var. barbertona	11,598	atrovinosa	11,663	marojejyensis	12,006	mafiensis	11,784	annalis	11,964	andringitrensis	12,585	ingeana	9,050	clavisepala	9,265	parkeri	9,714
isaloensis	10,485	rimarum	10,607	trimera	10,716	polifolia	9,183	kammanassieae	9,287	madagascariensis	10,031	barrydalensis	11,590	goatcheriana var. drakensteinensis	11,663	robynsiana	11,932	cerinthoides	11,759	hanekomii	11,889	alopecurus	12,536	schlechteri	9,050	goatcheriana var. goatcheriana	9,265	perhispida	9,714
perspicua subsp. latifolia	10,464	fascicularis var. imperialis	10,554	trimera subsp. trimera	10,716	mackayana	9,165	alticola	9,271	calcicola	9,680	colorans	11,590	incarnata	11,663	trichroma	11,860	cerinthoides var. cerinthoides	11,759	mallotocalyx	11,873	holtii	12,536	swaziensis	9,050	richardii	9,180	collina	9,618
macowanii	10,392	patersonii	10,554	wangfatiana	10,716	spinifera	9,064	mafiensis	9,221	radicans subsp. schlechteri	9,630	glutinosa	11,590	urna-viridis	11,663	junonia var. minor	11,821	sacciflora	11,751	mollis	11,837	cavartica	12,468	rufescens	8,999	beatricis	9,156	cyrilliflora	9,610
macowanii subsp. macowanii	10,392	piquetbergensis	10,527	nana	10,713	pilosiflora subsp. purpurea	9,058	densata	9,219	coventryi	9,584	maderi	11,590	obliqua	11,643	bodkinii	11,806	sparrmannii	11,734	excavata	11,821	fastigiata	12,432	wendlandiana	8,999	oatesii	9,156	hendricksei	9,610
pumila	10,382	trimera subsp. keniensis	10,508	kogelbergensis	10,698	baccans	9,054	barbigeroides	9,215	cylindrica	9,584	cernua	11,533	cerinthoides var. barbertona	11,598	lanuginosa	11,806	verticillata	11,734	jugicola	11,821	pageana	12,432	unicolor subsp. georgensis	8,945	tetralix	9,156	trichophora	9,610
intonsa	10,358	isaloensis	10,485	saxigena	10,698	baueri subsp. gouriquae	9,054	kraussiana	9,183	armandiana	9,376	flexistyla	11,533	barrydalensis	11,590	richardii	11,806	selaginifolia	11,716	maderensis	11,821	glutinosa	12,402	garciae	8,893	abbottii	9,050	turrisbabylonica	9,610
nevillei	10,358	perspicua subsp. latifolia	10,464	forsteri	10,668	blandfordii	9,054	mackayana	9,165	boutonii	9,376	notoporina	11,533	colorans	11,590	manipuliflora	11,787	bergiana	11,692	mafiensis	11,784	lawsonii	12,397	glandulifera	8,893	canescens	9,050	algida	9,576
andringitrensis	10,352	macowanii	10,392	baccans	10,607	ceraria	9,054	spinifera	9,064	brachyphylla	9,344	physodes	11,533	glutinosa	11,590	vagans	11,787	brownleeae	11,663	sacciflora	11,751	octonaria	12,397	hirtiflora	8,893	haematocodon	9,050	hameriana	9,551
sicula	10,348	macowanii subsp. macowanii	10,392	duthieae	10,607	foliacea	9,054	pilosiflora subsp. purpurea	9,058	cryptoclada	9,344	salicina	11,533	maderi	11,590	abelii	11,782	coventryi	11,659	sparrmannii	11,734	nabea	12,275	velutina	8,893	schlechteri	9,050	empetrina	9,438
sicula subsp. sicula	10,348	pumila	10,382	pilulifera	10,607	foliacea subsp. foliacea	9,054	baccans	9,054	trimera	9,344	interrupta	11,372	cernua	11,533	columnaris	11,692	lecomtei subsp. lecomtei	11,599	verticillata	11,734	savileae	12,275	penduliflora	8,886	swaziensis	9,050	hanekomii	9,429
innovans	10,339	intonsa	10,358	rimarum	10,607	foliacea subsp. fulgens	9,054	baueri subsp. gouriquae	9,054	trimera subsp. trimera	9,344	lecomtei	11,362	notoporina	11,533	goatcheriana var. drakensteinensis	11,663	pillansii	11,461	selaginifolia	11,716	savileae var. grandiflora	12,275	aestiva	8,869	rufescens	8,999	situshiemalis	9,429
platycodon subsp. maderincola	10,325	nevillei	10,358	fascicularis var. imperialis	10,554	galpinii	9,054	blandfordii	9,054	jumellei	9,294	sicula	11,357	physodes	11,533	incarnata	11,663	pillansii subsp. fervida	11,461	bergiana	11,692	savileae var. mutica	12,275	alexandri	8,869	unicolor subsp. georgensis	8,945	xanthina	9,429
feminarum	10,286	andringitrensis	10,352	patersonii	10,554	gracilis	9,054	ceraria	9,054	kammanassieae	9,287	sicula subsp. sicula	11,357	salicina	11,533	fairii	11,623	psittacina	11,404	brownleeae	11,663	savileae var. savileae	12,275	alexandri subsp. acockii	8,869	garciae	8,893	viscaria subsp. viscaria	9,323
cunoniensis	10,253	sicula	10,348	piquetbergensis	10,527	regerminans	9,054	foliacea	9,054	mafiensis	9,221	curvifolia	11,304	lecomtei	11,362	cerinthoides var. barbertona	11,598	lecomtei	11,362	coventryi	11,659	croceovirens	12,251	alexandri subsp. alexandri	8,869	glandulifera	8,893	caterviflora	9,265
abietina subsp. constantiana	10,199	sicula subsp. sicula	10,348	trimera subsp. keniensis	10,508	sacciflora	9,054	foliacea subsp. foliacea	9,054	densata	9,219	imerinensis	11,275	sicula	11,357	colorans	11,590	pilosiflora subsp. purpurea	11,356	lecomtei subsp. lecomtei	11,599	hibbertii	12,251	esterhuyseniae	8,869	hirtiflora	8,893	clavisepala	9,265
coruscans	10,199	innovans	10,339	isaloensis	10,485	unicolor subsp. georgensis	9,054	foliacea subsp. fulgens	9,054	mackayana	9,165	galpinii	11,088	sicula subsp. sicula	11,357	cernua	11,533	dregei	11,300	pillansii	11,461	hebeclada	12,124	gibbosa	8,869	velutina	8,893	goatcheriana var. goatcheriana	9,265
cygnea	10,199	platycodon subsp. maderincola	10,325	perspicua subsp. latifolia	10,464	placentiflora	8,954	galpinii	9,054	spinifera	9,064	conspicua subsp. roseoflora	10,894	curvifolia	11,304	physodes	11,533	tumida	11,280	pillansii subsp. fervida	11,461	modesta	12,089	hottentotica	8,869	penduliflora	8,886	richardii	9,180
pageana	10,199	feminarum	10,286	andringitrensis	10,352	gerhardii	8,929	sacciflora	9,054	pilosiflora subsp. purpurea	9,058	ecklonii	10,841	imerinensis	11,275	salicina	11,533	conspicua subsp. roseoflora	11,234	psittacina	11,404	goatcheriana var. petrensis	12,002	humidicola	8,869	aestiva	8,869	beatricis	9,156
squarrosa	10,199	cunoniensis	10,253	sicula	10,348	conspicua subsp. roseoflora	8,879	unicolor subsp. georgensis	9,054	baccans	9,054	williamsiorum	10,841	galpinii	11,088	lecomtei	11,362	ribisaria	11,174	lecomtei	11,362	hanekomii	11,889	johnstoniana	8,869	alexandri	8,869	oatesii	9,156
longiaristata	10,120	abietina subsp. constantiana	10,199	sicula subsp. sicula	10,348	platycalyx	8,722	placentiflora	8,954	baueri subsp. gouriquae	9,054	bibax	10,774	conspicua subsp. roseoflora	10,894	sicula	11,357	solandri	11,174	pilosiflora subsp. purpurea	11,356	mallotocalyx	11,873	psittacina	8,869	alexandri subsp. acockii	8,869	tetralix	9,156
rossii	9,761	coruscans	10,199	innovans	10,339	abietina subsp. atrorosea	8,700	gerhardii	8,929	blandfordii	9,054	globiceps subsp. globiceps	10,769	ecklonii	10,841	sicula subsp. sicula	11,357	colorans	11,013	dregei	11,300	mollis	11,837	scabriuscula	8,869	alexandri subsp. alexandri	8,869	abbottii	9,050
filamentosa	9,730	cygnea	10,199	platycodon subsp. maderincola	10,325	bibax	8,700	conspicua subsp. roseoflora	8,879	ceraria	9,054	octonaria	10,664	williamsiorum	10,841	curvifolia	11,304	maderi	11,013	tumida	11,280	excavata	11,821	senilis	8,869	gibbosa	8,869	haematocodon	9,050
cylindrica	9,622	pageana	10,199	feminarum	10,286	venustiflora	8,685	platycalyx	8,722	foliacea	9,054	annectens	10,631	bibax	10,774	imerinensis	11,275	cyrilliflora	11,004	conspicua subsp. roseoflora	11,234	maderensis	11,821	strigilifolia	8,629	hottentotica	8,869	swaziensis	9,050
denticulata	9,622	squarrosa	10,199	cunoniensis	10,253	venustiflora subsp. glandulosa	8,685	abietina subsp. atrorosea	8,700	foliacea subsp. foliacea	9,054	cylindrica	10,631	octonaria	10,664	galpinii	11,088	turrisbabylonica	11,004	ribisaria	11,174	mafiensis	11,784	massonii	8,591	humidicola	8,869	rufescens	8,999
mackayana	9,516	longiaristata	10,120	abietina subsp. constantiana	10,199	venustiflora subsp. venustiflora	8,685	bibax	8,700	foliacea subsp. fulgens	9,054	denticulata	10,631	annectens	10,631	conspicua subsp. roseoflora	10,894	russakiana	10,867	solandri	11,174	sacciflora	11,751	mackayana	8,494	johnstoniana	8,869	unicolor subsp. georgensis	8,945
densata	9,465	rossii	9,761	coruscans	10,199	coccinea subsp. coccinea	8,649	venustiflora	8,685	galpinii	9,054	gerhardii	10,631	cylindrica	10,631	ecklonii	10,841	nevillei	10,861	colorans	11,013	verticillata	11,734	betsileana	8,481	psittacina	8,869	garciae	8,893
spiculifolia	9,448	cylindrica	9,622	cygnea	10,199	grandiflora subsp. grandiflora	8,649	venustiflora subsp. glandulosa	8,685	sacciflora	9,054	walkeri	10,631	denticulata	10,631	bibax	10,774	macowanii	10,823	maderi	11,013	selaginifolia	11,716	trichostigma	8,385	scabriuscula	8,869	glandulifera	8,893
bosseri	9,441	denticulata	9,622	pageana	10,199	reunionensis	8,608	venustiflora subsp. venustiflora	8,685	unicolor subsp. georgensis	9,054	chloroloma	10,495	gerhardii	10,631	octonaria	10,664	macowanii subsp. macowanii	10,823	cyrilliflora	11,004	coventryi	11,659	propendens	8,377	senilis	8,869	velutina	8,893
myriadenia	9,354	mackayana	9,516	longiaristata	10,120	trimera subsp. elgonensis	8,608	coccinea subsp. coccinea	8,649	placentiflora	8,954	multiflora	10,272	walkeri	10,631	cylindrica	10,631	sicula	10,779	turrisbabylonica	11,004	lecomtei subsp. lecomtei	11,599	hebeclada	8,199	massonii	8,591	aestiva	8,869
interrupta	9,297	densata	9,465	rossii	9,761	ignita	8,468	grandiflora subsp. grandiflora	8,649	williamsiorum	8,950	blenna	10,201	glauca var. elegans	10,529	denticulata	10,631	sicula subsp. sicula	10,779	russakiana	10,867	pillansii	11,461	myriadenia	8,199	mackayana	8,494	alexandri	8,869
lecomtei	9,287	spiculifolia	9,448	cylindrica	9,622	lawsonii	8,468	reunionensis	8,608	gerhardii	8,929	caledonica	10,201	chloroloma	10,495	gerhardii	10,631	discolor variant C 'hebecalyx'	10,726	nevillei	10,861	pillansii subsp. fervida	11,461	viridimontana	8,103	betsileana	8,481	alexandri subsp. acockii	8,869
imerinensis	9,201	bosseri	9,441	mackayana	9,516	octonaria	8,468	trimera subsp. elgonensis	8,608	conspicua subsp. roseoflora	8,879	eugenea	10,201	multiflora	10,272	walkeri	10,631	feminarum	10,717	macowanii	10,823	psittacina	11,404	viridimontana subsp. nivicola	8,103	trichostigma	8,385	alexandri subsp. alexandri	8,869
trimera subsp. meruensis	9,128	myriadenia	9,354	densata	9,465	ecklonii	8,339	ignita	8,468	globiceps subsp. globiceps	8,877	lanipes	10,201	blenna	10,201	glauca var. elegans	10,529	unicolor subsp. georgensis	10,631	macowanii subsp. macowanii	10,823	lecomtei	11,362	viridimontana subsp. viridimontana	8,103	propendens	8,377	gibbosa	8,869
viridimontana	9,125	interrupta	9,297	spiculifolia	9,448	nyassana	8,108	lawsonii	8,468	platycalyx	8,722	pycnantha	10,201	caledonica	10,201	chloroloma	10,495	albospicata	10,621	sicula	10,779	pilosiflora subsp. purpurea	11,356	isaloensis	8,093	hebeclada	8,199	hottentotica	8,869
viridimontana subsp. nivicola	9,125	lecomtei	9,287	bosseri	9,441	tragomontana	8,072	octonaria	8,468	bibax	8,700	nana	10,177	eugenea	10,201	multiflora	10,272	botryoides	10,517	sicula subsp. sicula	10,779	dregei	11,300	cooperi	7,955	myriadenia	8,199	humidicola	8,869
viridimontana subsp. viridimontana	9,125	imerinensis	9,201	myriadenia	9,354	erinus	8,057	ecklonii	8,339	venustiflora	8,685	forsteri	10,132	lanipes	10,201	blenna	10,201	rufescens	10,393	discolor variant C 'hebecalyx'	10,726	tumida	11,280	frigida	7,955	viridimontana	8,103	johnstoniana	8,869
baueri subsp. gouriquae	9,092	trimera subsp. meruensis	9,128	interrupta	9,297	globiceps	7,985	nyassana	8,108	venustiflora subsp. glandulosa	8,685	perrieri	10,126	pycnantha	10,201	caledonica	10,201	wendlandiana	10,393	feminarum	10,717	conspicua subsp. roseoflora	11,234	lawsonii	7,808	viridimontana subsp. nivicola	8,103	psittacina	8,869
ceraria	9,092	viridimontana	9,125	lecomtei	9,287	globiceps subsp. consors	7,985	tragomontana	8,072	venustiflora subsp. venustiflora	8,685	baccans	10,071	nana	10,177	eugenea	10,201	barrydalensis	10,358	unicolor subsp. georgensis	10,631	ribisaria	11,174	leucotrachela subsp. monicae	7,808	viridimontana subsp. viridimontana	8,103	senilis	8,869
foliacea	9,092	viridimontana subsp. nivicola	9,125	imerinensis	9,201	globiceps subsp. gracilis	7,985	erinus	8,057	reunionensis	8,608	blandfordii	10,071	forsteri	10,132	lanipes	10,201	garciae	10,287	albospicata	10,621	solandri	11,174	sparrmannii	7,808	isaloensis	8,093	mackayana	8,494
foliacea subsp. foliacea	9,092	viridimontana subsp. viridimontana	9,125	trimera subsp. meruensis	9,128	similis	7,985	globiceps	7,985	trimera subsp. elgonensis	8,608	duthieae	10,071	perrieri	10,126	pycnantha	10,201	glandulifera	10,287	botryoides	10,517	colorans	11,013	varderi	7,798	cooperi	7,955	betsileana	8,481
foliacea subsp. fulgens	9,092	baueri subsp. gouriquae	9,092	viridimontana	9,125	spiculifolia	7,955	globiceps subsp. consors	7,985	ignita	8,468	eburnea	10,071	baccans	10,071	nana	10,177	hirtiflora	10,287	rufescens	10,393	cyrilliflora	11,004	irrorata	7,769	frigida	7,955	trichostigma	8,385
sacciflora	9,092	ceraria	9,092	viridimontana subsp. nivicola	9,125	rakotozafyana	7,953	globiceps subsp. gracilis	7,985	lawsonii	8,468	gracilis	10,071	blandfordii	10,071	forsteri	10,132	atrovinosa	10,201	wendlandiana	10,393	turrisbabylonica	11,004	glutinosa	7,750	lawsonii	7,808	propendens	8,377
unicolor subsp. georgensis	9,092	foliacea	9,092	viridimontana subsp. viridimontana	9,125	x flavisepala	7,859	spiculifolia	7,955	octonaria	8,468	laeta	10,071	duthieae	10,071	perrieri	10,126	taxifolia	10,201	glandulifera	10,287	sicula	10,779	maderi	7,750	leucotrachela subsp. monicae	7,808	hebeclada	8,199
vallis-aranearum	9,092	foliacea subsp. foliacea	9,092	baueri subsp. gouriquae	9,092	albertyniae	7,828	rakotozafyana	7,953	ecklonii	8,339	pilulifera	10,071	eburnea	10,071	baccans	10,071	urna-viridis	10,201	hirtiflora	10,287	sicula subsp. sicula	10,779	lecomtei subsp. lecomtei	7,685	sparrmannii	7,808	myriadenia	8,199
conspicua subsp. roseoflora	8,819	foliacea subsp. fulgens	9,092	ceraria	9,092	piquetbergensis	7,643	x flavisepala	7,859	nyassana	8,108	rimarum	10,071	gracilis	10,071	blandfordii	10,071	fascicularis	10,177	atrovinosa	10,201	feminarum	10,717	trimera subsp. jaegeri	7,663	varderi	7,798	viridimontana	8,103
steinbergiana	8,810	sacciflora	9,092	foliacea	9,092	bosseri	7,574	albertyniae	7,828	tragomontana	8,072	fascicularis var. imperialis	10,018	laeta	10,071	duthieae	10,071	fascicularis var. fascicularis	10,177	taxifolia	10,201	unicolor subsp. georgensis	10,631	trimera subsp. kilimanjarica	7,663	irrorata	7,769	viridimontana subsp. nivicola	8,103
praenitens	8,786	unicolor subsp. georgensis	9,092	foliacea subsp. foliacea	9,092	dregei	7,574	piquetbergensis	7,643	spiculifolia	7,955	hameriana	10,018	pilulifera	10,071	eburnea	10,071	haematosiphon	10,177	urna-viridis	10,201	botryoides	10,517	trimera subsp. meruensis	7,663	glutinosa	7,750	viridimontana subsp. viridimontana	8,103
cyrilliflora	8,771	vallis-aranearum	9,092	foliacea subsp. fulgens	9,092	humbertii	7,574	bosseri	7,574	rakotozafyana	7,953	patersonii	10,018	rimarum	10,071	pilulifera	10,071	unicolor subsp. unicolor	10,177	fascicularis	10,177	rufescens	10,393	trichroma	7,660	maderi	7,750	isaloensis	8,093
trichophora	8,771	conspicua subsp. roseoflora	8,819	sacciflora	9,092	myriadenia	7,574	dregei	7,574	x flavisepala	7,859	ibityensis	9,910	fascicularis var. imperialis	10,018	rimarum	10,071	clavisepala	10,071	fascicularis var. fascicularis	10,177	wendlandiana	10,393	pillansii subsp. fervida	7,535	lecomtei subsp. lecomtei	7,685	cooperi	7,955
turrisbabylonica	8,771	cyrilliflora	8,771	unicolor subsp. georgensis	9,092	glandulosa subsp. breviflora	7,554	humbertii	7,574	albertyniae	7,828	betsileana	9,717	hameriana	10,018	fascicularis var. imperialis	10,018	goatcheriana var. goatcheriana	10,071	haematosiphon	10,177	atrovinosa	10,201	saxigena	7,535	trimera subsp. jaegeri	7,663	frigida	7,955
williamsiorum	8,766	turrisbabylonica	8,771	vallis-aranearum	9,092	stagnalis subsp. minor	7,538	myriadenia	7,574	piquetbergensis	7,643	trimera subsp. keniensis	9,442	patersonii	10,018	hameriana	10,018	ibityensis	9,910	clavisepala	10,071	taxifolia	10,201	cristata	7,378	trimera subsp. kilimanjarica	7,663	lawsonii	7,808
gysbertii	8,737	williamsiorum	8,766	conspicua subsp. roseoflora	8,819	trimera subsp. meruensis	7,492	glandulosa subsp. breviflora	7,554	bosseri	7,574	nyassana	9,355	ibityensis	9,910	patersonii	10,018	densata	9,896	goatcheriana var. goatcheriana	10,071	fascicularis	10,177	glomiflora	7,378	trimera subsp. meruensis	7,663	leucotrachela subsp. monicae	7,808
massonii	8,737	gysbertii	8,737	cyrilliflora	8,771	isaloensis	7,419	stagnalis subsp. minor	7,538	dregei	7,574	x serratifolia	9,240	betsileana	9,717	ibityensis	9,910	bosseri	9,872	ibityensis	9,910	fascicularis var. fascicularis	10,177	lanuginosa	7,378	trichroma	7,660	varderi	7,798
globiceps subsp. globiceps	8,694	massonii	8,737	turrisbabylonica	8,771	prolata	7,381	trimera subsp. meruensis	7,492	humbertii	7,574	microdonta	9,226	trimera subsp. keniensis	9,442	betsileana	9,717	myriadenia	9,785	densata	9,896	clavisepala	10,071	michellensis	7,354	pillansii subsp. fervida	7,535	irrorata	7,769
ignita	8,627	globiceps subsp. globiceps	8,694	williamsiorum	8,766	nevillei	7,309	isaloensis	7,419	myriadenia	7,574	dulcis	9,202	nyassana	9,355	trimera subsp. keniensis	9,442	trimera subsp. abyssinica	9,770	bosseri	9,872	goatcheriana var. goatcheriana	10,071	tumida	7,354	cristata	7,378	glutinosa	7,750
marojejyensis	8,541	ignita	8,627	propendens	8,749	annectens	7,127	nevillei	7,309	glandulosa subsp. breviflora	7,554	leucotrachela subsp. monicae	9,202	x serratifolia	9,240	nyassana	9,355	pillansii subsp. pillansii	9,659	myriadenia	9,785	ibityensis	9,910	florifera	7,249	lanuginosa	7,378	maderi	7,750
coventryi	8,194	marojejyensis	8,541	ignita	8,627	leucotrachela subsp. monicae	7,127	annectens	7,127	stagnalis subsp. minor	7,538	trimera subsp. meruensis	9,128	microdonta	9,226	x serratifolia	9,240	isaloensis	9,525	trimera subsp. abyssinica	9,770	densata	9,896	lasiocarpa	7,249	michellensis	7,354	lecomtei subsp. lecomtei	7,685
dulcis	8,194	coventryi	8,194	marojejyensis	8,541	praenitens	7,127	leucotrachela subsp. monicae	7,127	trimera subsp. meruensis	7,492	mackayana	8,980	dulcis	9,202	microdonta	9,226	amatolensis	9,480	pillansii subsp. pillansii	9,659	bosseri	9,872	mollis	7,249	tumida	7,354	trimera subsp. jaegeri	7,663
perrieri	8,051	dulcis	8,194	coventryi	8,194	x cavendishiana	7,127	x cavendishiana	7,127	isaloensis	7,419	rossii	8,695	leucotrachela subsp. monicae	9,202	dulcis	9,202	x serratifolia	9,240	isaloensis	9,525	myriadenia	9,785	reenensis	7,249	florifera	7,249	trimera subsp. kilimanjarica	7,663
nyassana	7,964	perrieri	8,051	dulcis	8,194	terminalis	7,077	terminalis	7,077	brownii	7,305	viridimontana	8,589	trimera subsp. meruensis	9,128	leucotrachela subsp. monicae	9,202	bibax	9,202	amatolensis	9,480	trimera subsp. abyssinica	9,770	ribisaria	7,249	lasiocarpa	7,249	trimera subsp. meruensis	7,663
trimera subsp. jaegeri	7,892	nyassana	7,964	perrieri	8,051	x serratifolia	7,077	x serratifolia	7,077	leucotrachela subsp. monicae	7,127	viridimontana subsp. nivicola	8,589	mackayana	8,980	trimera subsp. meruensis	9,128	kogelbergensis	9,202	x serratifolia	9,240	pillansii subsp. pillansii	9,659	solandri	7,249	mollis	7,249	pillansii subsp. fervida	7,535
ibityensis	7,835	trimera subsp. jaegeri	7,892	nyassana	7,964	perrieri	6,717	perrieri	6,717	x cavendishiana	7,127	viridimontana subsp. viridimontana	8,589	rossii	8,695	mackayana	8,980	leucotrachela subsp. monicae	9,202	bibax	9,202	isaloensis	9,525	stokoei	7,249	reenensis	7,249	lanuginosa	7,378
x flavisepala	7,716	ibityensis	7,835	trimera subsp. jaegeri	7,892	marojejyensis	6,707	marojejyensis	6,707	terminalis	7,077	baueri subsp. gouriquae	8,556	viridimontana	8,589	rossii	8,695	irrorata	9,163	kogelbergensis	9,202	x serratifolia	9,240	forsteri	7,150	ribisaria	7,249	michellensis	7,354
glandulosa subsp. breviflora	7,676	x flavisepala	7,716	ibityensis	7,835	rossii	6,588	rossii	6,588	x serratifolia	7,077	ceraria	8,556	viridimontana subsp. nivicola	8,589	viridimontana	8,589	discolor variant A 'discolor'	9,105	leucotrachela subsp. monicae	9,202	bibax	9,202	cerinthoides	7,008	solandri	7,249	tumida	7,354
betsileana	7,642	glandulosa subsp. breviflora	7,676	x flavisepala	7,716	interrupta	6,413	interrupta	6,413	perrieri	6,717	dianthifolia	8,556	viridimontana subsp. viridimontana	8,589	viridimontana subsp. nivicola	8,589	saxigena	8,929	irrorata	9,163	kogelbergensis	9,202	cerinthoides var. cerinthoides	7,008	stokoei	7,249	florifera	7,249
prolata	7,381	betsileana	7,642	glandulosa subsp. breviflora	7,676	stagnalis	6,302	stagnalis	6,302	marojejyensis	6,707	fairii	8,556	ceraria	8,556	viridimontana subsp. viridimontana	8,589	abelii	8,748	saxigena	8,929	leucotrachela subsp. monicae	9,202	chloroloma	6,654	forsteri	7,150	mollis	7,249
bibax	7,309	bibax	7,309	betsileana	7,642	trimera subsp. jaegeri	6,256	trimera subsp. jaegeri	6,256	rossii	6,588	foliacea	8,556	dianthifolia	8,556	ceraria	8,556	cerinthoides var. barbertona	8,564	abelii	8,748	saxigena	8,929	macrocalyx	6,578	chloroloma	6,654	reenensis	7,249
lawsonii	7,198	lawsonii	7,198	bibax	7,309	imerinensis	6,151	imerinensis	6,151	interrupta	6,413	foliacea subsp. foliacea	8,556	fairii	8,556	dianthifolia	8,556	fascicularis var. imperialis	8,556	cerinthoides var. barbertona	8,564	abelii	8,748	coventryi	6,575	macrocalyx	6,578	ribisaria	7,249
octonaria	7,198	octonaria	7,198	lawsonii	7,198	lecomtei	6,151	lecomtei	6,151	stagnalis	6,302	foliacea subsp. fulgens	8,556	foliacea	8,556	foliacea	8,556	glutinosa	8,556	fascicularis var. imperialis	8,556	cerinthoides var. barbertona	8,564	robynsiana	6,544	coventryi	6,575	stokoei	7,249
terminalis	7,198	terminalis	7,198	octonaria	7,198	viguieri	6,151	viguieri	6,151	trimera subsp. jaegeri	6,256	penduliflora	8,556	foliacea subsp. foliacea	8,556	foliacea subsp. foliacea	8,556	macrocalyx	7,972	glutinosa	8,556	fascicularis var. imperialis	8,556	longiaristata	6,302	robynsiana	6,544	forsteri	7,150
annectens	7,165	annectens	7,165	terminalis	7,198	wangfatiana	6,151	wangfatiana	6,151	imerinensis	6,151	sacciflora	8,556	foliacea subsp. fulgens	8,556	foliacea subsp. fulgens	8,556	stagnalis	7,731	macrocalyx	7,972	macrocalyx	7,972	stagnalis	6,302	longiaristata	6,302	macrocalyx	6,578
gerhardii	7,165	gerhardii	7,165	annectens	7,165	macrocalyx	5,897	macrocalyx	5,897	lecomtei	6,151	vallis-aranearum	8,556	sacciflora	8,556	sacciflora	8,556	trimera subsp. meruensis	7,666	stagnalis	7,731	stagnalis	7,731	stagnalis subsp. minor	6,302	stagnalis	6,302	coventryi	6,575
walkeri	7,165	walkeri	7,165	gerhardii	7,165	williamsiorum	5,882	williamsiorum	5,882	viguieri	6,151	macrocalyx	7,972	vallis-aranearum	8,556	vallis-aranearum	8,556	stagnalis subsp. minor	7,538	trimera subsp. meruensis	7,666	trimera subsp. meruensis	7,666	pectinifolia	6,259	stagnalis subsp. minor	6,302	robynsiana	6,544
x serratifolia	7,165	x serratifolia	7,165	walkeri	7,165	betsileana	5,847	betsileana	5,847	wangfatiana	6,151	trimera subsp. jaegeri	7,892	macrocalyx	7,972	macrocalyx	7,972	mackayana	7,518	stagnalis subsp. minor	7,538	stagnalis subsp. minor	7,538	kingaensis subsp. bequaertii	6,213	pectinifolia	6,259	longiaristata	6,302
lecomtei subsp. lecomtei	6,744	lecomtei subsp. lecomtei	6,744	x serratifolia	7,165	danguyana	5,847	danguyana	5,847	macrocalyx	5,897	glandulosa subsp. breviflora	7,676	trimera subsp. jaegeri	7,892	trimera subsp. jaegeri	7,892	chloroloma	7,461	mackayana	7,518	mackayana	7,518	alopecurus	6,153	kingaensis subsp. bequaertii	6,213	stagnalis	6,302
trimera subsp. kilimanjarica	6,430	trimera subsp. kilimanjarica	6,430	lecomtei subsp. lecomtei	6,744	globiceps subsp. globiceps	5,810	globiceps subsp. globiceps	5,810	betsileana	5,847	prolata	7,381	glandulosa subsp. breviflora	7,676	glandulosa subsp. breviflora	7,676	prolata	7,381	chloroloma	7,461	chloroloma	7,461	holtii	6,153	alopecurus	6,153	stagnalis subsp. minor	6,302

barnettiana	6,406	barnettiana	6,406	trimera subsp. kilimanjarica	6,430
lyallii	6,120	lyallii	6,120	barnettiana	6,406
stagnalis	5,929	stagnalis	5,929	lyallii	6,120
leucotrachela subsp. monicae	5,737	leucotrachela subsp. monicae	5,737	stagnalis	5,929
propendens	5,737	propendens	5,737	leucotrachela subsp. monicae	5,737
stagnalis subsp. minor	5,737	stagnalis subsp. minor	5,737	stagnalis subsp. minor	5,737
x cavendishiana	5,737	x cavendishiana	5,737	x cavendishiana	5,737
lecomtei subsp. ravinakely	5,316	lecomtei subsp. ravinakely	5,316	lecomtei subsp. ravinakely	5,316
kingaensis subsp. bequaertii	5,067	kingaensis subsp. bequaertii	5,067	kingaensis subsp. bequaertii	5,067
trimera subsp. abyssinica	4,915	trimera subsp. abyssinica	4,915	trimera subsp. abyssinica	4,915
macrocalyx	4,506	macrocalyx	4,506	macrocalyx	4,506
glandulosa subsp. bondiae	4,463	glandulosa subsp. bondiae	4,463	glandulosa subsp. bondiae	4,463
baurii	3,762	baurii	3,762	baurii	3,762
sicula subsp. bocquetii	3,552	sicula subsp. bocquetii	3,552	sicula subsp. bocquetii	3,552
stagnalis subsp. stagnalis	2,524	stagnalis subsp. stagnalis	2,524	stagnalis subsp. stagnalis	2,524
granulosa	2,457	granulosa	2,457	granulosa	2,457
maesta	1,236	maesta	1,236	maesta	1,236
glauca var. elegans	1,095	glauca var. elegans	1,095	glauca var. elegans	1,095
kingaensis subsp. kingaensis	1,095	kingaensis subsp. kingaensis	1,095	kingaensis subsp. kingaensis	1,095
latituba	1,095	latituba	1,095	latituba	1,095
macowanii subsp. lanceolata	1,095	macowanii subsp. lanceolata	1,095	macowanii subsp. lanceolata	1,095
mannii	1,095	mannii	1,095	mannii	1,095
mannii subsp. mannii	1,095	mannii subsp. mannii	1,095	mannii subsp. mannii	1,095
mannii subsp. pallidiflora	1,095	mannii subsp. pallidiflora	1,095	mannii subsp. pallidiflora	1,095
mannii subsp. usambarensis	1,095	mannii subsp. usambarensis	1,095	mannii subsp. usambarensis	1,095
quadratiflora	1,095	quadratiflora	1,095	quadratiflora	1,095
x darleyensis	1,095	x darleyensis	1,095	x darleyensis	1,095
x griffithsii	1,095	x griffithsii	1,095	x griffithsii	1,095
x stuartii	1,095	x stuartii	1,095	x stuartii	1,095
x veitchii	1,095	x veitchii	1,095	x veitchii	1,095
x vinacea	1,095	x vinacea	1,095	x vinacea	1,095
x watsonii	1,095	x watsonii	1,095	x watsonii	1,095
x williamsii	1,095	x williamsii	1,095	x williamsii	1,095
natalensis	0	natalensis	0	natalensis	0

innovans	5,653	innovans	5,653	danguyana	5,847
mauritiensis	5,354	mauritiensis	5,354	innovans	5,653
forsteri	4,962	forsteri	4,962	mauritiensis	5,354
trimera subsp. abyssinica	4,915	trimera subsp. abyssinica	4,915	forsteri	4,962
trimera subsp. kilimanjarica	4,915	trimera subsp. kilimanjarica	4,915	trimera subsp. abyssinica	4,915
glandulosa subsp. bondiae	4,715	glandulosa subsp. bondiae	4,715	trimera subsp. kilimanjarica	4,915
brownii	4,238	brownii	4,238	glandulosa subsp. bondiae	4,715
kingaensis subsp. bequaertii	3,552	kingaensis subsp. bequaertii	3,552	kingaensis subsp. bequaertii	3,552
sicula subsp. bocquetii	3,552	sicula subsp. bocquetii	3,552	sicula subsp. bocquetii	3,552
ibityensis	3,270	ibityensis	3,270	ibityensis	3,270
maesta	3,038	maesta	3,038	maesta	3,038
lyallii	2,897	lyallii	2,897	lyallii	2,897
x vinacea	2,897	x vinacea	2,897	x vinacea	2,897
baurii	2,621	baurii	2,621	baurii	2,621
granulosa	2,457	granulosa	2,457	granulosa	2,457
lecomtei subsp. lecomtei	2,180	lecomtei subsp. lecomtei	2,180	lecomtei subsp. lecomtei	2,180
lecomtei subsp. ravinakely	2,180	lecomtei subsp. ravinakely	2,180	lecomtei subsp. ravinakely	2,180
glauca var. elegans	1,095	glauca var. elegans	1,095	glauca var. elegans	1,095
kingaensis subsp. kingaensis	1,095	kingaensis subsp. kingaensis	1,095	kingaensis subsp. kingaensis	1,095
latituba	1,095	latituba	1,095	latituba	1,095
macowanii subsp. lanceolata	1,095	macowanii subsp. lanceolata	1,095	macowanii subsp. lanceolata	1,095
mannii	1,095	mannii	1,095	mannii	1,095
mannii subsp. mannii	1,095	mannii subsp. mannii	1,095	mannii subsp. mannii	1,095
mannii subsp. pallidiflora	1,095	mannii subsp. pallidiflora	1,095	mannii subsp. pallidiflora	1,095
mannii subsp. usambarensis	1,095	mannii subsp. usambarensis	1,095	mannii subsp. usambarensis	1,095
quadratiflora	1,095	quadratiflora	1,095	quadratiflora	1,095
stagnalis subsp. stagnalis	1,095	stagnalis subsp. stagnalis	1,095	stagnalis subsp. stagnalis	1,095
x darleyensis	1,095	x darleyensis	1,095	x darleyensis	1,095
x griffithsii	1,095	x griffithsii	1,095	x griffithsii	1,095
x stuartii	1,095	x stuartii	1,095	x stuartii	1,095
x veitchii	1,095	x veitchii	1,095	x veitchii	1,095
x watsonii	1,095	x watsonii	1,095	x watsonii	1,095
x williamsii	1,095	x williamsii	1,095	x williamsii	1,095
natalensis	0	natalensis	0	natalensis	0

terminalis	7,198	prolata	7,381	prolata	7,381
x flavisepala	7,180	terminalis	7,198	terminalis	7,198
x cavendishiana	7,127	x flavisepala	7,180	x flavisepala	7,180
lecomtei subsp. lecomtei	6,744	x cavendishiana	7,127	x cavendishiana	7,127
trimera subsp. kilimanjarica	6,430	lecomtei subsp. lecomtei	6,744	lecomtei subsp. lecomtei	6,744
lyallii	6,120	trimera subsp. kilimanjarica	6,430	trimera subsp. kilimanjarica	6,430
stagnalis	5,929	lyallii	6,120	lyallii	6,120
stagnalis subsp. minor	5,737	stagnalis	5,929	stagnalis	5,929
lecomtei subsp. ravinakely	5,316	stagnalis subsp. minor	5,737	stagnalis subsp. minor	5,737
baurii	5,152	lecomtei subsp. ravinakely	5,316	lecomtei subsp. ravinakely	5,316
trimera subsp. abyssinica	4,915	baurii	5,152	baurii	5,152
glandulosa subsp. bondiae	4,463	trimera subsp. abyssinica	4,915	trimera subsp. abyssinica	4,915
kingaensis subsp. bequaertii	2,610	glandulosa subsp. bondiae	4,463	glandulosa subsp. bondiae	4,463
stagnalis subsp. stagnalis	2,524	kingaensis subsp. bequaertii	2,610	kingaensis subsp. bequaertii	2,610
maesta	1,236	stagnalis subsp. stagnalis	2,524	stagnalis subsp. stagnalis	2,524
glauca var. elegans	1,095	maesta	1,236	maesta	1,236
kingaensis subsp. kingaensis	1,095	kingaensis subsp. kingaensis	1,095	kingaensis subsp. kingaensis	1,095
latituba	1,095	latituba	1,095	latituba	1,095
macowanii subsp. lanceolata	1,095	macowanii subsp. lanceolata	1,095	macowanii subsp. lanceolata	1,095
mannii	1,095	mannii	1,095	mannii	1,095
mannii subsp. mannii	1,095	mannii subsp. mannii	1,095	mannii subsp. mannii	1,095
mannii subsp. pallidiflora	1,095	mannii subsp. pallidiflora	1,095	mannii subsp. pallidiflora	1,095
mannii subsp. usambarensis	1,095	mannii subsp. usambarensis	1,095	mannii subsp. usambarensis	1,095
quadratiflora	1,095	quadratiflora	1,095	quadratiflora	1,095
sicula subsp. bocquetii	1,095	sicula subsp. bocquetii	1,095	sicula subsp. bocquetii	1,095
x darleyensis	1,095	x darleyensis	1,095	x darleyensis	1,095
x griffithsii	1,095	x griffithsii	1,095	x griffithsii	1,095
x stuartii	1,095	x stuartii	1,095	x stuartii	1,095
x veitchii	1,095	x veitchii	1,095	x veitchii	1,095
x vinacea	1,095	x vinacea	1,095	x vinacea	1,095
x watsonii	1,095	x watsonii	1,095	x watsonii	1,095
x williamsii	1,095	x williamsii	1,095	x williamsii	1,095
granulosa	0	granulosa	0	granulosa	0
natalensis	0	natalensis	0	natalensis	0

viridimontana	7,127	viridimontana	7,127	viridimontana	7,127
viridimontana subsp. nivicola	7,127	viridimontana subsp. nivicola	7,127	viridimontana subsp. nivicola	7,127
viridimontana subsp. viridimontana	7,127	viridimontana subsp. viridimontana	7,127	viridimontana subsp. viridimontan	7,127
x cavendishiana	7,127	x cavendishiana	7,127	x cavendishiana	7,127
trimera subsp. jaegeri	6,430	trimera subsp. jaegeri	6,430	trimera subsp. jaegeri	6,430
trimera subsp. kilimanjarica	6,430	trimera subsp. kilimanjarica	6,430	trimera subsp. kilimanjarica	6,430
terminalis	5,737	terminalis	5,737	terminalis	5,737
lecomtei subsp. ravinakely	5,316	lecomtei subsp. ravinakely	5,316	lecomtei subsp. ravinakely	5,316
baurii	5,152	baurii	5,152	baurii	5,152
kingaensis subsp. bequaertii	5,067	kingaensis subsp. bequaertii	5,067	kingaensis subsp. bequaertii	5,067
glandulosa subsp. breviflora	4,641	glandulosa subsp. breviflora	4,641	glandulosa subsp. breviflora	4,641
sicula subsp. bocquetii	3,552	sicula subsp. bocquetii	3,552	sicula subsp. bocquetii	3,552
glandulosa subsp. bondiae	3,230	glandulosa subsp. bondiae	3,230	glandulosa subsp. bondiae	3,230
maesta	3,038	maesta	3,038	maesta	3,038
lyallii	2,897	lyallii	2,897	lyallii	2,897
x vinacea	2,897	x vinacea	2,897	x vinacea	2,897
stagnalis subsp. stagnalis	2,524	stagnalis subsp. stagnalis	2,524	stagnalis subsp. stagnalis	2,524
granulosa	2,457	granulosa	2,457	granulosa	2,457
glauca var. elegans	1,095	glauca var. elegans	1,095	glauca var. elegans	1,095
kingaensis subsp. kingaensis	1,095	kingaensis subsp. kingaensis	1,095	kingaensis subsp. kingaensis	1,095
latituba	1,095	latituba	1,095	latituba	1,095
macowanii subsp. lanceolata	1,095	macowanii subsp. lanceolata	1,095	macowanii subsp. lanceolata	1,095
mannii	1,095	mannii	1,095	mannii	1,095
mannii subsp. mannii	1,095	mannii subsp. mannii	1,095	mannii subsp. mannii	1,095
mannii subsp. pallidiflora	1,095	mannii subsp. pallidiflora	1,095	mannii subsp. pallidiflora	1,095
mannii subsp. usambarensis	1,095	mannii subsp. usambarensis	1,095	mannii subsp. usambarensis	1,095
quadratiflora	1,095	quadratiflora	1,095	quadratiflora	1,095
x darleyensis	1,095	x darleyensis	1,095	x darleyensis	1,095
x griffithsii	1,095	x griffithsii	1,095	x griffithsii	1,095
x stuartii	1,095	x stuartii	1,095	x stuartii	1,095
x veitchii	1,095	x veitchii	1,095	x veitchii	1,095
x watsonii	1,095	x watsonii	1,095	x watsonii	1,095
x williamsii	1,095	x williamsii	1,095	x williamsii	1,095
natalensis	0	natalensis	0	natalensis	0

trimera subsp. abyssinica	6,148	holtii	6,153	kingaensis subsp. bequaertii	6,213
prolata	6,145	trimera subsp. abyssinica	6,148	holtii	6,153
pillansii subsp. pillansii	5,733	prolata	6,145	trimera subsp. abyssinica	6,148
terminalis	5,733	pillansii subsp. pillansii	5,733	prolata	6,145
sicula	5,633	terminalis	5,733	terminalis	5,733
sicula subsp. sicula	5,633	sicula	5,633	sicula	5,633
lecomtei subsp. ravinakely	5,316	sicula subsp. sicula	5,633	sicula subsp. sicula	5,633
cerinthoides var. barbertona	4,638	lecomtei subsp. ravinakely	5,316	lecomtei subsp. ravinakely	5,316
glandulosa subsp. breviflora	4,638	cerinthoides var. barbertona	4,638	cerinthoides var. barbertona	4,638
x cavendishiana	4,500	glandulosa subsp. breviflora	4,638	glandulosa subsp. breviflora	4,638
glandulosa subsp. bondiae	3,035	x cavendishiana	4,500	x cavendishiana	4,500
lyallii	2,897	glandulosa subsp. bondiae	3,035	glandulosa subsp. bondiae	3,035
x vinacea	2,897	lyallii	2,897	lyallii	2,897
maesta	1,802	x vinacea	2,897	x vinacea	2,897
baurii	1,230	baurii	1,230	baurii	1,230
glauca var. elegans	1,095	glauca var. elegans	1,095	glauca var. elegans	1,095
kingaensis subsp. kingaensis	1,095	kingaensis subsp. kingaensis	1,095	kingaensis subsp. kingaensis	1,095
latituba	1,095	latituba	1,095	latituba	1,095
macowanii subsp. lanceolata	1,095	macowanii subsp. lanceolata	1,095	macowanii subsp. lanceolata	1,095
mannii	1,095	mannii	1,095	mannii	1,095
mannii subsp. mannii	1,095	mannii subsp. mannii	1,095	mannii subsp. mannii	1,095
mannii subsp. pallidiflora	1,095	mannii subsp. pallidiflora	1,095	mannii subsp. pallidiflora	1,095
mannii subsp. usambarensis	1,095	mannii subsp. usambarensis	1,095	mannii subsp. usambarensis	1,095
quadratiflora	1,095	quadratiflora	1,095	quadratiflora	1,095
sicula subsp. bocquetii	1,095	sicula subsp. bocquetii	1,095	sicula subsp. bocquetii	1,095
stagnalis subsp. stagnalis	1,095	stagnalis subsp. stagnalis	1,095	stagnalis subsp. stagnalis	1,095
x darleyensis	1,095	x darleyensis	1,095	x darleyensis	1,095
x griffithsii	1,095	x griffithsii	1,095	x griffithsii	1,095
x stuartii	1,095	x stuartii	1,095	x stuartii	1,095
x veitchii	1,095	x veitchii	1,095	x veitchii	1,095
x watsonii	1,095	x watsonii	1,095	x watsonii	1,095
x williamsii	1,095	x williamsii	1,095	x williamsii	1,095
granulosa	0	granulosa	0	granulosa	0
natalensis	0	natalensis	0	natalensis	0
