# Supplementary material for: ﻿An integrative approach to alpha taxonomy in Erica L. (Ericaceae) with three new species from the Western Cape, South Africa
Source: PhytoKeys. 2025 Jun 4;257:95–117. doi: 10.3897/phytokeys.257.139457 (PMC12159662; doi:10.3897/phytokeys.257.139457)
Supplement: Supplementary material 3 — Full phylogenetic data and results [file phytokeys-257-095_article-139457__-s003.zip › SUPPLEMENTARY_MATERIALS_PHYLOGENETIC-May2025/Phylogeny plots/Figure_S3.pdf]

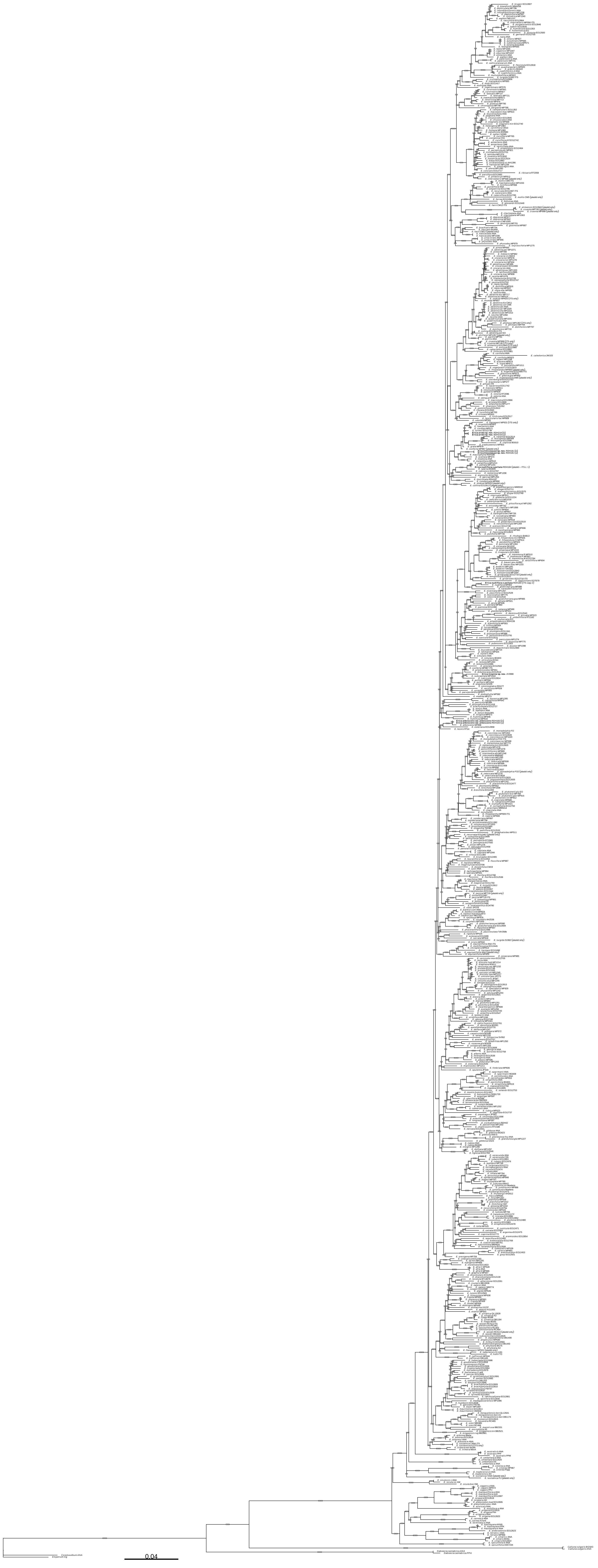

Figure S3: Complete phylogeny based on all locus alignments concatenated. Branch support values are SH−alrt/UFBoot %.
