# Supplementary material for: ﻿An integrative approach to alpha taxonomy in Erica L. (Ericaceae) with three new species from the Western Cape, South Africa
Source: PhytoKeys. 2025 Jun 4;257:95–117. doi: 10.3897/phytokeys.257.139457 (PMC12159662; doi:10.3897/phytokeys.257.139457)
Supplement: Supplementary material 4 — Summarised descriptions of new Erica taxa [file phytokeys-257-095_article-139457__-s004.docx]

**Supplementary Material 4: Summarised descriptions of new *Erica* taxa**

***Erica arida*** R.D.Hoekstra, sp. nov.

Rounded to semi-spreading, resprouting shrublet; leaves 3-nate, sparse, mostly glabrous, saddle-shaped, margins revolute; pedicel stipitate-glandular, viscid, light green to pink; bracts and bracteoles remote; sepals glandular, viscid; corolla white, open cup-shaped, 3.0-4.0mm long, glabrous, dry, lobes mostly erect, throat not constricted; anthers included, muticous, thecae unfused; style exserted, stigma subcapitate; ovary hispid.

Erica hessequae R.D.Hoekstra, sp. nov.

Semi-spreading, reseeding shrublet; leaves 3-nate, hispidulous, margins revolute; pedicel stipitate-glandular, red; bracts and bracteoles remote; sepals unfused, viscid, glandular; corolla open cup-shaped, ± 4mm long, white, glabrous, viscid, lobes recurved; anthers manifest, awned; style exserted, stigma subcapitate, ovary densely lanate.

***Erica inopina*** J.H.J.Vlok, sp. nov.

Erect, reseeding shrublet; leaves 3-nate, glabrous narrowly oblong; flowers 1-3 per inflorescence, pseudo-axillary in axils of upper leaves; pedicel glabrous; bract and bracteoles glabrous, fully recaulescent; sepals glabrous; corolla yellow-green, glabrous; anthers 6, partially exserted, muticous; style exserted, stigma peltate; ovary glabrous with 3 weakly developed locules.
